# Enantioselective Nickel‐Catalyzed *anti*‐Arylmetallative Cyclizations onto Acyclic Ketones

**DOI:** 10.1002/chem.202100143

**Published:** 2021-03-05

**Authors:** Harley Green, Stephen P. Argent, Hon Wai Lam

**Affiliations:** ^1^ The GlaxoSmithKline Carbon Neutral Laboratories for, Sustainable Chemistry University of Nottingham Jubilee Campus, Triumph Road Nottingham NG7 2TU UK; ^2^ School of Chemistry University of Nottingham University Park Nottingham NG7 2RD UK

**Keywords:** asymmetric catalysis, cyclization, isomerization, ketones, nickel

## Abstract

Domino reactions involving nickel‐catalyzed additions of (hetero)arylboronic acids to alkynes, followed by cyclization of the alkenylnickel intermediates onto tethered acyclic ketones to give chiral tertiary‐alcohol‐containing products in high enantioselectivities, are described. The reversible *E*/*Z* isomerization of the alkenylnickel intermediates enables overall *anti*‐arylmetallative cyclization to occur. The ring system of the products are substructures of certain diarylindolizidine alkaloids.

## Graphical abstract

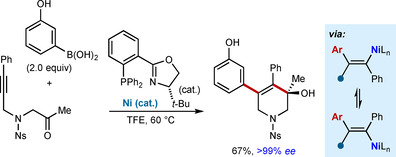

Stereogenic cyclic tertiary alcohols are important structural units that feature prominently in biologically active natural products and therapeutic compounds. Accordingly, new methods for the enantioselective construction of these units provide valuable tools for target synthesis. Of the strategies available, the catalytic asymmetric addition of carbon nucleophiles to ketones ranks highly in directness, versatility, and overall synthetic efficiency.^[1]^ One subset of these reactions are metal‐catalyzed domino sequences initiated by the addition of an arylboron reagent to an alkyne, followed by enantioselective cyclization of the resulting alkenylmetal species onto a tethered ketone, which are applicable to the synthesis of diverse carbo‐ and heterocycles.^[2]^ Recently, nickel‐catalyzed variants of these reactions have been developed in which reversible *E*/*Z* isomerization of the alkenylnickel intermediates is essential for cyclization.^[3]^ Application of this general method to achiral products has also been reported,^[4]^ and other related nickel‐catalyzed processes have also appeared.[^5^, ^6^, ^7^] In addition to nickel being much less expensive than comparable rhodium‐ or palladium‐catalyzed reactions,^[2]^ the diverse reactivity of nickel catalysis^[5]^ often enables unique transformations not available to other metal catalysts.

Our first contribution to this field included a study of enantioselective nickel‐catalyzed desymmetrizations of cyclic 1,3‐diketones, which give fused bicycles in high diastereo‐ and enantioselectivities (Scheme 1 A).^[3a]^ Although effective, the ability to use acyclic ketones in non‐desymmetrizing cyclizations would also be valuable to significantly broaden the substrate scope and provide simpler, non‐fused products. However, acyclic ketones are potentially less reactive than cyclic 1,3‐diketones because of their greater conformational flexibility, and because they lack the activation from the second ketone through its electron‐withdrawing effect as well as the electronic repulsion caused by having aligned dipoles. Two individual examples of non‐asymmetric nickel‐catalyzed arylative cyclizations onto acyclic ketones have been reported recently,^[6d]^ but to our knowledge, corresponding enantioselective processes have yet to be described.

**Scheme 1:**
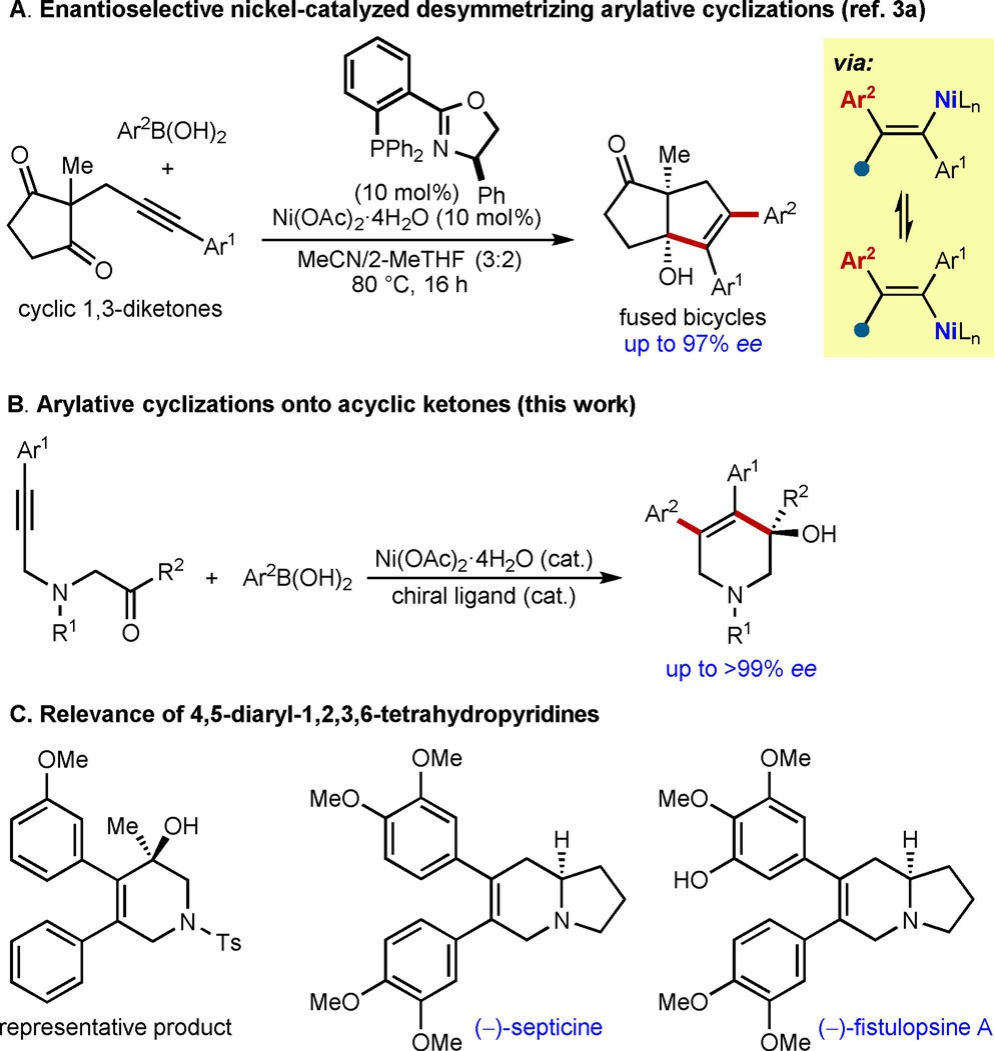
Enantioselective nickel‐catalyzed arylative cyclizations onto ketones.

Herein, we describe the successful use of acyclic dialkyl and alkyl‐aryl ketones in these reactions in the enantioselective preparation of aza‐ and carbocyclic tertiary alcohols (Scheme 1 B). Most of the products contain the 4,5‐diaryl‐1,2,3,6‐tetrahydropyridine ring system, which appears in indolizidine natural products such as (−)‐phyllosteminine,^[8]^ (−)‐septicine,^[9]^ and (−)‐fistulopsine A^[10]^ (Scheme 1 C).

This investigation began with the reaction of PhB(OH)_2_ with acyclic substrates **1**, which contain an alkyne tethered to a ketone through a sulfonamide (Table 1). Chiral phosphine‐oxazoline (PHOX) ligands have proven to be excellent ligands in related studies^[3]^ and we found (*S*)‐*t*Bu‐PHOX (**L1**) to be highly effective in these reactions. Heating a mixture of the substrate **1** and PhB(OH)_2_ (2.0 equiv) in the presence of 10 mol % each of Ni(OAc)_2_⋅4 H_2_O and **L1** in TFE (2,2,2‐trifluoroethanol) at 60 or 80 °C for 24 h provided azacycles **2 a**‐**2 k** in 44–90 % yield and up to 99 % *ee*.^[11]^ Small quantities of minor arylative cyclization products **3**, resulting from initial phenylnickelation of the alkyne with the regioselectivity opposite to that required for the formation of the major products **2**, were also observed by ^1^H NMR spectroscopy, but with the exception of the reaction producing **2 k** and **3 k**, these were not isolated. A range of aromatic ketones are tolerated in these reactions, with substrates containing phenyl (**2 a**), 4‐chlorophenyl (**2 b**), (3‐trifluoromethyl)phenyl (**2 c**), or 2‐methylphenyl ketones (**2 d**) readily undergoing arylative cyclization. Simple dialkyl ketones are also competent electrophiles, with ketones containing methyl (**2 e**, **2 f**, **2 i**, and **2 j**), ethyl (**2 k**), isopropyl (**2 g**), or methyl propanoate (**2 h**) groups reacting successfully. Regarding the alkynyl substituent, the process is tolerant of phenyl (**2 a**‐**2 h**), (4‐carbomethoxy)phenyl (**2 i**), and 3‐methoxyphenyl groups (**2 j**). A substrate with a vinyl group on the alkyne also reacted smoothly, but the enantiomeric excess of the resulting product **2 k** (45 % *ee*) was lower than in the other cases. Replacing the *para*‐toluenesulfonamide with a 4‐nitrophenylsulfonamide group is also possible (**2 f**), which has implications for subsequent product manipulation because 4‐nitrophenylsulfonyl groups are more readily deprotected than tosyl groups.

**Table 1:** Scope of alkynyl‐tethered ketones.^[a]^

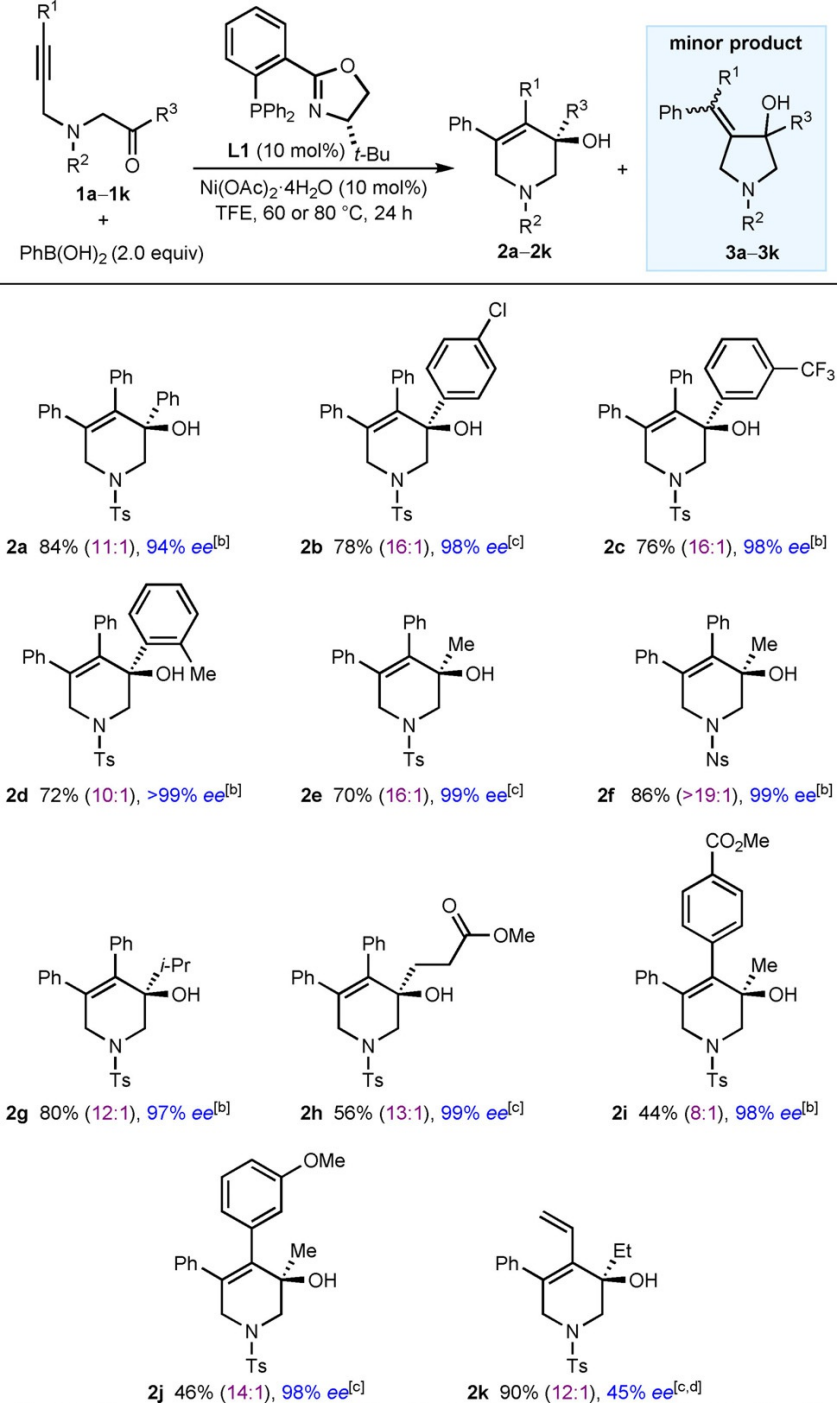

[a] Reactions were conducted using 0.30 mmol of **1** in TFE (3 mL). Yields are of isolated products. Values in parentheses refer to the ratio of **2**:**3** as determined by ^1^H NMR analysis of the crude reactions. Unless stated otherwise, the minor isomers **3** were not evident in the isolated products. Enantiomeric excesses were determined by HPLC analysis on a chiral stationary phase. [b] At 80 °C. [c] At 60 °C. [d] Product **2 k** was obtained as an inseparable 12:1 mixture together with the minor product **3 k** in 90 % combined yield.

Next, different boronic acids were investigated in reactions with substrate **1 f**, and we were pleased to observe that arylative cyclization products **2 l**‐**2 p** were obtained in up to 79 % yield and uniformly high enantioselectivities (98 % to >99 % *ee*, Table 2). Various substituted phenylboronic acids are compatible with this process including, notably, 3‐hydroxyphenylboronic acid (**2 m**). 2‐Naphthylboronic acid (**2 o**) and 3‐thienylboronic acid (**2 p**) also readily underwent the reaction.

**Table 2:** Scope of boronic acids.^[a]^

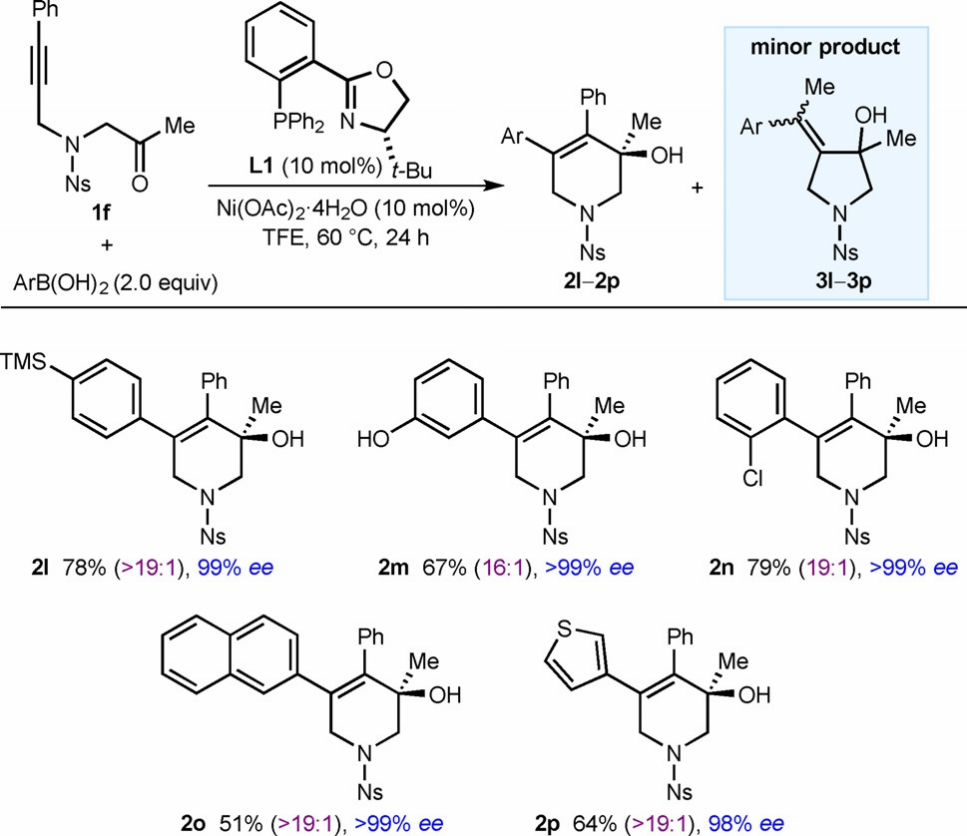

[a] Reactions were conducted using 0.30 mmol of **1 f** in TFE (3 mL). Yields are of isolated products. Values in parentheses refer to the ratio of **2**:**3** as determined by ^1^H NMR analysis of the crude reactions. Unless stated otherwise, the minor isomers **3** were not evident in the isolated products. Enantiomeric excesses were determined by HPLC analysis on a chiral stationary phase.

Further experiments to explore the scope of this process in the reactions of various other substrates with PhB(OH)_2_ are shown in Schemes 2, 3, 4, 5, 6. Changing the alkynyl substituent to a chloride was only moderately successful; substrate **1 l** reacted to give chloroalkene‐containing tetrahydropyridine **2 q** in 12 % yield and 71 % *ee*, with the remainder of the material being predominantly unreacted **1 l** (Scheme 2). Next, the preparation of carbocyclic products was attempted by changing the sulfonamide connecting the alkyne and the ketone to a malonyl group. Interestingly, the reaction of substrate **4** did give the six‐membered product **5** in 25 % yield and 84 % *ee*, but the cyclopent‐2‐enone **6 a** resulting from cyclization of the intermediate alkenylnickel species onto one of the ester groups was also obtained in 14 % yield^[12]^ and 85 % *ee* (Scheme 3). The enantioselective formation of cyclopent‐2‐enones in this manner was described by our group previously,^[3c]^ and in this case, it appears that despite the lower electrophilicity of the methyl esters in **4** compared with the phenyl ketone, the kinetic preference to form a five‐membered ring over a six‐membered ring makes the formation of **6 a** competitive with **5**. An attempt to prepare a five‐membered cycloalkanol by the reaction of PhB(OH)_2_ with substrate **7** was unsuccessful, and gave a complex mixture of unidentified products (Scheme 4). However, it should be noted that the formation of five‐membered rings by *anti*‐carbometallative cyclizations onto cyclic 1,3‐diketones was successful in our previous work (see Scheme 1 A).^[3a]^

**Scheme 2:**
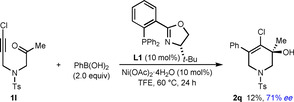
Reaction of chloroalkyne **1l**.

**Scheme 3:**
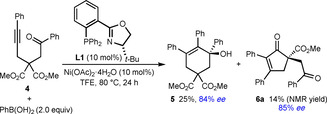
Formation of carbocyclic products **5** and **6 a**.

**Scheme 4:**
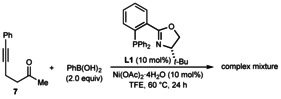
Attempted reaction of substrate **7**.

Attempts to form seven‐membered ring products are shown in Schemes 5 and 6. Substrate **8** reacted with PhB(OH)_2_ to give cyclopent‐2‐enone **6 b** in 55 % yield and 19 % *ee* ^[13]^ but no product resulting from cyclization onto the ketone was observed (Scheme 5). In addition, the reaction of substrate **9** with PhB(OH)_2_ led to the trisubstituted alkene (*Z*)‐**10** resulting from alkyne hydroarylation as the only isolable product in 34 % yield (Scheme 6). The identity of the remainder of the material in this reaction was not clear; although this did not appear to contain an appreciable quantity of the corresponding *E*‐isomer of **10**, we cannot rule out its presence resulting from *E*/*Z* isomerization of the alkenylnickel intermediate.

**Scheme 5:**
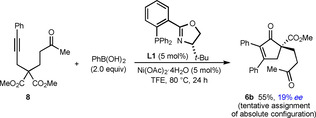
Attempted formation of a seven‐membered carbocycle.

**Scheme 6:**
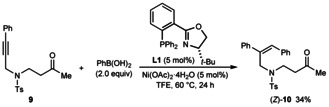
Attempted formation of a seven‐membered azacycle.

In summary, we have described enantioselective nickel‐catalyzed *anti*‐arylmetallative cyclizations of (hetero)arylboronic acids with substrates containing an alkyne tethered to an acyclic ketone, which proceed to give chiral tertiary alcohols with high enantioselectivities in most cases (often ≥99 % *ee*). Compared with a previous study,^[3a]^ this work demonstrates a substantial increase in scope of ketones that can be used as electrophiles. The products are 4,5‐diaryl‐1,2,3,6‐tetrahydropyridines, a ring system that is seen in certain indolizidine alkaloids. The formation of carbocyclic products is also possible.^[14]^

## Conflict of interest

The authors declare no conflict of interest.

## Supporting information

As a service to our authors and readers, this journal provides supporting information supplied by the authors. Such materials are peer reviewed and may be re‐organized for online delivery, but are not copy‐edited or typeset. Technical support issues arising from supporting information (other than missing files) should be addressed to the authors.

Supplementary
