# Supplementary material for: Enantioselective Nickel‐Catalyzed anti‐Arylmetallative Cyclizations onto Acyclic Ketones
Source: Chemistry. 2021 Mar 5;27(19):5897–900. doi: 10.1002/chem.202100143 (PMC8048927; doi:10.1002/chem.202100143)
Supplement: Supplementary file 1 — Supplementary [file CHEM-27-5897-s001.pdf]

Chemistry–A European Journal

Supporting Information

Enantioselective Nickel-Catalyzed *anti*-Arylmetallative Cyclizations onto Acyclic Ketones

Harley Green,[a, b] Stephen P. Argent,[b] and Hon Wai Lam*[a, b]

Author Contributions

H.G. Formal analysis: Lead; Investigation: Lead; Methodology: Lead; Validation: Lead; Writing – review & editing: Supporting
S.A. Data curation: Supporting; Formal analysis: Supporting; Validation: Supporting
H.L. Conceptualization: Equal; Formal analysis: Supporting; Funding acquisition: Lead; Investigation: Supporting; Supervision: Lead; Writing – original draft: Lead; Writing – review & editing: Lead.

Supporting Information

Contents

1. General Information.......... 2
2. Preparation of Substrates for Arylative Cyclizations onto Ketones.......... 3
2.1 Preparation of Key Intermediates.......... 3
2.2 Preparation of Final Arylative Cyclization Precursors.......... 3
3. Enantioselective Nickel-Catalyzed Arylative Cyclizations onto Ketones.......... 16
4. NMR Spectra of New Compounds.......... 30
5. HPLC Traces.......... 68
6. References.......... 87

1. General Information

All air-sensitive reactions were carried out under an inert atmosphere using oven-dried apparatus. 2,2,2-Trifluoroethanol (TFE) was purchased from Fluorochem and degassed before use using a stream of argon gas (20 min). All commercially available reagents were used as received unless otherwise stated. Petroleum ether refers to Sigma-Aldrich product 24587 (petroleum ether boiling point 40−60 °C). Thin layer chromatography (TLC) was performed on Merck DF-Alufoilien 60F254 0.2 mm precoated plates. Compounds were visualized by exposure to UV light or by dipping the plates into solutions of potassium permanganate or vanillin followed by gentle heating. Flash column chromatography was carried out using silica gel (Fisher Scientific 60 Å particle size 35-70 micron or Fluorochem 60 Å particle size 40-63 micron). Melting points were recorded on a Gallenkamp melting point apparatus and are uncorrected. The solvent of recrystallization is reported in parentheses. Infrared (IR) spectra were recorded on Bruker platinum alpha FTIR spectrometer on the neat compound using the attenuated total reflectance technique. NMR spectra were acquired on Bruker Ascend 400 or Ascend 500 spectrometers. ^{1}H and ^{13}C NMR spectra were referenced to external tetramethylsilane via the residual protonated solvent (^{1}H) or the solvent itself (^{13}C). ^{19}F NMR spectra were referenced through the solvent lock (^{2}H) signal according to the IUPAC-recommended secondary referencing method following Bruker protocols. All chemical shifts are reported in parts per million (ppm). For $CDCl_3$, the shifts are referenced to 7.26 ppm for ^{1}H NMR spectroscopy and 77.16 ppm for ^{13}C NMR spectroscopy. Coupling constants (*J*) are quoted to the nearest 0.1 Hz. Assignments were made using the DEPT sequence with secondary pulses at 90° and 135°. High-resolution mass spectra were recorded using electrospray ionization (ESI) techniques. X-ray diffraction data were collected at 120 K on an Agilent SuperNova diffractometer using CuKα radiation. Solvents (THF, CH_2Cl_2, $CHCl_3$, toluene, Et_3N and DMF) were freshly degassed (20 min with a stream of argon). Ligand **L1** was prepared according to a literature procedure.[1] 2-[2-(Diphenylphosphino)ethyl]pyridine or racemic Ph-PHOX[2] were used as achiral ligands to prepare authentic racemic products for obtaining chiral HPLC assays.

2. Preparation of Substrates for Arylative Cyclizations onto Ketones

2.1 Preparation of Key Intermediates

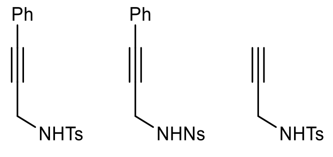

Alkynes **S1**,[3] **S2**[4] and **S3**[5] were prepared according to previously reported procedures.

2.2 Preparation of Final Arylative Cyclization Precursors

General Procedure A: Sonogashira Coupling

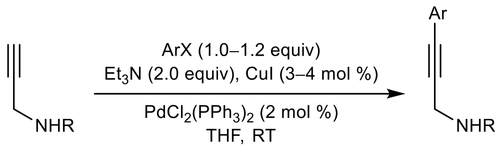

To a suspension of aryl halide (1.0–1.2 equiv), Et_3N (2.0 equiv), $Pd(PPh_3)_2Cl_2$ (2 mol%) and CuI (3–4 mol%) in anhydrous THF under an argon atmosphere at room temperature was added the alkyne (1.0 equiv) and the resulting mixture was stirred at room temperature for the indicated time. The mixture was filtered through celite (using Et_2O as the eluent) and the filtrate was concentrated *in vacuo*. The residue was purified by column chromatography to give the title compound.

General Procedure B: Sulfonamide Alkylation

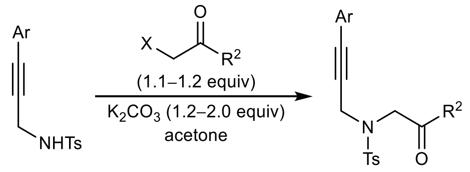

To a suspension of the alkyne (1.0 equiv) and K_2CO_3 (1.2–2.0 equiv) in acetone at room temperature was added the α-haloketone (1.1–1.2 equiv) and the mixture was stirred at the indicated temperature for the indicated time. The mixture was filtered through celite (EtOAc) and the filtrate was concentrated *in vacuo*. The residue was purified by column chromatography to give the title compound.

4-Methyl-*N*-(2-oxo-2-phenylethyl)-*N*-(3-phenylprop-2-yn-1-yl)benzenesulfonamide (1a)[6]

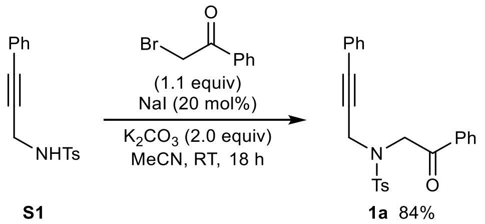

To a solution of alkyne **S1**[3] (1.00 g, 3.50 mmol) and 2-bromo-1-phenylethan-1-one (766 mg, 3.85 mmol) in MeCN (11 mL) at room temperature was added K_2CO_3 (967 mg, 7.00 mmol) and NaI (105 mg, 0.70 mmol), and the resulting mixture was stirred at room temperature for 18 h. H_2O (10 mL) was added and the MeCN was removed *in vacuo*. The resulting mixture was extracted with EtOAc (3 × 10 mL) and the combined organic layers were washed with brine (10 mL), dried ($MgSO_4$), filtered, and concentrated *in vacuo*. The residue was purified by column chromatography (15% EtOAc/pet. ether) to give the ketone **1a** (1.18 g, 84%) as an orange oil. The analytical data were consistent with those reported previously.[6]

***N*-[2-(4-Chlorophenyl)-2-oxoethyl]-4-methyl-*N*-(3-phenylprop-2-yn-1-yl)benzenesulfonamide (1b)**

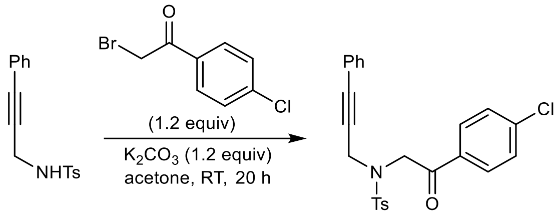

To a suspension of alkyne **S1**[3] (250 mg, 0.88 mmol) and K_2CO_3 (145 mg, 1.05 mmol) in acetone (6 mL) under an argon atmosphere at room temperature was added 2-bromo-4′-chloroacetophenone (245 mg, 1.05 mmol) and the resulting mixture was stirred for 20 h. H_2O (10 mL) and brine (5 mL) were added and the mixture extracted with EtOAc (3 × 10 mL). The combined organic layers were dried ($MgSO_4$), filtered, and concentrated *in vacuo*. The residue was purified by column chromatography (20% EtOAc/pet. ether) to give the ketone **1b** (265 mg, 69%) as a white solid. R_f = 0.44 (20% EtOAc/pet. ether); m.p. 123–125 °C ($CHCl_3$); IR 2916, 1699 (C=O), 1590, 1490, 1402, 1349, 1225, 1161, 1093, 913 cm^{-1}; ^1H NMR (400 MHz, $CDCl_3$) δ 8.32 (2H, d, J = 8.7 Hz, Ar**H**), 8.17 (2H, d, J = 8.7 Hz, Ar**H**), 7.80 (2H, d, J = 8.0 Hz, Ar**H**), 7.34 (2H, d, J = 8.0 Hz, Ar**H**), 7.30-7.22 (3H, m, Ar**H**), 7.10 (2H, d, J = 7.2 Hz, Ar**H**), 4.76 (2H, s, C**H**$_2$C=O), 4.47 (2H, s, C**H**$_2$C≡C), 2.41 (3H, s, C**H**$_3$); ^{13}C NMR (101 MHz, $CDCl_3$) δ 192.6 (C), 144.1 (C), 140.5 (C), 135.9 (C), 133.3 (C), 131.7 (2 × CH), 129.9 (2 × CH), 129.8 (2 × CH), 129.3 (2 × CH), 128.8 (CH), 128.3 (2 × CH), 127.9 (2 × CH), 122.0

(C), 86.5 (C), 81.5 (C), 52.2 (CH_2), 38.5 (CH_2), 21.6 (CH_3); HRMS (ESI) Exact mass calculated for $[C_{24}H_{20}ClNO_3SNa]^+$ $[M+Na]^+$: 460.0745, found: 460.0744.

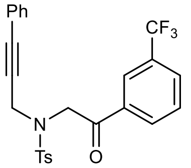

4-Methyl-*N*-{2-oxo-2-[3-(trifluoromethyl)phenyl]ethyl}-*N*-(3-phenylprop-2-yn-1-yl)benzenesulfonamide (1c). Prepared according to General Procedure B, using alkyne **S1**[3] (500 mg, 1.75 mmol), 2-bromo-1-[3-(trifluoromethyl)phenyl]ethan-1-one (0.35 mL, 2.10 mmol) and K_2CO_3 (484 mg, 3.50 mmol) in acetone (6 mL) at room temperature for 24 h. Purification by column chromatography (0 to 20% EtOAc/pet. ether) gave the ketone **1c** (307 mg, 37%) as an orange solid. R_f = 0.26 (10% EtOAc/pet. ether); m.p. 118–120 °C ($CHCl_3$); IR 2924, 1706 (C=O), 1612, 1598, 1490, 1327, 1263, 1158, 1126, 1092 cm^{-1}; ^{1}H NMR (400 MHz, $CDCl_3$) δ 8.25-8.17 (2H, m, Ar**H**), 7.88-7.77 (3H, m, Ar**H**), 7.63 (1H, t, *J* = 7.8 Hz, Ar**H**), 7.36-7.31 (2H, m, Ar**H**), 7.30-7.20 (3H, m, Ar**H**), 7.15-7.07 (2H, m, Ar**H**), 4.79 (2H, s, C**H**$_2$C=O), 4.46 (2H, s, C**H**$_2$C≡), 2.40 (3H, s, C**H**$_3$); ^{13}C NMR (101 MHz, $CDCl_3$) δ 192.6 (C), 144.2 (C), 135.8 (C), 135.5 (C), 131.7 (2 × CH), 131.6 (q, *J* = 33.0 Hz, C), 131.6, (CH), 130.3 (q, *J* = 3.5 Hz, CH), 129.9 (2 × CH), 129.7 (CH), 128.8 (CH), 128.3 (2 × CH), 127.9 (2 × CH), 125.2 (q, *J* = 3.8 Hz, CH), 123.7 (q, *J* = 272.7 Hz, C), 121.9 (C), 86.7 (C), 81.4 (C), 52.4 (CH_2), 38.6 (CH_2), 21.6 (CH_3); ^{19}F NMR (376 MHz, $CDCl_3$) δ −62.8 (s, 3 × F); HRMS (ESI) Exact mass calculated for $[C_{25}H_{21}F_3NO_3S]^+$ $[M+H]^+$: 472.1189, found: 472.1188.

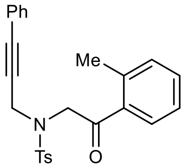

4-Methyl-*N*-[2-oxo-2-(*o*-tolyl)ethyl]-*N*-(3-phenylprop-2-yn-1-yl)benzenesulfonamide (1d). Prepared according to General Procedure B, using alkyne **S1**[3] (500 mg, 1.75 mmol), 2-bromo-1-(*o*-tolyl)ethan-1-one (305 µL, 2.10 mmol) and K_2CO_3 (484 mg, 3.50 mmol) in acetone (6 mL) at room temperature for 24 h. Purification by column chromatography (15 to 20% EtOAc/pet. ether) gave the ketone **1d** (400 mg, 55%) as an orange solid. R_f = 0.17 (10% EtOAc/pet. ether); m.p. 95–97 °C ($CHCl_3$); IR 2925, 1695 (C=O), 1598, 1570, 1489, 1347, 1223, 1157, 1091, 1068 cm^{-1}; ^{1}H NMR (400 MHz, $CDCl_3$) δ 7.84-7.77 (2H, m, Ar**H**), 7.67 (1H, dd, *J* = 8.0, 1.4 Hz, Ar**H**), 7.40 (1H, app td, *J* = 7.5, 1.4 Hz, Ar**H**), 7.33-7.20 (7H, m, Ar**H**), 7.16-7.09 (2H, m, Ar**H**), 4.69 (2H, s, C**H**$_2$C=O), 4.52 (2H, s, C**H**$_2$C≡), 2.48 (3H, C**H**$_3$), 2.38 (3H, s, C**H**$_3$); ^{13}C NMR (101 MHz, $CDCl_3$) δ 197.2 (C), 143.9 (C), 138.7 (C), 136.1 (C), 135.5 (C), 132.2 (CH), 132.1 (CH), 131.7 (2 × CH), 129.8 (2 × CH), 128.7 (CH), 128.33 (CH), 128.27 (2 × CH), 127.8 (2 × CH), 125.9 (CH), 122.0 (C), 86.3 (C), 81.7 (C), 53.8 (CH_2), 38.4 (CH_2), 21.6 (CH_3), 21.1 (CH_3); HRMS (ESI) Exact mass calculated for $[C_{25}H_{24}NO_3S]^+$ $[M+H]^+$: 418.1471, found: 418.1471.

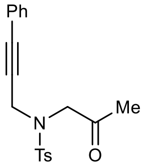

4-Methyl-*N*-(2-oxopropyl)-*N*-(3-phenylprop-2-yn-1-yl)benzenesulfonamide (1e). Prepared according to General Procedure B, using alkyne **S1**[3] (1.79 g, 6.28 mmol), chloroacetone (0.55 mL, 6.91 mmol) and K_2CO_3 (1.04 g, 7.53 mmol) in acetone (18 mL) at room temperature for 44 h. Purification by column chromatography (50% EtOAc/pet. ether) gave the ketone **1e** (1.79 g, 84%) as an orange solid. R_f = 0.22 (20% EtOAc/pet. ether); m.p. 94–96 °C (Et_2O); IR 3061, 2923, 1733 (C=O), 1598, 1491, 1349, 1161, 1099, 815, 758 cm^{-1}; ^{1}H NMR (400 MHz, $CDCl_3$) δ 7.81-7.71 (2H, m, Ar**H**), 7.35-7.21 (5H, m, Ar**H**), 7.17-7.09 (2H, m, Ar**H**), 4.39 (2H, s, C**H**$_2$C=O), 4.05 (2H, s, C**H**$_2$C≡C), 2.37 (3H, s, ArC**H**$_3$), 2.27 (3H, s, C**H**$_3$C=O); ^{13}C NMR (101 MHz, $CDCl_3$) δ 203.7 (C), 144.1 (C), 135.6 (C), 131.7 (2 × CH), 129.9 (2 × CH), 128.8 (CH), 128.4 (2 × CH), 127.8 (2 × CH), 122.0 (C), 86.4 (C), 81.4 (C), 55.9 (CH_2), 39.1 (CH_2), 27.3 (CH_3), 21.6 (CH_3); HRMS (ESI) Exact mass calculated for $[C_{19}H_{19}NO_3SNa]^+$ $[M+Na]^+$: 364.0978, found: 364.0976.

4-Nitro-*N*-(2-oxopropyl)-*N*-(3-phenylprop-2-yn-1-yl)benzenesulfonamide (1f)

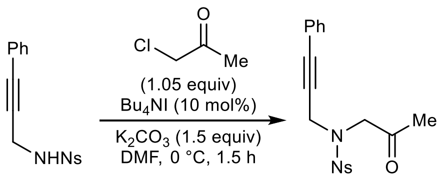

S2 **1f** 74%

To a solution of alkyne **S21**[4] (316 mg, 1.00 mmol), chloroacetone (90 μL, 1.05 mmol) and Bu_4NI (37.0 mg, 0.10 mmol) in anhydrous DMF under an argon atmosphere at 0 °C was added K_2CO_3 (207 mg, 1.50 mmol) and the resulting mixture was stirred for 1.5 h. H_2O (5 mL) was added and the mixture extracted with EtOAc (3 × 5 mL). The combined organic layers were washed with brine (5 mL), dried ($MgSO_4$), filtered, and concentrated *in vacuo*. The residue was purified by column chromatography (50% Et_2O/pet. ether) to give the ketone **1f** (294 mg, 74%) as an orange solid. R_f = 0.58 (10% EtOAc/$CHCl_3$); m.p. 146–148 °C (Et_2O/CH_2Cl_2); IR 3102 (NH), 1735 (C=O), 1607, 1530, 1351, 1311, 1165, 1105, 1013, 928 cm^{-1}; ^{1}H NMR (400 MHz, $CDCl_3$) δ 8.34-8.29 (2H, m, Ar**H**), 8.08-8.03 (2H, m, Ar**H**), 7.35-7.29 (1H, m, Ar**H**), 7.29-7.22 (2H, m, Ar**H**), 7.17-7.11 (2H, m, Ar**H**), 4.45 (2H, s, C**H**$_2$C=O), 4.21 (2H, s, C**H**$_2$C≡), 2.23 (3H, s, C**H**$_3$); ^{13}C NMR (101 MHz, $CDCl_3$) δ 202.1 (C), 150.3 (C), 144.9 (C), 131.6 (2 × CH), 129.3 (CH), 129.0 (2 × CH), 128.6 (2 × CH), 124.4 (2 × CH), 121.5 (C), 87.0 (C), 80.8 (C), 55.5 (CH_2), 39.0 (CH_2), 27.2 (CH_3); HRMS (ESI) Exact mass calculated for $[C_{18}H_{16}N_2O_5SNa]^+$ $[M+Na]^+$: 395.0672, found: 395.0668.

4-Methyl-*N*-(3-methyl-2-oxobutyl)-*N*-(3-phenylprop-2-yn-1-yl)benzenesulfonamide (1g)

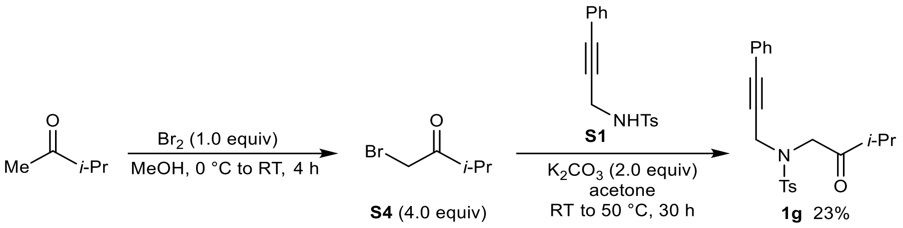

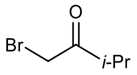

1-Bromo-3-methylbutan-2-one (S4). To a solution of 3-methylbutan-2-one (3.00 g, 34.8 mmol) in anhydrous MeOH (20 mL) at 0 °C was added bromine (1.79 mL, 34.8 mmol) and the resulting solution was stirred at 0 °C for 30 min. The ice bath was then removed and the mixture was stirred for a further 3.5 h at room temperature. H_2O (20 mL) was added and the mixture was stirred for 30 min. Further H_2O (30 mL) was added and the mixture extracted with Et_2O (3 × 30 mL). The combined organic layers were washed with saturated aqueous $NaHCO_3$ (30 mL), H_2O (30 mL), brine (30 mL), dried (Na_2SO_4), filtered, and concentrated *in vacuo*. The crude product was used without further purification.

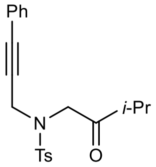

4-Methyl-*N*-(3-methyl-2-oxobutyl)-*N*-(3-phenylprop-2-yn-1-yl)benzenesulfonamide (1g). Prepared according to a modification of General Procedure B, using alkyne **S1**[3] (750 mg, 2.62 mmol), 1-bromo-3-methylbutan-2-one (**S4**, 864 mg, 5.24 mmol) and K_2CO_3 (730 mg, 5.24 mmol) in acetone (8 mL) at room temperature for 4 h and then at 50 °C for 20 h. Further 1-bromo-3-methylbutan-2-one (864 mg, 5.24 mmol) was added and the mixture was stirred for at 50 °C for 6 h. Purification by column chromatography (10% EtOAc/pet. ether) followed by recrystallisation (EtOAc/pet. ether) gave the ketone **1g** (222 mg, 23%) as a white solid. R_f = 0.48 (20% EtOAc/pet. ether); m.p. 74–76 °C ($CHCl_3$); IR 2960, 2925, 1727 (C=O), 1598, 1490, 1443, 1348, 1161, 1090, 1042 cm^{-1}; ^1H NMR (400 MHz, $CDCl_3$) δ 7.79-7.72 (2H, m, Ar**H**), 7.33-7.27 (3H, m, Ar**H**), 7.26-7.22 (2H, m, Ar**H**), 7.16-7.09 (2H, m, Ar**H**), 4.41 (2H, s, C**H**$_2$C≡), 4.20 (2H, s, C**H**$_2$C=O), 2.80 (1H, sept, *J* = 6.9 Hz, C**H**(CH_3)$_2$), 2.38 (3H, s, ArC**H**$_3$), 1.12 (6H, d, *J* = 6.9 Hz, CH(C**H**$_3$)$_2$); ^{13}C NMR (101 MHz, $CDCl_3$) δ 208.7 (C), 144.0 (C), 135.8 (C), 131.7 (2 × CH), 129.8 (2 × CH), 128.8 (CH), 128.4 (2 × CH), 127.9 (2 × CH), 122.1 (C), 86.3 (C), 81.6 (C), 53.1 (CH_2), 38.5 (CH_2), 38.2 (CH), 21.6 (CH_3), 18.3 (2 × CH_3); HRMS (ESI) Exact mass calculated for $[C_{21}H_{24}NO_3S]^+$ $[M+H]^+$: 370.1471, found: 370.1478.

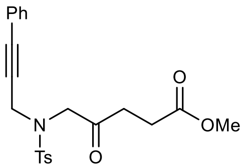

Methyl 5-{[4-methyl-*N*-(3-phenylprop-2-yn-1-yl)phenyl]sulfonamide}-4-oxopentanoate (1h). Prepared according to General Procedure B, using alkyne **S1**[3] (750 mg, 2.62 mmol), methyl 5-bromo-4-oxopentanoate (1.10 g, 5.26 mmol) and K_2CO_3 (730 mg, 5.26 mmol) in acetone (8 mL) at room temperature for 24 h. Purification by column chromatography (20 to 30% EtOAc/pet. ether) gave the ketone **1h** (693 mg, 64%) as an off-white solid. R_f = 0.23 (20% EtOAc/pet. ether); m.p. 97–99 °C ($CHCl_3$); IR 2952, 1730 (C=O), 1598, 1491, 1438, 1411, 1347, 1210, 1160, 1092 cm^{-1}; ^{1}H NMR (500 MHz, $CDCl_3$) δ 7.78-7.73 (2H, m, Ar**H**), 7.33-7.27 (3H, m, Ar**H**), 7.26-7.22 (2H, m, Ar**H**), 7.14-7.10 (2H, m, Ar**H**), 4.40 (2H, s, C**H**$_2$C≡), 4.09 (2H, s, NC**H**$_2$C=O), 3.66 (3H, s, OC**H**$_3$), 2.89 (2H, t, *J* = 6.5 Hz, C**H**$_2$CH$_2$), 2.64 (2H, t, *J* = 6.5 Hz, CH$_2$C**H**$_2$), 2.36 (3H, s, ArC**H**$_3$); ^{13}C NMR (126 MHz, $CDCl_3$) δ 204.5 (C), 173.0 (C), 144.1 (C), 135.4 (C), 131.7 (2 × CH), 129.8 (2 × CH), 128.8 (CH), 128.3 (2 × CH), 127.9 (2 × CH), 122.0 (C), 86.4 (C), 81.3 (C), 55.5 (CH_2), 52.0 (CH_3), 39.2 (CH_2), 34.3 (CH_2), 27.8 (CH_2), 21.6 (CH_3); HRMS (ESI) Exact mass calculated for $[C_{22}H_{23}NO_5SNa]^+$ $[M+Na]^+$: 436.1189, found: 436.1193.

Methyl 4-(3-{[4-methyl-*N*-(2-oxopropyl)phenyl]sulfonamide}prop-1-yn-1-yl)benzoate (1i)

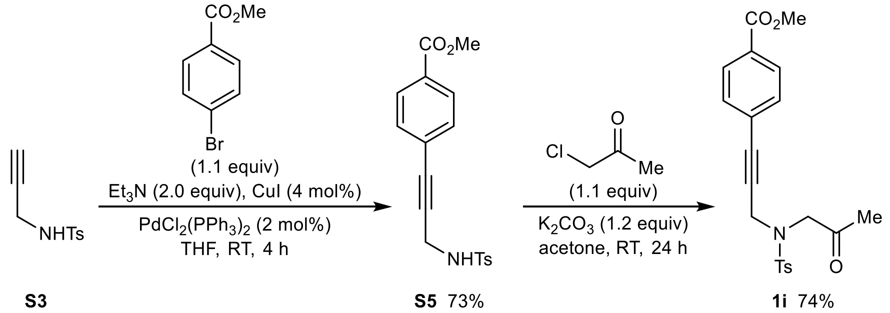

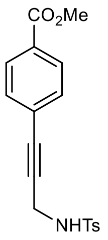

Methyl 4-{3-[(4-methylphenyl)sulfonamide]prop-1-yn-1-yl}benzoate (S5).[7] Prepared according to General Procedure A, using alkyne **S3**[5] (1.50 g, 7.17 mmol), methyl 4-iodobenzoate (2.07 g, 7.88 mmol), Et_3N (2.0 mL, 14.3 mmol), $Pd(PPh_3)_2Cl_2$ (101 mg, 0.14 mmol) and CuI (55.0 mg, 0.29 mmol) in anhydrous THF (7 mL) for 4 h. Purification by column chromatography (25 to 50% EtOAc/pet. ether) gave the alkyne **S5** (1.80 g, 73%) as an orange solid. The analytical data were consistent with those reported previously.[7]

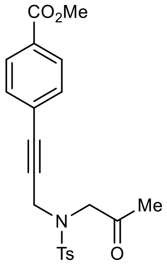

Methyl 4-(3-{[4-methyl-*N*-(2-oxopropyl)phenyl]sulfonamide}prop-1-yn-1-yl)benzoate (1i). Prepared according to General Procedure B, using alkyne **S5** (750 mg, 2.18 mmol), chloroacetone (0.19 mL, 2.40 mmol) and K_2CO_3 (362 mg, 2.62 mmol) in acetone (6 mL) at room temperature for 24 h. Purification by column chromatography (30% EtOAc/pet. ether) gave the ketone **1i** (645 mg, 74%) as an off-white solid. R_f = 0.45 (30% EtOAc/pet. ether); m.p. 107–109 °C ($CHCl_3$); IR 2953, 1720 (C=O), 1606, 1436, 1349, 1308, 1277, 1161, 1106, 1019 cm^{-1}; ^{1}H NMR (400 MHz, $CDCl_3$) δ 7.96-7.88 (2H, m, Ar**H**), 7.79-7.72 (2H, m, Ar**H**), 7.29 (2H, d, *J* = 7.8 Hz, Ar**H**), 7.21-7.14 (2H, m, Ar**H**), 4.41 (2H, s, C**H**$_2$C≡), 4.06 (2H, s, C**H**$_2$C=O), 3.91 (3H, s, OC**H**$_3$), 2.37 (3H, s, C**H**$_3$C=O), 2.26 (3H, s, ArC**H**$_3$); ^{13}C NMR (101 MHz, $CDCl_3$) δ 203.4 (C), 166.4 (C), 144.2 (C), 135.5 (C), 131.6 (2 × CH), 130.1 (C), 129.9 (2 × CH), 129.5 (2 × CH), 127.8 (2 × CH), 126.6 (C), 85.5 (C), 84.5 (C), 55.8 (CH_2), 52.4 (CH_3), 39.0 (CH_2), 27.3 (CH_3), 21.6 (CH_3); HRMS (ESI) Exact mass calculated for $[C_{21}H_{22}NO_5S]^+$ $[M+H]^+$: 400.1213, found: 400.1213.

N-[3-(3-Methoxyphenyl)prop-2-yn-1-yl]-4-methyl-N-(2-oxopropyl)benzenesulfonamide (1j)

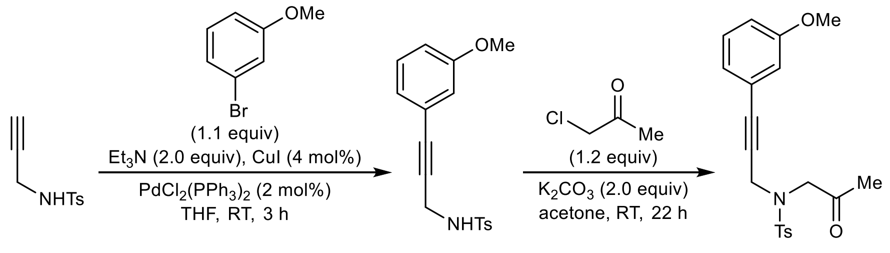

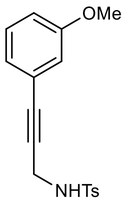

N-[3-(3-Methoxyphenyl)prop-2-yn-1-yl]-4-methylbenzenesulfonamide (S6). Prepared according to General Procedure A, using alkyne **S3**[5] (2.00 g, 9.56 mmol), 3-bromoanisole (1.4 mL, 11.5 mmol), Et_3N (2.7 mL, 19.1 mmol), $PdCl_2(PPh_3)_2$ (134 mg, 0.19 mmol) and CuI (55 mg, 0.39 mmol) in anhydrous THF (10 mL) for 3 h. Purification by column chromatography (20% EtOAc/pet. ether) gave the alkyne **S6** (2.22 g, 74%) as an orange solid. R_f = 0.19 (20% EtOAc/pet. ether); m.p. 109–110 °C ($CHCl_3$); IR 3274 (NH), 2943, 1598, 1574, 1480, 1427, 1392, 1289, 1156, 1046 cm^{-1}; ^{1}H NMR (400 MHz, $CDCl_3$) δ 7.85-7.77 (2H, m, Ar**H**), 7.32-7.24 (2H, m, Ar**H**), 7.15 (1H, app t, *J* = 8.0 Hz, Ar**H**), 6.88-6.80 (1H, m, Ar**H**), 6.73 (1H, app dt, *J* = 7.6, 1.2, Ar**H**), 6.68-6.66 (1H, m, Ar**H**), 4.71-4.64 (1H, br m, N**H**), 4.07 (2H, d, *J* = 6.1 Hz, C **H**$_2$), 3.77 (3H, s, OC**H**$_3$), 2.36 (3H, s, ArC**H**$_3$); ^{13}C NMR (101 MHz, $CDCl_3$) δ 159.3 (C), 144.0 (C), 137.0 (C), 129.9 (2 × CH), 129.4 (CH), 127.6 (2 × CH), 124.2 (CH), 123.2 (C), 116.9 (CH), 114.9 (CH), 84.8 (C), 83.2 (C), 55.4 (CH_3), 33.9 (CH_2), 21.6 (CH_3); HRMS (ESI) Exact mass calculated for $[C_{17}H_{17}NO_3SNa]^+$ $[M+Na]^+$: 338.0821, found: 338.0821.

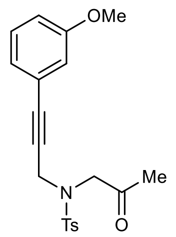

***N*-[3-(3-Methoxyphenyl)prop-2-yn-1-yl]-4-methyl-*N*-(2-oxopropyl)benzenesulfonamide (1j).** Prepared according to a modification of General Procedure B, using alkyne **S6** (750 mg, 2.38 mmol), chloroacetone (0.23 mL, 2.85 mmol) and K_2CO_3 (657 mg, 4.76 mmol) in acetone (7 mL) at room temperature for 22 h. Purification by silica plug (50% EtOAc/pet. ether) gave the ketone **1j** (869 mg, 98%) as a yellow solid. R*f* = 0.42 (30% EtOAc/pet. ether); m.p. 72–73 °C ($CHCl_3$); IR 2941, 1733 (C=O), 1597, 1575, 1481, 1422, 1348, 1290, 1204, 1160 cm^{-1}; ^{1}H NMR (400 MHz, $CDCl_3$) δ 7.77-7.73 (2H, m, Ar**H**), 7.33-7.27 (2H, m, Ar**H**), 7.16 (1H, app t, *J* = 8.1 HZ, Ar**H**), 6.87-6.82 (1H, m, Ar**H**), 6.72 (1H, app dt, *J* = 7.6, 1.2 Hz, Ar**H**), 6.67-6.64 (1H, m, Ar**H**), 4.38 (2H, s, C**H**$_2$C≡), 4.05 (2H, s, C**H**$_2$C=O), 3.77 (3H, s, OC**H**$_3$), 2.38 (3H, s, C**H**$_3$C=O), 2.26 (3H, s, ArC**H**$_3$); ^{13}C NMR (101 MHz, $CDCl_3$) δ 203.6 (C), 159.3 (C), 144.2 (C), 135.5 (C), 129.9 (2 × CH), 129.4 (CH), 127.8 (2 × CH), 124.1 (CH), 122.9 (C), 117.0 (CH), 114.9 (CH), 86.3 (C), 81.2 (C), 55.8 (CH_2), 55.4 (CH_3), 39.0 (CH_2), 27.3 (CH_3), 21.6 (CH_3); HRMS (ESI) Exact mass calculated for $[C_{20}H_{22}NO_4S]^+$ $[M+H]^+$: 372.1264, found: 372.1262.

4-Methyl-*N*-(pent-4-en-2-yn-1-yl)benzenesulfonamide (1k)

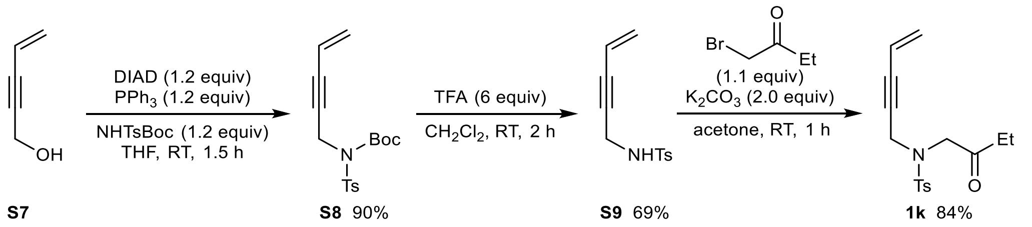

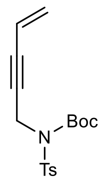

***tert*-Butyl pent-4-en-2-yn-1-yl(tosyl)carbamate (S8)**. To a solution of alcohol **S7**[8] (400 mg, 4.79 mmol), *tert*-butyl tosylcarbamate (1.56 g, 5.75 mmol) and PPh_3 (1.51 g, 5.75 mmol) in anhydrous THF (16 mL) under an argon atmosphere at 0 °C was added DIAD (1.13 mL, 5.75 mmol), the ice bath was removed and the resulting solution was stirred at room temperature for 1.5 h. The mixture was concentrated *in vacuo*. The residue was purified by column chromatography (15% Et_2O/pet. ether) to give the carbamate **S8** (1.44 g, 90%) as a white solid. R*f* = 0.27 (15% Et_2O/pet. ether); m.p. 116–118 °C (Et_2O); IR 2982, 2937, 1722 (C=O), 1595, 1350, 1257, 1147, 1087, 908, 838 cm^{-1}; ^{1}H NMR (400 MHz, $CDCl_3$) δ 7.92 (2H, d, *J* = 8.1 Hz, Ar**H**), 7.30 (2H, d, *J* = 8.1 Hz, Ar**H**), 5.80 (1H, ddt, *J* = 17.7, 11.0, 1.9 Hz, **H**C=CH_2), 5.63 (1H, dd, *J* = 17.7, 2.2 Hz, =C**H**$_a$H$_b$), 5.51 (1H, dd, *J* = 11.0, 2.2 Hz, =CH$_a$**H**$_b$), 4.74 (2H, d, *J* = 1.9 Hz, NC**H**$_2$), 2.44 (3H, s, ArC**H**$_3$), 1.35 (9H, s, (C**H**$_3$)$_3$); ^{13}C NMR (101 MHz, $CDCl_3$) δ 150.4 (C), 144.5 (C), 136.9 (C), 129.3 (2 × CH), 128.4 (2 × CH), 127.8 (CH_2), 116.8 (CH), 85.2 (C), 85.0 (C), 82.7 (C), 36.6

(CH_2), 28.0 (3 × CH_3), 21.8 (CH_3); HRMS (ESI) Exact mass calculated for $[C_{17}H_{21}NO_4SNa]^+$ $[M+Na]^+$: 358.1083, found: 358.1074.

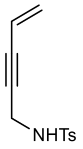

4-Methyl-*N*-(pent-4-en-2-yn-1-yl)benzenesulfonamide (S9).[9] To a solution of carbamate **S8** (1.00 g, 2.98 mmol) in CH_2Cl_2 (10 mL) under an argon atmosphere at 0 °C was added TFA (1.40 mL, 17.9 mmol). The ice bath was removed and the resulting mixture was stirred at room temperature for 2 h. The mixture was concentrated *in vacuo*, and the residue was dissolved in EtOAc (15 mL), washed with H_2O (15 mL) and brine (15 mL). The combined organic layers were dried ($MgSO_4$), filtered, and concentrated *in vacuo* to give the sulfonamide **S9** (485 mg, 69%) as an off-white solid. The analytical data were consistent with those reported previously.[9]

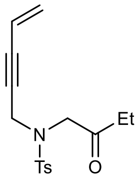

4-Methyl-*N*-(2-oxobutyl)-*N*-(pent-4-en-2-yn-1-yl)benzenesulfonamide (1k). Prepared according to General Procedure B, using alkyne **S9** (175 mg, 0.74 mmol), 1-bromobutan-2-one (84 µL, 0.82 mmol) and K_2CO_3 (200 mg, 1.49 mmol) in acetone (3 mL) at room temperature for 1 h. Purification by column chromatography (20% EtOAc/pet. ether) gave the ketone **23k** (189 mg, 84%) as a colorless oil. R_f = 0.27 (20% EtOAc/pet. ether); IR 2978, 1728 (C=O), 1598, 1494, 1411, 1344, 1557, 1103, 1039, 974 cm^{-1}; ^1H NMR (500 MHz, $CDCl_3$) δ 7.71-7.65 (2H, m, Ar**H**), 7.30-7.25 (2H, m, Ar**H**), 5.51 (1H, ddt, *J* = 17.1, 11.5, 2.0 Hz, C**H**=CH_2), 5.40-5.32 (2H, m, =C**H**$_2$), 4.24 (2H, d, *J* = 2.0 Hz, C**H**$_2$C≡), 3.97 (2H, s, C**H**$_2$C=O), 2.53 (2H, q, *J* = 7.3 Hz, C**H**$_2$$CH_3$), 2.38 (3H, s, ArC**H**$_3$), 1.04 (3H, t, *J* = 7.3 Hz, CH_2C**H**$_3$); ^{13}C NMR (126 MHz, $CDCl_3$) δ 206.1 (C), 143.9 (C), 135.4 (C), 129.7 (2 × CH), 127.9 (CH_2), 127.7 (2 × CH), 116.0 (CH), 84.8 (C), 82.1 (C), 54.7 (CH_2), 38.8 (CH_2), 33.0 (CH_2), 21.5 (CH_3), 7.4 (CH_3); HRMS (ESI) Exact mass calculated for $[C_{16}H_{20}NO_3S]^+$ $[M+H]^+$: 306.1158, found: 306.1163.

N-(3-Chloroprop-2-yn-1-yl)-4-methyl-N-(2-oxopropyl)benzenesulfonamide (1l)

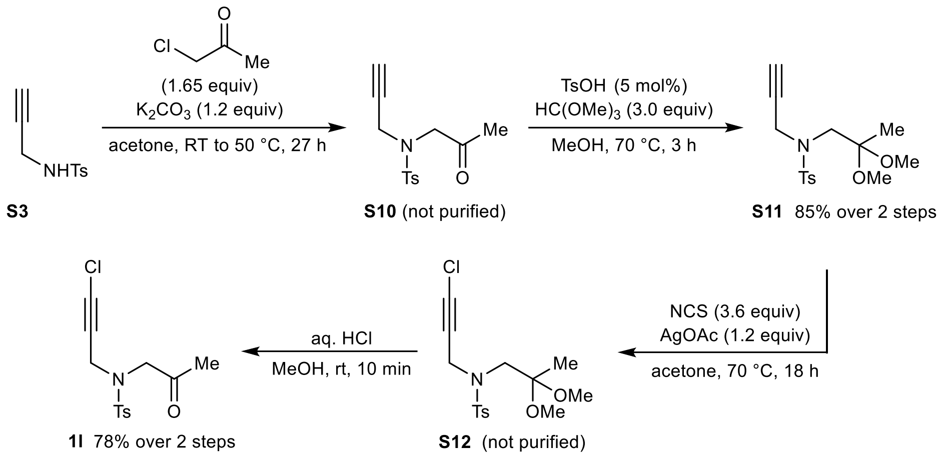

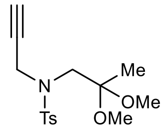

***N*-(2,2-Dimethoxypropyl)-4-methyl-*N*-(prop-2-yn-1-yl)benzenesulfonamide (S11).** To a suspension of alkyne **S3**[5] (1.00 g, 4.78 mmol) and K_2CO_3 (793 mg, 5.73 mmol) in acetone (15 mL) at room temperature was added chloroacetone (0.42 mL, 5.26 mmol) and the resulting mixture was stirred at room temperature for 24 h. Further chloroacetone (0.21 mL, 2.58 mmol) was added and the mixture was stirred at 50 °C for a further 3 h. The reaction mixture was filtered through celite (EtOAc) and the filtrate was concentrated *in vacuo* to leave the ketone **S10**, which was used directly in the next step without further purification. The flask containing the ketone **S10** was evacuated and backfilled with argon three times, and anhydrous MeOH (25 mL), followed by *p*-toluenesulfonic acid monohydrate (46 mg, 0.24 mmol) were added. The solution was heated to 70 °C and trimethyl orthoformate (1.57 mL, 14.3 mmol) was added, and the mixture was stirred at 70 °C for 3 h. The reaction was cooled to room temperature, diluted with Et_2O (70 mL), and then washed with saturated aqueous $NaHCO_3$ (20 mL), H_2O (20 mL), and brine (20 mL). The organic layer was dried ($MgSO_4$), filtered, and concentrated *in vacuo*. The residue was purified by column chromatography (15% EtOAc/pet. ether) to give the dimethyl acetal **S11** (1.26 g, 85% over 2 steps) as a colorless oil. R_f = 0.68 (30% EtOAc/pet. ether); IR 3272, 2946, 1598, 1434, 1385, 1348, 1331, 1280, 1158, 1122 cm^{-1}; ^{1}H NMR (500 MHz, $CDCl_3$) δ 7.75-7.70 (2H, m, Ar**H**), 7.28 (2H, d, *J* = 8.0 Hz, Ar**H**), 4.30 (2H, d, *J* = 2.4 Hz, C**H**$_2$C≡), 3.33 (2H, s, C**H**$_2$C(OCH$_3$)$_2$), 3.21 (6H, s, 2 × OC**H**$_3$), 2.41 (3H, s, ArC**H**$_3$), 1.93 (1H, t, *J* = 2.4 Hz, ≡C**H**), 1.43 (3H, s, CC**H**$_3$); ^{13}C NMR (126 MHz, $CDCl_3$) δ 143.7 (C), 136.3 (C), 129.5 (2 × CH), 128.0 (2 × CH), 101.9 (C), 77.3 (C), 73.9 (CH), 48.9 (CH_2), 48.7 (2 × CH_3), 37.4 (CH_2), 21.7 (CH_3), 20.2 (CH_3); HRMS (ESI) Exact mass calculated for $[C_{15}H_{21}NO_4SNa]^+$ $[M+Na]^+$: 334.1086, found: 334.1086.

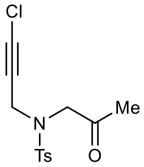

***N*-(3-Chloroprop-2-yn-1-yl)-4-methyl-*N*-(2-oxopropyl)benzenesulfonamide (1l).** To a solution of alkyne **S11** (467 mg, 1.50 mmol) in acetone (5 mL) under an argon atmosphere at room temperature was added NCS (721 mg, 5.40 mmol) and $AgNO_3$ (300 mg, 1.80 mmol), and the resulting mixture was stirred at 70 °C for 18 h. The reaction was filtered through celite (EtOAc) and concentrated *in vacuo* to leave the chloroalkyne **S12**, which was used directly in the next step without further purification. To the chloroalkyne **S12** was added MeOH (5 mL) and 10% aqueous HCl (1 mL) and the resulting suspension was stirred at room temperature for 10 min. Saturated aqueous $NaHCO_3$ (10 mL) was added and the volatiles were removed *in vacuo*. The mixture was extracted with EtOAc (20 mL) and the organic layer was washed with brine (10 mL), dried (Na_2SO_4), filtered, and concentrated *in vacuo*. The residue was purified by column chromatography (0 to 20% EtOAc/cyclohexane) to give the ketone **1l** (351 mg, 78%) as a colorless solid. R_f = 0.28 (20% EtOAc/pet. ether); m.p. 67–69 °C ($CHCl_3$); IR 2923, 2242, 1732 (C=O), 1598, 1495, 1418, 1346, 1156, 1101, 1006 cm^{-1}; ^1H NMR (400 MHz, $CDCl_3$) δ 7.70 (2H, d, *J* = 8.1 Hz, Ar**H**), 7.33 (2H, d, *J* = 8.1 Hz, Ar**H**), 4.16 (2H, s, C**H**$_2$C≡), 3.99 (2H, s, C**H**$_2$C=O), 2.43 (3H, s, ArC**H**$_3$), 2.22 (3H, s, C**H**$_3$C=O); ^{13}C NMR (101 MHz, $CDCl_3$) δ 203.1 (C), 144.3 (C), 135.4 (C), 129.9 (2 × CH), 127.8 (2 × CH), 64.5 (C), 62.2 (C), 55.6 (CH_2), 38.5 (CH_2), 27.3 (CH_3), 21.7 (CH_3); HRMS (ESI) Exact mass calculated for $[C_{13}H_{14}NO_3SClNa]^+$ $[M+Na]^+$: 322.0275, found: 322.0272.

Dimethyl 2-(2-oxo-2-phenylethyl)-2-(3-phenylprop-2-yn-1-yl)malonate (4)

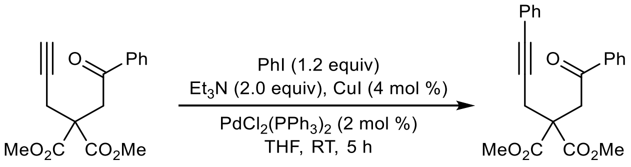

To a suspension of iodobenzene (0.40 mL, 3.60 mmol), Et_3N (0.84 mL, 6.00 mmol), $Pd(PPh_3)_2Cl_2$ (42.1 mg, 0.06 mmol) and CuI (22.9 mg, 0.12 mmol) in anhydrous THF (3 mL) under an argon atmosphere at room temperature was added the alkyne **S13**[10] (865 mg, 3.00 mmol) and the resulting mixture was stirred at room temperature for the 5 h. The mixture was filtered through celite (using Et_2O as the eluent) and the filtrate was concentrated *in vacuo*. The residue was purified by column chromatography (0 to 20% EtOAc/cyclohexane) to give the alkyne **4** (633 mg, 58%) as an orange solid. R_f = 0.43 (20% EtOAc/pet. ether); m.p. 78–80 °C ($CHCl_3$); IR 3056, 2953, 1739 (C=O), 1686 (C=O), 1596, 1581, 1448, 1251, 1205, 1183 cm^{-1}; ^1H NMR (400 MHz, $CDCl_3$) δ 8.07-7.99 (2H, m, Ar**H**), 7.62-7.54 (1H, m, Ar**H**), 7.47 (2H, t, *J* = 7.7 Hz, Ar**H**), 7.33-7.22 (5H, m, Ar**H**), 3.98 (2H, s, C**H**$_2$C=O), 3.80 (6H, s, 2 × OC**H**$_3$), 3.34 (2H, s, C**H**$_2$C≡); ^{13}C NMR (101 MHz, $CDCl_3$) δ 197.0 (C),

170.1 (2 × C), 136.5 (C), 133.7 (CH), 131.8 (2 × CH), 128.8 (2 × CH), 128.34 (2 × CH), 128.33 (2 × CH), 128.2 (CH), 123.2 (C), 84.7 (C), 84.1 (C), 55.2 (C), 53.3 (2 × CH_3), 41.4 (CH_2), 24.5 (CH_2); HRMS (ESI) Exact mass calculated for $[C_{22}H_{20}O_5Na]^+$ $[M+Na]^+$: 387.1203, found: 387.1198.

6-Phenylhex-5-yn-2-one (7)

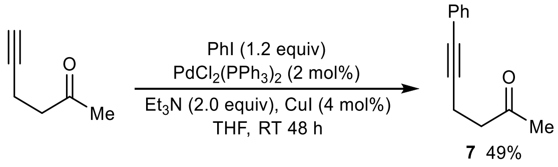

To a suspension of phenyl iodide (0.42 mL, 3.70 mmol), Et_3N (0.86 mL, 6.20 mmol), $Pd(PPh_3)_2Cl_2$ (43.5 mg, 0.06 mmol), and CuI (23.6 mg, 0.12 mmol) in anhydrous THF (3 mL) under an argon atmosphere at room temperature was added hex-5-yn-2-one[11] (298 mg, 3.10 mmol) and the resulting mixture stirred at room temperature for 48 h. The mixture was filtered through celite (using Et_2O as eluent) and concentrated *in vacuo*. The residue was purified by column chromatography (pentane to 4% Et_2O/pentane) to give **7** (263 mg, 49%) as an orange oil. The analytical data were consistent with those reported previously.[12]

Dimethyl 2-(3-oxobutyl)-2-(3-phenylprop-2-yn-1-yl)malonate (8)

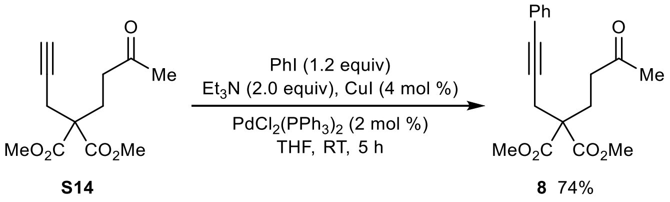

To a suspension of iodobenzene (0.40 mL, 3.60 mmol), Et_3N (0.84 mL, 6.00 mmol), $Pd(PPh_3)_2Cl_2$ (42.1 mg, 0.06 mmol) and CuI (22.9 mg, 0.12 mmol) in anhydrous THF (3 mL) under an argon atmosphere at room temperature was added the alkyne **S14**[13] (721 mg, 3.00 mmol) and the resulting mixture was stirred at room temperature for 5 h. The mixture was filtered through celite (using Et_2O as the eluent) and the filtrate was concentrated *in vacuo*. The residue was purified by column chromatography (0 to 40% EtOAc/cyclohexane) gave the alkyne **8** (707 mg, 74%) as an orange oil. R_f = 0.23 (20% EtOAc/pet. ether); IR 3001, 2964, 1731 (C=O), 1598, 1572, 1491, 1436, 1272, 1198, 1093 cm^{-1}; ^1H NMR (400 MHz, $CDCl_3$) δ 7.37-7.33 (2H, m, Ar**H**), 7.29-7.24 (3H, m, Ar**H**), 3.75 (6H, s, 2 × OC**H**$_3$), 3.03 (2H, s, C**H**$_2$C≡), 2.55 (2H, dd, *J* = 8.7, 6.6 Hz, C**H**$_2$C=O), 2.39 (2H, dd, *J* = 8.7, 6.6 Hz, C**H**$_2$CH$_2$C=O), 2.15 (3H, s, C**H**$_3$C=O); ^{13}C NMR (101 MHz, $CDCl_3$) δ 207.2 (C), 170.7 (2 × C), 131.8 (2 × CH), 128.4 (2 × CH), 128.2 (CH), 123.1 (C), 84.0 (C), 83.9 (C), 56.6 (C), 53.0 (2 × CH_3), 38.8 (CH_2), 30.0 (CH_3), 26.8 (CH_2), 24.9 (CH_2); HRMS (ESI) Exact mass calculated for $[C_{18}H_{20}O_5Na]^+$ $[M+Na]^+$: 339.1203, found: 339.1198.

4-Methyl-*N*-(3-oxobutyl)-*N*-(3-phenylprop-2-yn-1-yl)benzenesulfonamide (9)

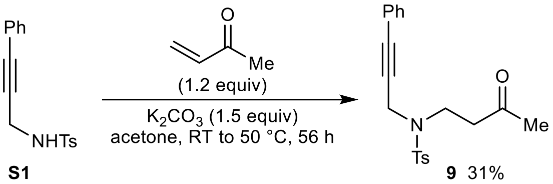

To a suspension of alkyne **S1**[3] (500 mg, 1.75 mmol) and K_2CO_3 (363 mg, 2.62 mmol) in acetone (4 mL) at room temperature was added but-3-en-2-one (0.17 mL, 2.10 mmol) and the resulting mixture stirred at room temperature for 36 h and then at 50 °C for 20 h. The mixture was filtered through celite (using EtOAc as eluent) and the filtrate was concentrated *in vacuo*. The residue was purified by column chromatography (40% Et_2O/pet. ether) to give ketone **9** (210 mg, 31%) as an orange oil. R_f = 0.21 (40% Et_2O/pet. ether); IR 2924, 1715 (C=O), 1598, 1490, 1346, 1158, 1095, 1019, 902, 815 cm^{-1}; ^{1}H NMR (400 MHz, $CDCl_3$) δ 7.80-7.73 (2H, m, Ar**H**), 7.31-7.18 (5H, m, Ar**H**), 7.10-7.03 (2H, m, Ar**H**), 4.35 (2H, s, C$\mathbf{H}_2$C≡), 3.49 (2H, t, J = 7.0 Hz, NC$\mathbf{H}_2$CH$_2$), 2.89 (2H, t, J = 7.0 Hz, C$\mathbf{H}_2$C=O), 2.34 (3H, s, C$\mathbf{H}_3$C=O), 2.19 (3H, s, ArC$\mathbf{H}_3$); ^{13}C NMR (101 MHz, $CDCl_3$) δ 206.8 (C), 143.8 (C), 135.6 (C), 131.6 (2 × CH), 129.7 (2 × CH), 128.6 (CH), 128.3 (2 × CH), 128.0 (2 × CH), 122.2 (C), 85.7 (C), 82.1 (C), 43.3 (CH_2), 42.2 (CH_2), 38.9 (CH_2), 30.3 (CH_3), 21.5 (CH_3); HRMS (ESI) Exact mass calculated for $[C_{20}H_{21}NO_3SNa]^+$ $[M+Na]^+$: 378.1133, found: 378.1133.

3. Enantioselective Nickel-Catalyzed Arylative Cyclizations onto Ketones

General Procedure C

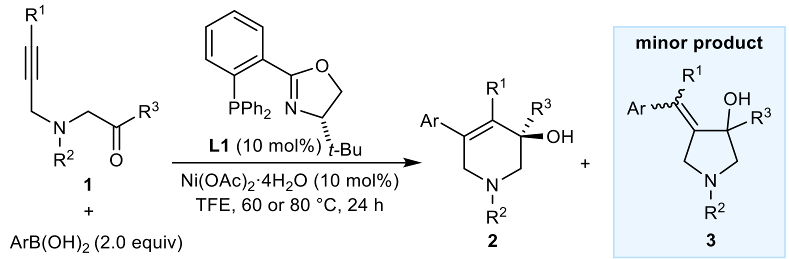

An oven dried microwave vial fitted with a magnetic stirrer bar was charged with the appropriate substrate **1** (0.30 mmol), boronic acid (0.60 mmol), $Ni(OAc)_2·4H_2O$ (7.5 mg, 0.03 mmol) and (*S*)-*t*-BuPhox (**L1**, 11.6 mg, 0.03 mmol). The vial was capped with a crimped cap seal and evacuated and backfilled with argon (3 cycles). Freshly degassed TFE (3 mL, using a stream of argon for 20 min) was added, and the mixture was stirred at room temperature for 10 min, and then at the specified temperature for 24 h. The reaction was cooled to room temperature, diluted with EtOAc (5 mL) and washed with brine (10 mL). The aqueous layer was extracted with EtOAc (3 × 5 mL). The combined organic layers were dried ($MgSO_4$), filtered, and concentrated *in vacuo*. The residue was purified by column chromatography to give the arylative cyclization product **2**.

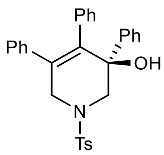

(*R*)-3,4,5-Triphenyl-1-tosyl-1,2,3,6-tetrahydropyridin-3-ol (2a). Prepared according to General Procedure C, using alkynone **1a** (121 mg, 0.30 mmol) and phenylboronic acid (73.0 mg, 0.60 mmol) at 80 °C. ^{1}H NMR analysis of the crude material showed the ratio of major:minor products was 11:1. Purification by column chromatography (CH_2Cl_2) gave **2a** (121 mg, 84%) as an off-white solid. R_f = 0.17 (CH_2Cl_2); m.p. 198–200 °C (Et_2O); $[\alpha]_D^{25}$ +16.0 (*c* 1.00, $CHCl_3$); IR 3530 (OH), 3051, 1596, 1442, 1342, 1248, 1186, 1000, 937, 815 cm^{-1}; ^{1}H NMR (400 MHz, $CDCl_3$) δ 7.68-7.60 (2H, m, Ar**H**), 7.49-7.41 (2H, m, Ar**H**), 7.37-7.29 (2H, m, Ar**H**), 7.33-7.21 (2H, m, Ar**H**), 7.25-7.15 (1H, m, Ar**H**), 7.19-7.07 (3H, m, Ar**H**), 7.04-6.95 (2H, m, Ar**H**), 6.95-6.84 (3H, m, Ar**H**), 6.84-6.76 (2H, m, Ar**H**), 4.15 (1H, d, *J* = 16.3 Hz, C$\mathbf{H}_a$$H_b$C=), 3.91 (1H, d, *J* = 16.3 Hz, $CH_a$$\mathbf{H}_b$C=), 3.60 (1H, d, *J* = 11.5 Hz, C$\mathbf{H}_a$$H_b$COH), 3.30 (1H, d, *J* = 11.5 Hz, $CH_a$$\mathbf{H}_b$COH), 2.82 (1H, s, O**H**), 2.45 (3H, s, ArC$\mathbf{H}_3$); ^{13}C NMR (101 MHz, $CDCl_3$) δ 144.2 (C), 142.3 (C), 139.0 (C), 138.9 (C), 136.6 (C), 134.7 (C), 132.9 (C), 130.7 (2 × CH), 130.0 (2 × CH), 128.7 (2 × CH), 128.2 (2 × CH), 128.1 (2 × CH), 128.0 (2 × CH), 127.5 (2 × CH), 127.44 (CH), 127.41 (CH), 126.8 (CH), 126.5 (2 × CH), 74.4 (C), 57.6 (CH_2), 50.1 (CH_2), 21.7 (CH_3); HRMS (ESI) Exact mass calculated for $[C_{30}H_{27}NO_3SNa]^+$ $[M+Na]^+$: 504.1604, found: 504.1595; Enantiomeric excess was determined by HPLC using a Chiralpak IC column (80:20 *iso*-hexane:*i*-PrOH, 0.5 mL/min, 254 nm, 25 °C); t_r (major) = 26.1 min, t_r (minor) = 28.7 min, 94% ee.

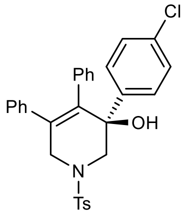

(*R*)-3-(4-Chlorophenyl)-4,5-diphenyl-1-tosyl-1,2,3,6-tetrahydropyridin-3-ol (2b). Prepared according to General Procedure C, using alkynone **1b** (131 mg, 0.30 mmol) and phenylboronic acid (73.0 mg, 0.60 mmol) at 60 °C. ^{1}H NMR analysis of the crude material showed the ratio of major:minor products was 16:1. Purification by column chromatography (5 to 15% EtOAc/pentane) gave **2b** (121 mg, 78%) as an off-white solid. R_f = 0.21 (15% EtOAc/pentane); m.p. 143–144°C ($CHCl_3$); IR 3496 (OH), 2924, 1598, 1491, 1443, 1401, 1348, 1166, 1091, 1038 cm^{-1}; $[\alpha]_D^{25}$ +4.0 (*c* 1.00, $CHCl_3$); ^{1}H NMR (500 MHz, $CDCl_3$) δ 7.66-7.61 (2H, m, Ar**H**), 7.40-7.37 (2H, m, Ar**H**), 7.33 (2H, d, *J* = 8.0 Hz, Ar**H**), 7.23-7.19 (2H, m, Ar**H**), 7.16-7.11 (3H, m, Ar**H**), 7.00-6.88 (5H, m, Ar**H**), 6.82-6.76 (2H, m, Ar**H**), 4.12 (1H, d, *J* = 16.5 Hz, C$\mathbf{H}_a$H$_b$C=), 3.94 (1H, d, *J* = 16.5 Hz, CH$_a\mathbf{H}_b$C=), 3.54 (1H, d, *J* = 11.6 Hz, C$\mathbf{H}_a$H$_b$COH), 3.27 (1H, d, *J* = 11.6 Hz, CH$_a\mathbf{H}_b$COH), 2.80 (1H, s, O**H**), 2.46 (3H, s, C$\mathbf{H}_3$); ^{13}C NMR (101 MHz, $CDCl_3$) δ 144.3 (C), 141.0 (C), 138.7 (C), 138.5 (C), 136.3 (C), 135.0 (C), 133.3 (C), 132.9 (C), 130.6 (2 × CH), 130.1 (2 × CH), 128.6 (2 × CH), 128.3 (4 × CH, 2 peaks merged into one peak), 127.9 (4 × CH, 2 peaks merged into one peak), 127.7 (2 × CH), 127.5 (CH), 127.0 (CH), 74.1 (C), 57.4 (CH_2), 50.0 (CH_2), 21.8 (CH_3); HRMS (ESI) Exact mass calculated for $[C_{30}H_{27}ClNO_3S]^+$ $[M+H]^+$: 516.1395, found: 516.1375; Enantiomeric excess was determined by HPLC using a Chiralcel OD-H column (95:5 *iso*-hexane:*i*-PrOH, mL/min, 230 nm, 25 °C); t_r (minor) = 20.4 min, t_r (major) = 23.8 min, 98% ee.

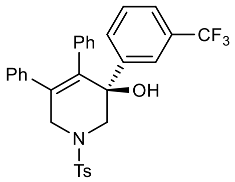

(*R*)-4,5-Diphenyl-1-tosyl-3-[3-(trifluoromethyl)phenyl]-1,2,3,6-tetrahydropyridin-3-ol (2c). Prepared according to General Procedure C, using alkynone **1c** (142 mg, 0.30 mmol) and phenylboronic acid (73.0 mg, 0.60 mmol) at 80 °C. ^{1}H NMR analysis of the crude material showed the ratio of major:minor products was 16:1. Purification by column chromatography (5 to 10% acetone/pentane) gave **2c** (125 mg, 76%) as an off-white solid. R_f = 0.36 (10% acetone/pentane); m.p. 164–166 °C ($CHCl_3$); IR 3491 (OH), 2926, 1598, 1492, 1443, 1327, 1162, 1121, 1091, 1074 cm^{-1}; $[\alpha]_D^{25}$ +44.0 (*c* 1.00, $CHCl_3$); ^{1}H NMR (400 MHz, $CDCl_3$) δ 7.72-7.62 (4H, m, Ar**H**), 7.46-7.37 (1H, m, Ar**H**), 7.41-7.29 (3H, m, Ar**H**), 7.22-7.08 (3H, m, Ar**H**), 7.05-6.94 (2H, m, Ar**H**), 6.97-6.85 (3H, m, Ar**H**), 6.85-6.75 (2H, m, Ar**H**), 4.19 (1H, d, *J* = 16.4 Hz, C$\mathbf{H}_a$H$_b$C=), 3.94 (1H, d, *J* = 16.4 Hz, CH$_a\mathbf{H}_b$C=), 3.66 (1H, d, *J* = 11.6 Hz, C$\mathbf{H}_a$H$_b$COH), 3.22 (1H, d, *J* = 11.6 Hz, CH$_a\mathbf{H}_b$COH), 3.07 (1H, s, O**H**), 2.46 (3H, s, C$\mathbf{H}_3$); ^{13}C NMR (101 MHz, $CDCl_3$) δ 144.4 (C), 143.3 (C), 138.6 (C), 138.3 (C), 136.3 (C), 135.5 (C), 132.8 (C), 130.5 (2 × CH), 130.3 (q, J_{C-F} = 31.0 Hz, C), 130.1 (2 × CH), 130.1 (CH), 128.6 (2 × CH), 128.5 (CH), 128.3 (2 × CH), 128.0 (2 × CH), 127.6 (2 × CH); 127.6 (CH), 127.0 (CH), 124.2 (q, J_{C-F} = 3.6 Hz, CH), 124.2 (q, J_{C-F} = 272.4 Hz, C), 123.4 (q, J_{C-F} = 3.7 Hz, CH), 74.2 (C), 57.5 (CH_2), 50.0 (CH_2),

21.7 (CH_3); ^{19}F NMR (376 MHz, $CDCl_3$) δ −62.6 (s, 3 × F); HRMS (ESI) Exact mass calculated for $[C_{31}H_{27}F_3NO_3S]^+$ $[M+H]^+$: 550.1645, found: 550.1658; Enantiomeric excess was determined by HPLC using a Chiralpak IC column (90:10 *iso*-hexane:*i*-PrOH, 0.5 mL/min, 254 nm, 25 °C); t_r (major) = 20.1 min, t_r (minor) = 22.3 min, 98% ee.

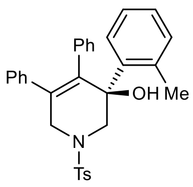

(*R*)-4,5-Diphenyl-3-(*o*-tolyl)-1-tosyl-1,2,3,6-tetrahydropyridin-3-ol (2d). Prepared according to General Procedure C, using alkynone **1d** (125 mg, 0.30 mmol) and phenylboronic acid (73.0 mg, 0.60 mmol) at 80 °C. ^{1}H NMR analysis of the crude material showed the ratio of major:minor products was 10:1. Purification by column chromatography (80 to 90% CH_2Cl_2/pet. ether) gave **2d** (107 mg, 72%) as an off-white solid. R_f = 0.24 (90% CH_2Cl_2/pet. ether); m.p. 190–192°C ($CHCl_3$); IR 3500 (OH), 3056, 2925, 1598, 1443, 1346, 1166, 1124, 1094, 1021 cm^{-1}; $[\alpha]_D^{25}$ +16.0 (*c* 1.00, $CHCl_3$); ^{1}H NMR (400 MHz, $CDCl_3$) δ 7.80 (1H, s, Ar**H**), 7.67 (2H, d, *J* = 7.9 Hz, Ar**H**), 7.34 (2H, d, *J* = 7.9 Hz, Ar**H**), 7.20-7.03 (5H, m, Ar**H**), 7.04-6.80 (8H, m, Ar**H**), 4.37 (1H, d, *J* = 16.4 Hz, C**H**$_a$H$_b$C=), 3.78 (1H, d, *J* = 12.0 Hz, C**H**$_a$H$_b$COH), 3.69 (1H, d, *J* = 16.4 Hz, CH$_a$**H**$_b$C=), 3.26 (1H, d, *J* = 12.0 Hz, CH$_a$**H**$_b$COH), 3.15 (1H, br s, O**H**), 2.46 (3H, s, C**H**$_3$), 2.33 (3H, s, C**H**$_3$); ^{13}C NMR (101 MHz, DMSO-d_6) δ 143.3 (C), 140.1 (C), 139.3 (C), 137.6 (C), 137.1 (C), 133.8 (C), 132.8 (C), 132.5 (C), 131.2 (CH), 130.0 (2 × CH), 129.5 (2 × CH), 128.1 (2 × CH), 127.6 (2 × CH), 127.5 (CH), 127.2 (2 × CH), 126.5 (2 × CH peaks merged into one peak), 126.0 (2 × CH), 125.6 (CH), 124.8 (CH), 73.7 (C), 54.7 (CH_2), 49.0 (CH_2), 20.6 (CH_3), 20.6 (CH_3); HRMS (ESI) Exact mass calculated for $[C_{31}H_{30}NO_3S]^+$ $[M+H]^+$: 496.1941, found: 496.1928; Enantiomeric excess was determined by HPLC using a Chiralpak IC column (90:10 *iso*-hexane:*i*-PrOH, 0.5 mL/min, 210 nm, 25 °C); t_r (major) = 47.3 min, t_r (minor) = 50.6 min, >99% ee.

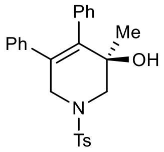

(*R*)-3-Methyl-4,5-diphenyl-1-tosyl-1,2,3,6-tetrahydropyridin-3-ol (2e). Prepared according to General Procedure C, using alkynone **1e** (102 mg, 0.30 mmol) and phenylboronic acid (73.0 mg, 0.60 mmol) at 60 °C. ^{1}H NMR analysis of the crude material showed the ratio of major:minor products was 16:1. Purification by column chromatography (0.5% MeOH/CH_2Cl_2) followed by trituration with cold Et_2O gave **2e** (88 mg, 70%) as a white solid. R_f = 0.12 (0.5% MeOH/CH_2Cl_2); m.p. 179–181 °C (Et_2O); IR 3550 (OH), 2992, 2955, 1598, 1340, 1156, 1091, 1050, 963, 835 cm^{-1}; $[\alpha]_D^{25}$ +112.0 (*c* 1.00, $CHCl_3$); ^{1}H NMR (500 MHz, $CDCl_3$) δ 7.76-7.70 (2H, d, *J* = 8.0 Hz, Ar**H**), 7.38 (2H, d, *J* = 8.0 Hz, Ar**H**), 7.15-6.98 (8H, m, Ar**H**), 6.93-6.87 (2H, m, Ar**H**), 3.99 (1H, d, *J* = 16.2 Hz, C**H**$_a$H$_b$C=), 3.70 (1H, d, *J* = 16.2 Hz, CH$_a$**H**$_b$C=), 3.56 (1H, d, *J* = 11.4 Hz, C**H**$_a$H$_b$COH), 2.98 (1H, d, *J* = 11.4 Hz, CH$_a$**H**$_b$COH), 2.50 (1H, s, O**H**), 2.47 (3H, s, ArC**H**$_3$), 1.19 (3H, s, C**H**$_3$COH); ^{13}C NMR (126 MHz, $CDCl_3$) δ 144.2 (C), 140.1 (C), 138.7 (C), 137.0 (C),

133.0 (C), 132.7 (C), 130.5 (2 × CH), 130.1 (2 × CH), 128.7 (2 × CH), 128.08 (2 × CH), 128.05 (2 × CH), 127.7 (2 × CH), 127.2 (CH), 126.9 (CH), 69.6 (C), 56.2 (CH_2), 50.2 (CH_2), 24.9 (CH_3), 21.7 (CH_3); HRMS (ESI) Exact mass calculated for $[C_{25}H_{25}NO_3SNa]^+$ $[M+Na]^+$: 442.1447, found: 442.1451; Enantiomeric excess was determined by HPLC using a Chiralpak AD-H column (90:10 *iso*-hexane:*i*-PrOH, 1.0 mL/min, 254 nm, 25 °C); t_r (minor) = 32.6 min, t_r (major) = 59.5 min, 99% ee.

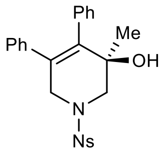

(*R*)-3-Methyl-1-[(4-nitrophenyl)sulfonyl]-4,5-diphenyl-1,2,3,6-tetrahydropyridin-3-ol (2f). Prepared according to General Procedure C, using alkynone **1f** (112 mg, 0.30 mmol) and phenylboronic acid (73.0 mg, 0.60 mmol) at 80 °C. ^{1}H NMR analysis of the crude material showed the ratio of major:minor products was >19:1. Purification by column chromatography (5% EtOAc/$CHCl_3$) gave **2f** (113 mg, 86%) as an off-white solid. R$_f$ = 0.20 (5% EtOAc/$CHCl_3$); m.p. 190–191 °C ($CHCl_3$); IR 3563 (OH), 2923, 2853, 1529, 1347, 1255, 1165, 1046, 965, 884 cm^{-1}; $[\alpha]_D^{25}$ +84.0 (*c* 1.00, $CHCl_3$); ^{1}H NMR (400 MHz, $CDCl_3$) δ 8.47-8.39 (2H, m, Ar**H**), 8.07-8.00 (2H, m Ar**H**), 7.17-7.05 (6H, m Ar**H**), 7.05-6.97 (2H, m Ar**H**), 6.94-6.87 (2H, m Ar**H**), 4.00 (1H, d, *J* = 16.2 Hz, C$\mathbf{H}_a$H$_b$C=C), 3.82 (1H, d, *J* = 16.2 Hz, CH$_a\mathbf{H}_b$C=C), 3.57 (1H, d, *J* = 11.5 Hz, C$\mathbf{H}_a$H$_b$COH), 3.15 (1H, d, *J* = 11.5 Hz, CH$_a\mathbf{H}_b$COH), 2.41 (1H, s, O**H**), 1.24 (3H, s, C$\mathbf{H}_3$); ^{13}C NMR (101 MHz, $CDCl_3$) δ 150.6 (C), 141.9 (C), 140.3 (C), 138.2 (C), 136.5 (C), 132.5 (C), 130.5 (2 × CH), 129.2 (2 × CH), 128.7 (2 × CH), 128.2 (2 × CH), 127.8 (2 × CH), 127.5 (CH), 127.1 (CH), 124.7 (2 × CH), 69.6 (C), 55.8 (CH_2), 49.9 (CH_2), 25.2 (CH_3); HRMS (ESI) Exact mass calculated for $[C_{24}H_{22}N_2O_5SNa]^+$ $[M+Na]^+$: 473.1142, found: 473.1139; Enantiomeric excess was determined by HPLC using a Chiralcel OD-H column (80:20 *iso*-hexane:*i*-PrOH, mL/min, 254 nm, 25 °C); t_r (minor) = 34.5 min, t_r (major) = 42.7 min, 99% ee.

Slow diffusion of pentane into a solution of **2f** in CH_2Cl_2 gave crystals that were suitable for X-ray crystallography:

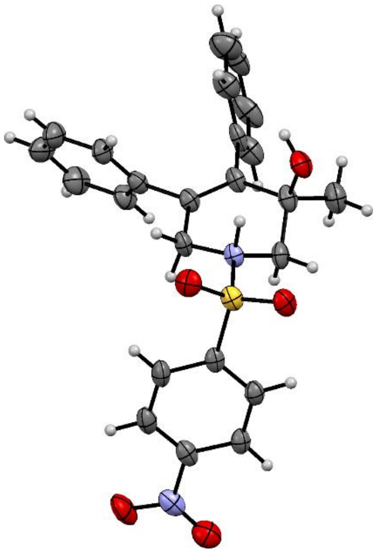

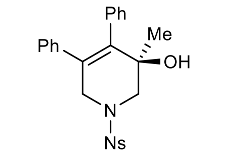

2f
CCDC 2040014
Flack parameter 0.031(12)

ORTEP with ellipsoid probabilities at 50%

Four molecules of **2f** are present in the P1 unit cell. Two different conformers are present in the cell; two molecules adopt each of the conformations (only one of the conformations is shown above, for clarity). The two conformations differ in the relative orientation of the tosyl group with respect to the hydroxyl groups.

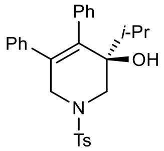

(*R*)-3-Isopropyl-4,5-diphenyl-1-tosyl-1,2,3,6-tetrahydropyridin-3-ol (2g). Prepared according to General Procedure C, using alkynone **1g** (111 mg, 0.30 mmol) and phenylboronic acid (73.0 mg, 0.60 mmol) at 80 °C. ^{1}H NMR analysis of the crude material showed the ratio of major:minor products was 12:1. Purification by column chromatography (1 to 2% EtOAc/$CHCl_3$) gave **2g** (107 mg, 80%) as a white solid. R_f = 0.15 (1% EtOAc/$CHCl_3$); m.p. 85–87 °C ($CHCl_3$); IR 3519 (OH), 3053, 2964, 1598, 1493, 1442, 1347, 1248, 1167, 1092 cm^{-1}; $[\alpha]^{25}_{D}$ +140.0 (*c* 1.00, $CHCl_3$); ^{1}H NMR (400 MHz, $CDCl_3$) δ 7.75-7.69 (2H, m, Ar**H**), 7.43-7.35 (2H, m, Ar**H**), 7.14-7.04 (8H, m, Ar**H**), 6.90-6.82 (2H, m, Ar**H**), 3.92 (1H, d, *J* = 16.0 Hz, C**H**$_a$H$_b$C=), 3.69 (1H, d, *J* = 16.0 Hz, CH$_a$**H**$_b$C=), 3.48 (1H, d, *J* = 11.7 Hz, C**H**$_a$H$_b$COH), 3.09 (1H, d, *J* = 11.7 Hz, CH$_a$**H**$_b$COH), 2.48 (3H, s, ArC**H**$_3$), 2.43 (1H, s, O**H**), 1.83 (1H, app hept, *J* = 6.9 Hz, C**H**(CH_3)$_2$), 1.04 (3H, d, *J* = 6.9 Hz, CHC**H**$_3$), 0.84 (3H, d, *J* = 6.9 Hz, CHC**H**$_3$); ^{13}C NMR (101 MHz, $CDCl_3$) δ 144.2 (C), 139.5 (C), 139.3 (C), 136.9 (C), 134.9 (C), 132.7 (C), 130.9 (2 × CH), 130.1 (2 × CH), 128.6 (2 × CH), 128.07 (2 × CH), 128.05 (2 × CH), 127.5 (2 × CH), 127.1 (CH), 126.8 (CH), 74.4 (C), 50.1 (CH_2), 49.8 (CH_2), 33.2 (CH), 21.8 (CH_3), 18.3 (CH_3), 16.1 (CH_3); HRMS (ESI) Exact mass calculated for $[C_{27}H_{29}NO_3SNa]^+$ $[M+Na]^+$: 470.1761, found: 470.1761; Enantiomeric excess was determined by HPLC using a Chiralpak IC column (80:20 *iso*-hexane:*i*-PrOH, 1.0 mL/min, 210 nm, 25 °C); t_r (major) = 9.5 min, t_r (minor) = 10.8 min, 97% ee.

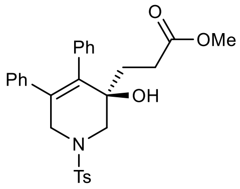

Methyl (*R*)-3-(3-hydroxy-4,5-diphenyl-1-tosyl-1,2,3,6-tetrahydropyridin-3-yl)propanoate (2h). Prepared according to General Procedure C, using alkynone **1h** (124 mg, 0.30 mmol) and phenylboronic acid (73.0 mg, 0.60 mmol) at 60 °C. ^{1}H NMR analysis of the crude material showed the ratio of major:minor products was 13:1. Purification by sequential column chromatography (15 to 20% EtOAc/pet. ether) then (3 to 5 % EtOAc/CH_2Cl_2) gave **1h** (88 mg, 56%) as a white solid. R_f = 0.40 (5% EtOAc/CH_2Cl_2); m.p. 145–146 °C ($CHCl_3$); IR 3503 (OH), 2952, 1632 (C=O), 1598, 1440, 1347, 1166, 1091, 1018, 976 cm^{-1}; $[\alpha]_D^{25}$ +112.0 (*c* 1.00, $CHCl_3$); ^{1}H NMR (400 MHz, $CDCl_3$) δ 7.74-7.64 (2H, m, Ar**H**), 7.40-7.32 (2H, m, Ar**H**), 7.16-7.05 (6H, m, Ar**H**), 7.05-6.98 (2H, m, Ar**H**), 6.94-6.86 (2H, m, Ar**H**), 3.99 (1H, d, *J* = 16.3 Hz, C$\mathbf{H}_a$H$_b$C=), 3.68 (1H, d, *J* = 16.3 Hz, CH$_a$$\mathbf{H}_b$C=), 3.61 (3H, s, OC$\mathbf{H}_3$), 3.40 (1H, d, *J* = 11.5 Hz, C$\mathbf{H}_a$H$_b$COH), 3.10 (1H, d, *J* = 11.5 Hz, CH$_a$$\mathbf{H}_b$COH), 2.76 (1H, s, O**H**), 2.60-2.40 (2H, m, C$\mathbf{H}_2$C=O), 2.45 (3H, s, ArC$\mathbf{H}_3$), 2.08-1.86 (2H, m, C$\mathbf{H}_2$CH$_2$C=O); ^{13}C NMR (101 MHz, $CDCl_3$) δ 174.9 (C), 144.3 (C), 140.2 (C), 138.5 (C), 136.2 (C), 133.6 (C), 132.6 (C), 131.0 (2 × CH), 130.1 (2 × CH), 128.9 (2 × CH), 128.1 (2 × CH), 128.0 (2 × CH), 127.8 (2 × CH), 127.3 (CH), 127.1 (CH), 71.4 (C), 52.0 (CH_2), 52.1 (CH_3), 50.2 (CH_2), 32.0 (CH_2), 28.2 (CH_2), 21.7 (CH_3); HRMS (ESI) Exact mass calculated for $[C_{28}H_{29}NO_5SNa]^+$ $[M+Na]^+$: 514.1659, found: 514.1663; Enantiomeric excess was determined by HPLC using a Chiralpak AD-H column (90:10 *iso*-hexane:*i*-PrOH, 1.0 mL/min, 254 nm, 25 °C); t_r (minor) = 35.2 min, t_r (major) = 50.8 min, 99% ee.

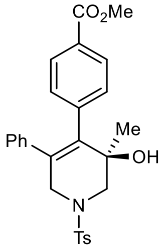

Methyl (*R*)-4-(3-hydroxy-3-methyl-5-phenyl-1-tosyl-1,2,3,6-tetrahydropyridin-4-yl)benzoate (2i). Prepared according to General Procedure C, using alkynone **1i** (120 mg, 0.30 mmol) and phenylboronic acid (73.0 mg, 0.60 mmol) at 80 °C. ^{1}H NMR analysis of the crude material showed the ratio of major:minor products was 8:1. Purification by column chromatography (5% EtOAc/CH_2Cl_2) gave **2i** (63 mg, 44%) as a white solid. R_f = 0.25 (5% EtOAc/CH_2Cl_2); m.p. 190–192 °C ($CHCl_3$); IR 3484 (OH), 2951, 1717 (C=O), 1608, 1493, 1436, 1403, 1339, 1276, 1164 cm^{-1}; $[\alpha]_D^{25}$ +132.0 (*c* 1.00, $CHCl_3$); ^{1}H NMR (500 MHz, $CDCl_3$) δ 7.80-7.75 (2H, m, Ar**H**), 7.75-7.70 (2H, m, Ar**H**), 7.38 (2H, d, *J* = 8.1 Hz, Ar**H**), 7.12 (2H, d, *J* = 7.8 Hz, Ar**H**), 7.12-7.05 (3H, m, Ar**H**), 6.91-6.84 (2H, m, Ar**H**), 4.04 (1H, d, *J* = 16.4 Hz, C$\mathbf{H}_a$H$_b$C=), 3.84 (3H, s, OC$\mathbf{H}_3$), 3.66 (1H, d, *J* = 16.4 Hz, CH$_a$$\mathbf{H}_b$C=), 3.64 (1H, d, *J* = 11.3 Hz, C$\mathbf{H}_a$H$_b$COH), 2.91 (1H, d, *J* = 11.3 Hz, CH$_a$$\mathbf{H}_b$COH), 2.64 (1H, s, O**H**), 2.47 (3H, s, ArC$\mathbf{H}_3$), 1.13 (3H, s, C$\mathbf{H}_3$COH); ^{13}C NMR (126 MHz, $CDCl_3$) δ 167.1 (C), 144.4 (C), 142.3 (C), 139.4 (C), 138.2 (C), 133.9 (C), 132.6 (C), 130.6 (2 × CH), 130.1 (2 × CH), 128.9 (2 × CH), 128.6 (C), 128.5 (2 × CH), 128.2 (2 × CH), 128.1 (2 × CH), 127.5 (CH), 69.4 (C), 56.3 (CH_2), 52.2 (CH_3), 50.1 (CH_2), 24.6 (CH_3), 21.7 (CH_3); HRMS (ESI) Exact mass calculated for $[C_{27}H_{28}NO_5S]^+$ $[M+H]^+$: 478.1683, found:

478.1678; Enantiomeric excess was determined by HPLC using a Chiralpak IC column (70:30 *iso*-hexane:*i*-PrOH, 1.0 mL/min, 210 nm, 25 °C); t_r (minor) = 31.5 min, t_r (major) = 38.2 min, 98% ee.

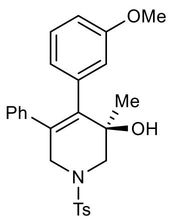

(*R*)-4-(3-Methoxyphenyl)-3-methyl-5-phenyl-1-tosyl-1,2,3,6-tetrahydropyridin-3-ol (2j). Prepared according to General Procedure C, using alkynone **1j** (111 mg, 0.30 mmol) and phenylboronic acid (73.0 mg, 0.60 mmol) at 60 °C. ^{1}H NMR analysis of the crude material showed the ratio of major:minor products was 14:1. Purification by column chromatography (0 to 2% EtOAc/CH_2Cl_2) gave **2j** (60 mg, 46%) as a white solid. R*f* = 0.19 (2% EtOAc/CH_2Cl_2); m.p. 160–162 °C ($CHCl_3$); IR 3543 (OH), 2974, 1597, 1491, 1450, 1344, 1317, 1257, 1223, 1156 cm^{-1}; $[\alpha]_D^{25}$ +108.0 (*c* 1.00, $CHCl_3$); ^{1}H NMR (500 MHz, $CDCl_3$) δ 7.75-7.68 (2H, m, Ar**H**), 7.37 (2H, d, *J* = 8.1 Hz, Ar**H**), 7.13-7.07 (3H, m, Ar**H**), 7.02 (1H, t, *J* = 7.9 Hz, Ar**H**), 6.94-6.90 (2H, m, Ar**H**), 6.67-6.61 (2H, m, Ar**H**), 6.60-6.56 (1H, m, Ar**H**), 3.98 (1H, d, *J* = 16.2 Hz, C**H**$_a$H$_b$C=), 3.70 (1H, d, *J* = 16.2 Hz, CH$_a$**H**$_b$C=), 3.63 (3H, s, OC**H**$_3$), 3.55 (1H, d, *J* = 11.3 Hz, C**H**$_a$H$_b$COH), 2.98 (1H, d, *J* = 11.3 Hz, CH$_a$**H**$_b$COH), 2.55 (1H, s, O**H**), 2.46 (3H, s, ArC**H**$_3$), 1.20 (3H, s, C**H**$_3$COH); ^{13}C NMR (126 MHz, $CDCl_3$) δ 158.9 (C), 144.2 (C), 139.9 (C), 138.7 (C), 138.3 (C), 132.9 (C), 132.6 (C), 130.0 (2 × CH), 128.6 (2 × CH), 128.6 (CH), 128.1 (2 × CH), 128.0 (2 × CH), 127.2 (CH), 123.1 (CH), 116.2 (CH), 112.5 (CH), 69.6 (C), 56.2 (CH_2), 55.2 (CH_3), 50.1 (CH_2), 24.9 (CH_3), 21.7 (CH_3); HRMS (ESI) Exact mass calculated for $[C_{26}H_{27}NO_4SNa]^+$ $[M+Na]^+$: 472.1553, found: 472.1553; Enantiomeric excess was determined by HPLC using a Chiralcel OD-H column (95:5 *iso*-hexane:*i*-PrOH, mL/min, 230 nm, 25 °C); t_r (minor) = 20.4 min, t_r (major) = 23.8 min, 98% ee.

Slow diffusion of pentane into a solution of **2j** in CH_2Cl_2 gave crystals that were suitable for X-ray crystallography:

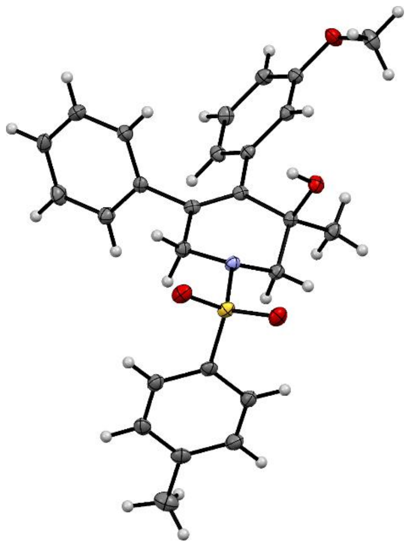

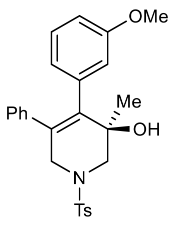

2j
CCDC 2040015
Flack parameter −0.011(16)

ORTEP with ellipsoid probabilities at 50%

(*R*)-3-Ethyl-5-phenyl-1-tosyl-4-vinyl-1,2,3,6-tetrahydropyridin-3-ol (2k) and 3-ethyl-4-(1-phenylallylidene)-1-tosylpyrrolidin-3-ol (3k)

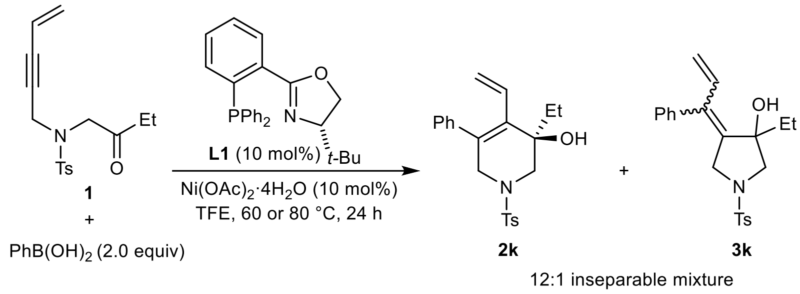

Prepared according to General Procedure C, using alkynone **1k** (92.0 mg, 0.30 mmol) and phenylboronic acid (73.0 mg, 0.60 mmol) at 60 °C. ^{1}H NMR analysis of the crude material showed the ratio of major:minor products was 12:1. Purification by column chromatography (5 to 10% EtOAc/pentane) gave a 12:1 inseparable mixture of **2k** and **3k**, respectively (103 mg, 90%), as a white solid. R_f = 0.21 (20% EtOAc/pentane); m.p. 43–45 °C ($CHCl_3$); IR 3511 (OH), 2967, 2928, 1598, 1493, 1443, 1340, 1165, 1090, 971 cm^{-1}; $[\alpha]_D^{25}$ +16.0 (*c* 1.00, $CHCl_3$); HRMS (ESI) Exact mass calculated for $[C_{22}H_{26}NO_3S]^+$ $[M+H]^+$: 384.1628, found: 384.1630.

*Data for **2k**:* ^{1}H NMR (400 MHz, $CDCl_3$) δ 7.71-7.64 (2H, m, Ar**H**), 7.40-7.28 (5H, m, Ar**H**), 7.15-7.07 (2H, m, Ar**H**), 6.08 (1H, dd, *J* = 18.0, 11.9 Hz, C**H**=CH_2), 5.42 (1H, dd, *J* = 18.0, 1.9 Hz, =C$\mathbf{H}_a$$H_b$), 5.06 (1H, dd, *J* = 11.9, 1.9 Hz, =C$H_a$$\mathbf{H}_b$), 3.76 (1H, d, *J* = 16.9 Hz, C$\mathbf{H}_a$$H_b$C=), 3.66 (1H, d, *J* = 16.9 Hz, C$H_a$$\mathbf{H}_b$C=), 3.25 (1H, d, *J* = 11.6 Hz, C$\mathbf{H}_a$$H_b$COH), 3.06 (1H, d, *J* = 11.6 Hz, C$H_a$$\mathbf{H}_b$COH), 2.44 (3H, s, ArC$\mathbf{H}_3$), 2.24 (1H, s, O**H**), 2.03-1.89 (2H, m, C$\mathbf{H}_2$$CH_3$), 0.94 (3H, t, *J* = 7.5 Hz, CH_2C$\mathbf{H}_3$); ^{13}C NMR (101 MHz, $CDCl_3$) δ 144.1 (C), 138.8 (C), 136.0 (C), 135.3 (C), 132.7 (C), 131.7 (CH), 130.0 (2 × CH) 128.9 (2 × CH), 128.6 (2 × CH), 128.0 (2 × CH), 127.9 (CH), 119.0 (CH_2), 72.8 (C), 53.4 (CH_2), 50.7 (CH_2), 29.9 (CH_2), 21.7 (CH_3), 8.1 (CH_3); Enantiomeric excess was determined by HPLC using a Chiralcel OD-H column (90:10 *iso*-hexane:*i*-PrOH, 1.5 mL/min, 254 nm, 25 °C); t_r (minor) = 6.0 min, t_r (major) = 7.1 min, 45% ee.

*Diagnostic ^{1}H NMR data for **3k**:* ^{1}H NMR (400 MHz, $CDCl_3$) δ 7.75-7.73 (1H, m, Ar**H**), 6.47 (1H, dd, *J* = 17.0, 10.5 Hz, C**H**=CH_2), 5.17 (1H, d, *J* = 10.2 Hz, =C$\mathbf{H}_a$$H_b$), 4.59 (1H, d, *J* = 17.0 Hz, =C$H_a$$\mathbf{H}_b$), 4.17-4.05 (2H, m, C$\mathbf{H}_2$C=), 3.39 (1H, d, *J* = 9.5 Hz, C$\mathbf{H}_a$$H_b$COH), 2.97 (1H, d, *J* = 9.5 Hz, C$H_a$$\mathbf{H}_b$COH), 0.78 (3H, t, *J* = 7.4 Hz, CH_2C$\mathbf{H}_3$).

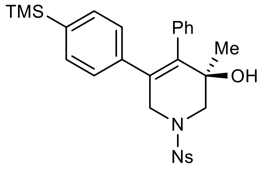

(*R*)-3-Methyl-1-[(4-nitrophenyl)sulfonyl]-4-phenyl-5-[4-(trimethylsilyl)phenyl]-1,2,3,6-tetrahydropyridin-3-ol (2l). Prepared according to General Procedure C, using alkynone **1f** (112 mg, 0.30 mmol) and 4-(trimethylsilyl)phenylboronic acid (116 mg, 0.60 mmol) at 60 °C. ^{1}H NMR

analysis of the crude material showed the ratio of major:minor products was >19:1. Purification by column chromatography (3 to 5% EtOAc/$CHCl_3$) gave **2l** (123 mg, 78%) as a white solid. R_f = 0.29 (5% EtOAc/$CHCl_3$); m.p. 180–182 °C ($CHCl_3$); IR 3517 (OH), 2956, 1604, 1528, 1401, 1348, 1331, 1248, 1169, 1112 cm^{-1}; $[\alpha]_D^{25}$ +108.0 (*c* 1.00, $CHCl_3$); ^1H NMR (400 MHz, $CDCl_3$) δ 8.46-8.39 (2H, m, Ar**H**), 8.07-7.99 (2H, m, Ar**H**), 7.28-7.21 (2H, m, Ar**H**), 7.19-7.08 (3H, m, Ar**H**), 7.07-7.00 (2H, m, Ar**H**), 6.92-6.84 (2H, m, Ar**H**), 3.98 (1H, d, *J* = 16.2 Hz, C**H**$_a$H$_b$C=), 3.81 (1H, d, *J* = 16.2 Hz, CH$_a$**H**$_b$C=), 3.57 (1H, d, *J* = 11.4 Hz, C**H**$_a$H$_b$COH), 3.14 (1H, d, *J* = 11.4 Hz, CH$_a$**H**$_b$COH), 2.39 (1H, s, O**H**), 1.23 (3H, s, C**H**$_3$COH), 0.17 (9H, s, Si(C**H**$_3$)$_3$); ^{13}C NMR (101 MHz, $CDCl_3$) δ 150.6 (C), 141.9 (C), 140.2 (C), 139.7 (C), 138.5 (C), 136.5 (C), 133.1 (2 × CH), 132.4 (C), 130.5 (2 × CH), 129.2 (2 × CH), 127.9 (2 × CH), 127.8 (2 × CH), 127.1 (CH), 124.7 (2 × CH), 69.6 (C), 55.8 (CH_2), 49.9 (CH_2), 25.2 (CH_3), –1.1 (3 × CH_3); HRMS (ESI) Exact mass calculated for $[C_{27}H_{30}N_2O_5SSiNa]^+$ $[M+Na]^+$: 545.1537, found: 545.1532; Enantiomeric excess was determined by HPLC using a Chiralcel OD-H column (90:10 *iso*-hexane:*i*-PrOH, 1.0 mL/min, 210 nm, 25 °C); t_r (minor) = 20.0 min, t_r (major) = 27.0 min, 99% ee.

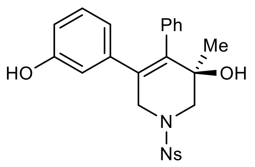

(*R*)-5-(3-Hydroxyphenyl)-3-methyl-1-[(4-nitrophenyl)sulfonyl]-4-phenyl-1,2,3,6-tetrahydropyridin-3-ol (2m). Prepared according to General Procedure C, using alkynone **1f** (112 mg, 0.30 mmol) and 3-hydroxyphenylboronic acid (83.0 mg, 0.60 mmol) at 60 °C. ^1H NMR analysis of the crude material showed the ratio of major:minor products was 16:1. Purification by column chromatography (5 to 10% EtOAc/CH_2Cl_2) gave **2m** (94 mg, 67%) as a yellow solid. R_f = 0.32 (10% EtOAc/CH_2Cl_2); m.p. 117–119 °C ($CHCl_3$); IR 3377 (OH), 3105, 1581, 1530, 1488, 1443, 1401, 1350, 1312, 1246 cm^{-1}; $[\alpha]_D^{25}$ +100.0 (*c* 1.00, $CHCl_3$); ^1H NMR (400 MHz, CD_3CN) δ 8.45-8.40 (2H, m, Ar**H**), 8.10-8.04 (2H, m, Ar**H**), 7.20-7.11 (3H, m, Ar**H**), 7.11-7.05 (2H, m, Ar**H**), 6.97 (1H, t, *J* = 7.9 Hz, Ar**H**), 6.77 (1H, br s, ArO**H**), 6.57-6.47 (2H, m, Ar**H**), 6.44 (1H, dd, *J* = 2.5, 1.6 Hz, Ar**H**), 3.93 (1H, d, *J* = 16.2 Hz, C**H**$_a$H$_b$C=), 3.72 (1H, d, *J* = 16.2 Hz, CH$_a$**H**$_b$C=), 3.54-3.49 (2H, m, C**H**$_a$H$_b$CO**H**), 3.06 (1H, d, *J* = 11.6 Hz, CH$_a$**H**$_b$COH), 1.09 (3H, s, C**H**$_3$COH); ^{13}C NMR (101 MHz, CD_3CN) δ 157.2 (C), 151.6 (C), 142.6 (C), 141.5 (C), 140.4 (C), 138.5 (C), 133.4 (C), 131.5 (2 × CH), 130.1 (2 × CH), 130.0 (CH), 128.1 (2 × CH), 127.4 (CH), 125.5 (2 × CH), 121.2 (CH), 116.7 (CH), 114.8 (CH), 69.8 (C), 56.6 (CH_2), 50.6 (CH_2), 25.1 (CH_3); HRMS (ESI) Exact mass calculated for $[C_{24}H_{22}N_2O_6SNa]^+$ $[M+Na]^+$: 489.1091, found: 489.1086; Enantiomeric excess was determined by HPLC using a Chiralcel OD-H column (80:20 *iso*-hexane:*i*-PrOH, 1.0 mL/min, 230 nm, 25 °C); t_r (minor) = 18.0 min, t_r (major) = 22.4 min, >99% ee.

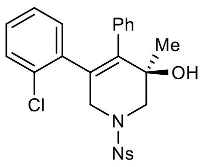

(*R*)-5-(2-Chlorophenyl)-3-methyl-1-[(4-nitrophenyl)sulfonyl]-4-phenyl-1,2,3,6-tetrahydropyridin-3-ol (2n). Prepared Prepared according to General Procedure C, using alkynone **1f** (112 mg, 0.30 mmol) and (2-chlorophenyl)boronic acid (94.0 mg, 0.60 mmol) at 60 °C. ^{1}H NMR analysis of the crude material showed the ratio of major:minor products was 19:1. Purification by column chromatography (3 to 4% EtOAc/$CHCl_3$) gave **2n** (114 mg, 79%) as a white solid. R_f = 0.17 (4% EtOAc/$CHCl_3$); m.p. 129–131 °C ($CHCl_3$); IR 3517 (OH), 2926, 1731, 1605, 1530, 1493, 1401, 1350, 1312, 1170 cm^{-1}; $[\alpha]_D^{25}$ +8.0 (*c* 1.00, $CHCl_3$); ^{1}H NMR (500 MHz, $CDCl_3$) δ 8.24-8.17 (2H, m, Ar**H**), 7.87-7.81 (1H, m, Ar**H**), 7.81-7.75 (2H, m, Ar**H**), 7.45 (1H, app td, *J* = 7.7, 1.5 Hz, Ar**H**), 7.41-7.34 (4H, m, Ar**H**), 7.28 (3H, dd, *J* = 7.8, 1.6 Hz, Ar**H**), 4.30 (1H, d, *J* = 12.3 Hz, C$\mathbf{H}_a$$H_b$C=), 3.94 (1H, d, *J* = 12.3 Hz, C$\mathbf{H}_a$$H_b$C=), 3.64 (1H, d, *J* = 15.4 Hz, C$\mathbf{H}_a$$H_b$COH), 3.36 (1H, d, *J* = 15.4 Hz, C$H_a$$\mathbf{H}_b$COH), 3.30 (1H, s, O**H**), 1.46 (3H, s, C$\mathbf{H}_3$); ^{13}C NMR (126 MHz, $CDCl_3$) δ 150.1 (C), 144.5 (C), 144.0 (C), 137.1 (C), 134.4 (C), 133.8 (C), 133.7 (C), 129.8 (CH), 129.7 (CH), 129.4 (CH), 128.9 (2 × CH), 128.8 (2 × CH), 128.2 (2 × CH), 128.0 (CH), 127.6 (CH), 124.5 (2 × CH), 76.4 (C), 57.1 (CH_2), 53.0 (CH_2), 29.8 (CH_3); HRMS (ESI) Exact mass calculated for $[C_{24}H_{21}ClN_2O_5SNa]^+$ $[M+Na]^+$: 507.0752, found: 507.0752; Enantiomeric excess was determined by HPLC using a Chiralpak AD-H column (80:20 *iso*-hexane:*i*-PrOH, 1.0 mL/min, 210 nm, 25 °C); t_r (minor) = 12.4 min, t_r (major) = 21.0 min, >99% ee.

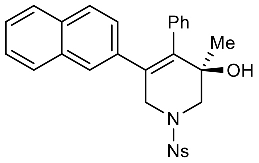

(*R*)-3-Methyl-5-(naphthalen-2-yl)-1-[(4-nitrophenyl)sulfonyl]-4-phenyl-1,2,3,6-tetrahydropyridin-3-ol (2o). Prepared according to General Procedure C, using alkynone **1f** (112 mg, 0.30 mmol) and naphthalen-2-ylboronic acid (103 mg, 0.60 mmol) at 60 °C. ^{1}H NMR analysis of the crude material showed the ratio of major:minor products was >19:1. Purification by column chromatography (3% EtOAc/$CHCl_3$) gave **2o** (77 mg, 51%) as a white solid. R_f = 0.39 (5% EtOAc/$CHCl_3$); m.p. 197–199 °C ($CHCl_3$); IR 3523 (OH), 2928, 1605, 1529, 1451, 1401, 1348, 1312,1169, 1091 cm^{-1}; $[\alpha]_D^{25}$ +136.0 (*c* 1.00, $CHCl_3$); ^{1}H NMR (500 MHz, $CDCl_3$) δ 8.45-8.39 (2H, m, Ar**H**), 8.07-8.01 (2H, m, Ar**H**), 7.72-7.63 (2H, m, Ar**H**), 7.54 (1H, d, *J* = 8.5 Hz, Ar**H**), 7.45 (1H, s, Ar**H**), 7.44-7.37 (2H, m, Ar**H**), 7.15-7.03 (5H, m, Ar**H**), 6.97 (1H, dd, *J* = 8.5, 1.8 Hz, Ar**H**), 4.07 (1H, d, *J* = 16.3 Hz, C$\mathbf{H}_a$$H_b$C=), 3.94 (1H, d, *J* = 16.3 Hz, C$H_a$$\mathbf{H}_b$C=), 3.61 (1H, d, *J* = 11.5 Hz, C$\mathbf{H}_a$$H_b$COH), 3.20 (1H, d, *J* = 11.5 Hz, C$H_a$$\mathbf{H}_b$COH), 2.45 (1H, s, O**H**), 1.27 (3H, s, C$\mathbf{H}_3$); ^{13}C NMR (126 MHz, $CDCl_3$) δ 150.6 (C), 141.9 (C), 140.7 (C), 136.4 (C), 135.7 (C), 132.9 (C), 132.4 (C), 132.3 (C), 130.5 (2 × CH), 129.2 (2 × CH), 127.9 (2 × CH), 127.9 (CH), 127.8 (CH), 127.7 (CH), 127.5 (CH), 127.2 (CH), 126.8 (CH), 126.37 (CH), 126.35 (CH), 124.7 (2 × CH), 69.7 (C), 55.8

(CH_2), 50.0 (CH_2), 25.2 (CH_3); HRMS (ESI) Exact mass calculated for $[C_{28}H_{24}N_2O_5SNa]^+$ $[M+Na]^+$: 523.1298, found: 523.1305; Enantiomeric excess was determined by HPLC using a Chiralcel OD-H column (80:20 *iso*-hexane:*i*-PrOH, 1.0 mL/min, 210 nm, 25 °C); t_r (major) = 27.6 min, t_r (minor) = 34.7 min, >99% ee.

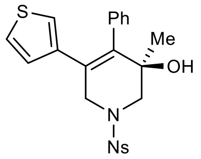

(*R*)-3-Methyl-1-[(4-nitrophenyl)sulfonyl]4-phenyl-5-(thiophen-3-yl)-1,2,3,6-tetrahydropyridin-3-ol (2p). Prepared according to General Procedure C, using alkynone **1f** (112 mg, 0.30 mmol) and 3-thienylboronic acid (77.0 mg, 0.60 mmol) at 60 °C. ^{1}H NMR analysis of the crude material showed the ratio of major:minor products was >19:1. Purification by column chromatography (4% EtOAc/$CHCl_3$) gave **2p** (87 mg, 64%) as a white solid. R_f = 0.20 (3% EtOAc/$CHCl_3$); m.p. 167–169 °C ($CHCl_3$); IR 3521 (OH), 3104, 2927, 1605, 1530, 1454, 1401, 1350, 1169, 1091 cm^{-1}; $[\alpha]_D^{25}$ +84.0 (*c* 1.00, $CHCl_3$); ^{1}H NMR (400 MHz, $CDCl_3$) δ 8.48-8.39 (2H, m, Ar**H**), 8.10-8.02 (2H, m, Ar**H**), 7.26-7.22 (3H, m, Ar**H**), 7.12-7.04 (2H, m, Ar**H**), 7.01 (1H, dd, *J* = 5.1, 3.0 Hz, Ar**H**), 6.78 (1H, dd, *J* = 2.9, 1.4 Hz, Ar**H**), 6.47 (1H, dd, *J* = 5.0, 1.4 Hz, Ar**H**), 4.05 (1H, d, *J* = 15.9 Hz, C$\mathbf{H}_a$$H_b$C=), 3.90 (1H, d, *J* = 15.9 Hz, C$H_a$$\mathbf{H}_b$C=), 3.52 (1H, d, *J* = 11.5 Hz, C$\mathbf{H}_a$$H_b$COH), 3.17 (1H, d, *J* = 11.5 Hz, C$H_a$$\mathbf{H}_b$COH), 2.31 (1H, s, O**H**), 1.23 (3H, s, C$\mathbf{H}_3$COH); ^{13}C NMR (101 MHz, $CDCl_3$) δ 150.6 (C), 142.0 (C), 140.1 (C), 138.0 (C), 137.1 (C), 130.2 (2 × CH), 129.2 (2 × CH), 128.3 (2 × CH), 127.7 (CH), 127.6 (CH), 126.7 (C), 125.0 (CH), 124.7 (2 × CH), 123.7 (CH), 69.7 (C), 55.6 (CH_2), 49.2 (CH_2), 25.2 (CH_3); HRMS (ESI) Exact mass calculated for $[C_{22}H_{20}N_2O_5S_2Na]^+$ $[M+Na]^+$: 479.0706, found: 479.0705; Enantiomeric excess was determined by HPLC using a Chiralpak IC column (80:20 *iso*-hexane:*i*-PrOH, 1.0 mL/min, 254 nm, 25 °C); t_r (minor) = 23.7 min, t_r (major) = 36.4 min, 98% ee.

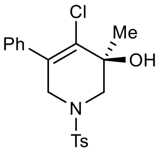

(*S*)-4-Chloro-3-methyl-5-phenyl-1-tosyl-1,2,3,6-tetrahydropyridin-3-ol (2q). Prepared according to General Procedure C, using alkynone **1l** (89.8 mg, 0.30 mmol) and phenylboronic acid (73.0 mg, 0.60 mmol) at 60 °C. The ratio of major:minor products could not be determined by ^{1}H NMR analysis of the crude material. Purification by column chromatography (5% EtOAc/$CHCl_3$) gave **2q** (13.1 mg, 12%) as a yellow solid. R_f = 0.29 (5% EtOAc/$CHCl_3$); m.p. 58–60 °C ($CHCl_3$); IR 3480 (OH), 2928, 1598, 1536, 1493, 1350, 1308, 1253, 1166, 1092 cm^{-1}; $[\alpha]_D^{20}$ –4.0 (*c* 1.00, $CHCl_3$); ^{1}H NMR (400 MHz, $CDCl_3$) δ 7.67 (2H, d, *J* = 8.1 Hz, Ar**H**), 7.43-7.31 (5H, m, Ar**H**), 7.25-7.20 (2H, m, Ar**H**), 3.87 (1H, d, *J* = 16.2 Hz, C$\mathbf{H}_a$$H_b$C=), 3.71 (1H, d, *J* = 16.2 Hz, C$H_a$$\mathbf{H}_b$C=), 3.36 (1H, d, *J* = 11.6 Hz, C$\mathbf{H}_a$$H_b$COH), 3.23 (1H, d, *J* = 11.6 Hz, C$H_a$$\mathbf{H}_b$COH), 2.58 (1H, s, O**H**), 2.44 (3H, s, ArC$\mathbf{H}_3$), 1.54 (3H, s, C$\mathbf{H}_3$COH); ^{13}C NMR (101 MHz, $CDCl_3$) δ 144.4 (C), 136.4 (C), 133.2 (C), 132.7 (C), 132.4 (C), 130.1 (2 × CH), 128.7 (2 × CH),

128.6 (CH), 128.5 (2 × CH), 127.9 (2 × CH), 70.8 (C), 55.9 (CH_2), 51.1 (CH_2), 24.8 (CH_3), 21.7 (CH_3); HRMS (ESI) Exact mass calculated for $[C_{19}H_{20}NO_3ClSNa]^+$ $[M+Na]^+$: 400.0745, found: 400.0751; Enantiomeric excess was determined by HPLC using a Chiralpak AD-H column (90:10 *iso*-hexane:*i*-PrOH, 1.0 mL/min, 210 nm, 25 °C); t_r (minor) = 28.3 min, t_r (major) = 31.8 min, 71% ee.

Dimethyl (*R*)-2'-hydroxy-6'-phenyl-2',5'-dihydro-[1,1':2',1''-terphenyl]-4',4'(3'*H*)-dicarboxylate (26) and methyl (*S*)-2-oxo-1-(2-oxo-2-phenylethyl)-3,4-diphenylcyclopent-3-ene-1-carboxylate (27a)

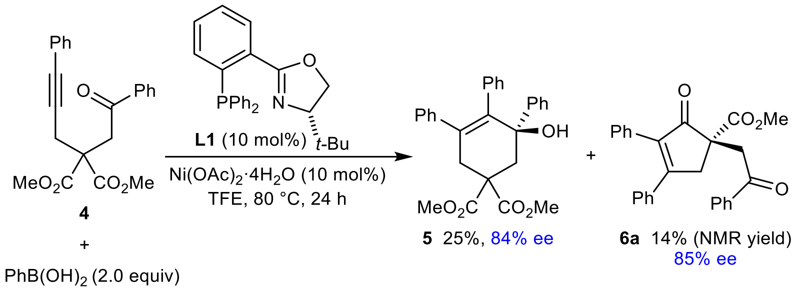

Prepared according to General Procedure C, using alkynone **4** (109 mg, 0.30 mmol) and phenylboronic acid (73.0 mg, 0.60 mmol) at 80 °C. Purification by column chromatography (85% $CHCl_3$/pentane to $CHCl_3$) gave **6a** as a colorless oil (27.4 mg; additional inseparable, unidentified impurities were present, and therefore the yield was calculated by ^{1}H NMR analysis using 1,3,5-trimethoxybenzene as an internal standard, *ca.* 65% purity. Calculated yield = 14%) followed by **5** (33.6 mg, 25%) as a colorless solid.

Data for **5**: R_f = 0.44 (1% EtOAc/$CHCl_3$); m.p. 50–52 °C ($CHCl_3$); IR 3496 (OH), 3056, 2854, 1731 (C=O), 1559, 1491, 1441, 1256, 1212, 909 cm^{-1}; $[\alpha]_D^{20}$ +36.0 (*c* 1.00, $CHCl_3$); ^{1}H NMR (400 MHz, $CDCl_3$) δ 7.50-7.44 (2H, m, Ar**H**), 7.33 (2H, t, *J* = 7.6 Hz, Ar**H**), 7.27-7.23 (1H, m, Ar**H**), 7.18-7.00 (5H, m, Ar**H**), 6.96-6.80 (3H, m, Ar**H**), 6.58-6.48 (2H, m, Ar**H**), 3.88 (3H, s, C**H**$_3$), 3.72 (3H, s, C**H**$_3$), 3.35 (1H, dd, *J* = 17.4, 2.6 Hz, C**H**$_a$H$_b$C=), 3.14 (1H, s, O**H**), 3.00 (1H, d, *J* = 17.4 Hz, CH$_a$**H**$_b$C=), 2.97 (1H, dd, *J* = 14.8, 2.6 Hz, C**H**$_a$H$_b$COH), 2.70 (1H, d, *J* = 14.8 Hz, CH$_a$**H**$_b$COH); ^{13}C NMR (101 MHz, $CDCl_3$) δ 172.7 (C), 171.7 (C), 146.6 (C), 142.1 (C), 139.6 (C), 137.9 (C), 136.3 (C), 131.5 (2 × CH), 129.1 (2 × CH), 128.2 (2 × CH), 127.9 (2 × CH), 127.2 (2 × CH), 127.0 (CH), 126.7 (CH), 126.6 (CH), 126.1 (2 × CH), 75.0 (C), 53.3 (C), 53.07 (CH_3), 53.04 (CH_3), 46.1 (CH_2), 38.3 (CH_2); HRMS (ESI) Exact mass calculated for $[C_{28}H_{26}O_5Na]^+$ $[M+Na]^+$: 465.1672, found: 465.1670; Enantiomeric excess was determined by HPLC using a Chiralpak AD-H column (90:10 *iso*-hexane:*i*-PrOH, 1.0 mL/min, 210 nm, 25 °C); t_r (minor) = 8.8 min, t_r (major) = 11.3 min, 84% ee.

Data for **6a**: R_f = 0.46 (1% EtOAc/$CHCl_3$); IR 2924, 2854, 1737 (C=O), 1688 (C=O), 1597, 1447, 1403, 1353, 1202, 1153 cm^{-1}; $[\alpha]_D^{20}$ +48.0 (*c* 1.00, $CHCl_3$); ^1H NMR (400 MHz, $CDCl_3$) δ 8.06-7.95 (2H, m, Ar**H**), 7.60-7.56 (1H, m, Ar**H**), 7.48 (2H, t, *J* = 7.6 Hz, Ar**H**), 7.43-7.27 (10H, m, Ar**H**), 4.28 (1H, d, *J* = 18.3 Hz, C$\mathbf{H}_a$$H_b$C=O), 4.10 (1H, d, *J* = 18.6 Hz, =CC$\mathbf{H}_a$$H_b$), 3.73 (3H, s, C$\mathbf{H}_3$), 3.33 (1H, d, *J* = 18.3 Hz, $CH_a$$\mathbf{H}_b$C=O), 3.04 (1H, d, *J* = 18.6 Hz, =C$CH_a$$\mathbf{H}_b$); ^{13}C NMR (101 MHz, $CDCl_3$) δ 202.4 (C), 197.3 (C), 170.3 (C), 168.3 (C), 136.7 (C), 136.4 (C), 135.0 (C), 133.7 (CH), 132.0 (C), 130.6 (CH), 129.7 (2 × CH), 128.9 (2 × CH), 128.8 (CH), 128.7 (2 × CH), 128.61 (2 × CH), 128.59 (2 × CH), 128.3 (2 × CH), 56.7 (C), 53.2 (CH_3), 44.5 (CH_2), 41.7 (CH_2); HRMS (ESI) Exact mass calculated for $[C_{27}H_{22}O_4Na]^+$ [M+Na] : 433.1410, found: 433.1410; Enantiomeric excess was determined by HPLC using a Chiralpak AD-H column (90:10 *iso*-hexane:*i*-PrOH, 1.0 mL/min, 210 nm, 25 °C); t_r (minor) = 6.5 min, t_r (minor) = 7.9 min, 85% ee.

Methyl (*R*)-2-oxo-1-(3-oxobutyl)-3,4-diphenylcyclopent-3-ene-1-carboxylate (6b)

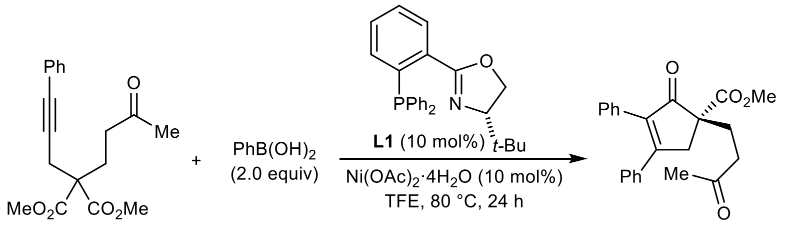

Prepared according to General Procedure C, using alkynone **8** (94.9 mg, 0.30 mmol) and phenylboronic acid (73.0 mg, 0.60 mmol) at 80 °C. Purification by column chromatography (1 to 5% EtOAc/$CHCl_3$) gave **6b** (59.8 mg, 55%) as an orange oil. R_f = 0.22 (2% EtOAc/$CHCl_3$); IR 3055, 2953, 1739 (C=O), 1698 (C=O), 1623, 1574, 1444, 1352, 1252, 1198 cm^{-1}; $[\alpha]_D^{20}$ –8.0 (*c* 1.00, $CHCl_3$); ^1H NMR (400 MHz, $CDCl_3$) δ 7.36-7.27 (8H, m, Ar**H**), 7.23-7.21 (2H, m, Ar**H**), 3.75 (3H, s, OC$\mathbf{H}_3$), 3.59 (1H, d, *J* = 18.1 Hz, =CC$\mathbf{H}_a$$H_b$), 2.92 (1H, d, *J* = 18.1 Hz, =C$CH_a$$\mathbf{H}_b$), 2.74 (1H, ddd, *J* = 17.6, 9.7, 6.0 Hz, C$\mathbf{H}_a$$H_b$C=O), 2.58 (1H, ddd, *J* = 17.6, 9.1, 6.0 Hz, $CH_a$$\mathbf{H}_b$C=O), 2.42-2.20 (2H, m, C$\mathbf{H}_2$$CH_2$C=O), 2.15 (3H, s, C$\mathbf{H}_3$C=O); ^{13}C NMR (101 MHz, $CDCl_3$) δ 207.8 (C), 203.0 (C), 171.6 (C), 166.4 (C), 137.5 (C), 134.8 (C), 131.7 (C), 130.5 (CH), 129.6 (2 × CH), 128.63 (2 × CH), 128.60 (2 × CH), 128.34 (2 × CH), 128.29 (CH), 57.4 (C), 52.9 (CH_3), 41.3 (CH_2), 38.9 (CH_2), 30.1 (CH_3), 29.0 (CH_2); HRMS (ESI) Exact mass calculated for $[C_{23}H_{22}O_4Na]^+$ $[M+Na]^+$: 385.1410, found: 385.1411; Enantiomeric excess was determined by HPLC using a Chiralpak AD-H column (90:10 *iso*-hexane:*i*-PrOH, 1.0 mL/min, 230 nm, 25 °C); t_r (minor) = 15.2 min, t_r (major) = 22.4 min, 19% ee.

(*Z*)-*N*-(2,3-Diphenylallyl)-4-methyl-*N*-(3-oxobutyl)benzenesulfonamide [(*Z*)-10]

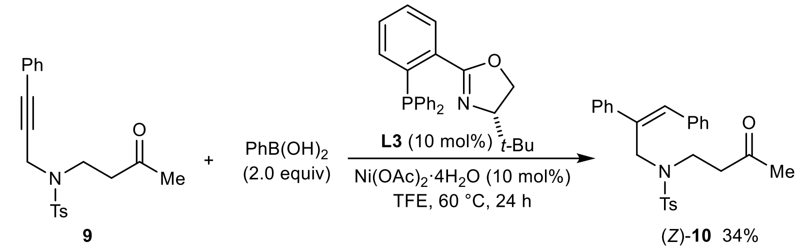

Prepared according to General Procedure C, using alkynone **9** (107 mg, 0.30 mmol) and phenylboronic acid (73.0 mg, 0.60 mmol) at 60 °C. Purification by column chromatography (15% EtOAc/pet. ether) gave (*Z*)-**10** (44.2 mg, 34%) as a colorless solid. R_f = 0.32 (20% EtOAc/pet. ether); m.p. 65–67 °C ($CHCl_3$); IR 3056, 2922, 1713 (C=O), 1598, 1495, 1343, 1305, 1154, 1104, 1020 cm^{-1}; ^{1}H NMR (400 MHz, $CDCl_3$) δ 7.53-7.44 (2H, m, Ar**H**), 7.41 (2H, d, *J* = 8.2 Hz, Ar**H**), 7.36-7.32 (5H, m, Ar**H**), 7.29 (1H, d, *J* = 7.3 Hz, Ar**H**), 7.22-7.17 (2H, m, Ar**H**), 7.16 (2H, d, *J* = 8.1 Hz, Ar**H**), 7.01 (1H, s, =C**H**), 4.48 (2H, s, =CC**H**$_2$), 3.05-2.95 (2H, m, NC**H**$_2$CH$_2$), 2.38 (3H, s, ArC**H**$_3$), 2.25-2.17 (2H, m, C**H**$_2$C=O), 1.86 (3H, s, C**H**$_3$C=O); ^{13}C NMR (101 MHz, $CDCl_3$) δ 207.0 (C), 143.4 (C), 140.0 (C), 137.0 (C), 136.5 (C), 135.3 (C), 133.7 (CH), 129.6 (2 × CH), 129.0 (2 × CH), 128.6 (2 × CH), 128.5 (2 × CH), 127.9 (CH), 127.54 (2 × CH), 127.45 (CH), 127.3 (2 × CH), 45.6 (CH_2), 42.0 (CH_2), 41.0 (CH_2), 30.0 (CH_3), 21.6 (CH_3); HRMS (ESI) Exact mass calculated for $[C_{26}H_{27}NO_3SNa]^+$ $[M+Na]^+$: 456.1602, found: 456.1602.

Assignment of the alkene stereochemistry of (*Z*)-**10** was made based on the following NOE interactions observed in a series of 1D NOESY experiments:

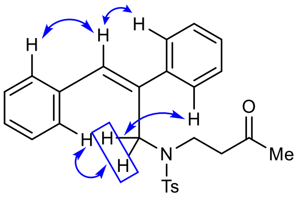

(*Z*)-**10**

4. NMR Spectra of New Compounds

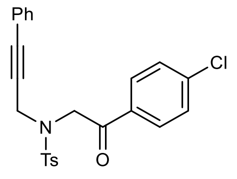

1b

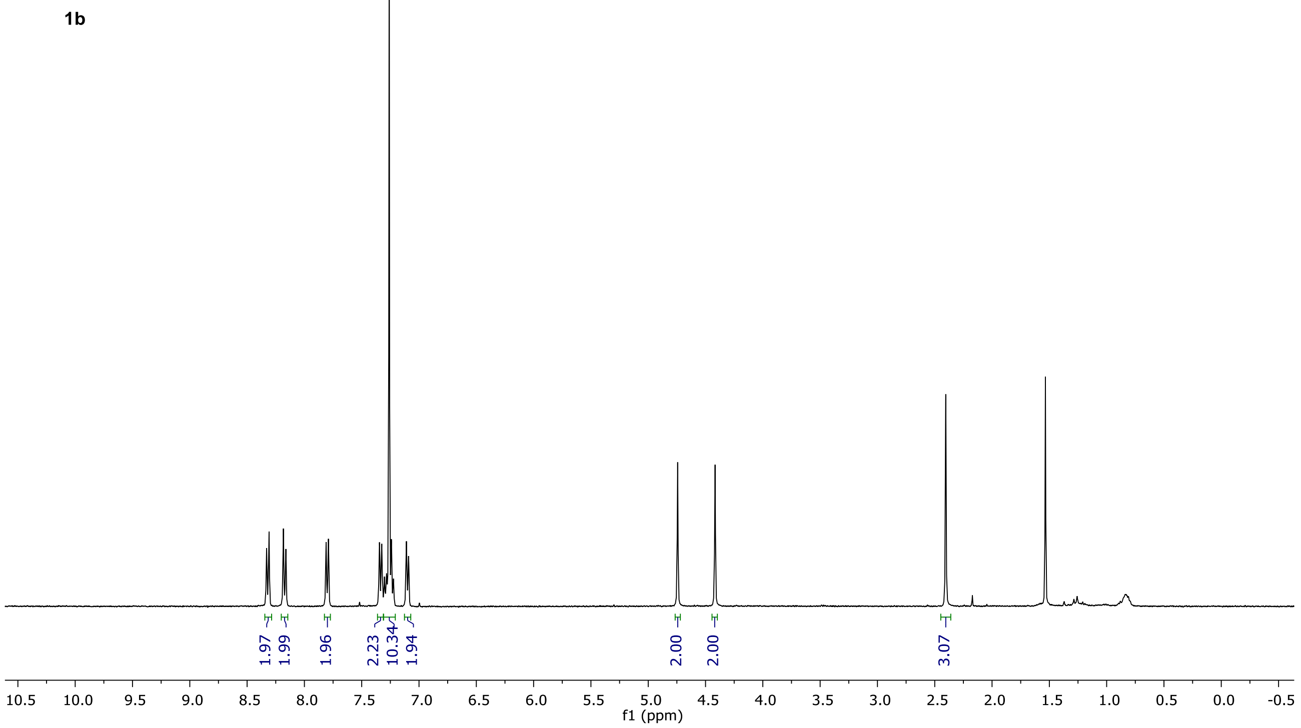

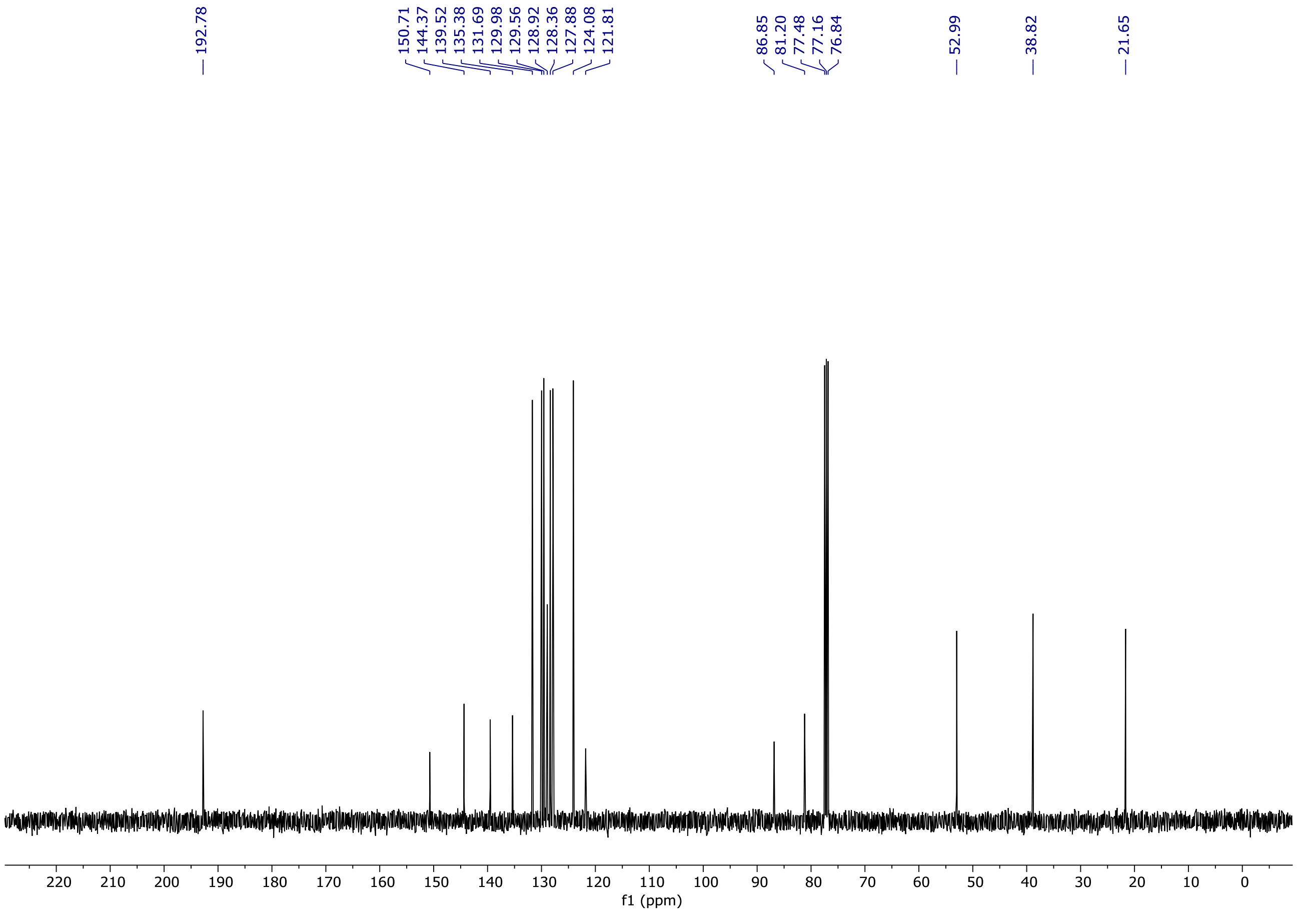

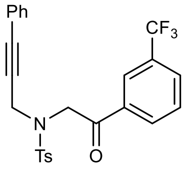
Ph
CF3
N
Ts
O

1c

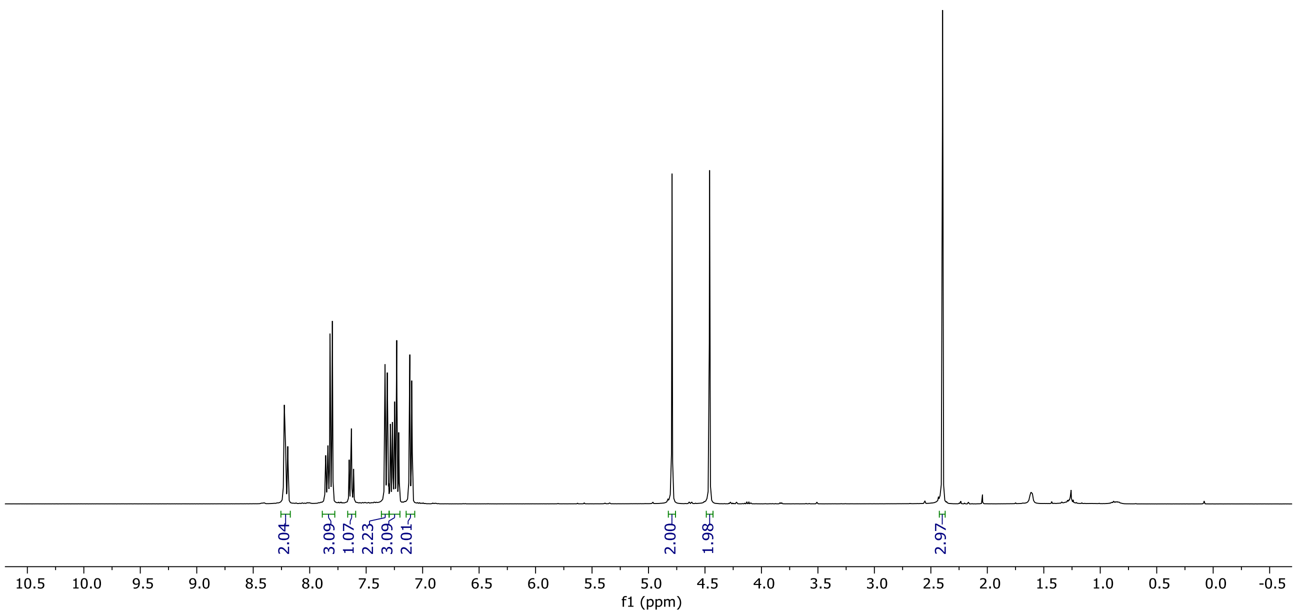
2.04
3.09
1.07
2.23
3.09
2.01
2.00
1.98
2.97
f1 (ppm)

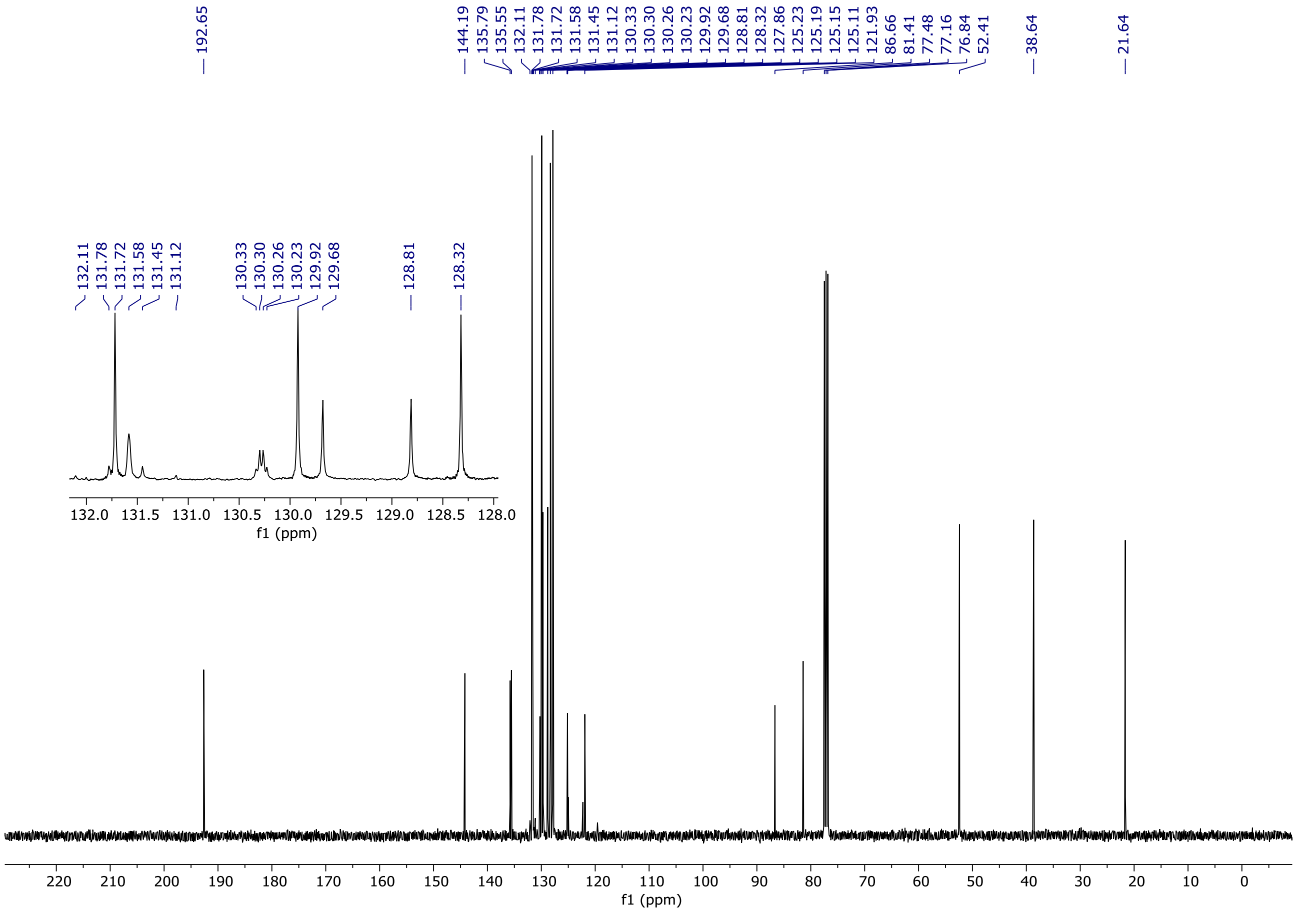
192.65
144.19
135.79
135.55
132.11
131.78
131.72
131.58
131.45
131.12
130.33
130.30
130.26
130.23
129.92
129.68
128.81
128.32
127.86
125.23
125.19
125.15
125.11
121.93
86.66
81.41
77.48
77.16
76.84
52.41
38.64
21.64
f1 (ppm)

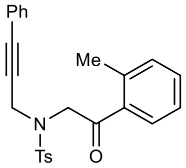
Ph
Me
N
Ts
O

1d

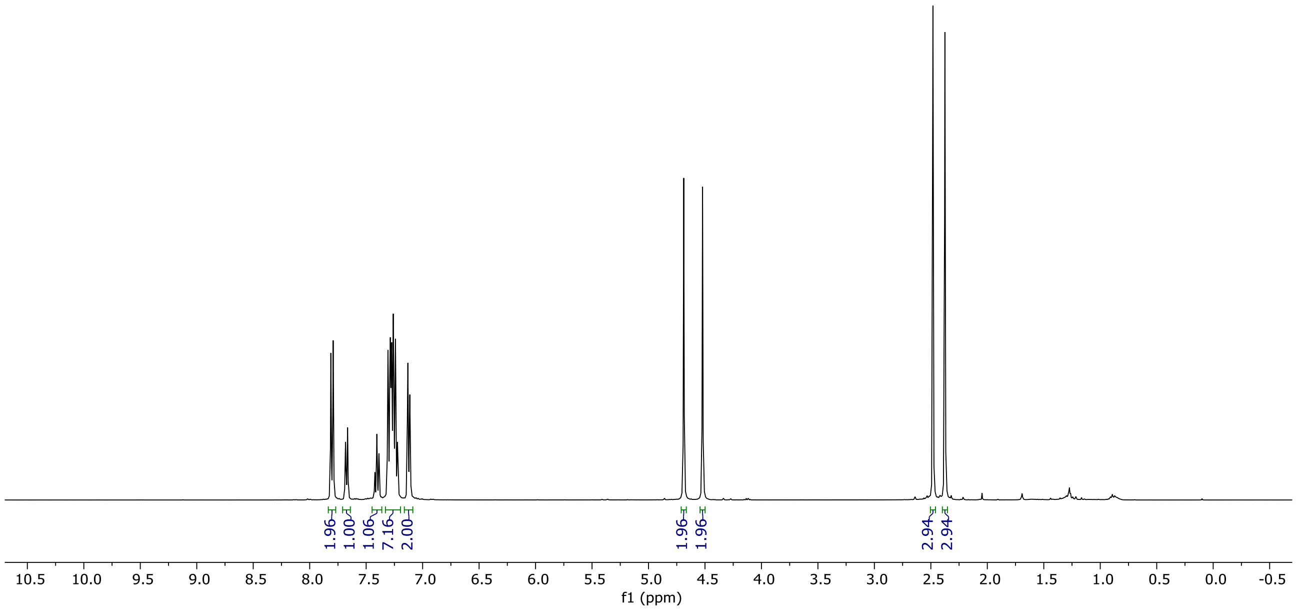
1.96
1.00
1.06
7.16
2.00
1.96
1.96
2.94
2.94
f1 (ppm)

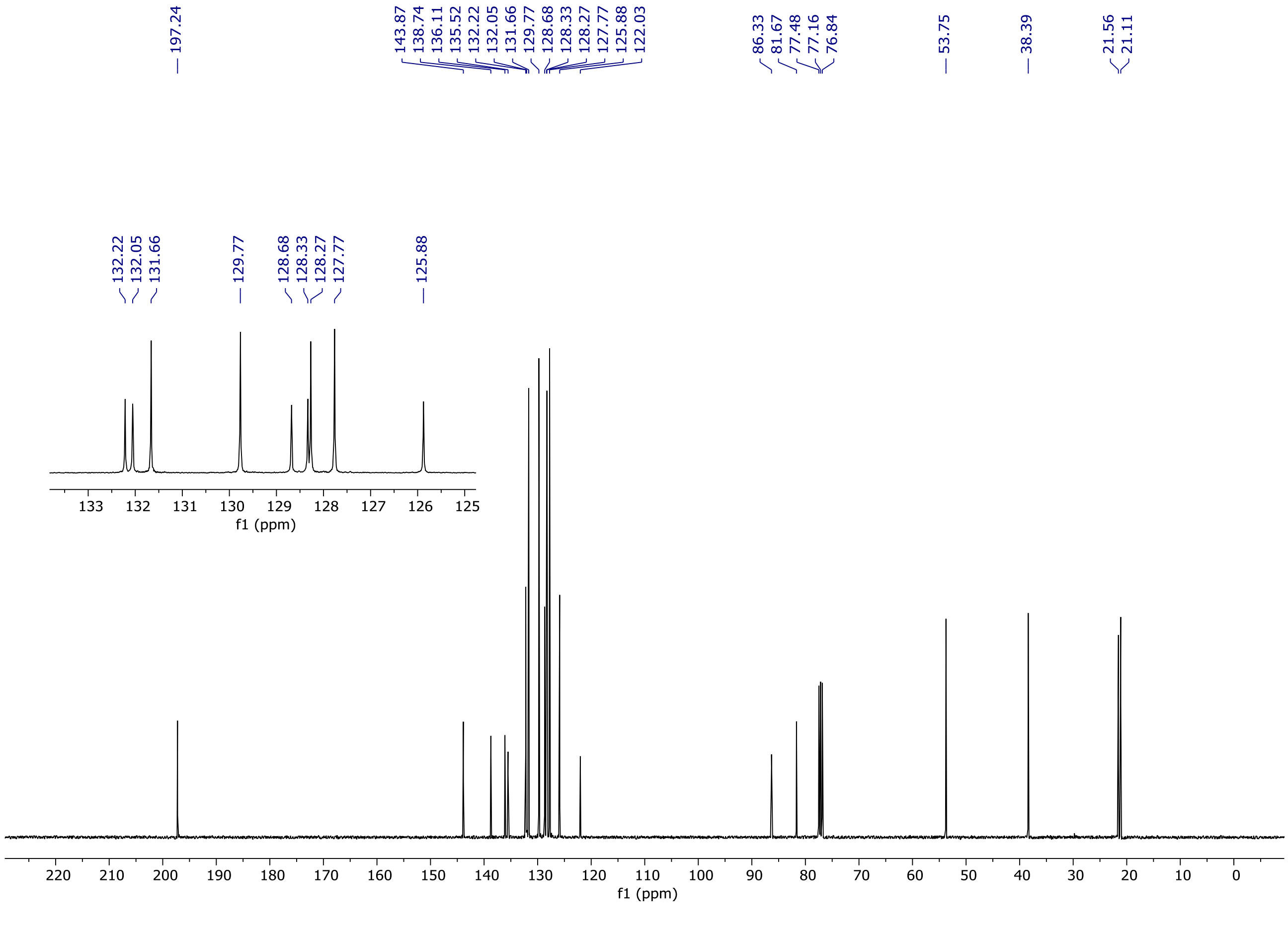
197.24
143.87
138.74
136.11
135.52
132.22
132.05
131.66
129.77
128.68
128.33
128.27
127.77
125.88
122.03
86.33
81.67
77.48
77.16
76.84
53.75
38.39
21.56
21.11
f1 (ppm)

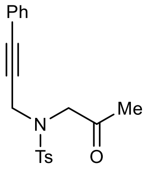
Ph
Me
N
Ts
O

1e

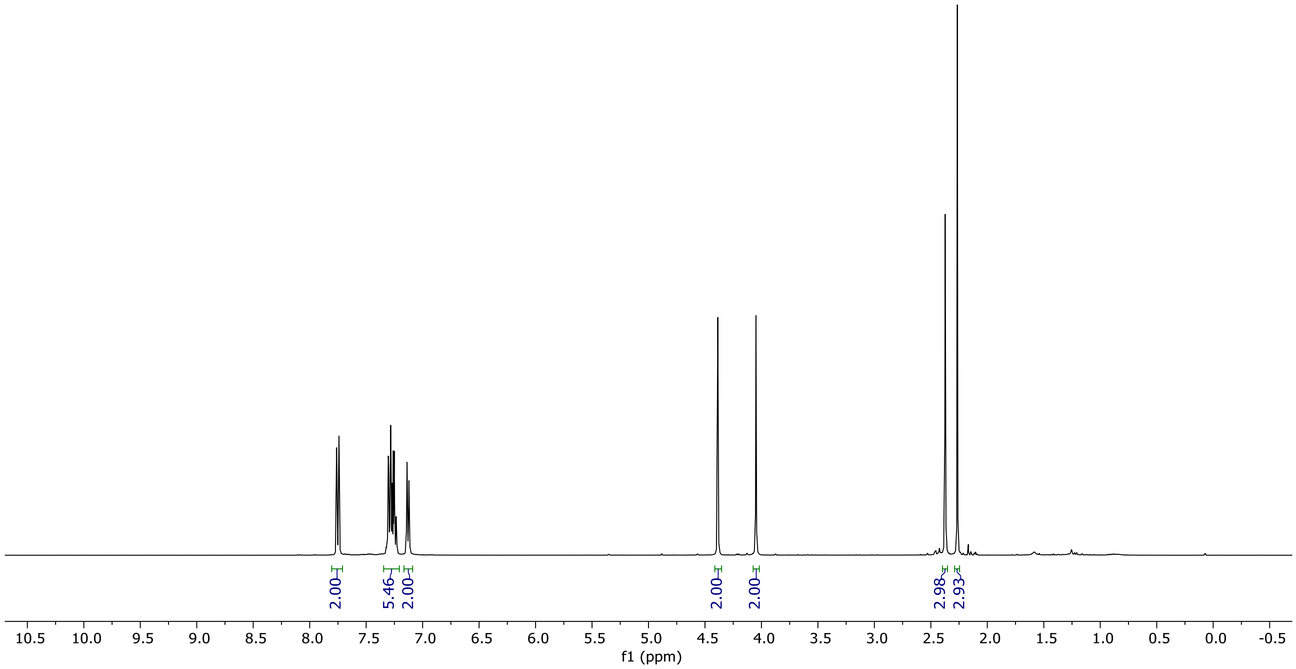
2.00
5.46
2.00
2.00
2.00
2.98
2.93
f1 (ppm)

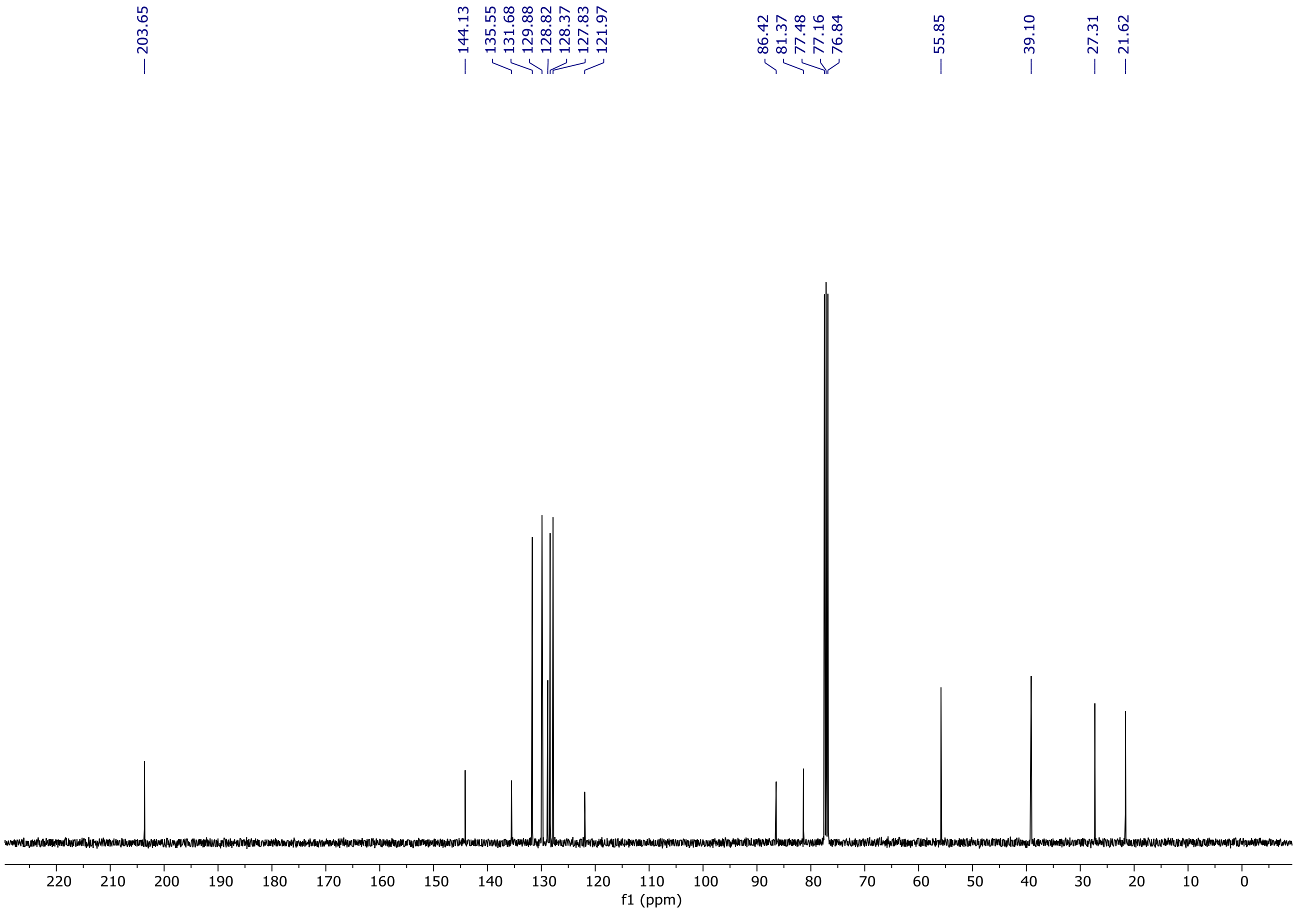
203.65
144.13
135.55
131.68
129.88
128.82
128.37
127.83
121.97
86.42
81.37
77.48
77.16
76.84
55.85
39.10
27.31
21.62
f1 (ppm)

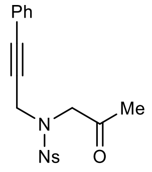
Ph
Me
N
Ns
O

1f

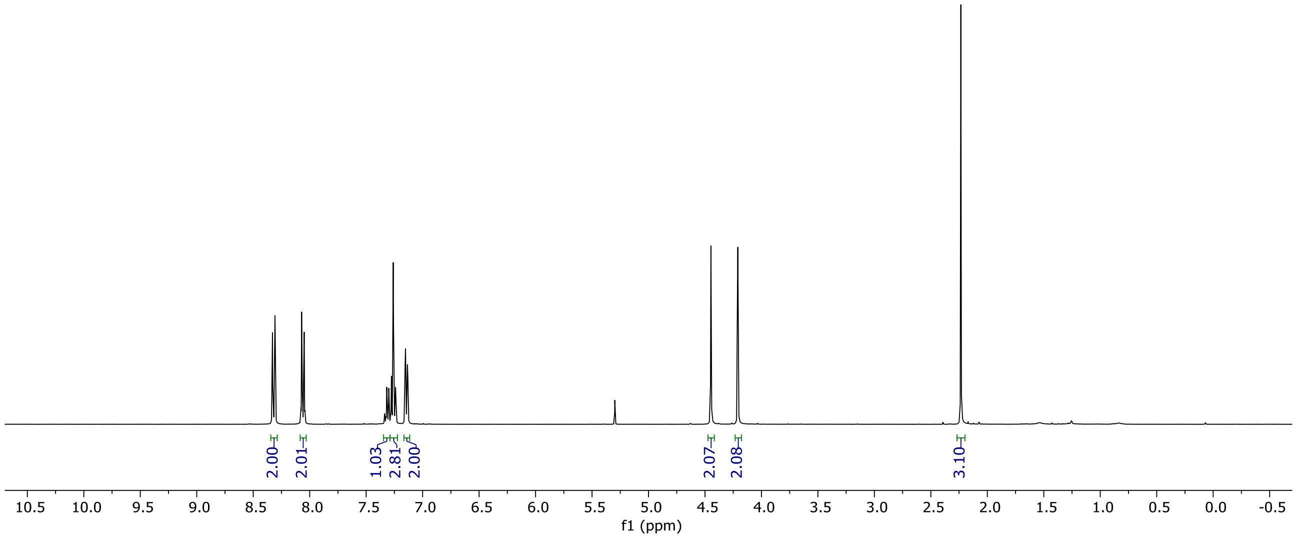
2.00
2.01
1.03
2.81
2.00
2.07
2.08
3.10
10.5 10.0 9.5 9.0 8.5 8.0 7.5 7.0 6.5 6.0 5.5 5.0 4.5 4.0 3.5 3.0 2.5 2.0 1.5 1.0 0.5 0.0 -0.5
f1 (ppm)

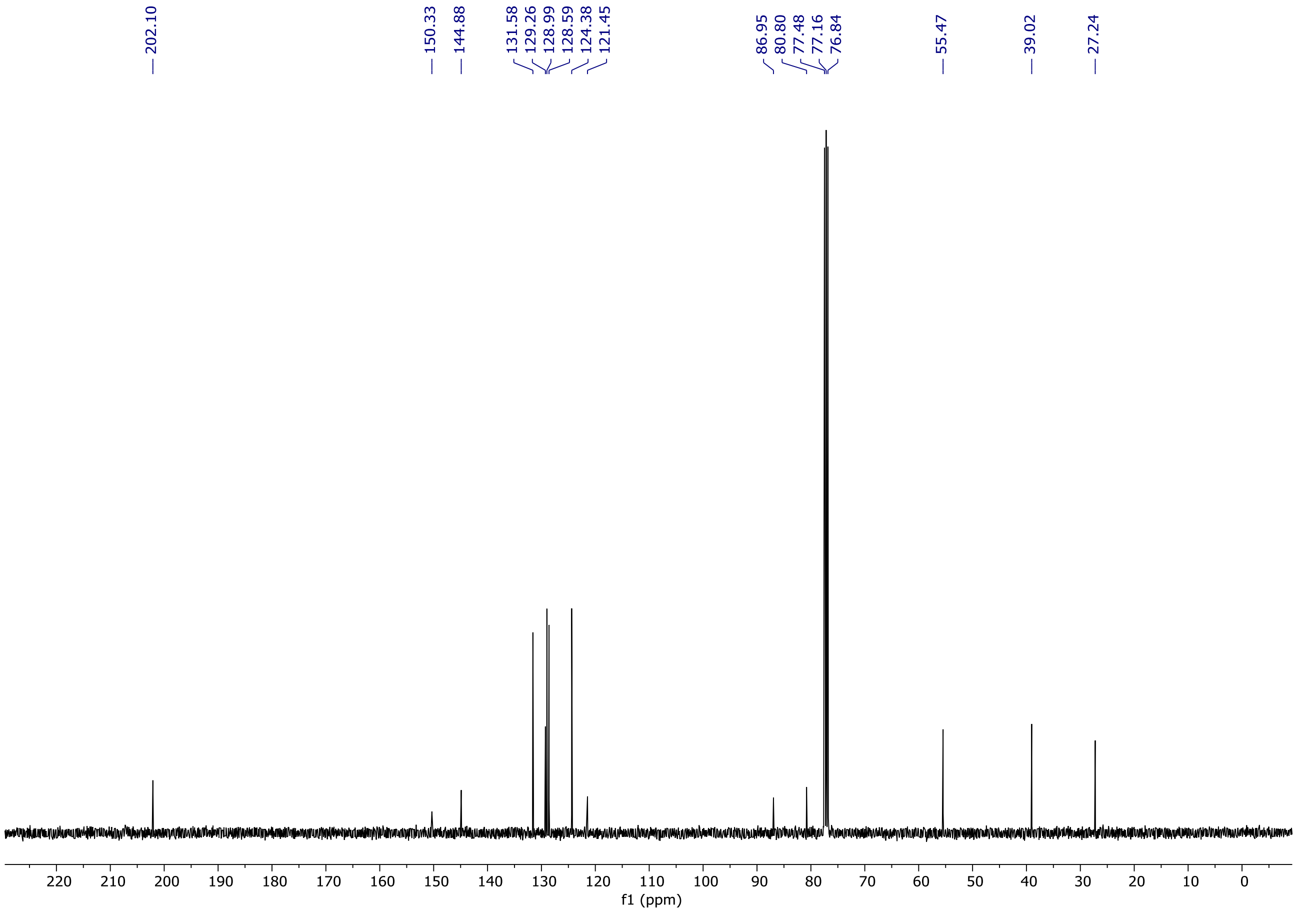
202.10
150.33
144.88
131.58
129.26
128.99
128.59
124.38
121.45
86.95
80.80
77.48
77.16
76.84
55.47
39.02
27.24
220 210 200 190 180 170 160 150 140 130 120 110 100 90 80 70 60 50 40 30 20 10 0
f1 (ppm)

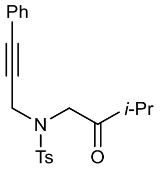
Ph
N
i-Pr
Ts
O

1g

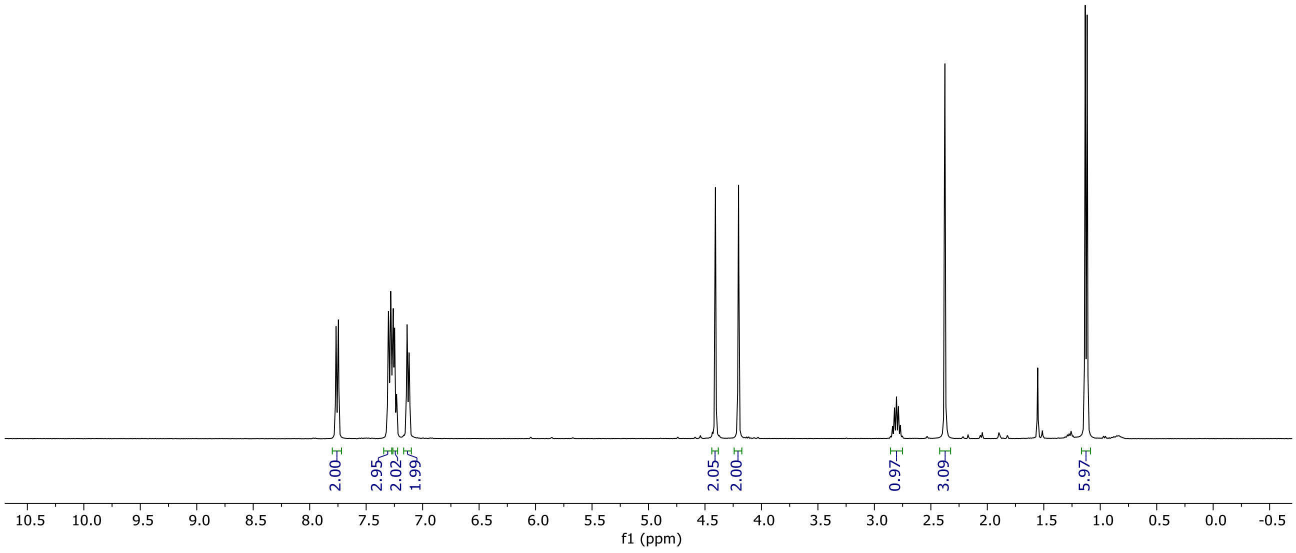
2.00
2.95
2.02
1.99
2.05
2.00
0.97
3.09
5.97
10.5 10.0 9.5 9.0 8.5 8.0 7.5 7.0 6.5 6.0 5.5 5.0 4.5 4.0 3.5 3.0 2.5 2.0 1.5 1.0 0.5 0.0 -0.5
f1 (ppm)

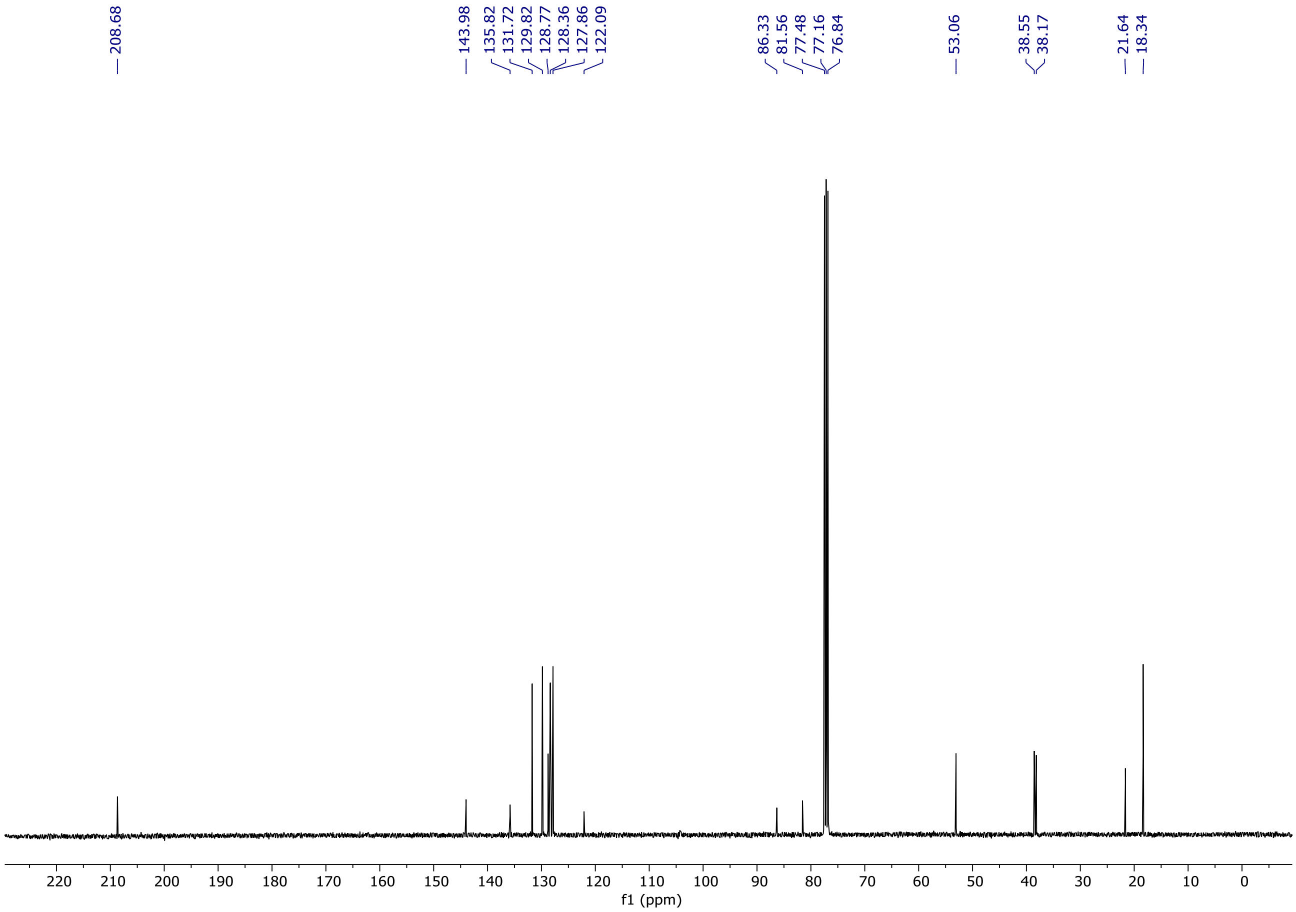
208.68
143.98
135.82
131.72
129.82
128.77
128.36
127.86
122.09
86.33
81.56
77.48
77.16
76.84
53.06
38.55
38.17
21.64
18.34
220 210 200 190 180 170 160 150 140 130 120 110 100 90 80 70 60 50 40 30 20 10 0
f1 (ppm)

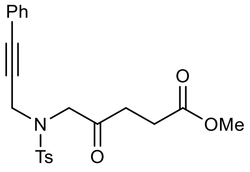
Ph
N
Ts
O
O
OMe

1h

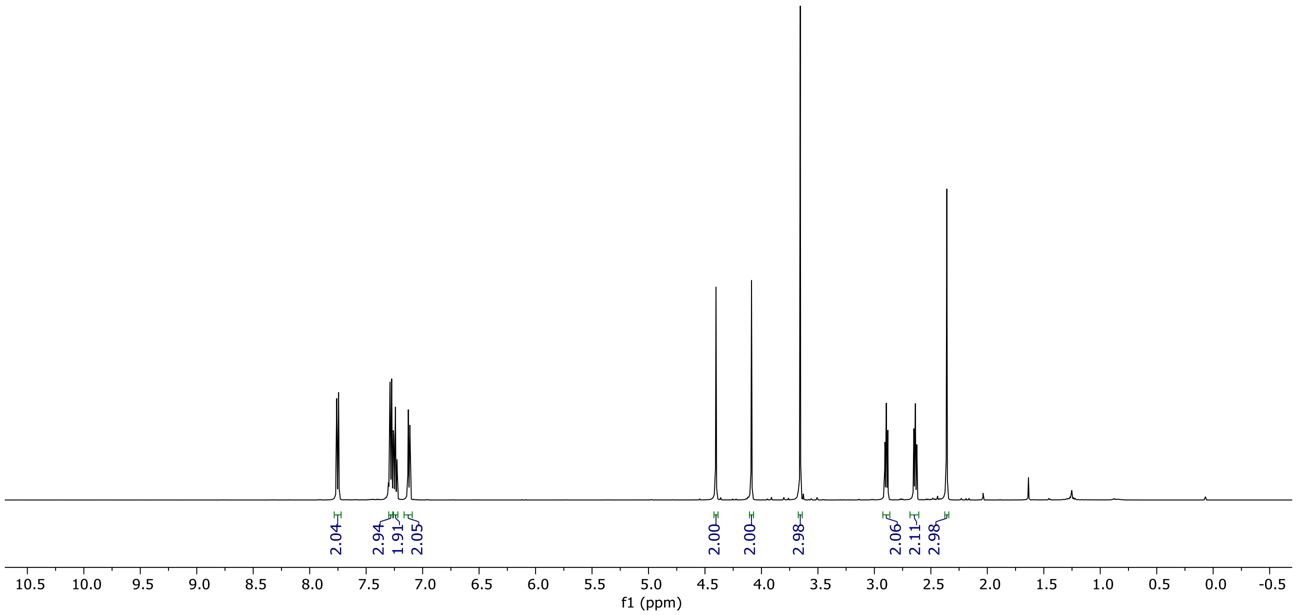
2.04
2.94
1.91
2.05
2.00
2.00
2.98
2.06
2.11
2.98
10.5 10.0 9.5 9.0 8.5 8.0 7.5 7.0 6.5 6.0 5.5 5.0 4.5 4.0 3.5 3.0 2.5 2.0 1.5 1.0 0.5 0.0 -0.5
f1 (ppm)

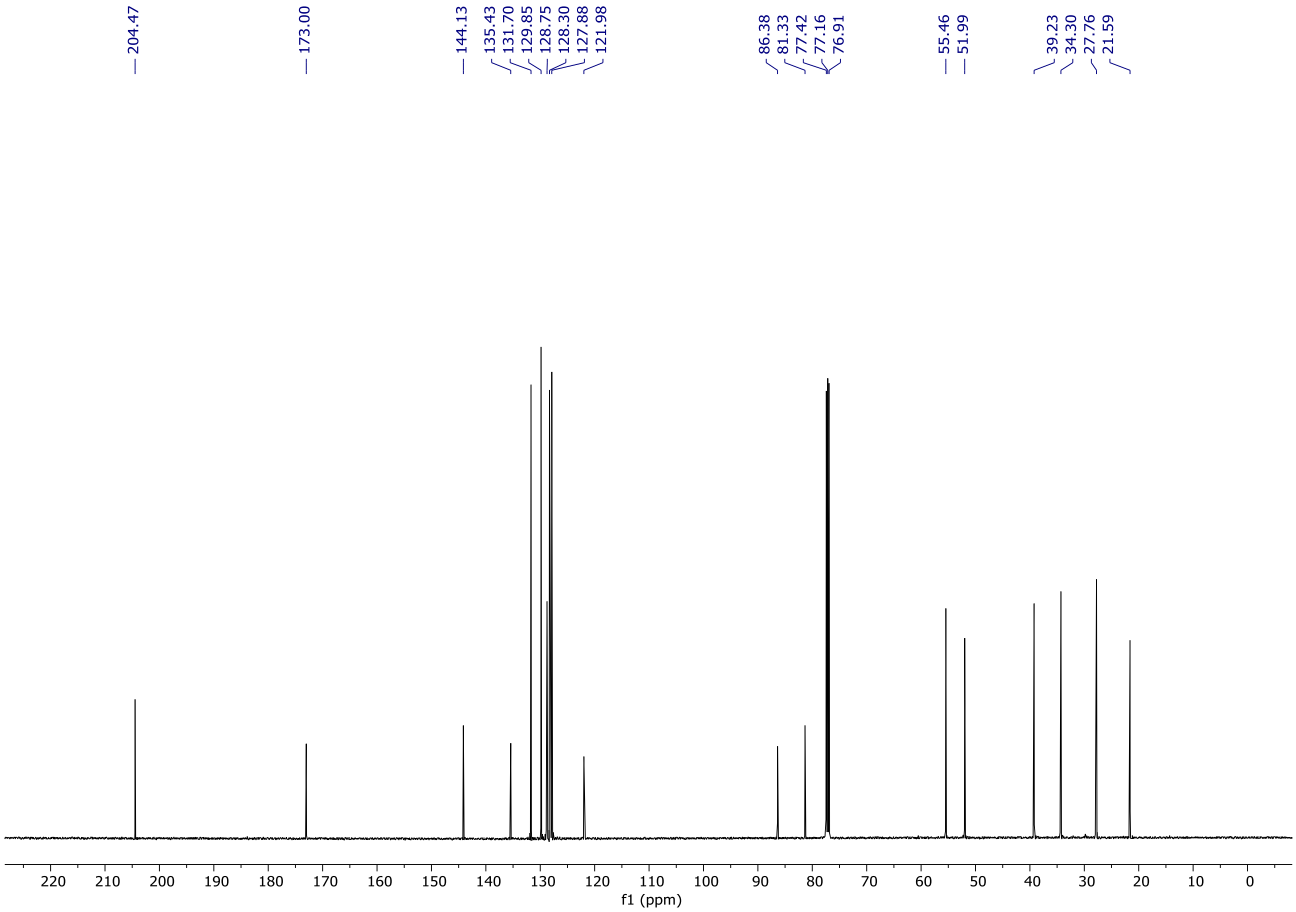
204.47
173.00
144.13
135.43
131.70
129.85
128.75
128.30
127.88
121.98
86.38
81.33
77.42
77.16
76.91
55.46
51.99
39.23
34.30
27.76
21.59
220 210 200 190 180 170 160 150 140 130 120 110 100 90 80 70 60 50 40 30 20 10 0
f1 (ppm)

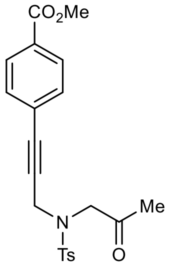
CO2Me
N
Ts
O
Me

1i

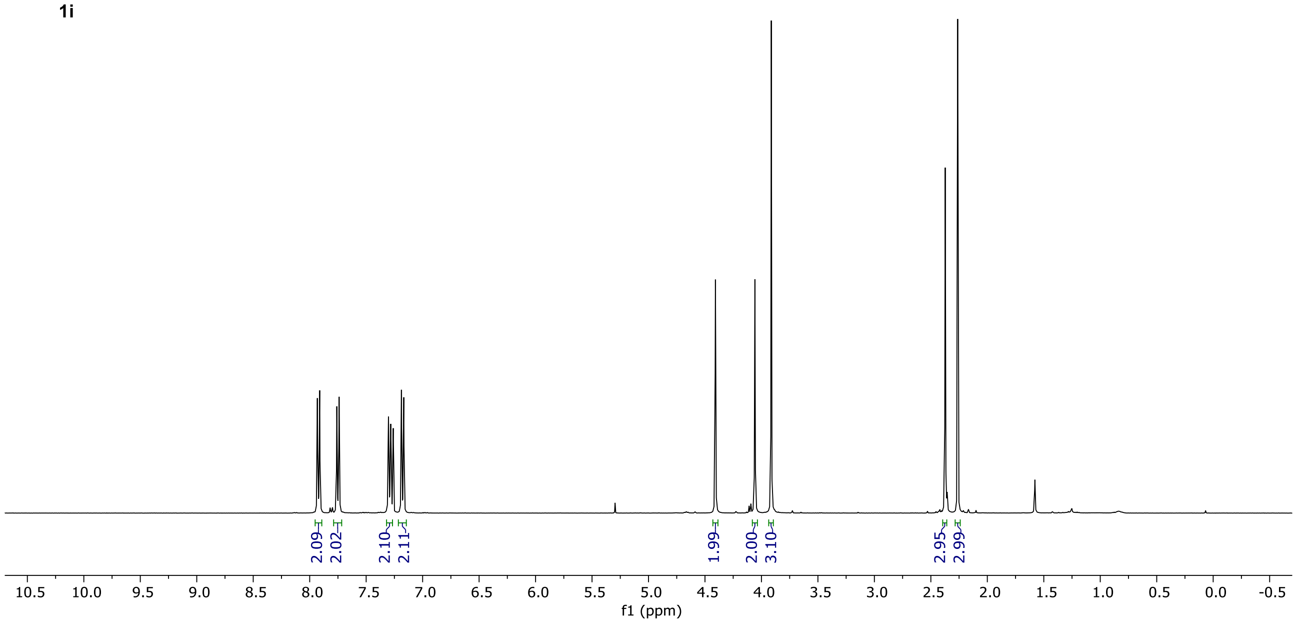
2.09
2.02
2.10
2.11
1.99
2.00
3.10
2.95
2.99
10.5 10.0 9.5 9.0 8.5 8.0 7.5 7.0 6.5 6.0 5.5 5.0 4.5 4.0 3.5 3.0 2.5 2.0 1.5 1.0 0.5 0.0 -0.5
f1 (ppm)

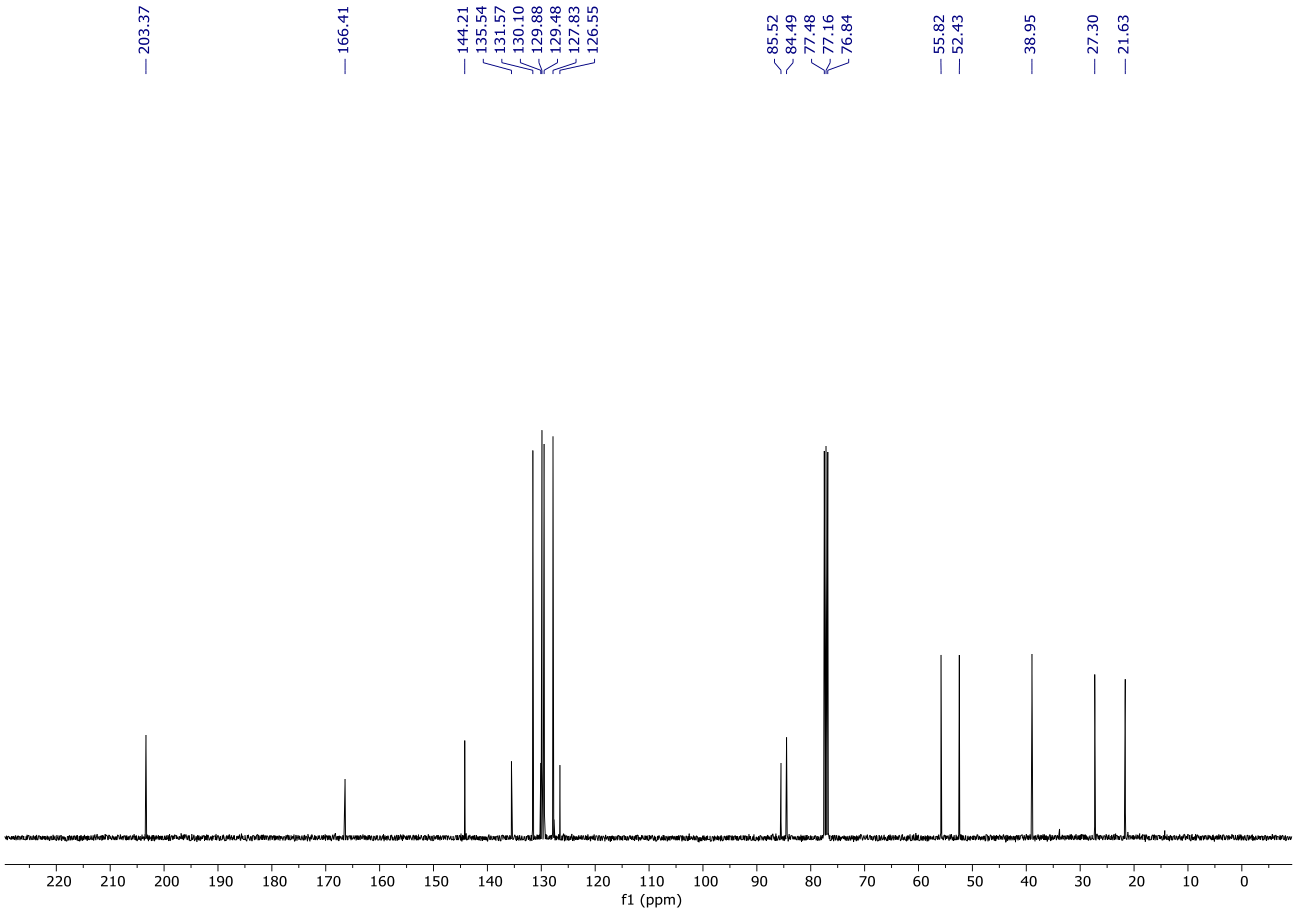
203.37
166.41
144.21
135.54
131.57
130.10
129.88
129.48
127.83
126.55
85.52
84.49
77.48
77.16
76.84
55.82
52.43
38.95
27.30
21.63
220 210 200 190 180 170 160 150 140 130 120 110 100 90 80 70 60 50 40 30 20 10 0
f1 (ppm)

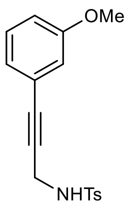
OMe
NHTs

S6

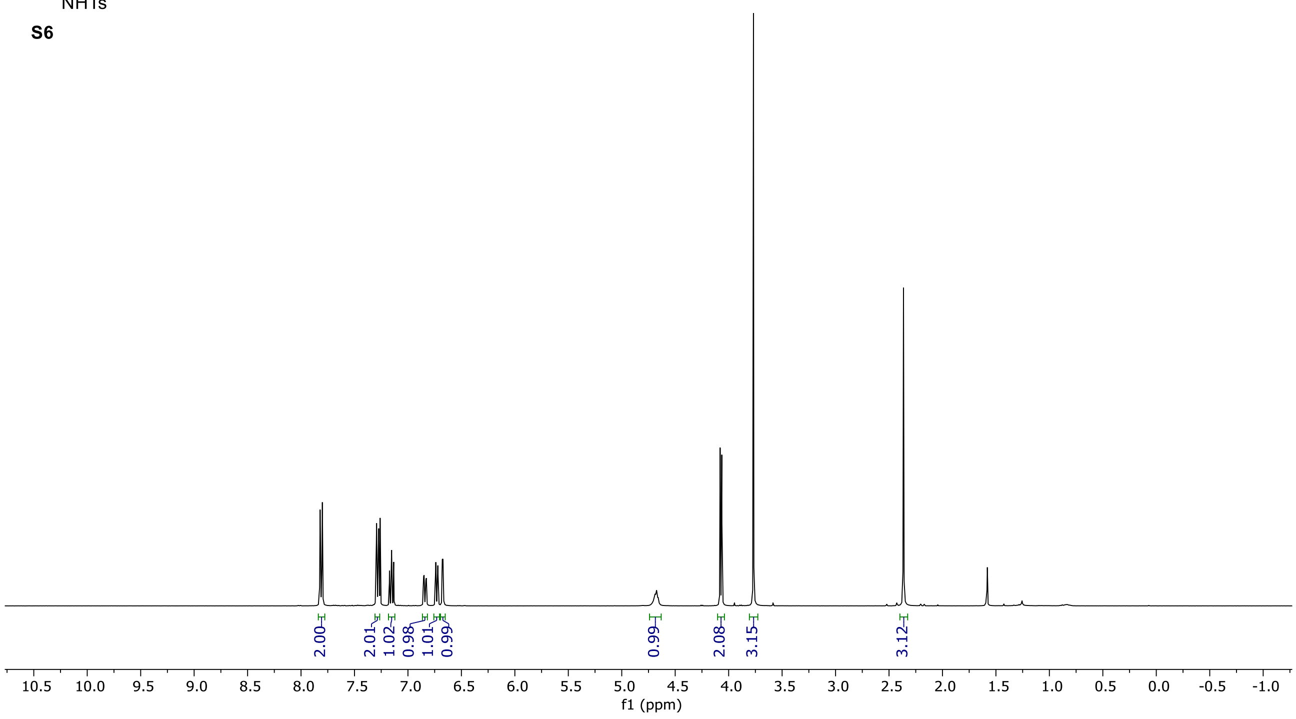
2.00
2.01
1.02
0.98
1.01
0.99
0.99
2.08
3.15
3.12
10.5
10.0
9.5
9.0
8.5
8.0
7.5
7.0
6.5
6.0
5.5
5.0
4.5
4.0
3.5
3.0
2.5
2.0
1.5
1.0
0.5
0.0
-0.5
-1.0
f1 (ppm)

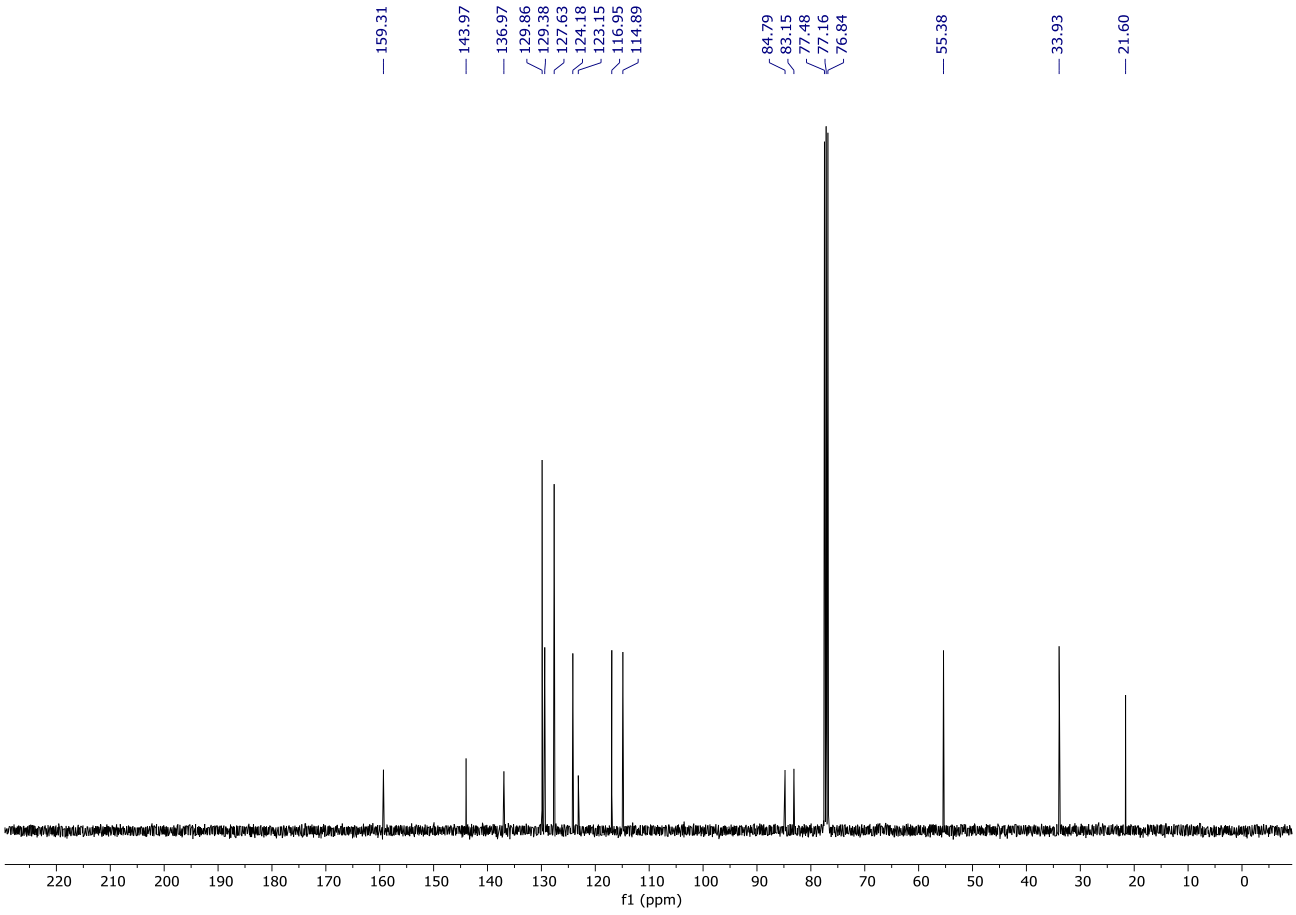
159.31
143.97
136.97
129.86
129.38
127.63
124.18
123.15
116.95
114.89
84.79
83.15
77.48
77.16
76.84
55.38
33.93
21.60
220
210
200
190
180
170
160
150
140
130
120
110
100
90
80
70
60
50
40
30
20
10
0
f1 (ppm)

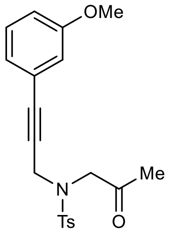
OMe
Me
N
Ts
O

1j

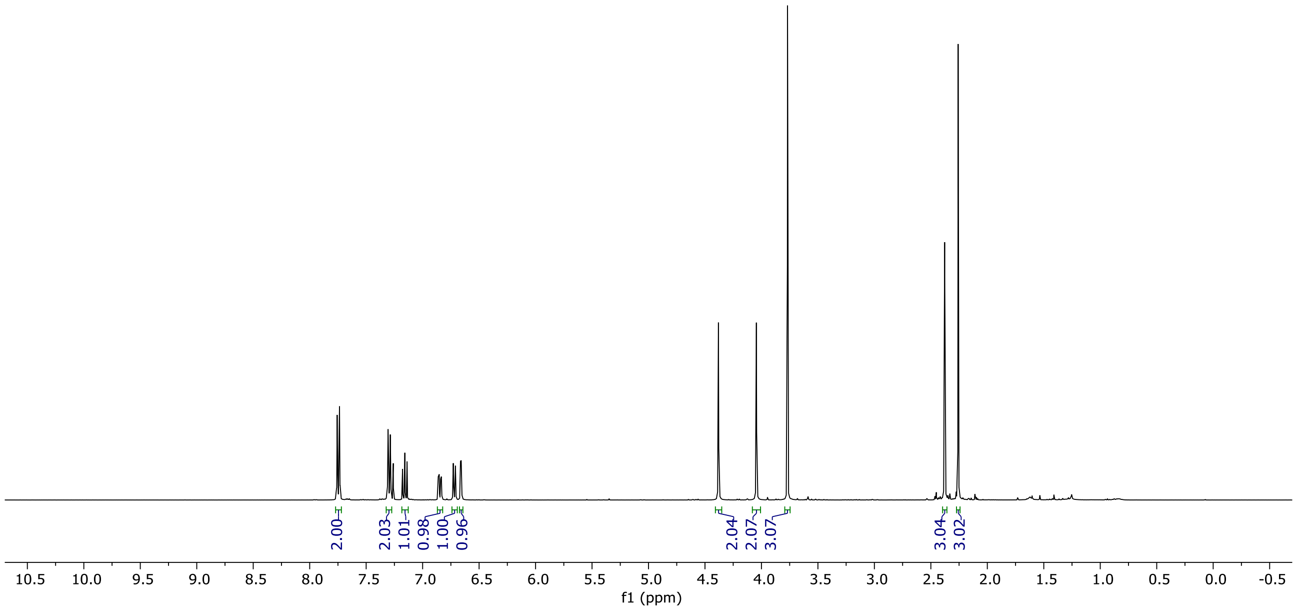
2.00
2.03
1.01
0.98
1.00
0.96
2.04
2.07
3.07
3.04
3.02
10.5
10.0
9.5
9.0
8.5
8.0
7.5
7.0
6.5
6.0
5.5
5.0
4.5
4.0
3.5
3.0
2.5
2.0
1.5
1.0
0.5
0.0
-0.5
f1 (ppm)

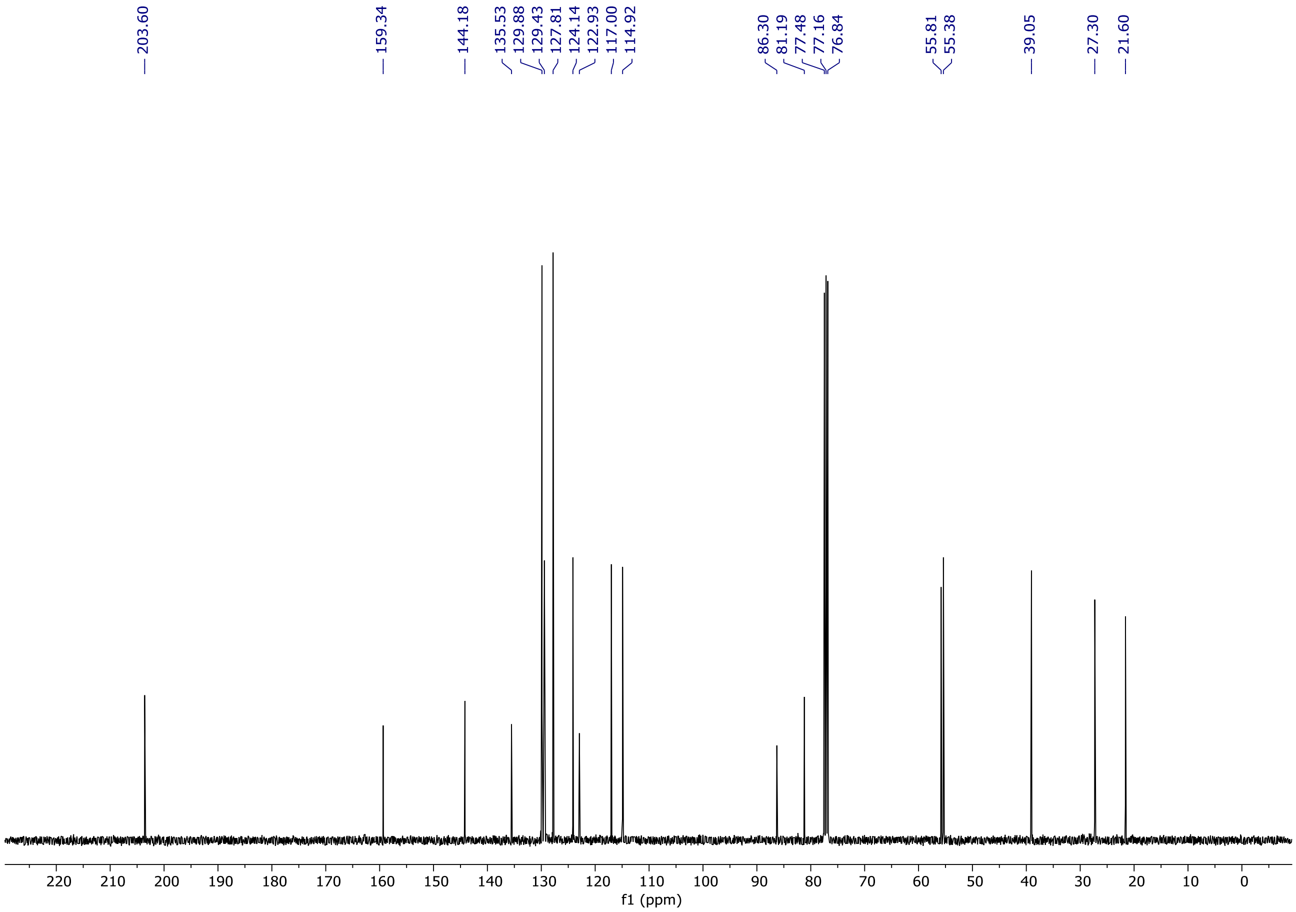
203.60
159.34
144.18
135.53
129.88
129.43
127.81
124.14
122.93
117.00
114.92
86.30
81.19
77.48
77.16
76.84
55.81
55.38
39.05
27.30
21.60
220
210
200
190
180
170
160
150
140
130
120
110
100
90
80
70
60
50
40
30
20
10
0
f1 (ppm)

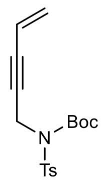
Boc
N
Ts

S8

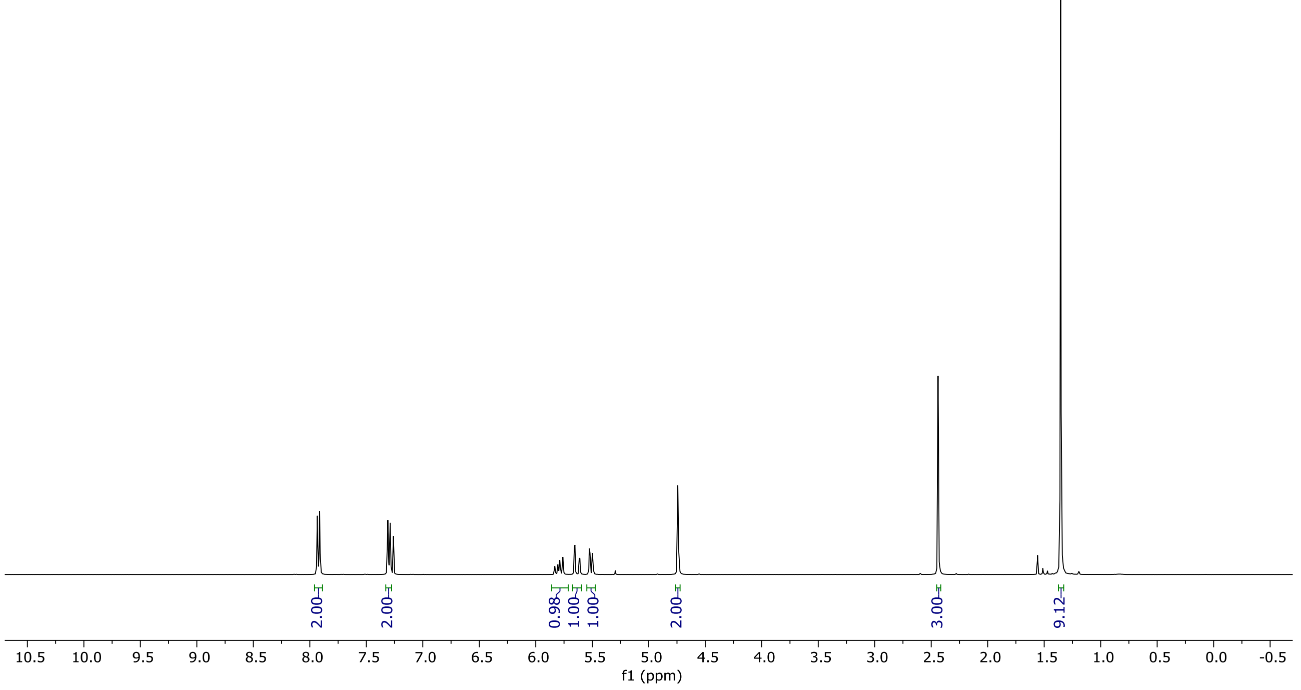
2.00
2.00
0.98
1.00
1.00
2.00
3.00
9.12
10.5 10.0 9.5 9.0 8.5 8.0 7.5 7.0 6.5 6.0 5.5 5.0 4.5 4.0 3.5 3.0 2.5 2.0 1.5 1.0 0.5 0.0 -0.5
f1 (ppm)

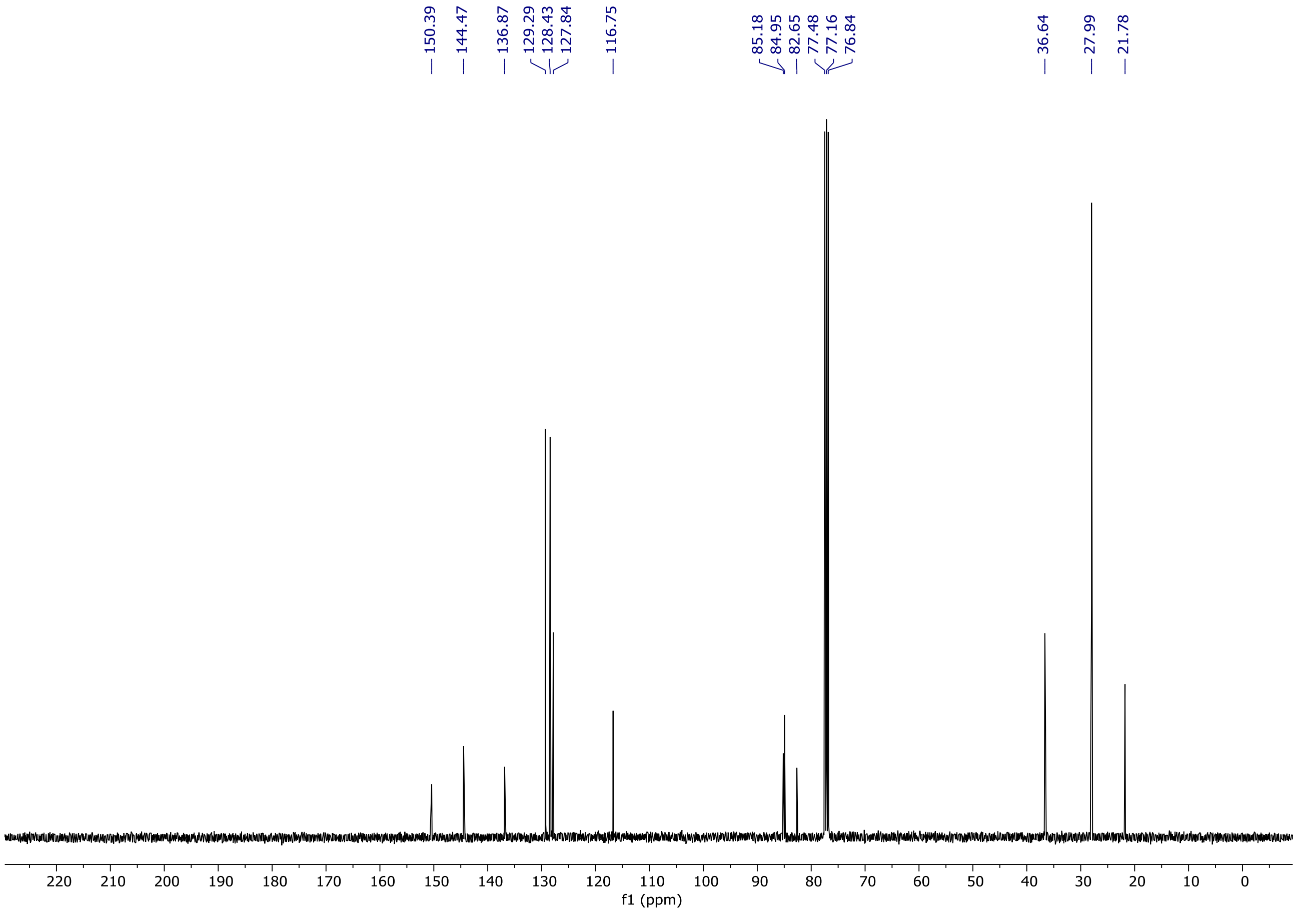
150.39
144.47
136.87
129.29
128.43
127.84
116.75
85.18
84.95
82.65
77.48
77.16
76.84
36.64
27.99
21.78
220 210 200 190 180 170 160 150 140 130 120 110 100 90 80 70 60 50 40 30 20 10 0
f1 (ppm)

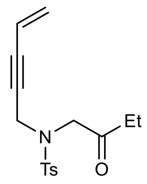

Et
N
Ts
O

1k

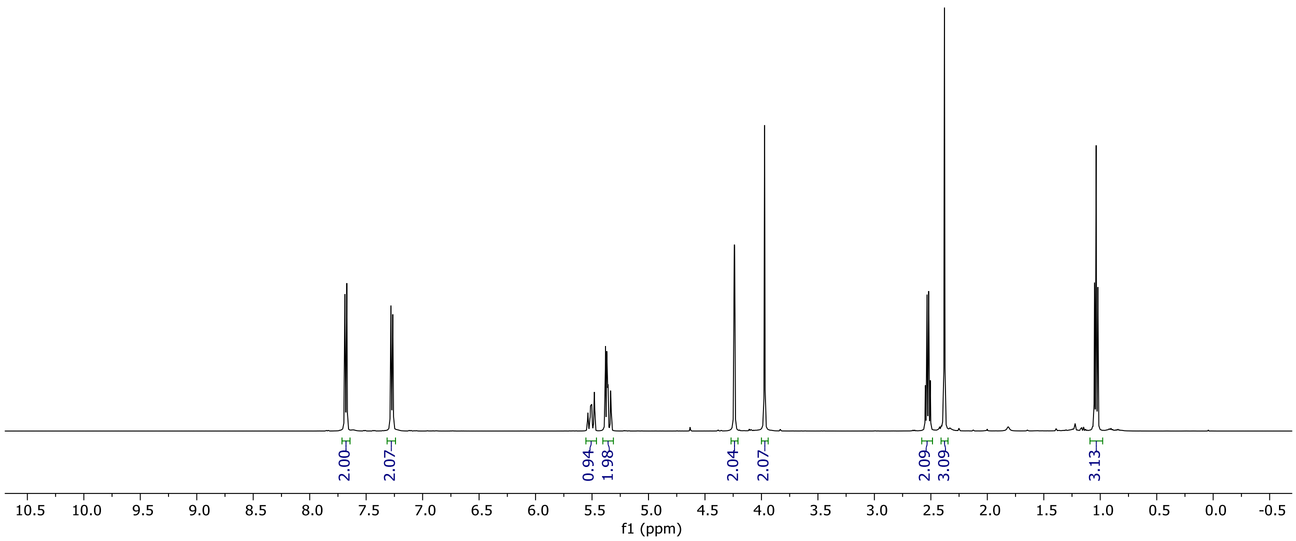

2.00
2.07
0.94
1.98
2.04
2.07
2.09
3.09
3.13
f1 (ppm)

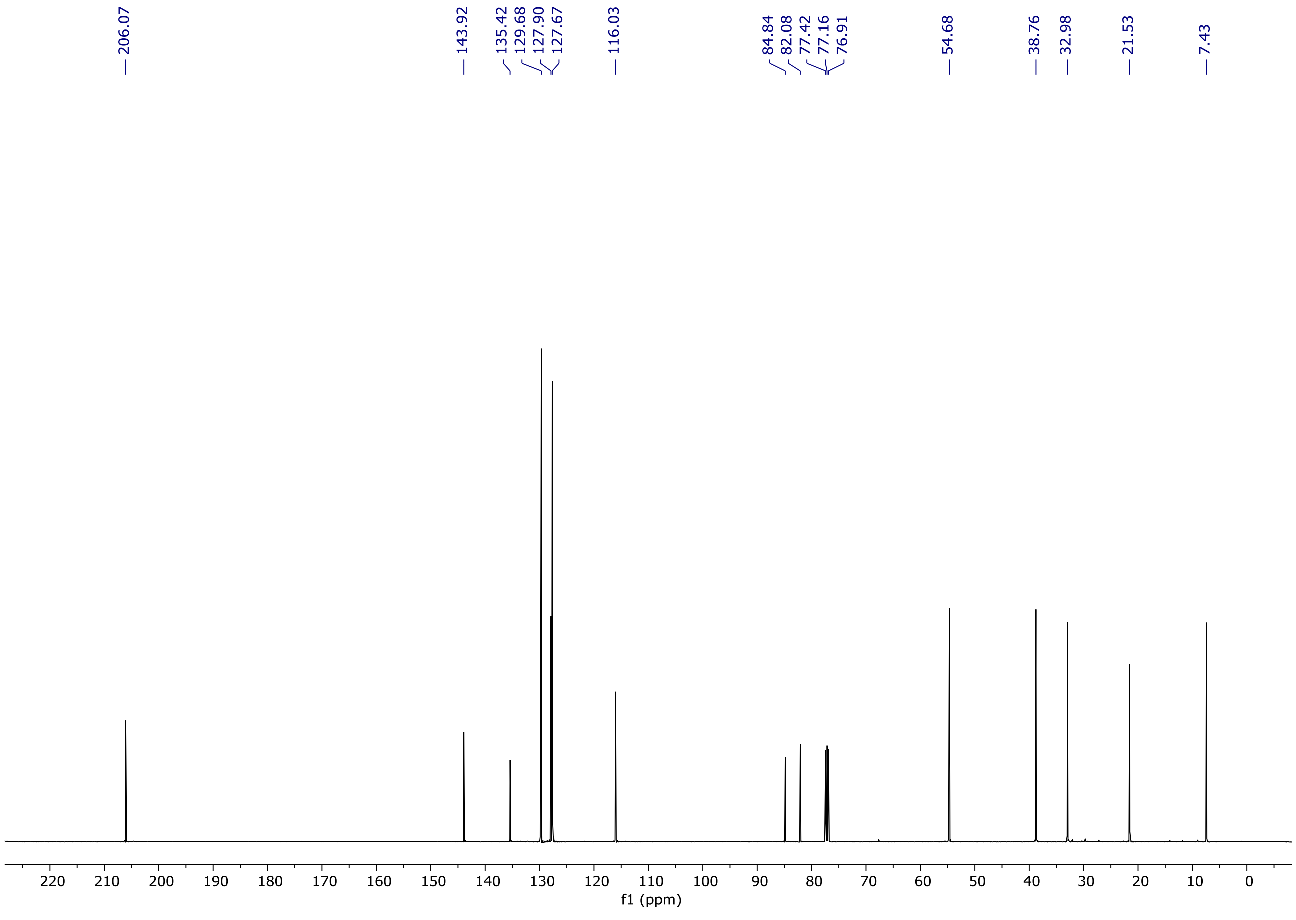

206.07
143.92
135.42
129.68
127.90
127.67
116.03
84.84
82.08
77.42
77.16
76.91
54.68
38.76
32.98
21.53
7.43
f1 (ppm)

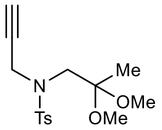
Me
N
OMe
Ts
OMe

S11

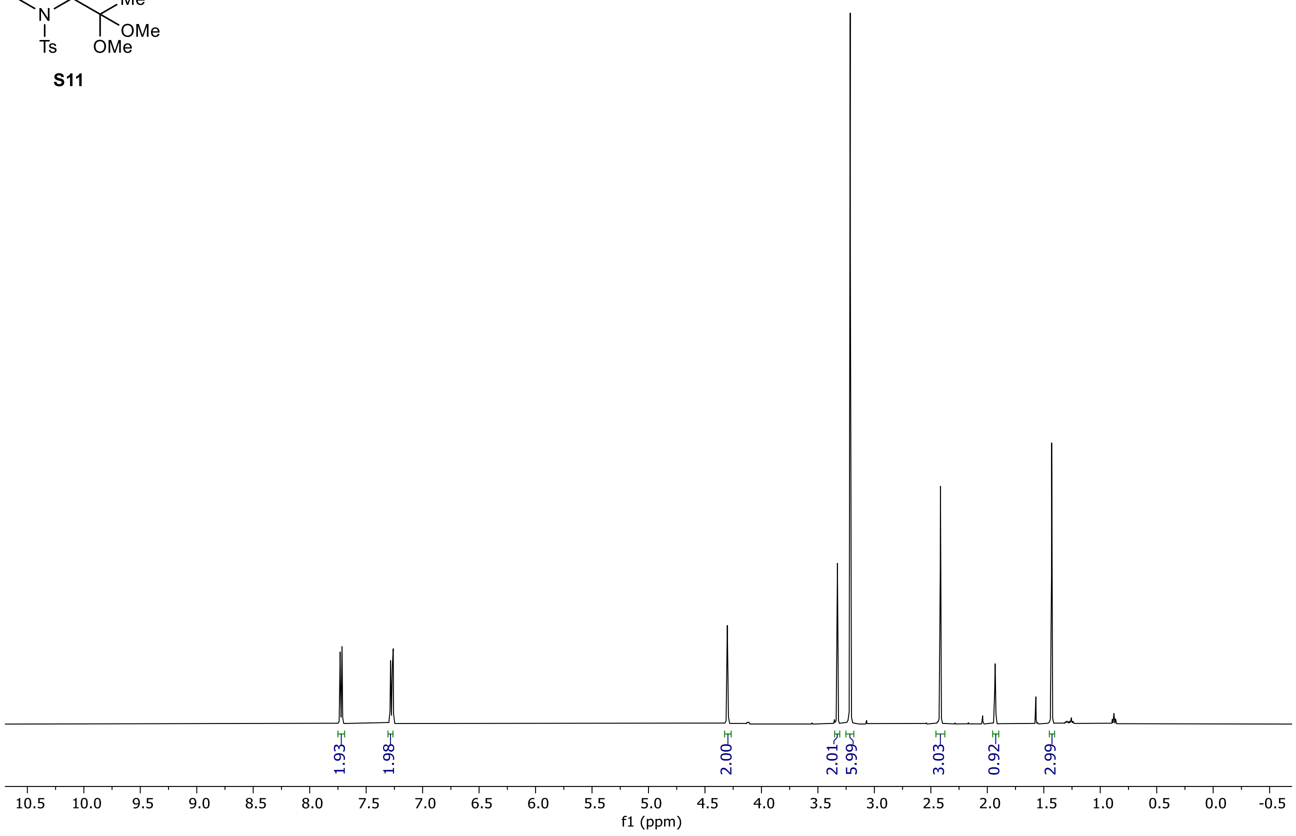
1.93
1.98
2.00
2.01
5.99
3.03
0.92
2.99
10.5 10.0 9.5 9.0 8.5 8.0 7.5 7.0 6.5 6.0 5.5 5.0 4.5 4.0 3.5 3.0 2.5 2.0 1.5 1.0 0.5 0.0 -0.5
f1 (ppm)

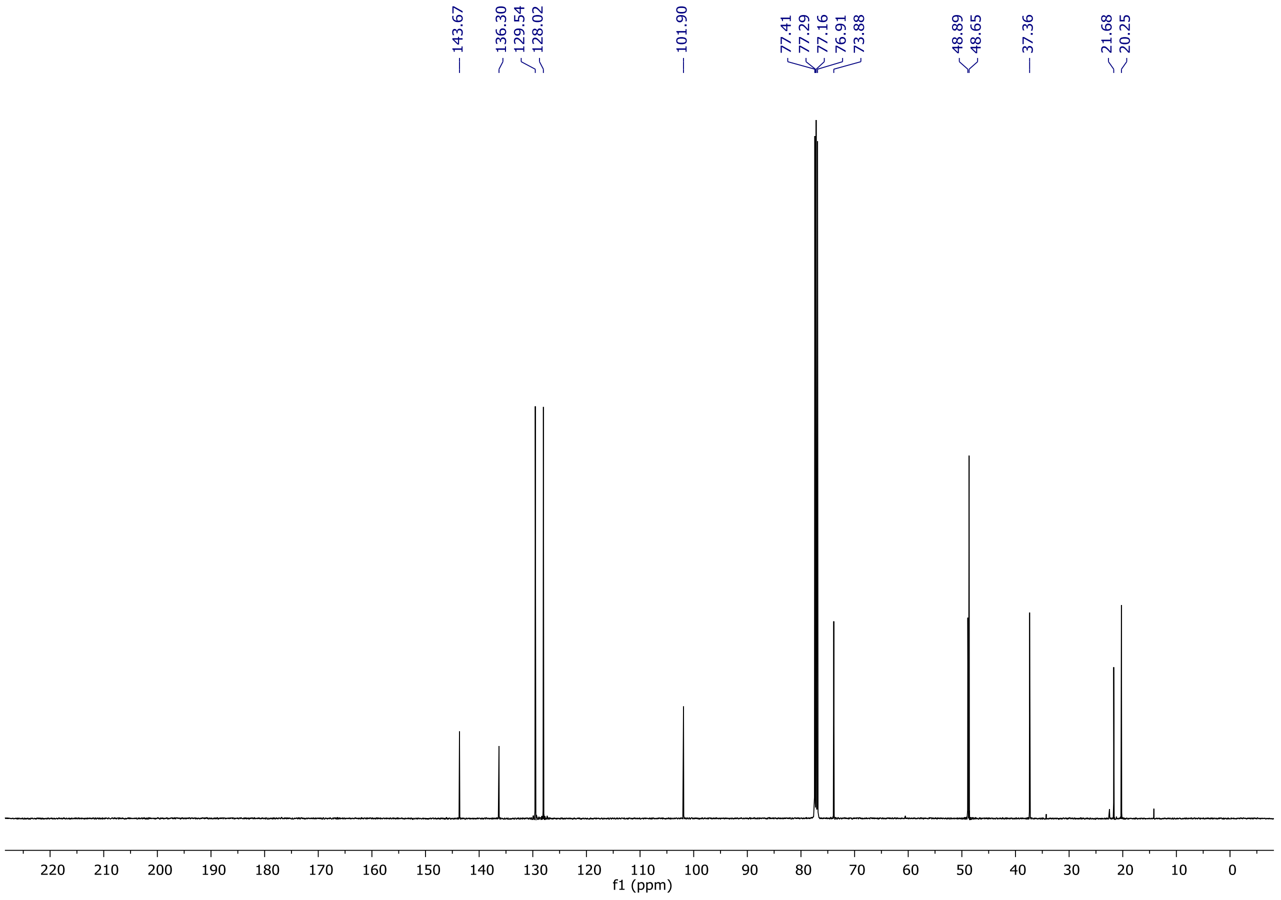
143.67
136.30
129.54
128.02
101.90
77.41
77.29
77.16
76.91
73.88
48.89
48.65
37.36
21.68
20.25
220 210 200 190 180 170 160 150 140 130 120 110 100 90 80 70 60 50 40 30 20 10 0
f1 (ppm)

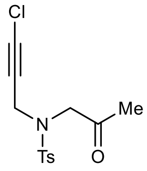
Cl
N
Ts
Me
O

1l

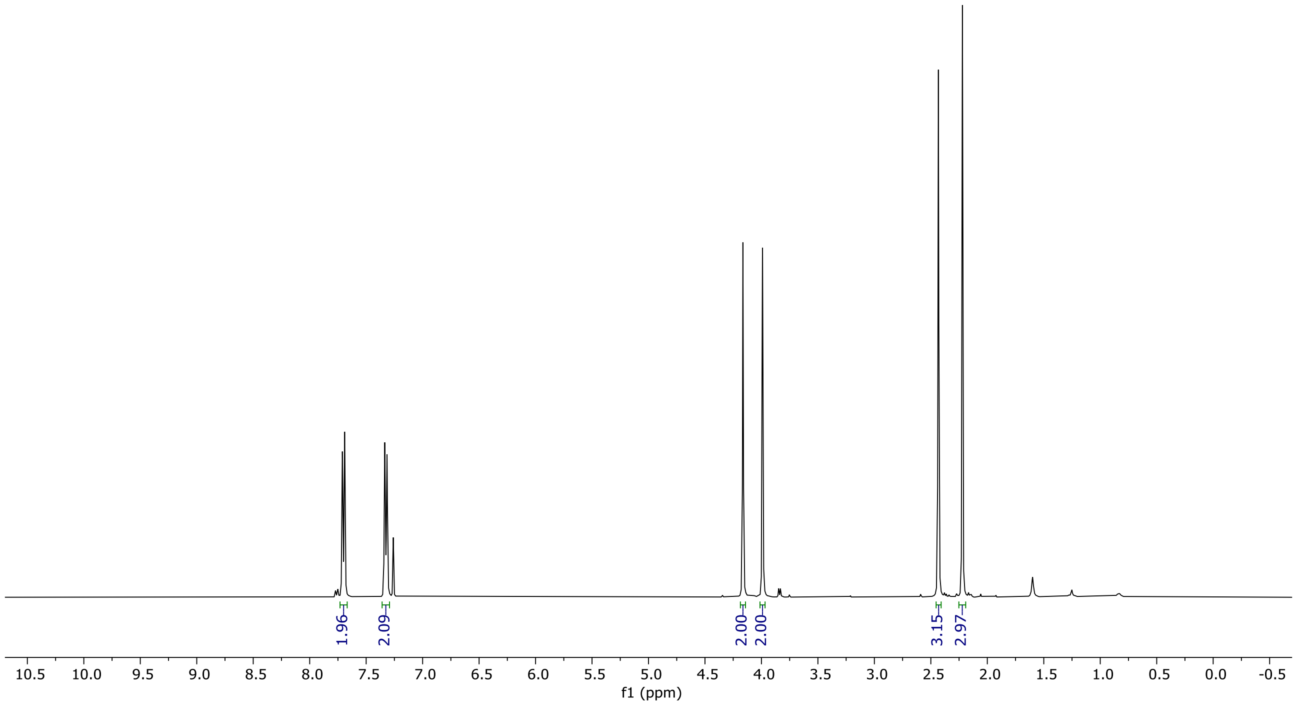
1.96
2.09
2.00
2.00
3.15
2.97
10.5 10.0 9.5 9.0 8.5 8.0 7.5 7.0 6.5 6.0 5.5 5.0 4.5 4.0 3.5 3.0 2.5 2.0 1.5 1.0 0.5 0.0 -0.5
f1 (ppm)

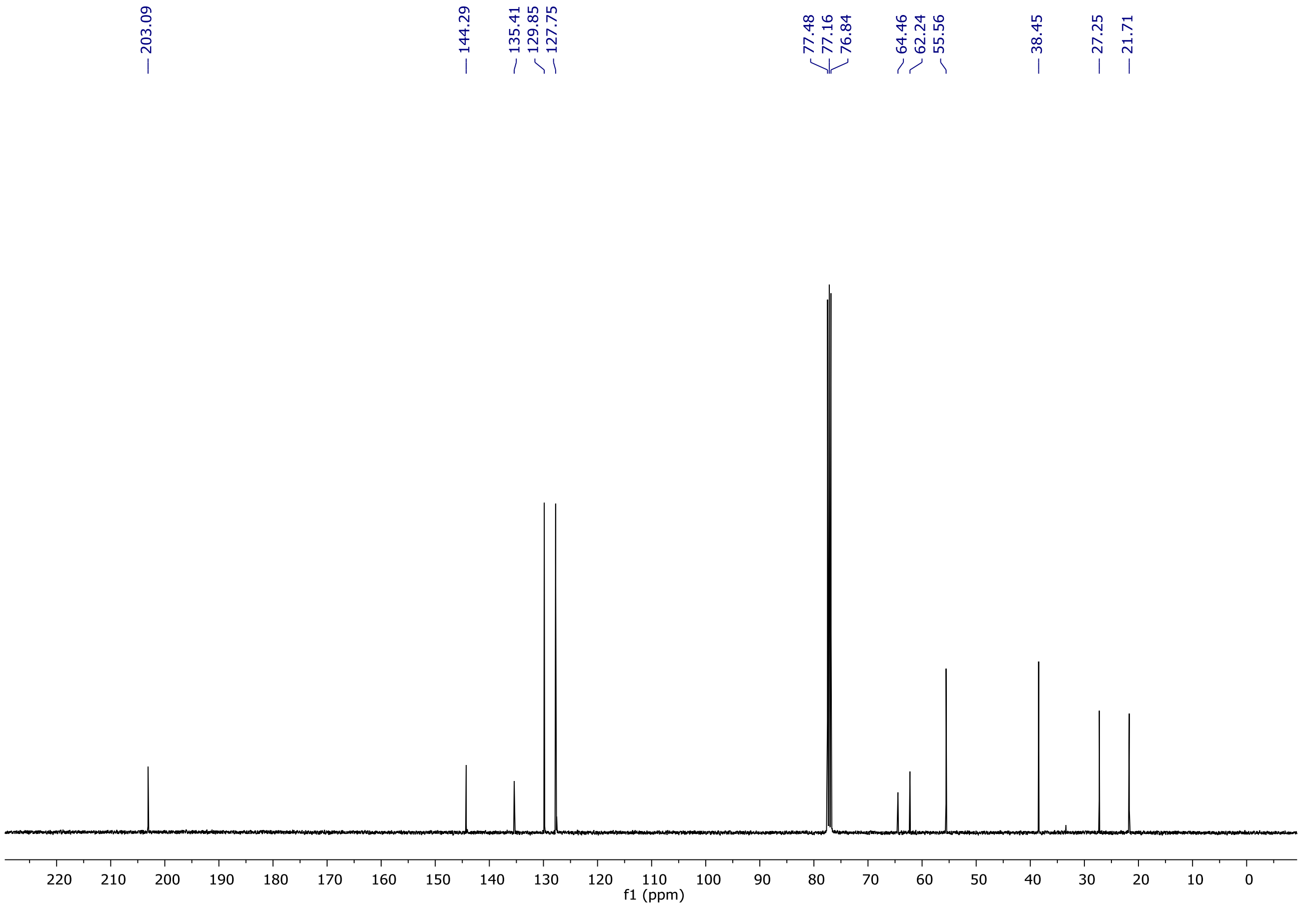
203.09
144.29
135.41
129.85
127.75
77.48
77.16
76.84
64.46
62.24
55.56
38.45
27.25
21.71
220 210 200 190 180 170 160 150 140 130 120 110 100 90 80 70 60 50 40 30 20 10 0
f1 (ppm)

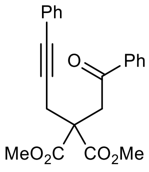
Ph
O
Ph
MeO2C
CO2Me

4

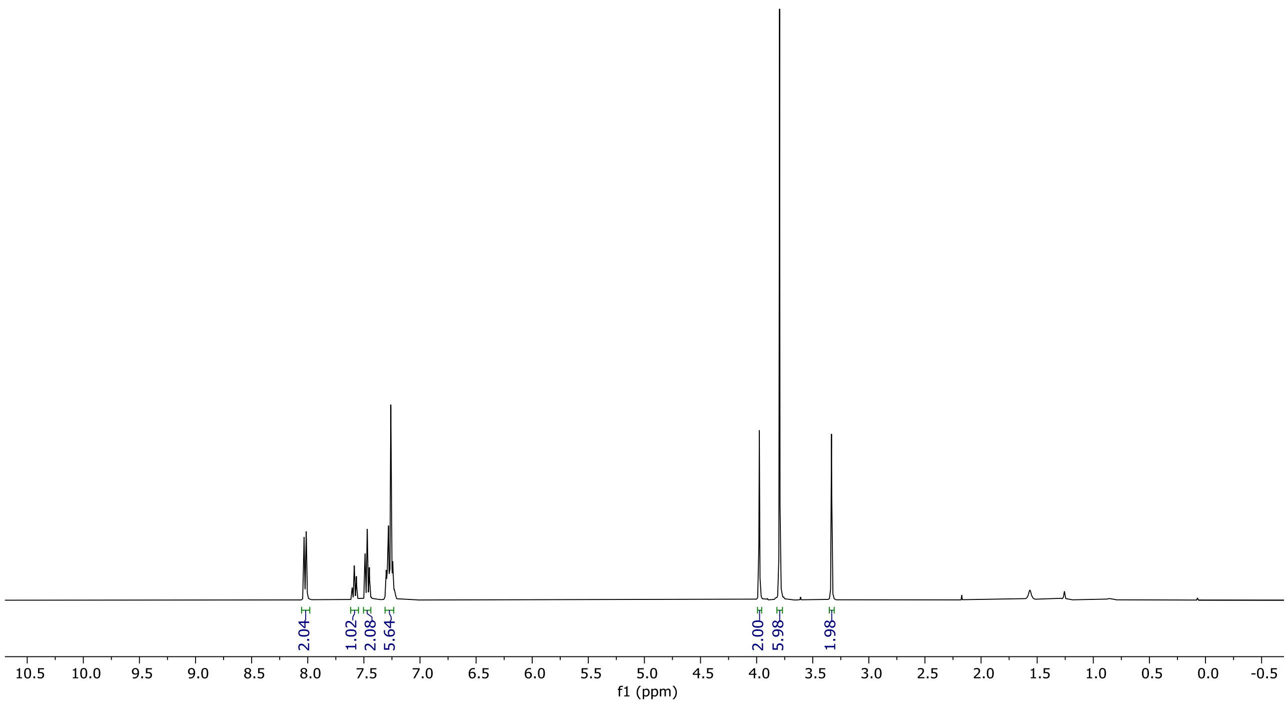
2.04
1.02
2.08
5.64
2.00
5.98
1.98
10.5
10.0
9.5
9.0
8.5
8.0
7.5
7.0
6.5
6.0
5.5
5.0
4.5
4.0
3.5
3.0
2.5
2.0
1.5
1.0
0.5
0.0
-0.5
f1 (ppm)

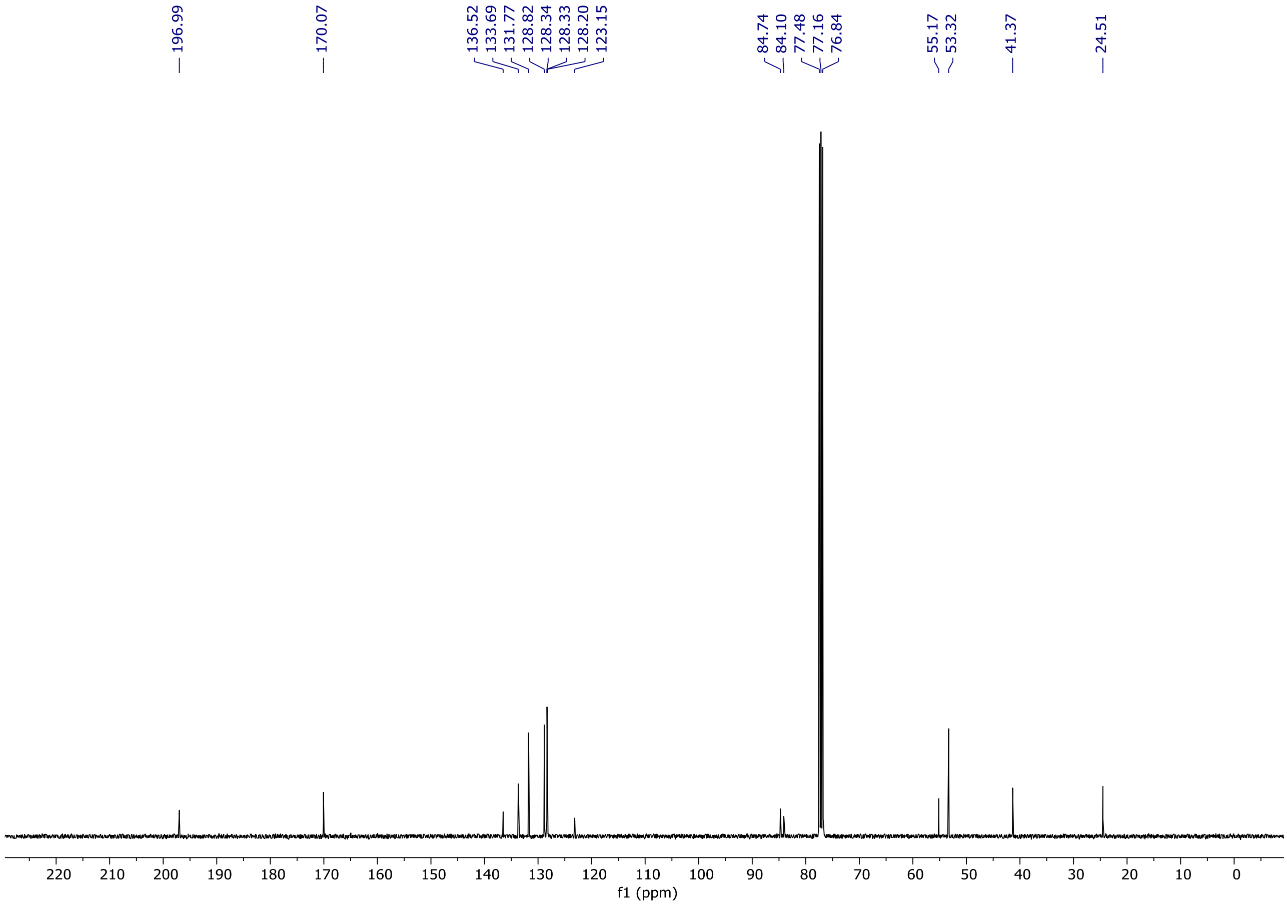
196.99
170.07
136.52
133.69
131.77
128.82
128.34
128.33
128.20
123.15
84.74
84.10
77.48
77.16
76.84
55.17
53.32
41.37
24.51
220
210
200
190
180
170
160
150
140
130
120
110
100
90
80
70
60
50
40
30
20
10
0
f1 (ppm)

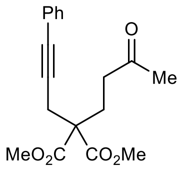
Ph
O
Me
MeO2C
CO2Me

8

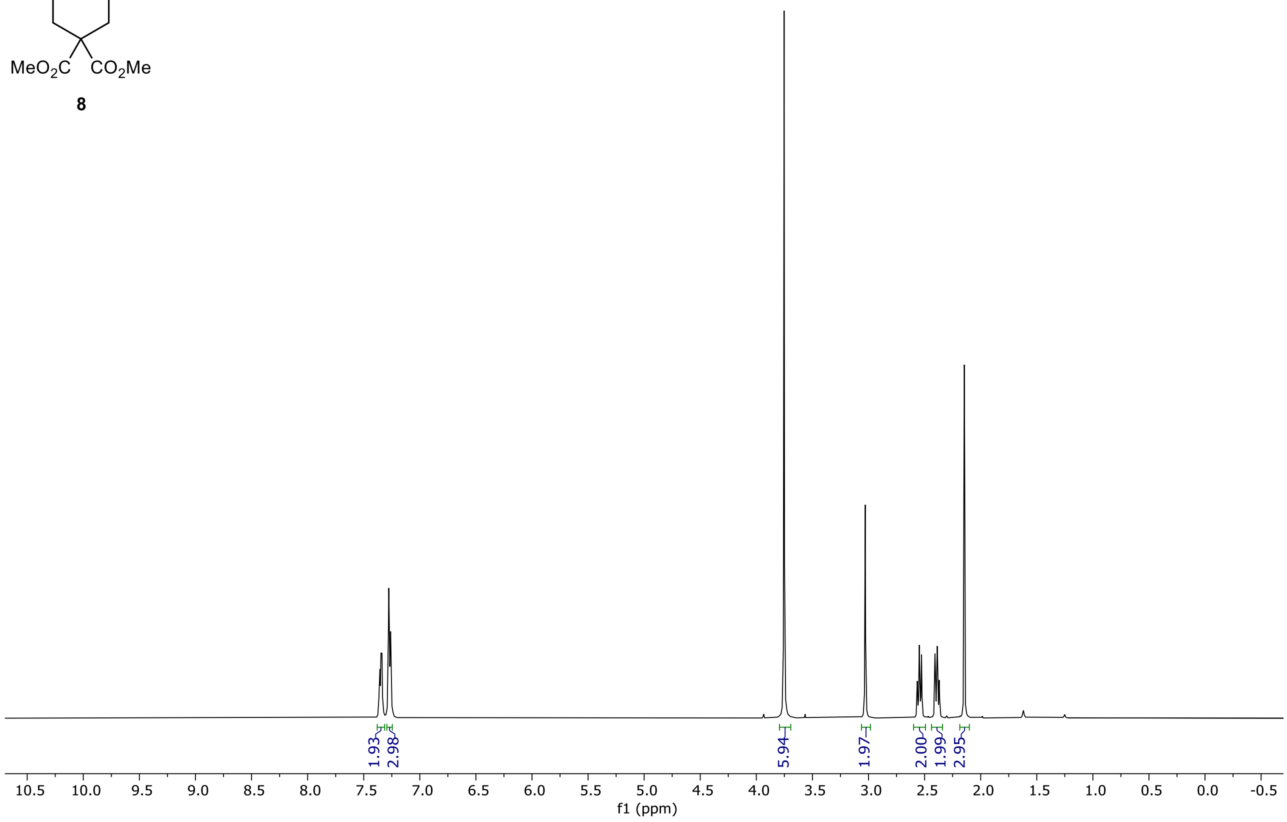
1.93
2.98
5.94
1.97
2.00
1.99
2.95
10.5 10.0 9.5 9.0 8.5 8.0 7.5 7.0 6.5 6.0 5.5 5.0 4.5 4.0 3.5 3.0 2.5 2.0 1.5 1.0 0.5 0.0 -0.5
f1 (ppm)

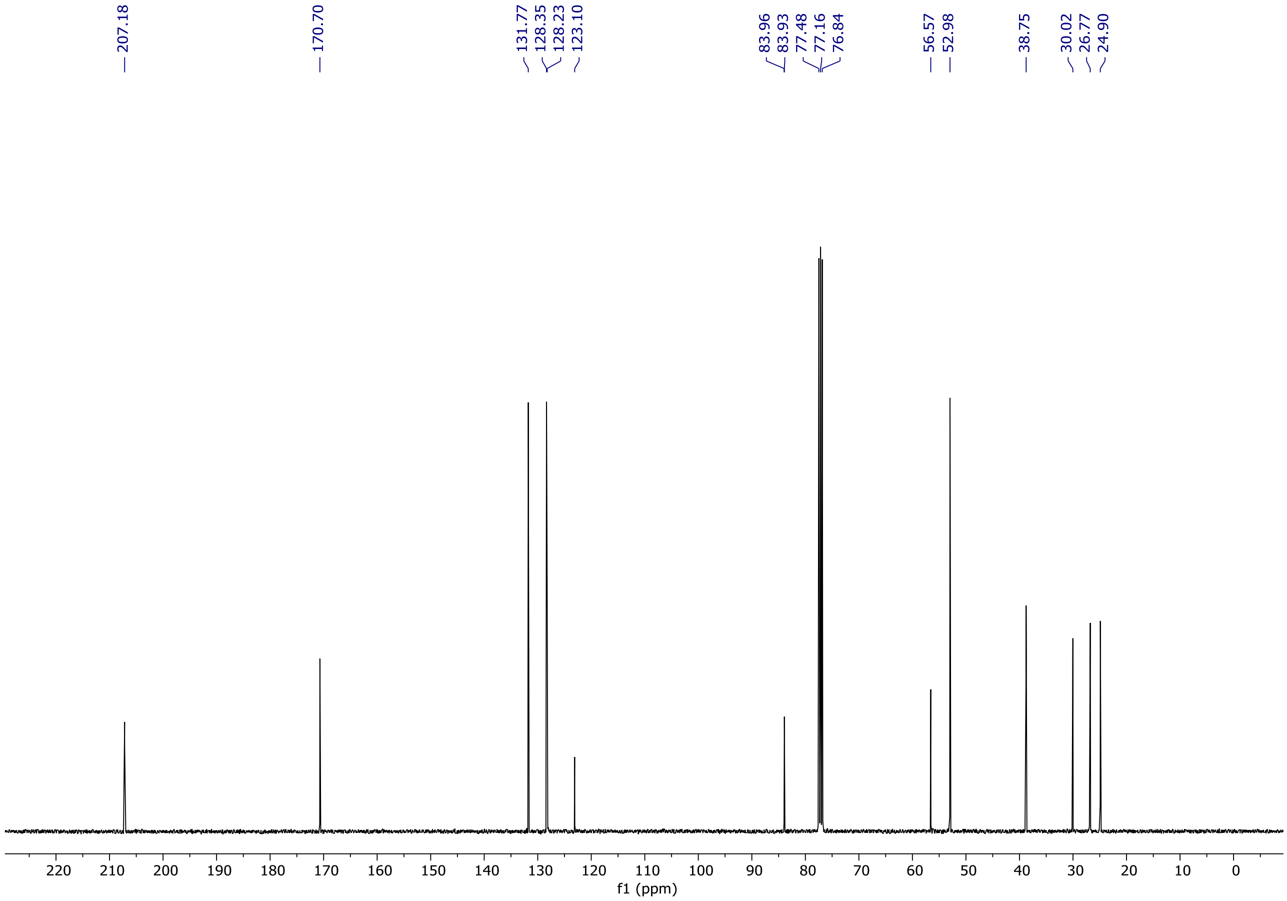
207.18
170.70
131.77
128.35
128.23
123.10
83.96
83.93
77.48
77.16
76.84
56.57
52.98
38.75
30.02
26.77
24.90
220 210 200 190 180 170 160 150 140 130 120 110 100 90 80 70 60 50 40 30 20 10 0
f1 (ppm)

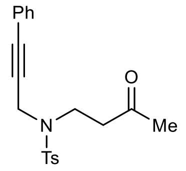
Ph
O
N
Me
Ts

9

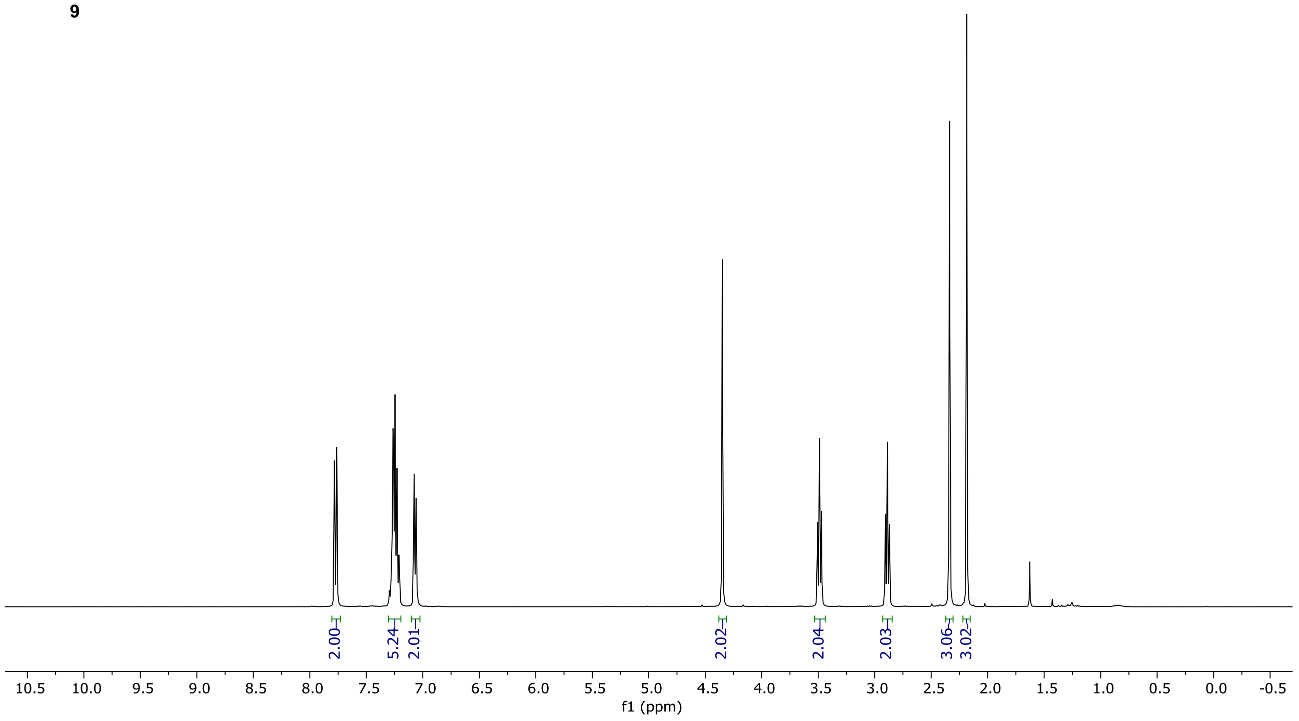
2.00
5.24
2.01
2.02
2.04
2.03
3.06
3.02
10.5 10.0 9.5 9.0 8.5 8.0 7.5 7.0 6.5 6.0 5.5 5.0 4.5 4.0 3.5 3.0 2.5 2.0 1.5 1.0 0.5 0.0 -0.5
f1 (ppm)

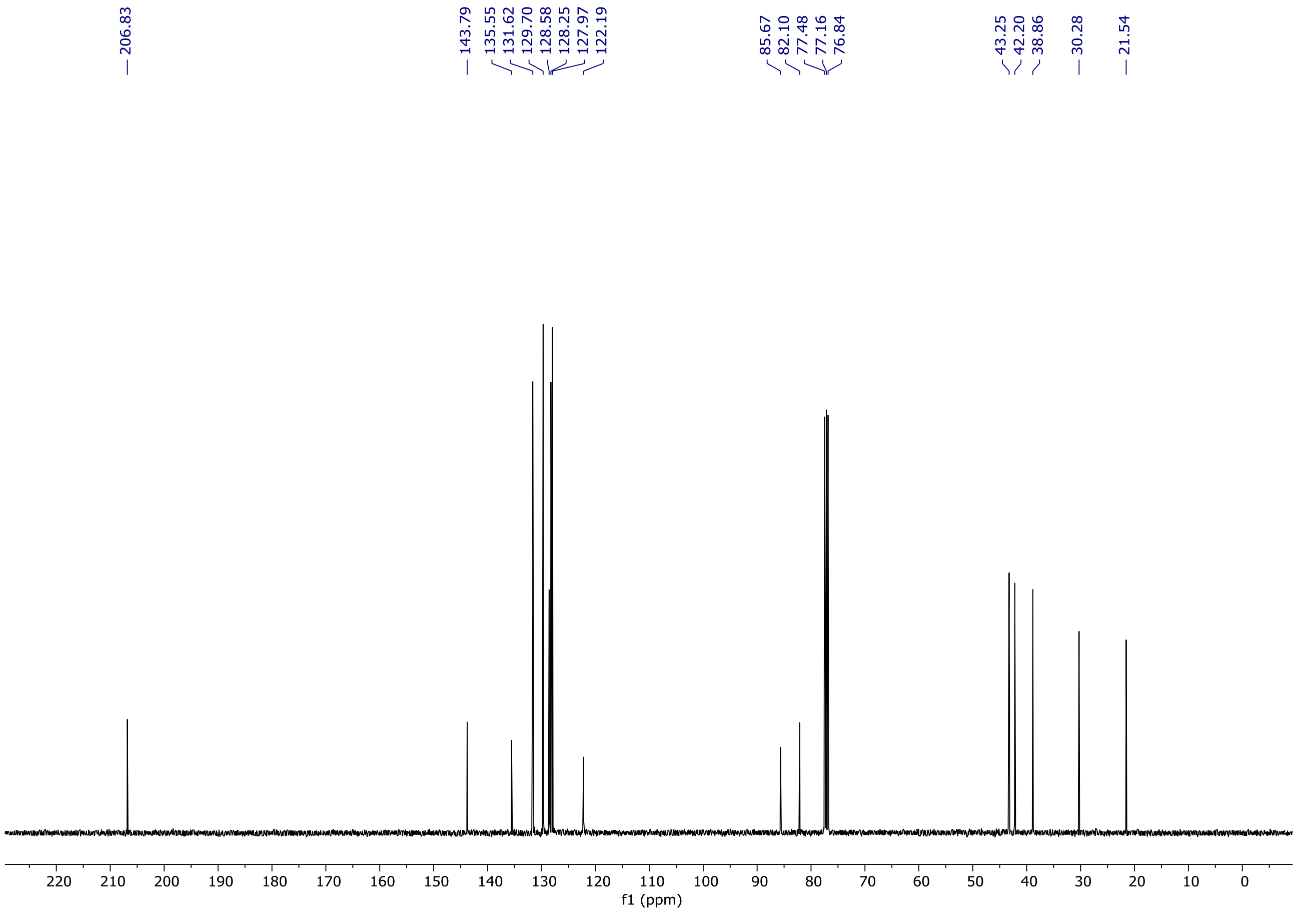
206.83
143.79
135.55
131.62
129.70
128.58
128.25
127.97
122.19
85.67
82.10
77.48
77.16
76.84
43.25
42.20
38.86
30.28
21.54
220 210 200 190 180 170 160 150 140 130 120 110 100 90 80 70 60 50 40 30 20 10 0
f1 (ppm)

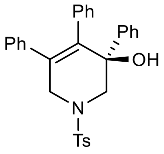
Ph
Ph
Ph
OH
N
Ts

2a

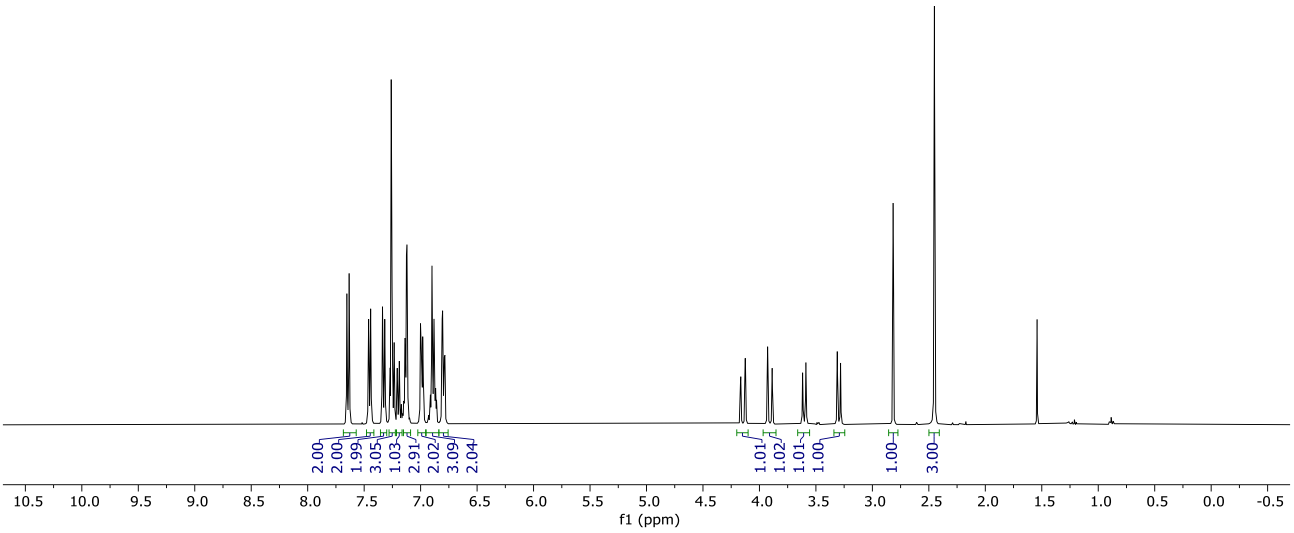
2.00
2.00
1.99
3.05
1.03
2.91
2.02
3.09
2.04
1.01
1.02
1.01
1.00
1.00
3.00
10.5 10.0 9.5 9.0 8.5 8.0 7.5 7.0 6.5 6.0 5.5 5.0 4.5 4.0 3.5 3.0 2.5 2.0 1.5 1.0 0.5 0.0 -0.5
f1 (ppm)

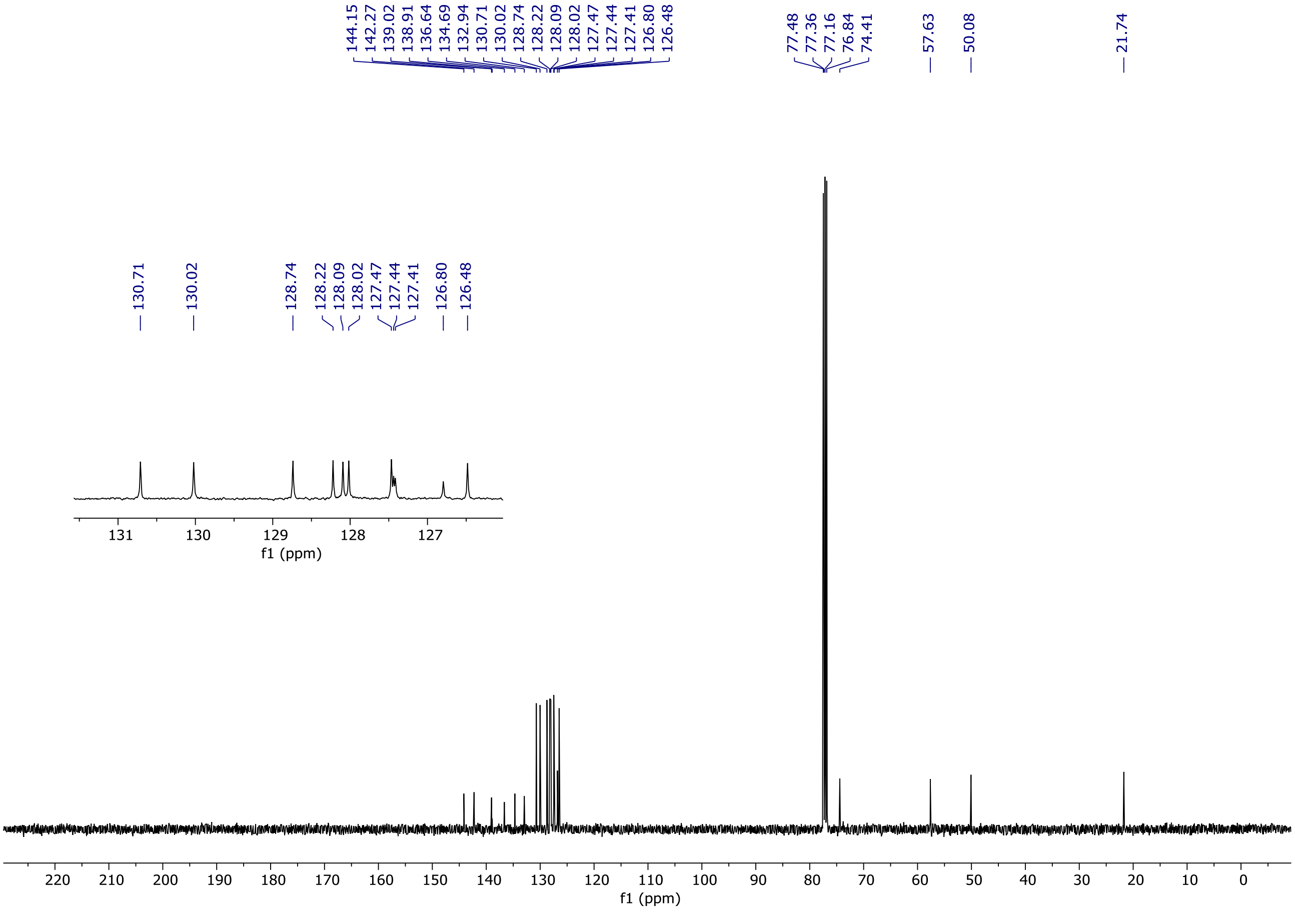
144.15
142.27
139.02
138.91
136.64
134.69
132.94
130.71
130.02
128.74
128.22
128.09
128.02
127.47
127.44
127.41
126.80
126.48
77.48
77.36
77.16
76.84
74.41
57.63
50.08
21.74
130.71
130.02
128.74
128.22
128.09
128.02
127.47
127.44
127.41
126.80
126.48
131 130 129 128 127
f1 (ppm)
220 210 200 190 180 170 160 150 140 130 120 110 100 90 80 70 60 50 40 30 20 10 0
f1 (ppm)

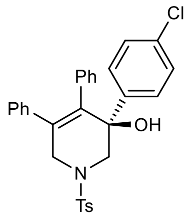
Cl
Ph
Ph
OH
N
Ts

2b

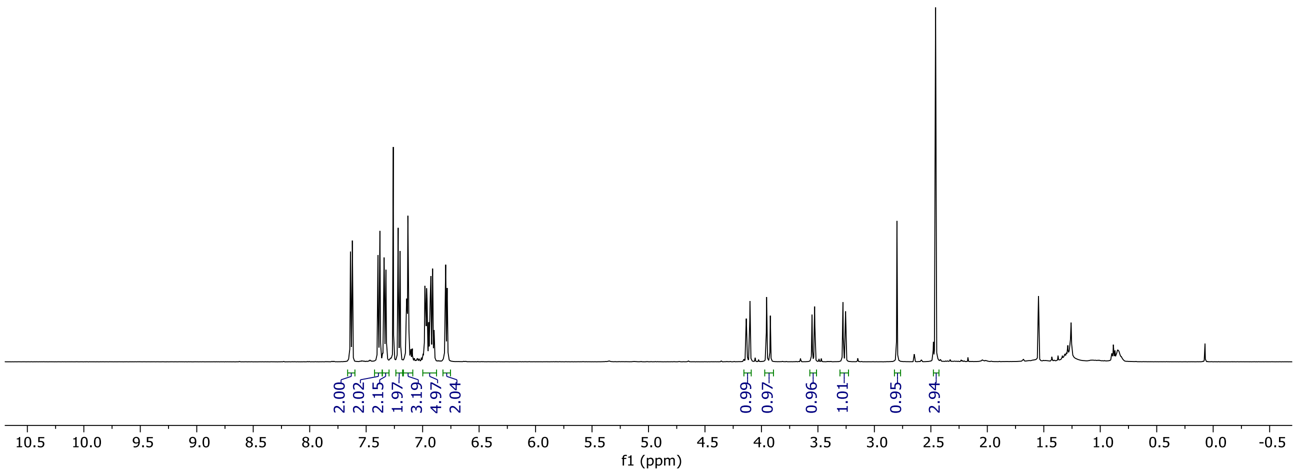
2.00
2.02
2.15
1.97
3.19
4.97
2.04
0.99
0.97
0.96
1.01
0.95
2.94
10.5 10.0 9.5 9.0 8.5 8.0 7.5 7.0 6.5 6.0 5.5 5.0 4.5 4.0 3.5 3.0 2.5 2.0 1.5 1.0 0.5 0.0 -0.5
f1 (ppm)

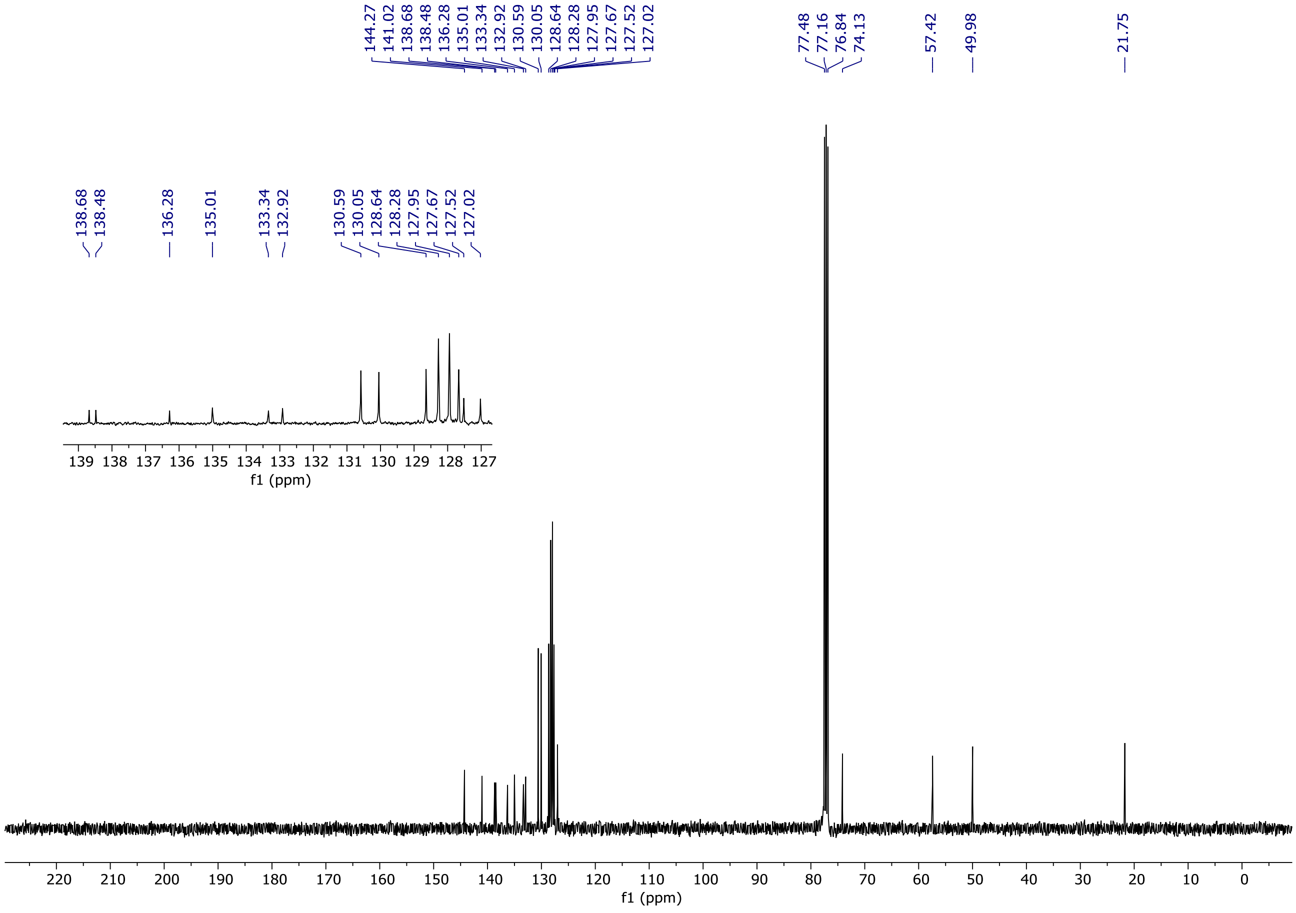
144.27
141.02
138.68
138.48
136.28
135.01
133.34
132.92
130.59
130.05
128.64
128.28
127.95
127.67
127.52
127.02
77.48
77.16
76.84
74.13
57.42
49.98
21.75
138.68
138.48
136.28
135.01
133.34
132.92
130.59
130.05
128.64
128.28
127.95
127.67
127.52
127.02
139 138 137 136 135 134 133 132 131 130 129 128 127
f1 (ppm)
220 210 200 190 180 170 160 150 140 130 120 110 100 90 80 70 60 50 40 30 20 10 0
f1 (ppm)

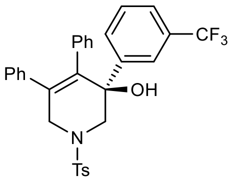

Ph
CF3
Ph
OH
N
Ts

2c

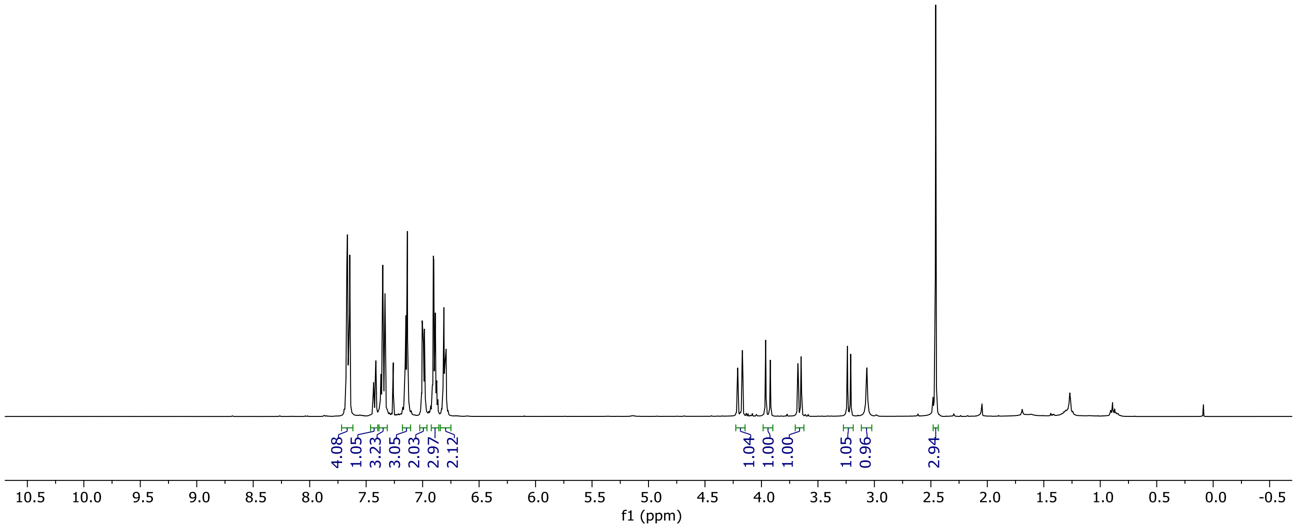

4.08
1.05
3.23
3.05
2.03
2.97
2.12
1.04
1.00
1.00
1.05
0.96
2.94
10.5 10.0 9.5 9.0 8.5 8.0 7.5 7.0 6.5 6.0 5.5 5.0 4.5 4.0 3.5 3.0 2.5 2.0 1.5 1.0 0.5 0.0 -0.5
f1 (ppm)

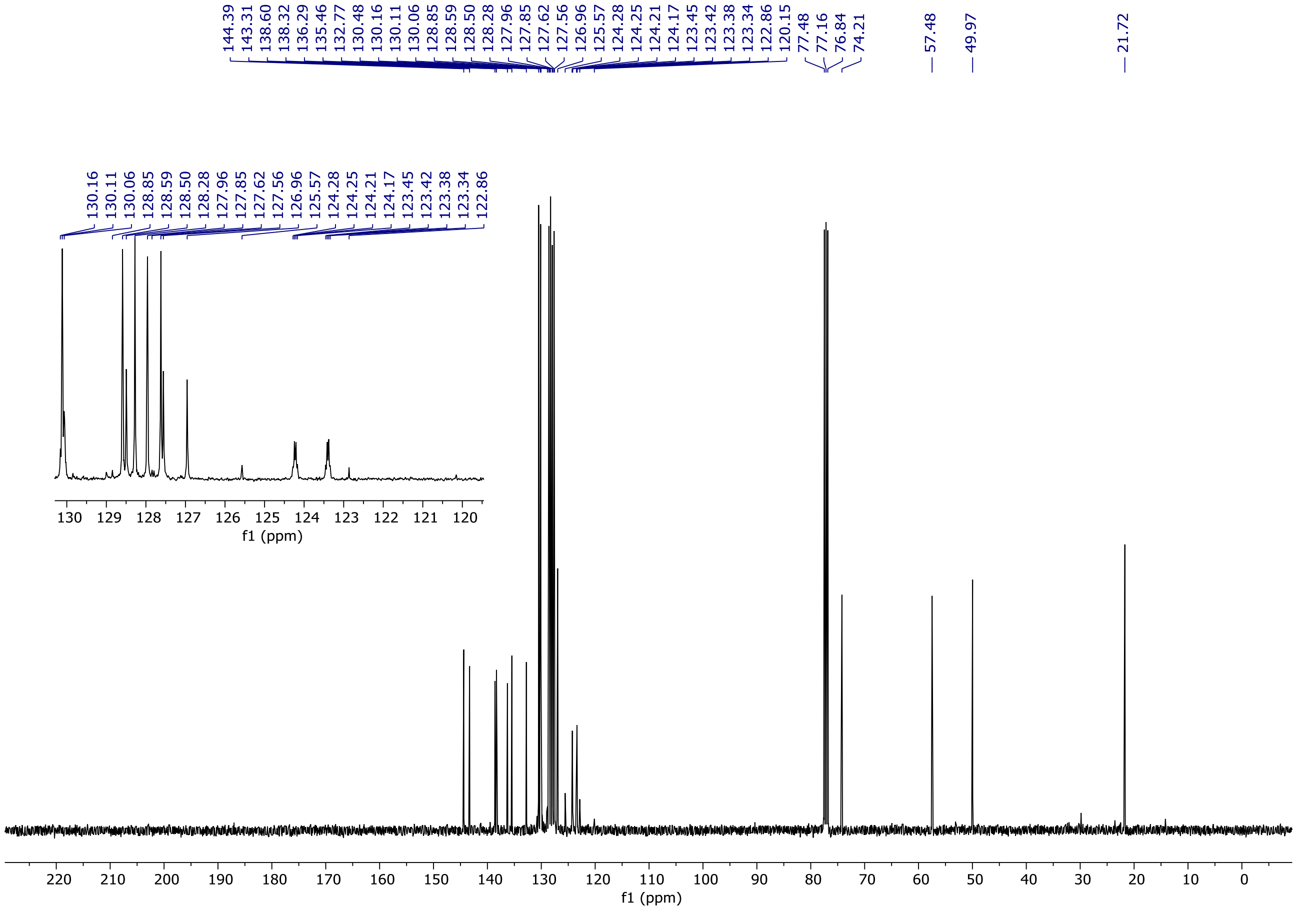

144.39
143.31
138.60
138.32
136.29
135.46
132.77
130.48
130.16
130.11
130.06
128.85
128.59
128.50
128.28
127.96
127.85
127.62
127.56
126.96
125.57
124.28
124.25
124.21
124.17
123.45
123.42
123.38
123.34
122.86
120.15
77.48
77.16
76.84
74.21
57.48
49.97
21.72
130.16
130.11
130.06
128.85
128.59
128.50
128.28
127.96
127.85
127.62
127.56
126.96
125.57
124.28
124.25
124.21
124.17
123.45
123.42
123.38
123.34
122.86
130 129 128 127 126 125 124 123 122 121 120
f1 (ppm)
220 210 200 190 180 170 160 150 140 130 120 110 100 90 80 70 60 50 40 30 20 10 0
f1 (ppm)

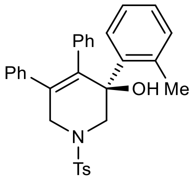
Ph
Ph
OH Me
N
Ts

2d

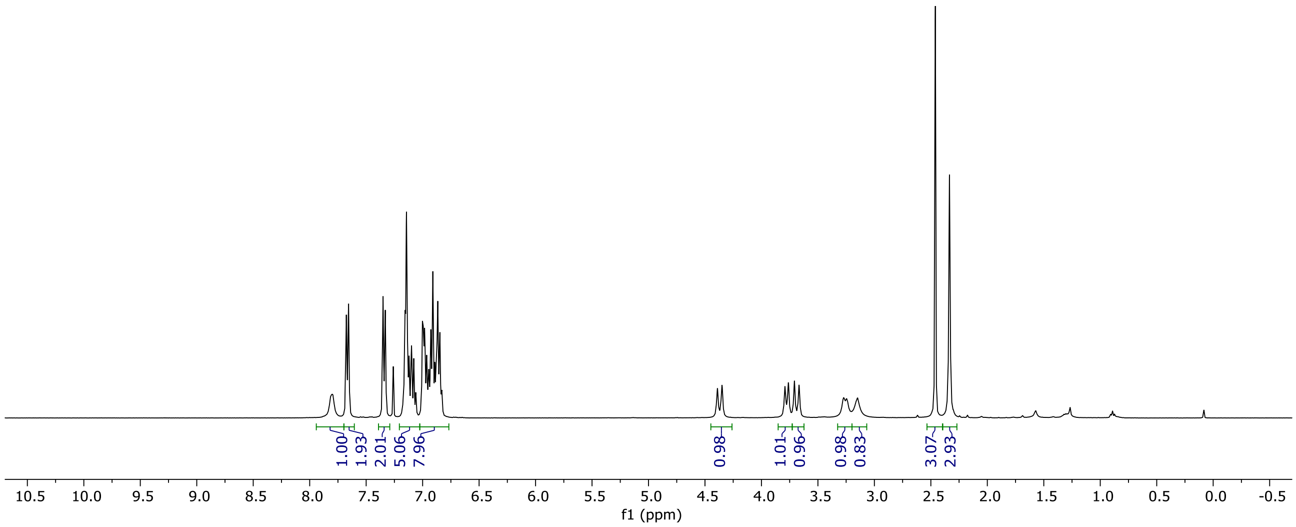
1.00
1.93
2.01
5.06
7.96
0.98
1.01
0.96
0.98
0.83
3.07
2.93
10.5 10.0 9.5 9.0 8.5 8.0 7.5 7.0 6.5 6.0 5.5 5.0 4.5 4.0 3.5 3.0 2.5 2.0 1.5 1.0 0.5 0.0 -0.5
f1 (ppm)

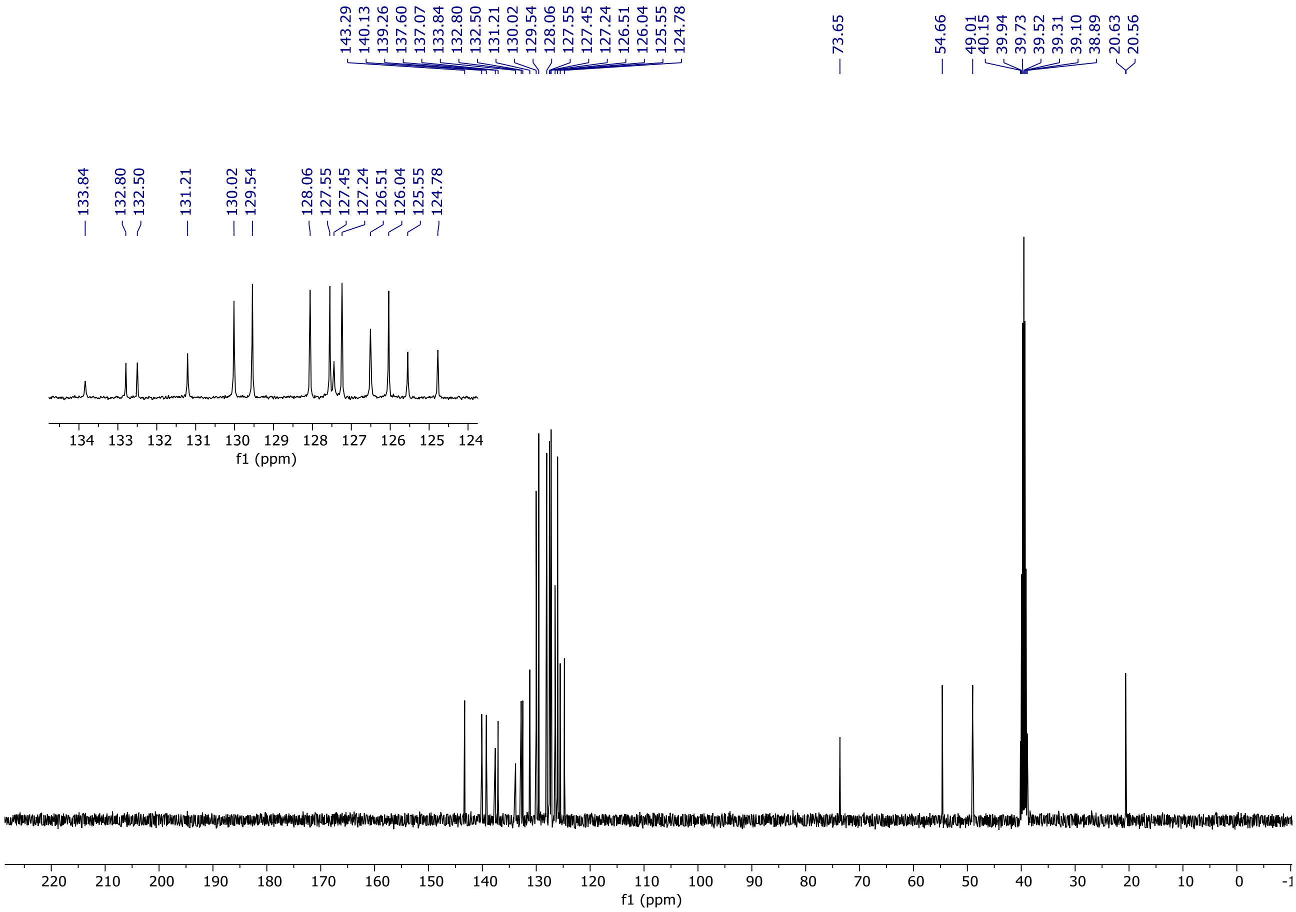
143.29
140.13
139.26
137.60
137.07
133.84
132.80
132.50
131.21
130.02
129.54
128.06
127.55
127.45
127.24
126.51
126.04
125.55
124.78
73.65
54.66
49.01
40.15
39.94
39.73
39.52
39.31
39.10
38.89
20.63
20.56
133.84
132.80
132.50
131.21
130.02
129.54
128.06
127.55
127.45
127.24
126.51
126.04
125.55
124.78
134 133 132 131 130 129 128 127 126 125 124
f1 (ppm)
220 210 200 190 180 170 160 150 140 130 120 110 100 90 80 70 60 50 40 30 20 10 0
f1 (ppm)

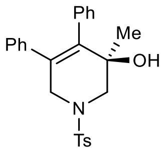
Ph
Me
Ph
OH
N
Ts

2e

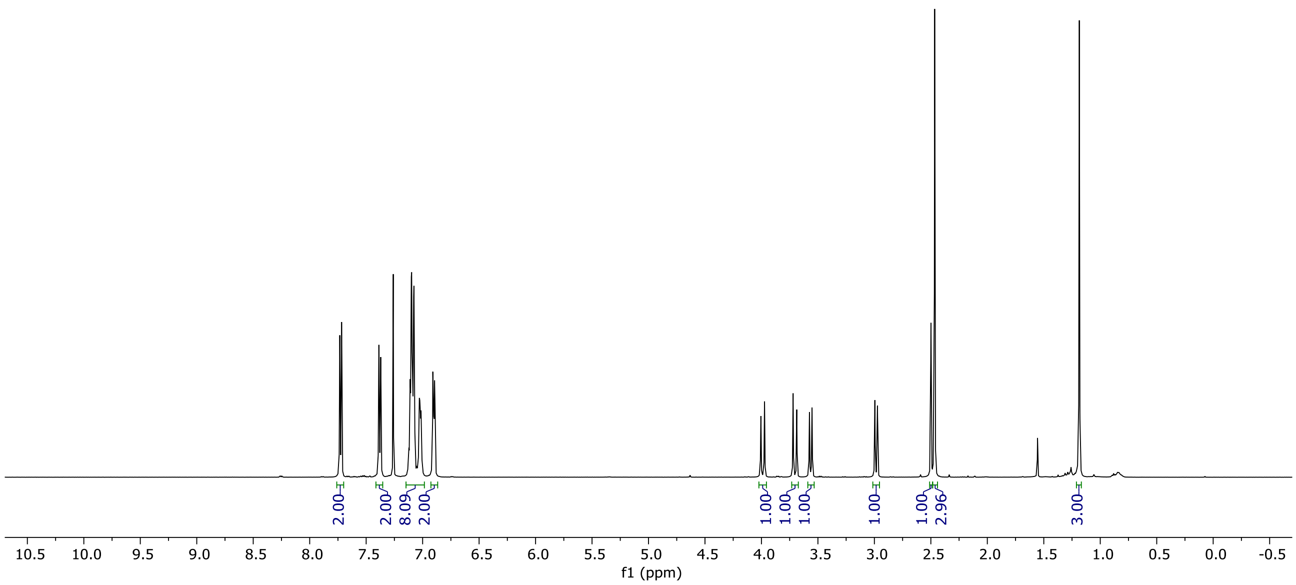
2.00
2.00
8.09
2.00
1.00
1.00
1.00
1.00
1.00
2.96
3.00
f1 (ppm)

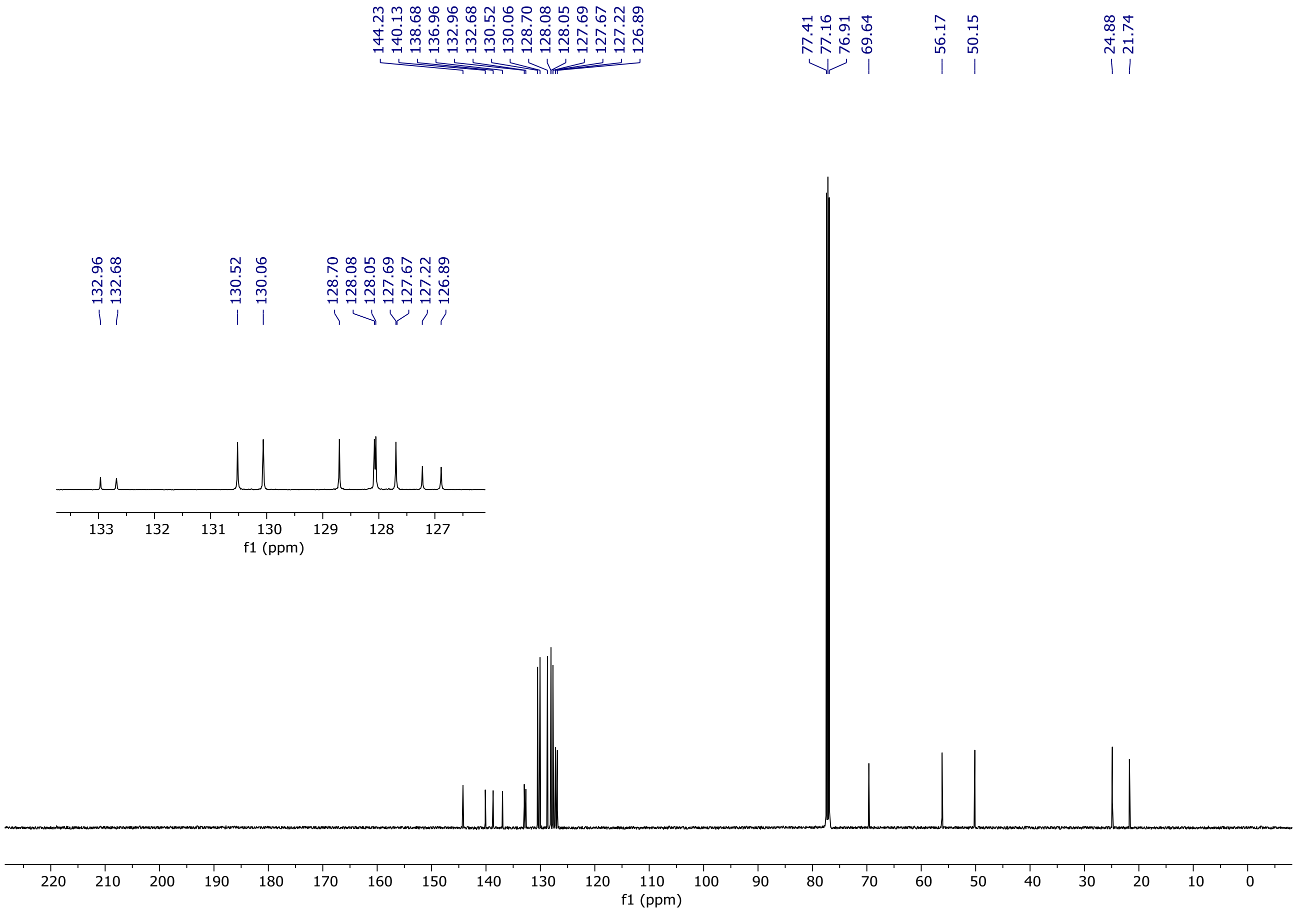
144.23
140.13
138.68
136.96
132.96
132.68
130.52
130.06
128.70
128.08
128.05
127.69
127.67
127.22
126.89
77.41
77.16
76.91
69.64
56.17
50.15
24.88
21.74
f1 (ppm)

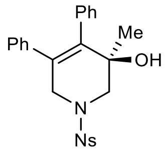
Ph
Me
Ph
OH
N
Ns

2f

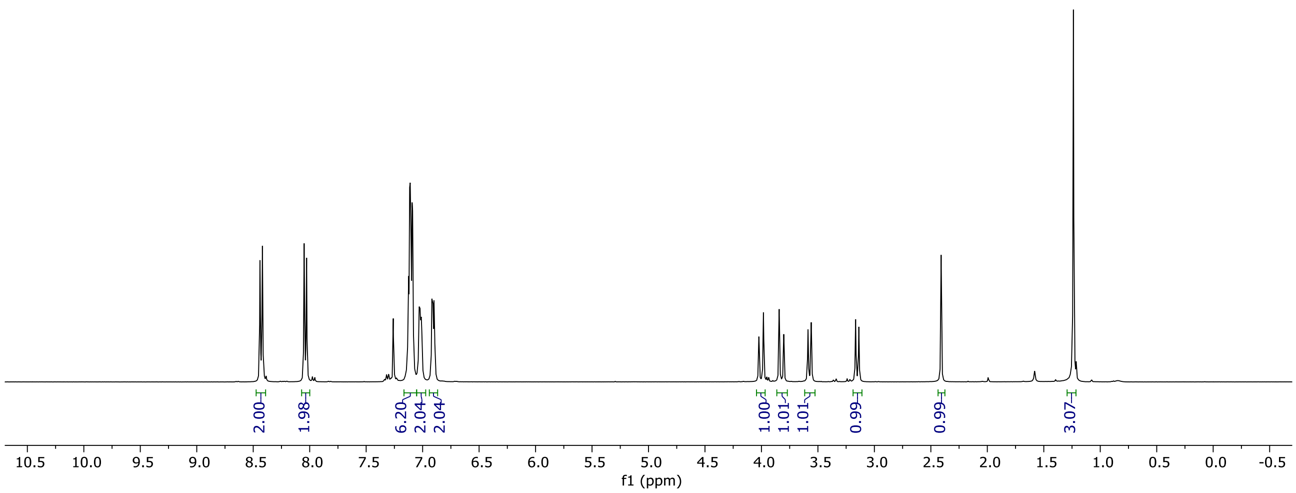
2.00
1.98
6.20
2.04
2.04
1.00
1.01
1.01
0.99
0.99
3.07
10.5 10.0 9.5 9.0 8.5 8.0 7.5 7.0 6.5 6.0 5.5 5.0 4.5 4.0 3.5 3.0 2.5 2.0 1.5 1.0 0.5 0.0 -0.5
f1 (ppm)

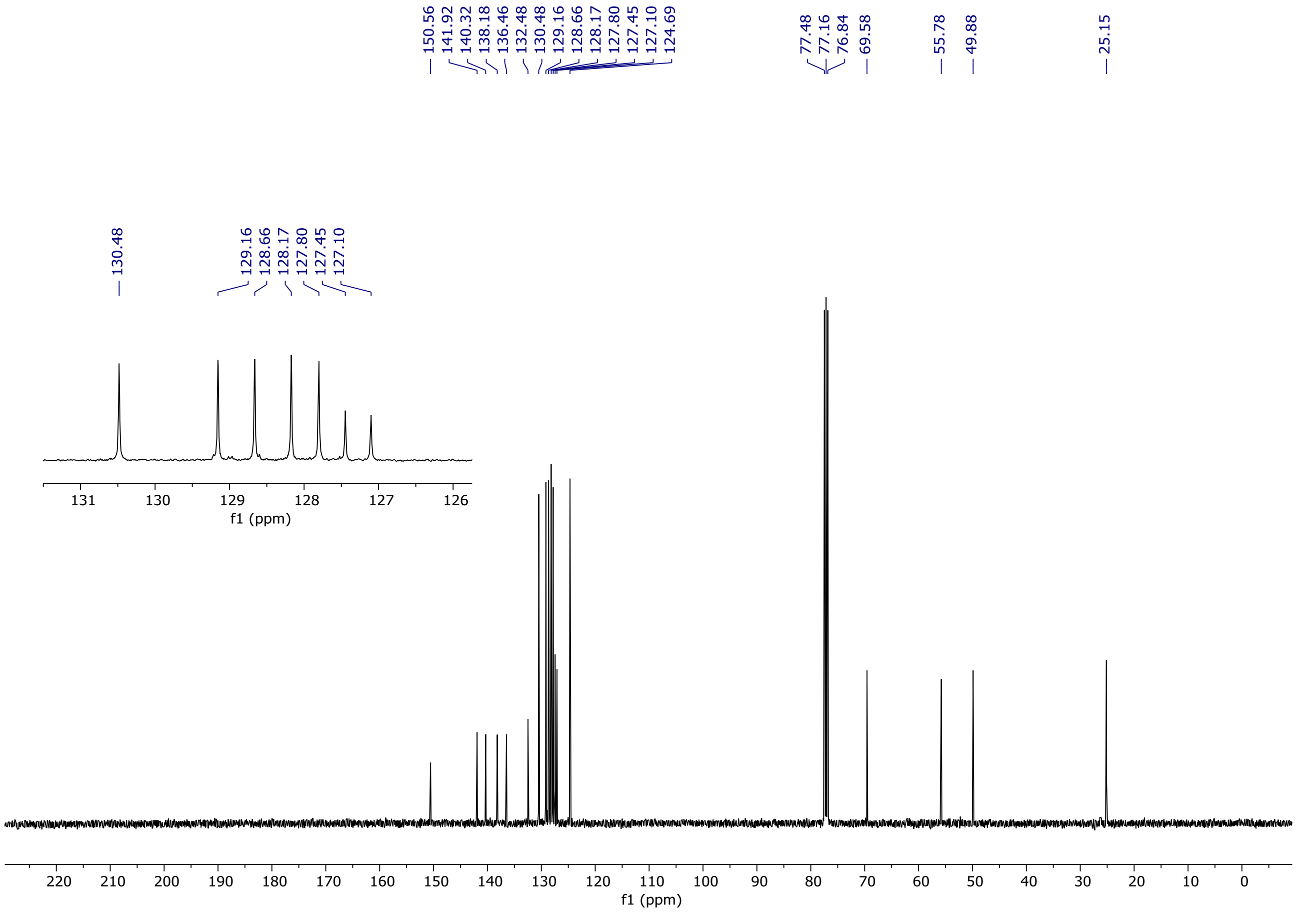
150.56
141.92
140.32
138.18
136.46
132.48
130.48
129.16
128.66
128.17
127.80
127.45
127.10
124.69
77.48
77.16
76.84
69.58
55.78
49.88
25.15
130.48
129.16
128.66
128.17
127.80
127.45
127.10
131 130 129 128 127 126
f1 (ppm)
220 210 200 190 180 170 160 150 140 130 120 110 100 90 80 70 60 50 40 30 20 10 0
f1 (ppm)

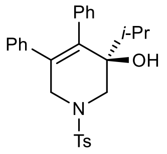
Ph
Ph
i-Pr
OH
N
Ts

2g

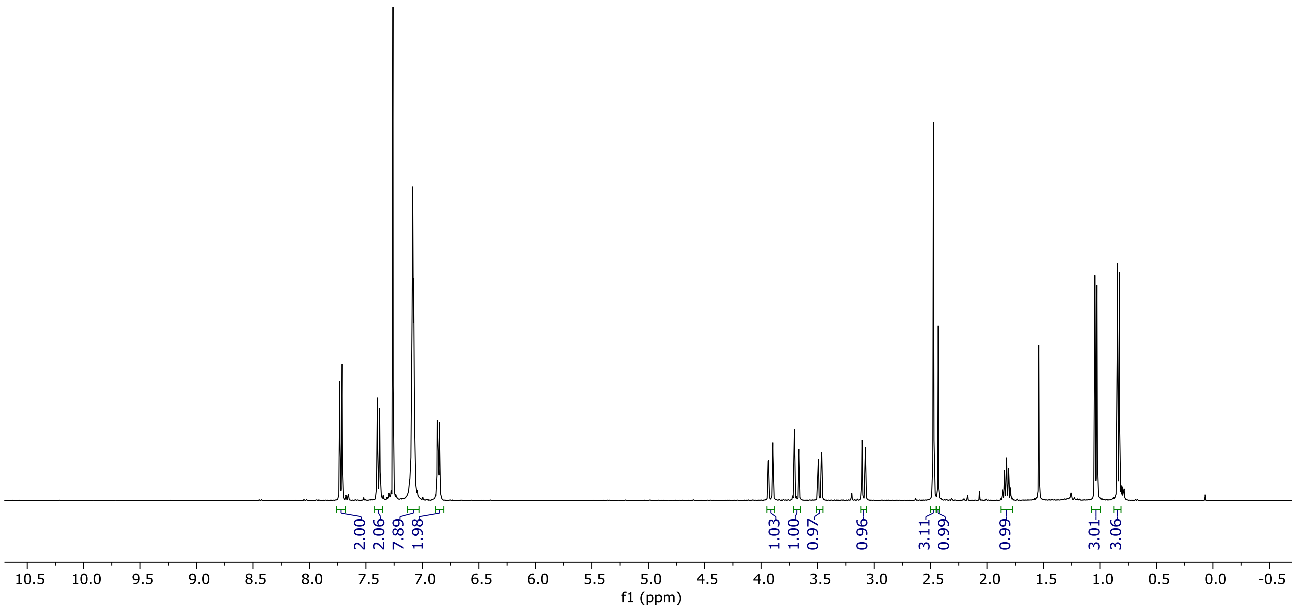
2.00
2.06
7.89
1.98
1.03
1.00
0.97
0.96
3.11
0.99
0.99
3.01
3.06
10.5 10.0 9.5 9.0 8.5 8.0 7.5 7.0 6.5 6.0 5.5 5.0 4.5 4.0 3.5 3.0 2.5 2.0 1.5 1.0 0.5 0.0 -0.5
f1 (ppm)

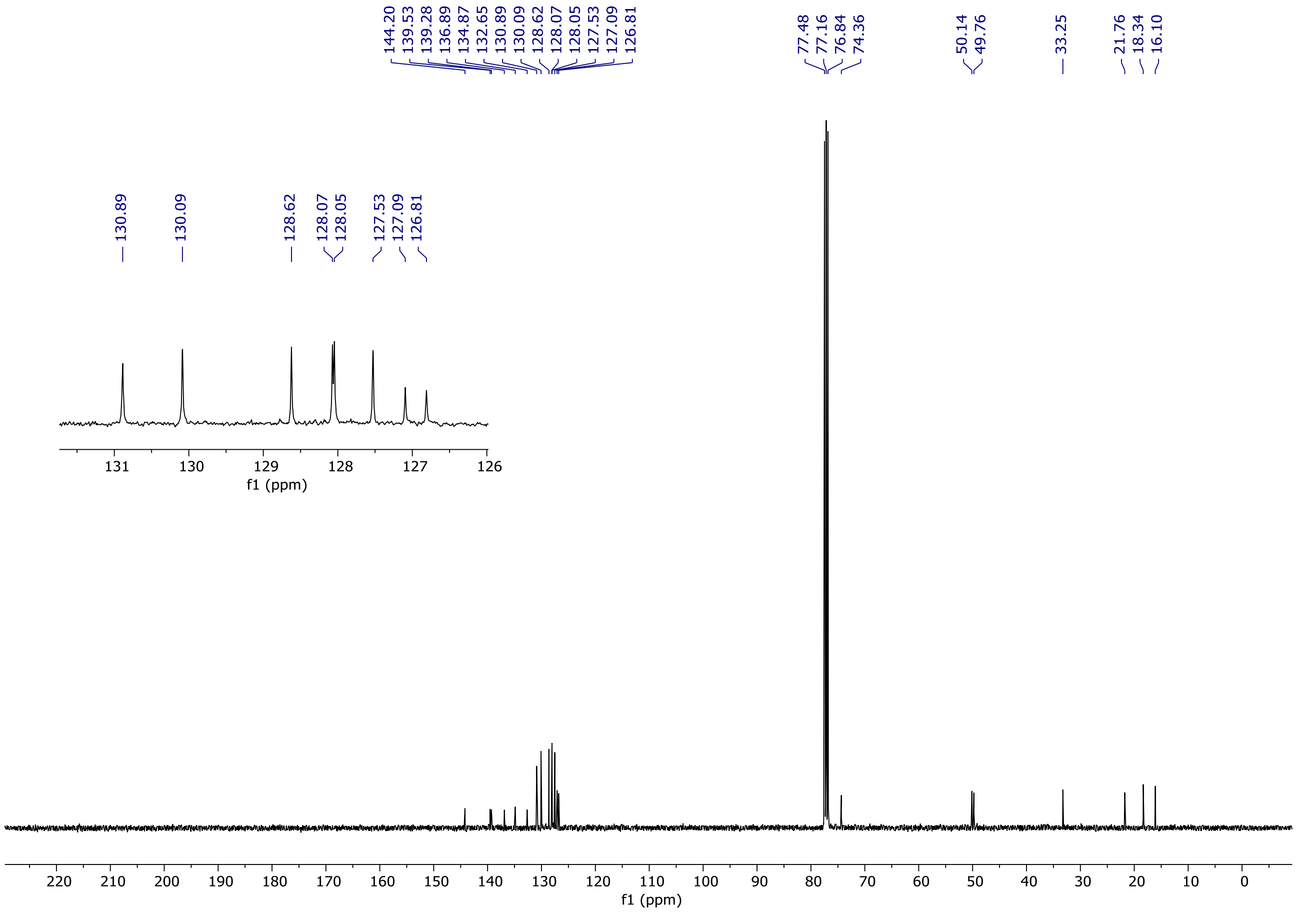
144.20
139.53
139.28
136.89
134.87
132.65
130.89
130.09
128.62
128.07
128.05
127.53
127.09
126.81
77.48
77.16
76.84
74.36
50.14
49.76
33.25
21.76
18.34
16.10
130.89
130.09
128.62
128.07
128.05
127.53
127.09
126.81
131 130 129 128 127 126
f1 (ppm)
220 210 200 190 180 170 160 150 140 130 120 110 100 90 80 70 60 50 40 30 20 10 0
f1 (ppm)

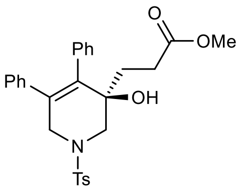
O
OMe
Ph
Ph
OH
N
Ts

2h

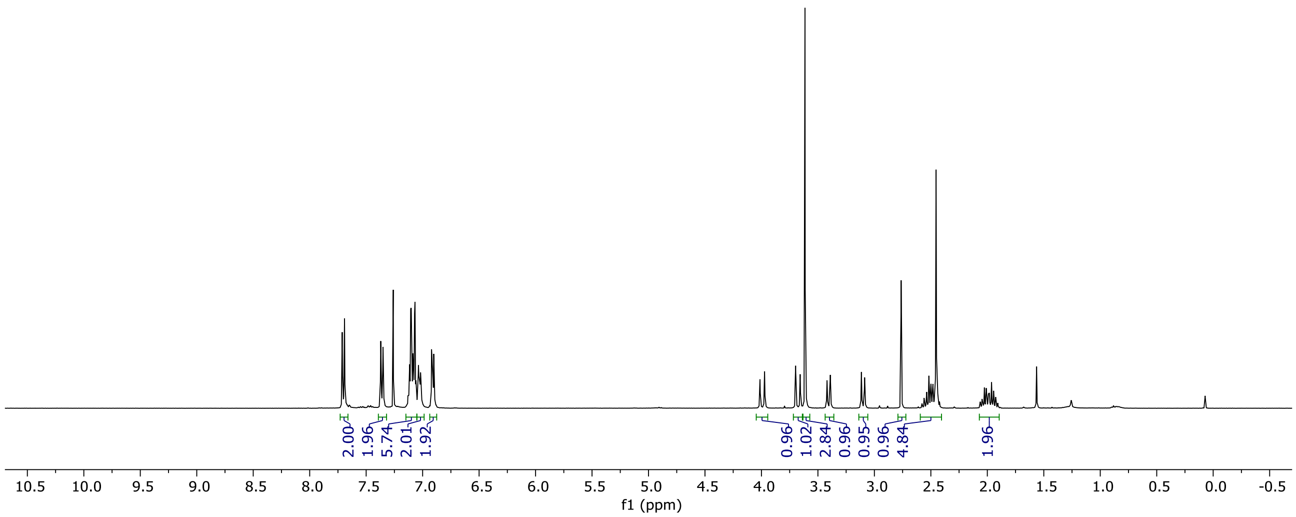
2.00
1.96
5.74
2.01
1.92
0.96
1.02
2.84
0.96
0.95
0.96
4.84
1.96
10.5 10.0 9.5 9.0 8.5 8.0 7.5 7.0 6.5 6.0 5.5 5.0 4.5 4.0 3.5 3.0 2.5 2.0 1.5 1.0 0.5 0.0 -0.5
f1 (ppm)

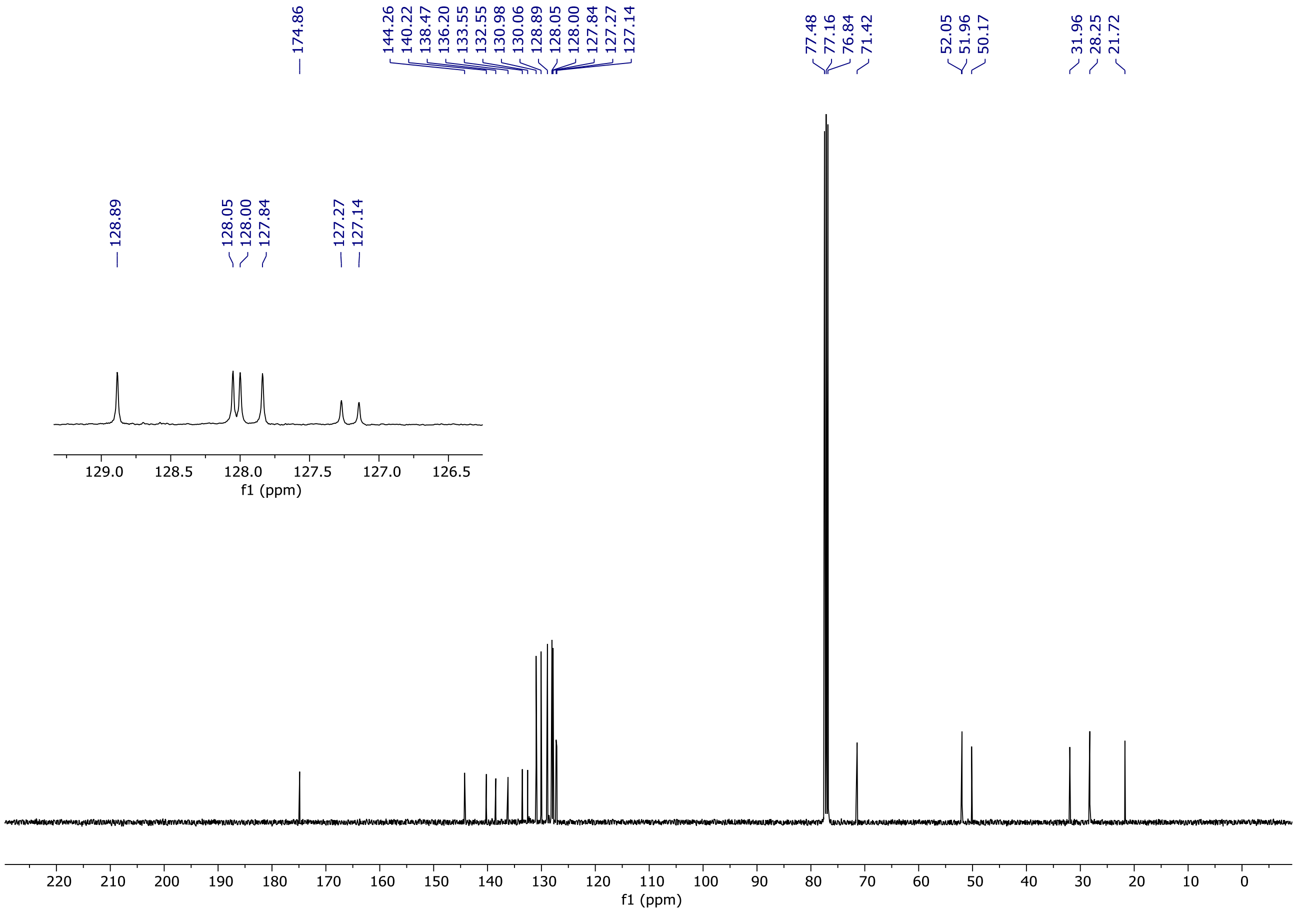
174.86
144.26
140.22
138.47
136.20
133.55
132.55
130.98
130.06
128.89
128.05
128.00
127.84
127.27
127.14
77.48
77.16
76.84
71.42
52.05
51.96
50.17
31.96
28.25
21.72
128.89
128.05
128.00
127.84
127.27
127.14
129.0 128.5 128.0 127.5 127.0 126.5
f1 (ppm)
220 210 200 190 180 170 160 150 140 130 120 110 100 90 80 70 60 50 40 30 20 10 0
f1 (ppm)

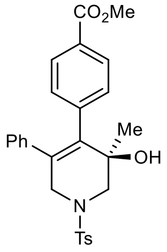
CO2Me
Me
Ph
OH
N
Ts

2i

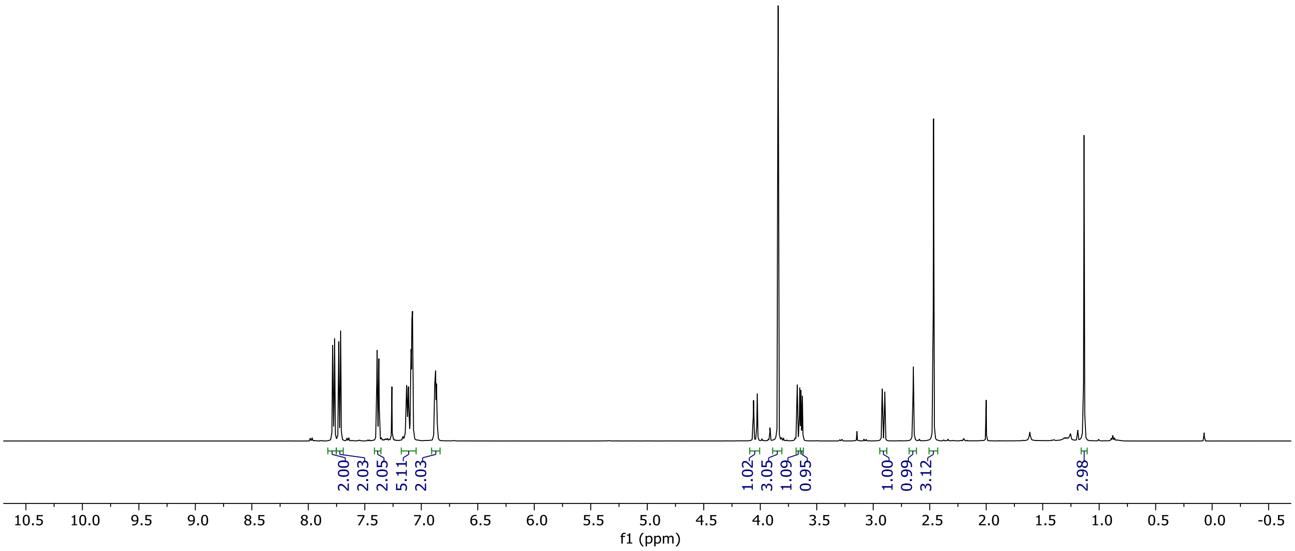
2.00
2.03
2.05
5.11
2.03
1.02
3.05
1.09
0.95
1.00
0.99
3.12
2.98
f1 (ppm)

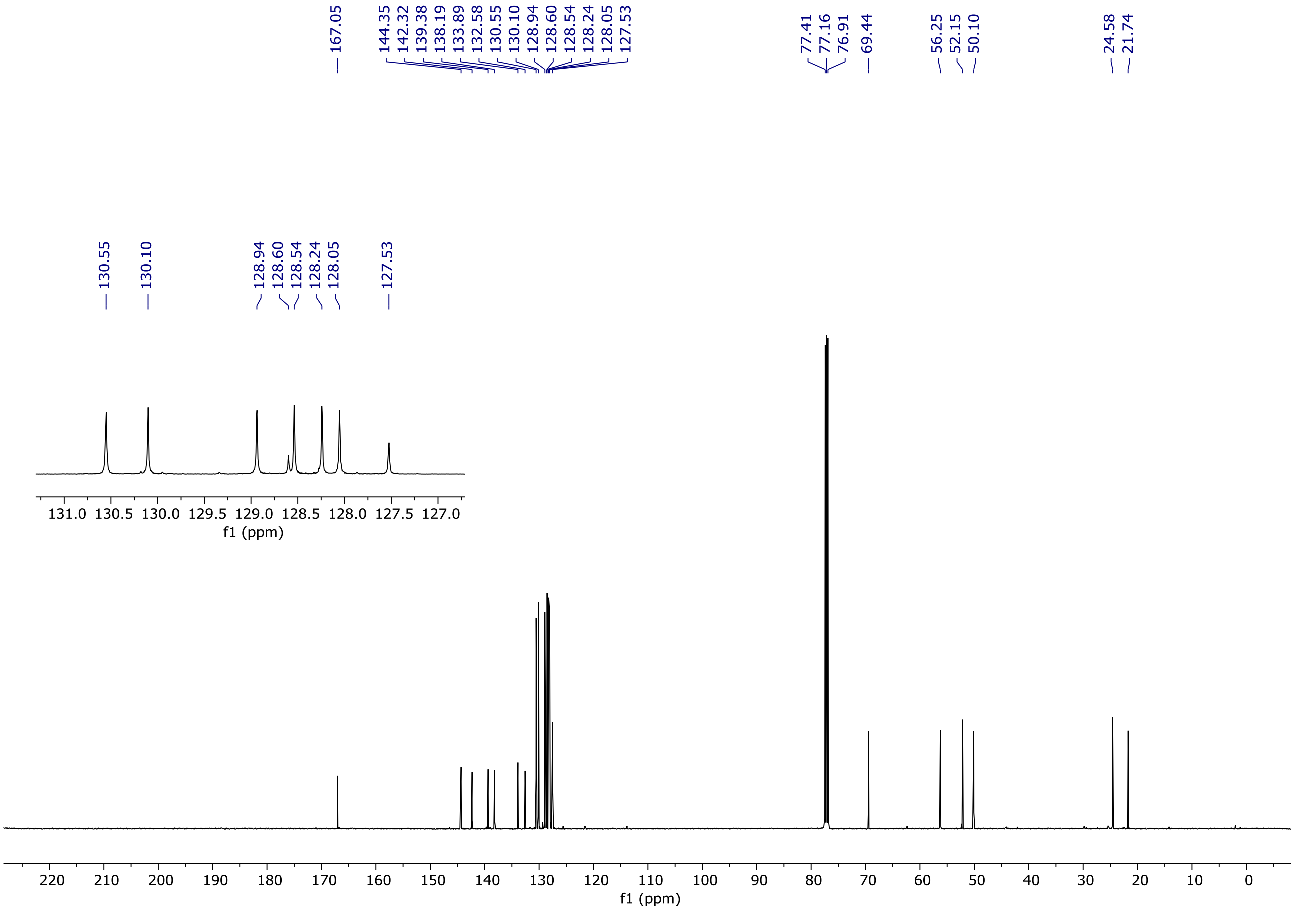
167.05
144.35
142.32
139.38
138.19
133.89
132.58
130.55
130.10
128.94
128.60
128.54
128.24
128.05
127.53
77.41
77.16
76.91
69.44
56.25
52.15
50.10
24.58
21.74
130.55
130.10
128.94
128.60
128.54
128.24
128.05
127.53
f1 (ppm)
f1 (ppm)

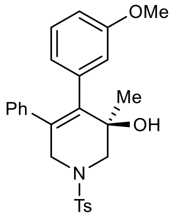
OMe
Me
Ph
OH
N
Ts

2j

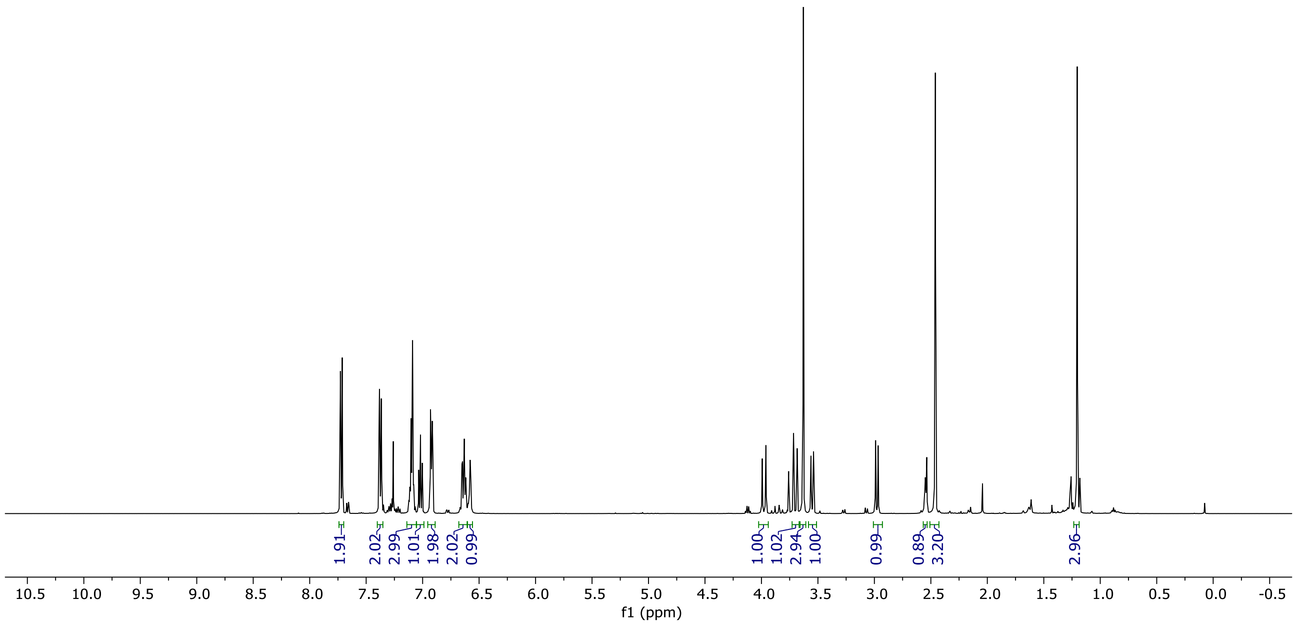
1.91
2.02
2.99
1.01
1.98
2.02
0.99
1.00
1.02
2.94
1.00
0.99
0.89
3.20
2.96
f1 (ppm)

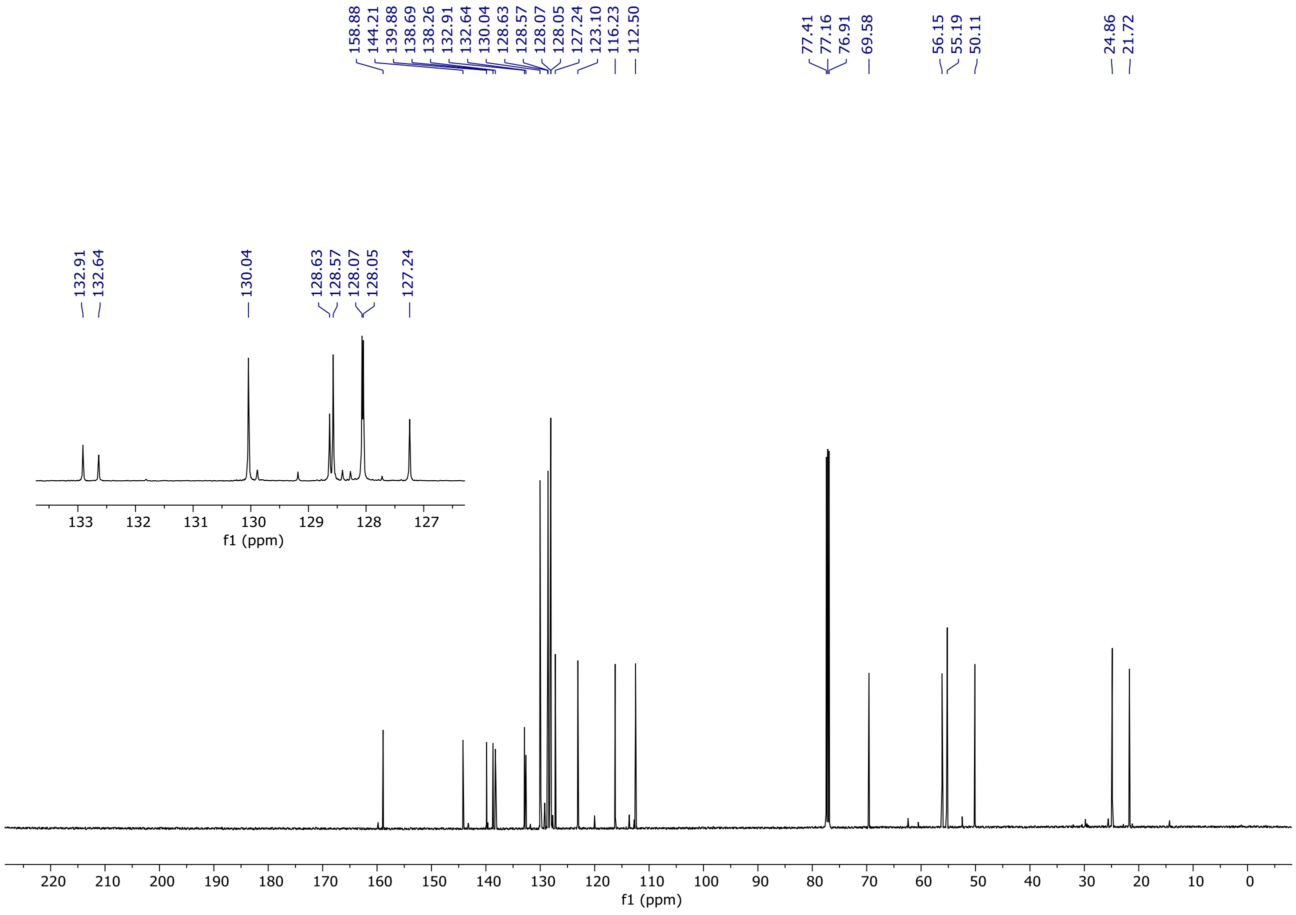
158.88
144.21
139.88
138.69
138.26
132.91
132.64
130.04
128.63
128.57
128.07
128.05
127.24
123.10
116.23
112.50
77.41
77.16
76.91
69.58
56.15
55.19
50.11
24.86
21.72
f1 (ppm)

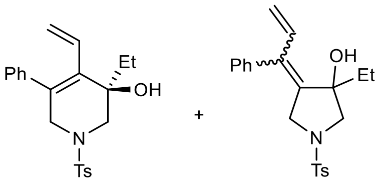

2k **3k**

12:1 inseparable mixture

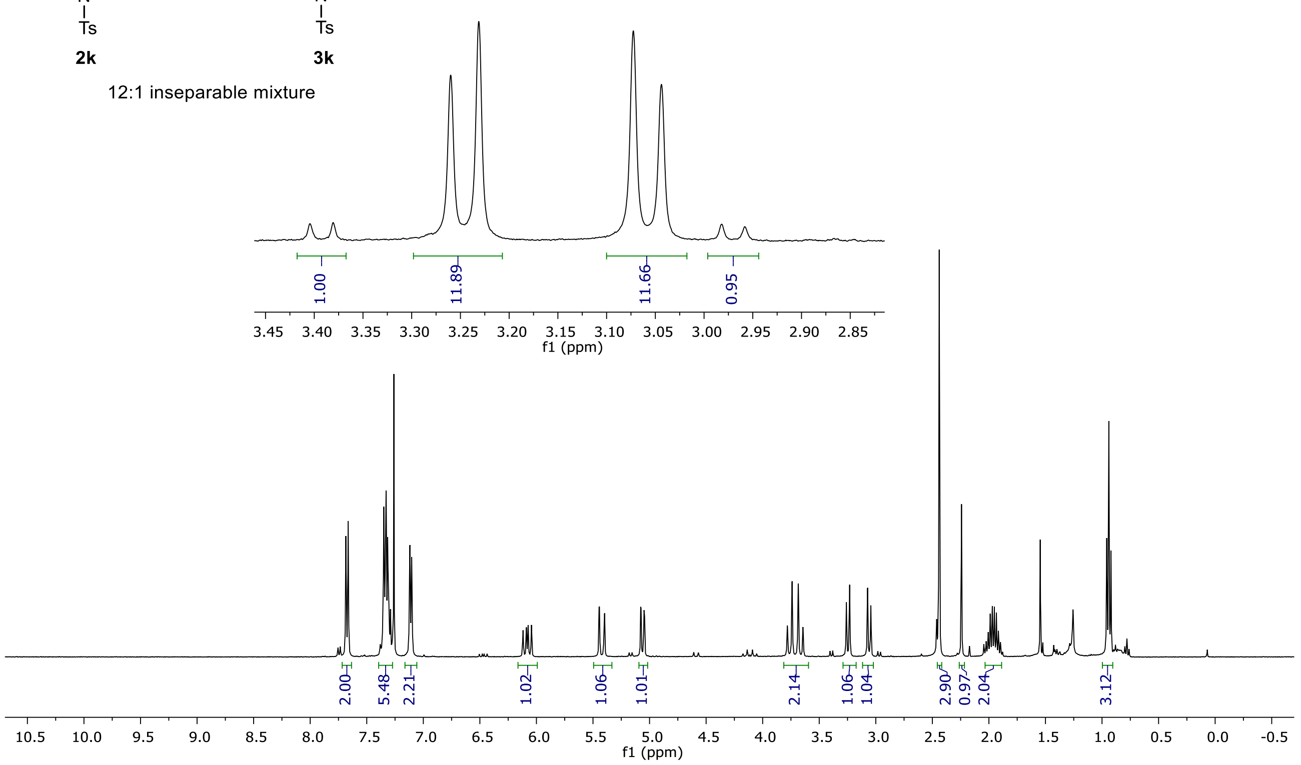

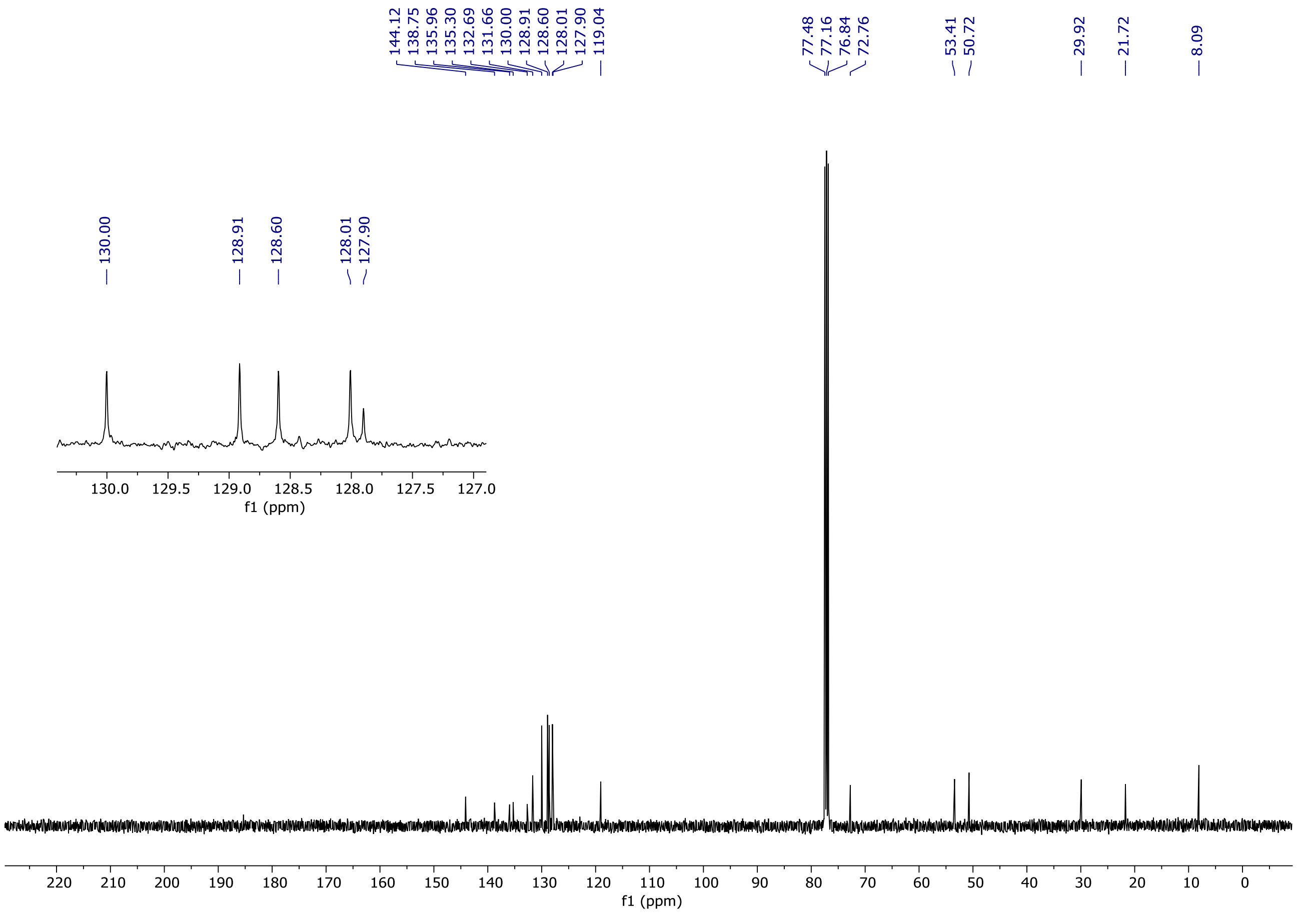

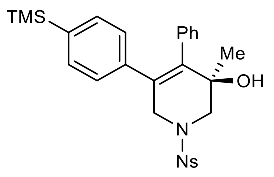
TMS
Ph
Me
OH
N
Ns

2l

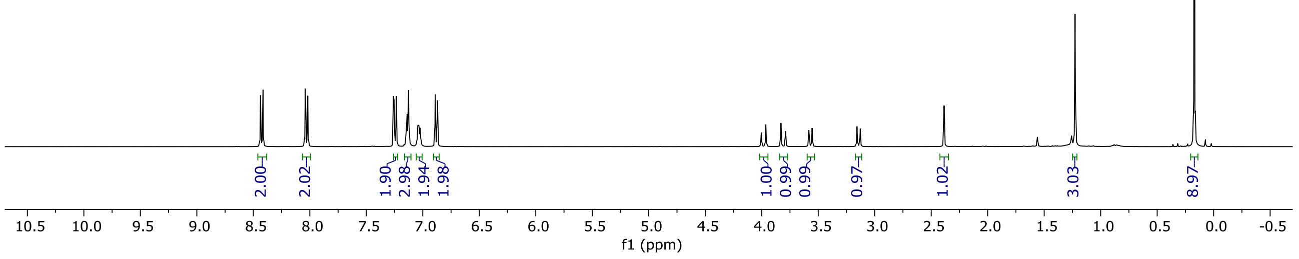
2.00
2.02
1.90
2.98
1.94
1.98
1.00
0.99
0.99
0.97
1.02
3.03
8.97
10.5 10.0 9.5 9.0 8.5 8.0 7.5 7.0 6.5 6.0 5.5 5.0 4.5 4.0 3.5 3.0 2.5 2.0 1.5 1.0 0.5 0.0 -0.5
f1 (ppm)

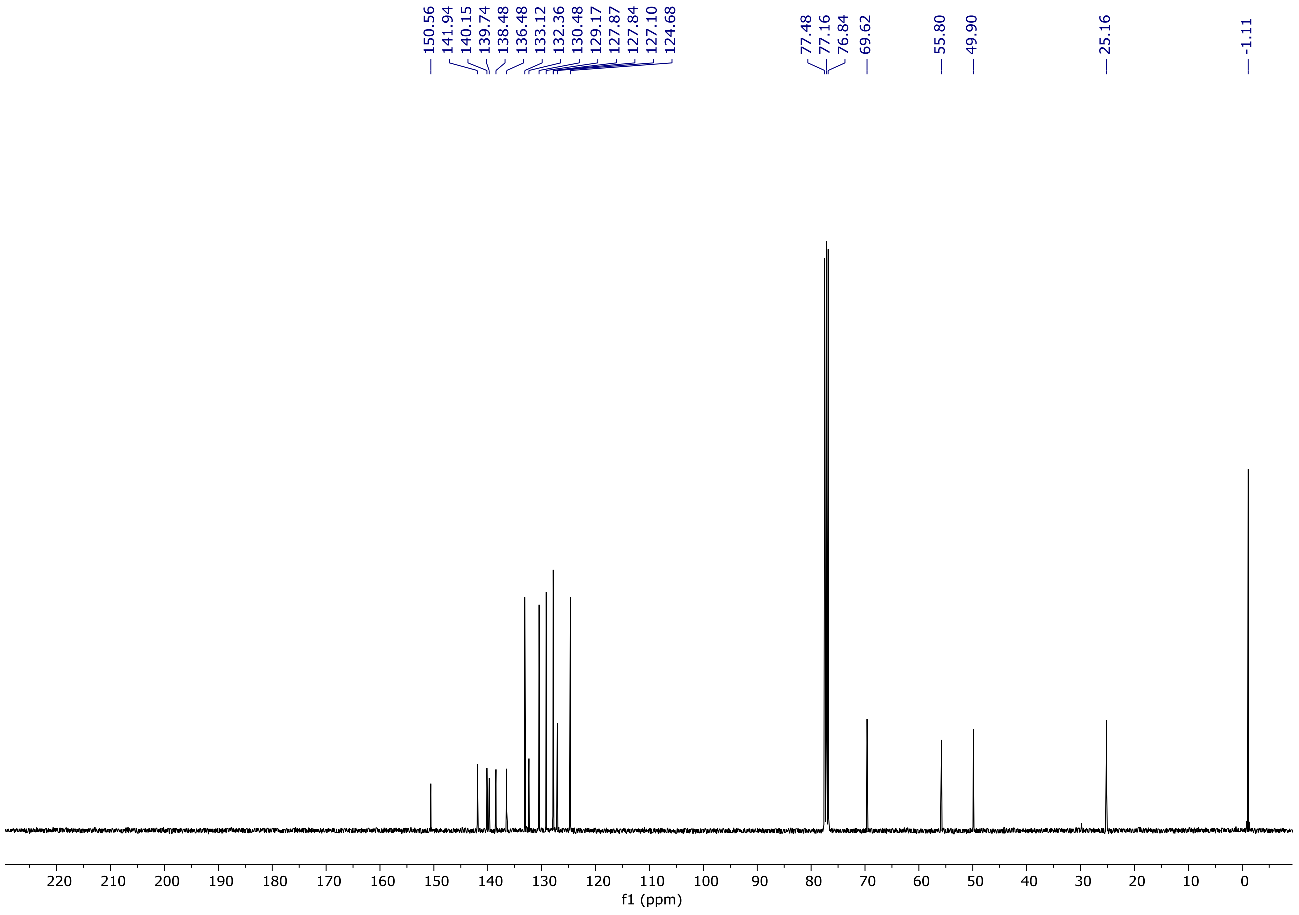
150.56
141.94
140.15
139.74
138.48
136.48
133.12
132.36
130.48
129.17
127.87
127.84
127.10
124.68
77.48
77.16
76.84
69.62
55.80
49.90
25.16
-1.11
220 210 200 190 180 170 160 150 140 130 120 110 100 90 80 70 60 50 40 30 20 10 0
f1 (ppm)

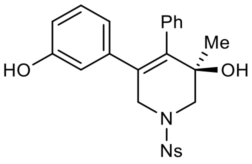
HO
Ph
Me
OH
N
Ns

2m

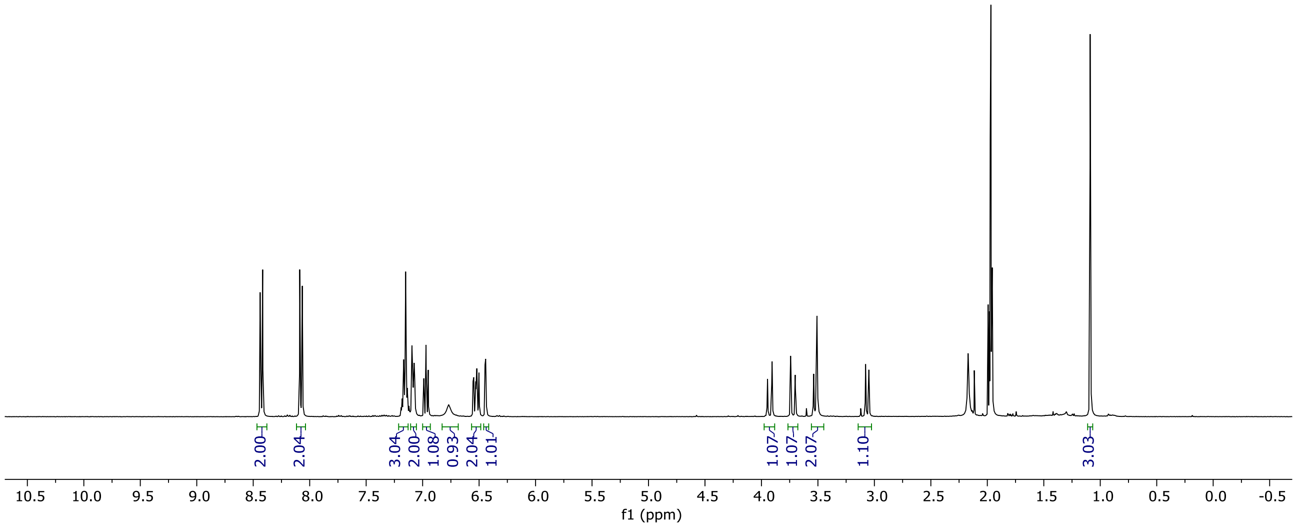
2.00
2.04
3.04
2.00
1.08
0.93
2.04
1.01
1.07
1.07
2.07
1.10
3.03
10.5 10.0 9.5 9.0 8.5 8.0 7.5 7.0 6.5 6.0 5.5 5.0 4.5 4.0 3.5 3.0 2.5 2.0 1.5 1.0 0.5 0.0 -0.5
f1 (ppm)

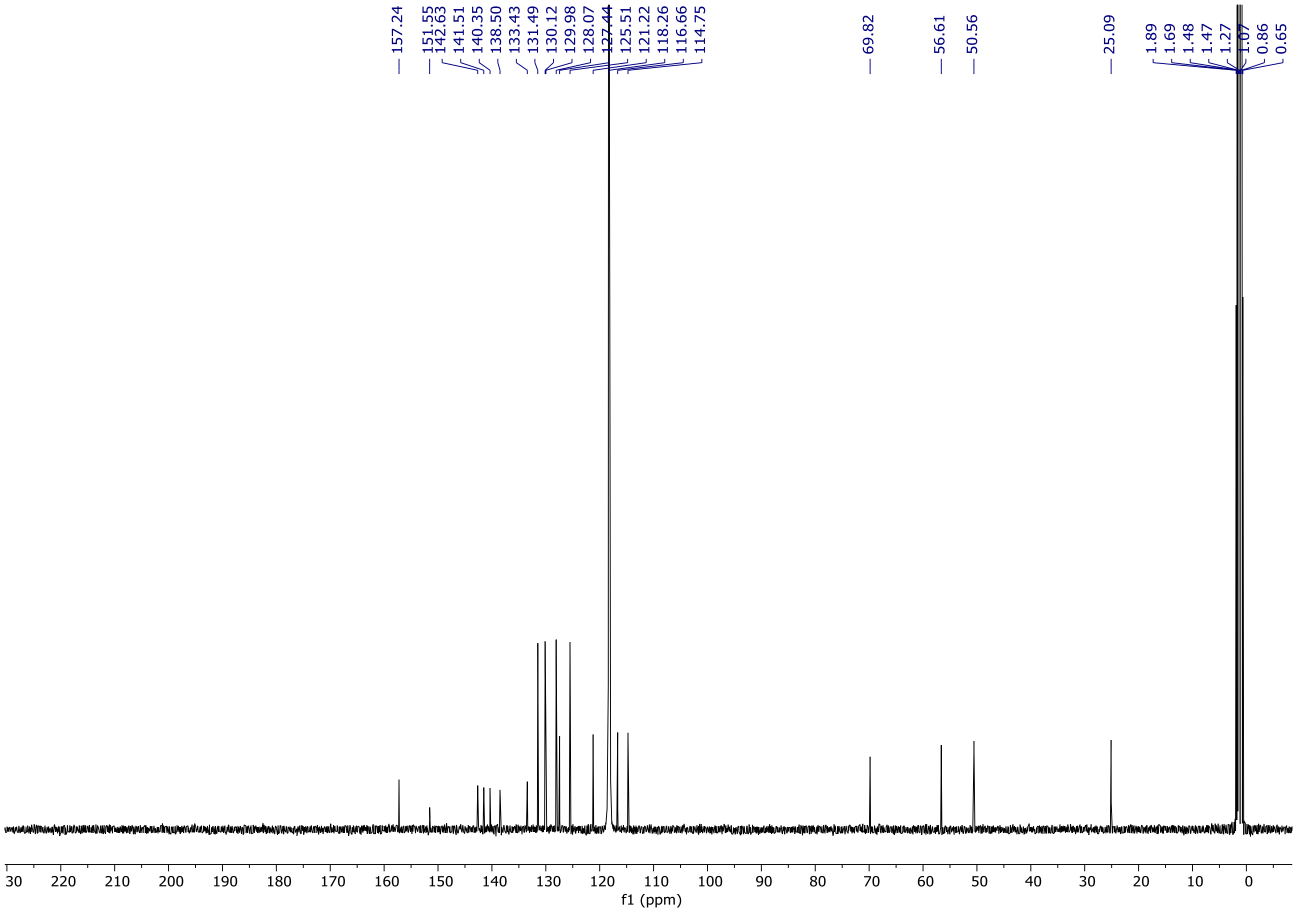
157.24
151.55
142.63
141.51
140.35
138.50
133.43
131.49
130.12
129.98
128.07
127.44
125.51
121.22
118.26
116.66
114.75
69.82
56.61
50.56
25.09
1.89
1.69
1.48
1.47
1.27
1.07
0.86
0.65
30 220 210 200 190 180 170 160 150 140 130 120 110 100 90 80 70 60 50 40 30 20 10 0
f1 (ppm)

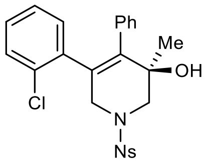
Ph
Me
OH
Cl
N
Ns

2n

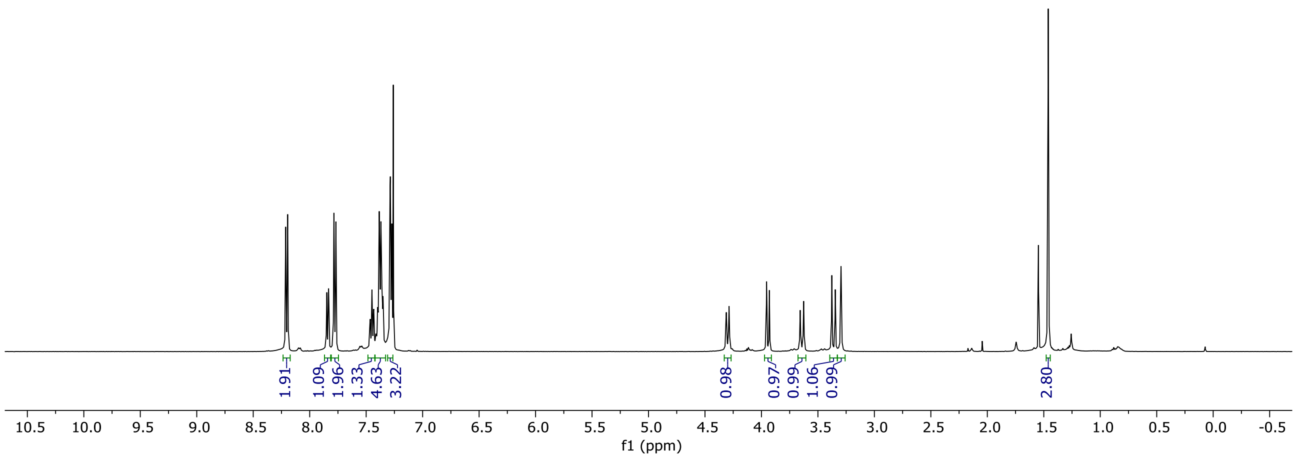
1.91
1.09
1.96
1.33
4.63
3.22
0.98
0.97
0.99
1.06
0.99
2.80
f1 (ppm)

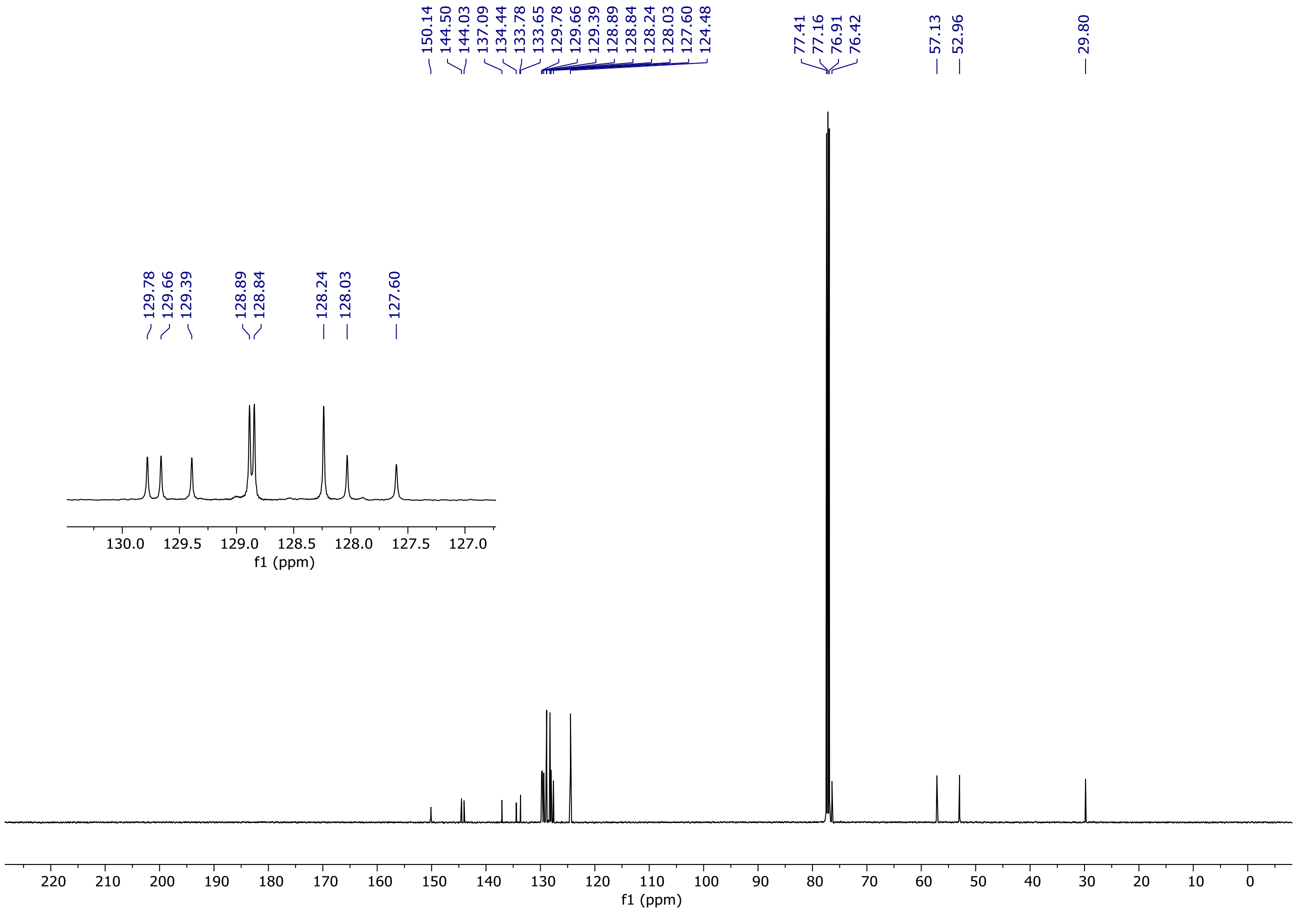
150.14
144.50
144.03
137.09
134.44
133.78
133.65
129.78
129.66
129.39
128.89
128.84
128.24
128.03
127.60
124.48
77.41
77.16
76.91
76.42
57.13
52.96
29.80
f1 (ppm)

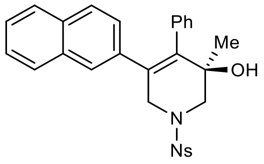
Ph
Me
OH
N
Ns

2o

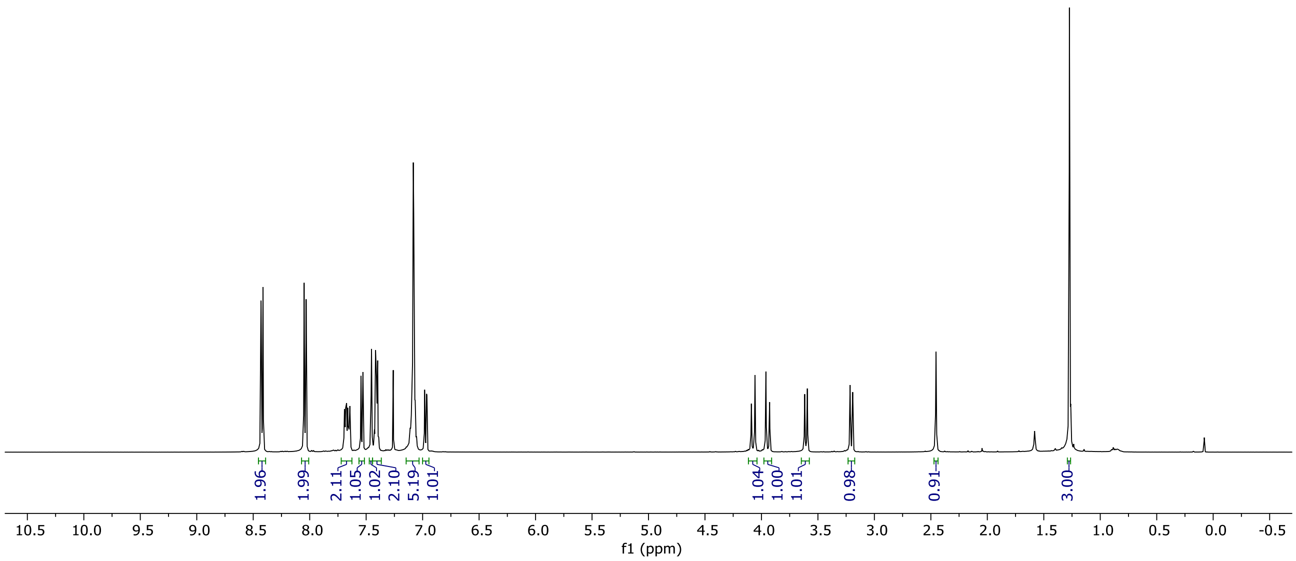
1.96
1.99
2.11
1.05
1.02
2.10
5.19
1.01
1.04
1.00
1.01
0.98
0.91
3.00
f1 (ppm)

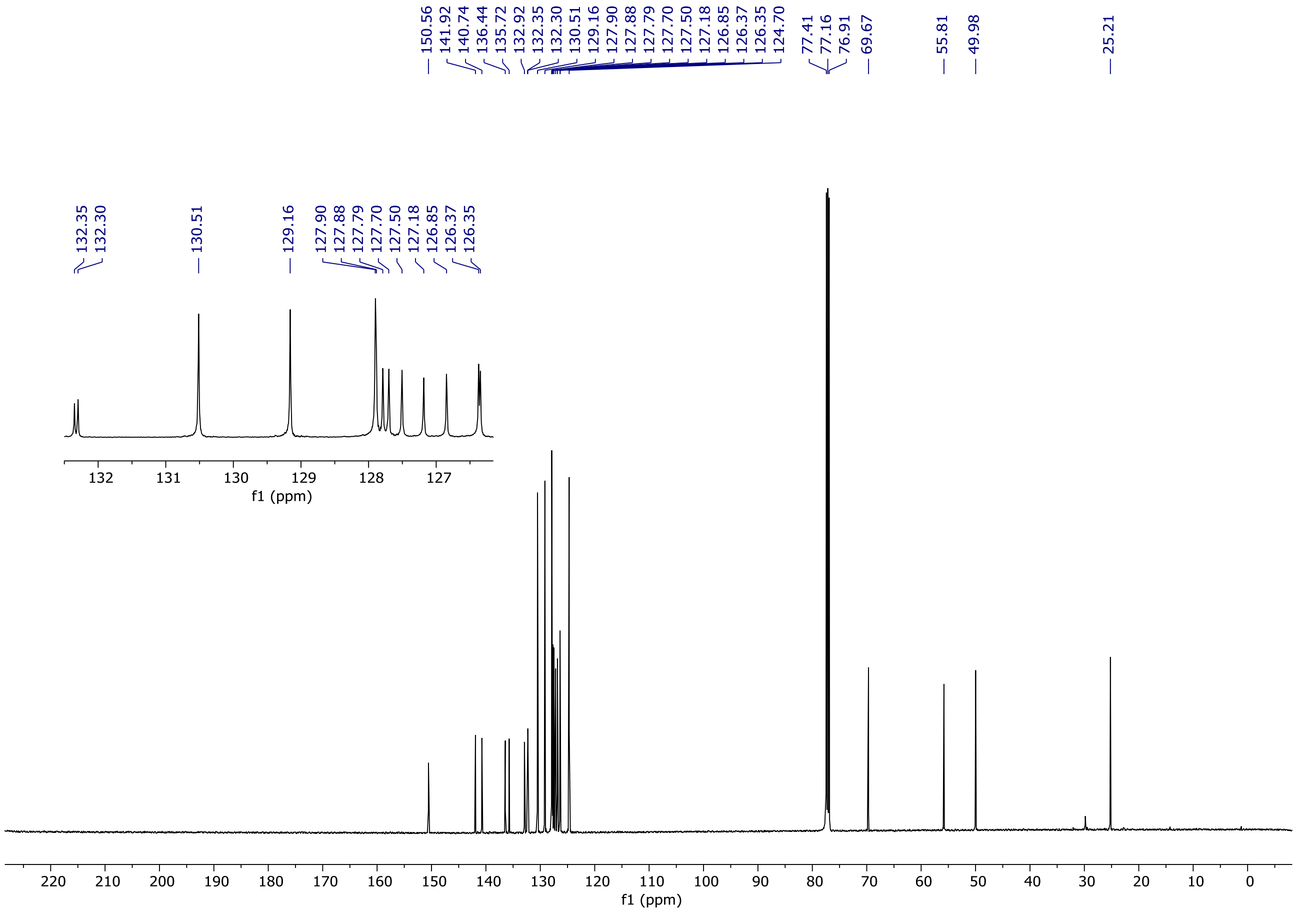
150.56
141.92
140.74
136.44
135.72
132.92
132.35
132.30
130.51
129.16
127.90
127.88
127.79
127.70
127.50
127.18
126.85
126.37
126.35
124.70
77.41
77.16
76.91
69.67
55.81
49.98
25.21
f1 (ppm)

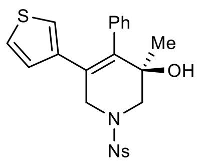
S
Ph
Me
OH
N
Ns

2p

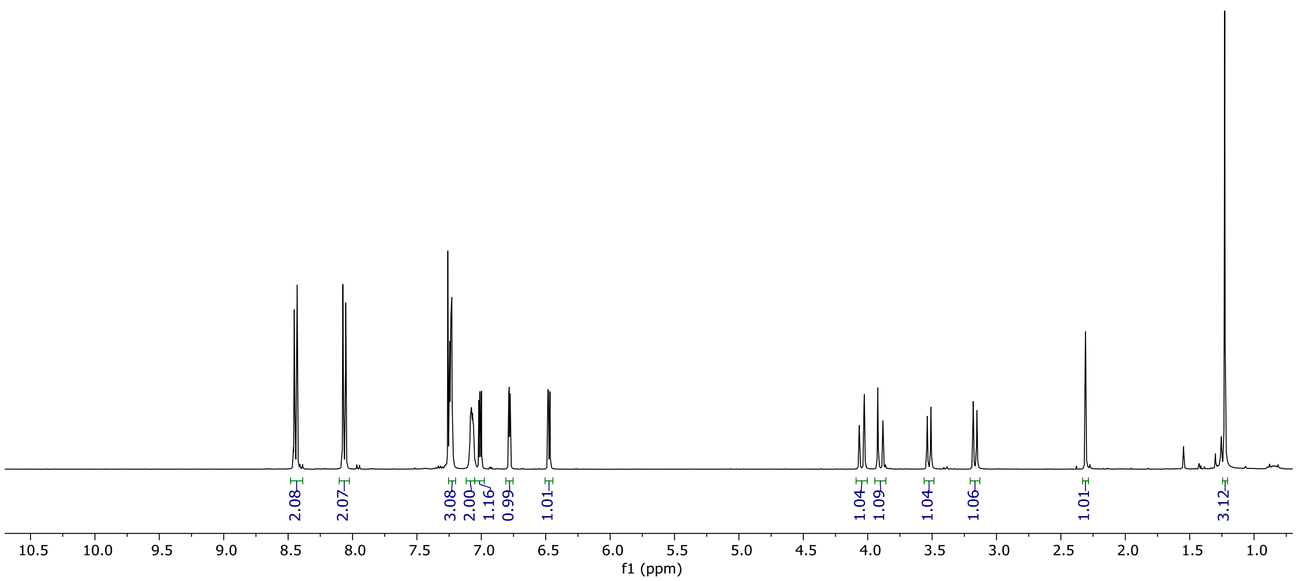
2.08
2.07
3.08
2.00
1.16
0.99
1.01
1.04
1.09
1.04
1.06
1.01
3.12
10.5 10.0 9.5 9.0 8.5 8.0 7.5 7.0 6.5 6.0 5.5 5.0 4.5 4.0 3.5 3.0 2.5 2.0 1.5 1.0
f1 (ppm)

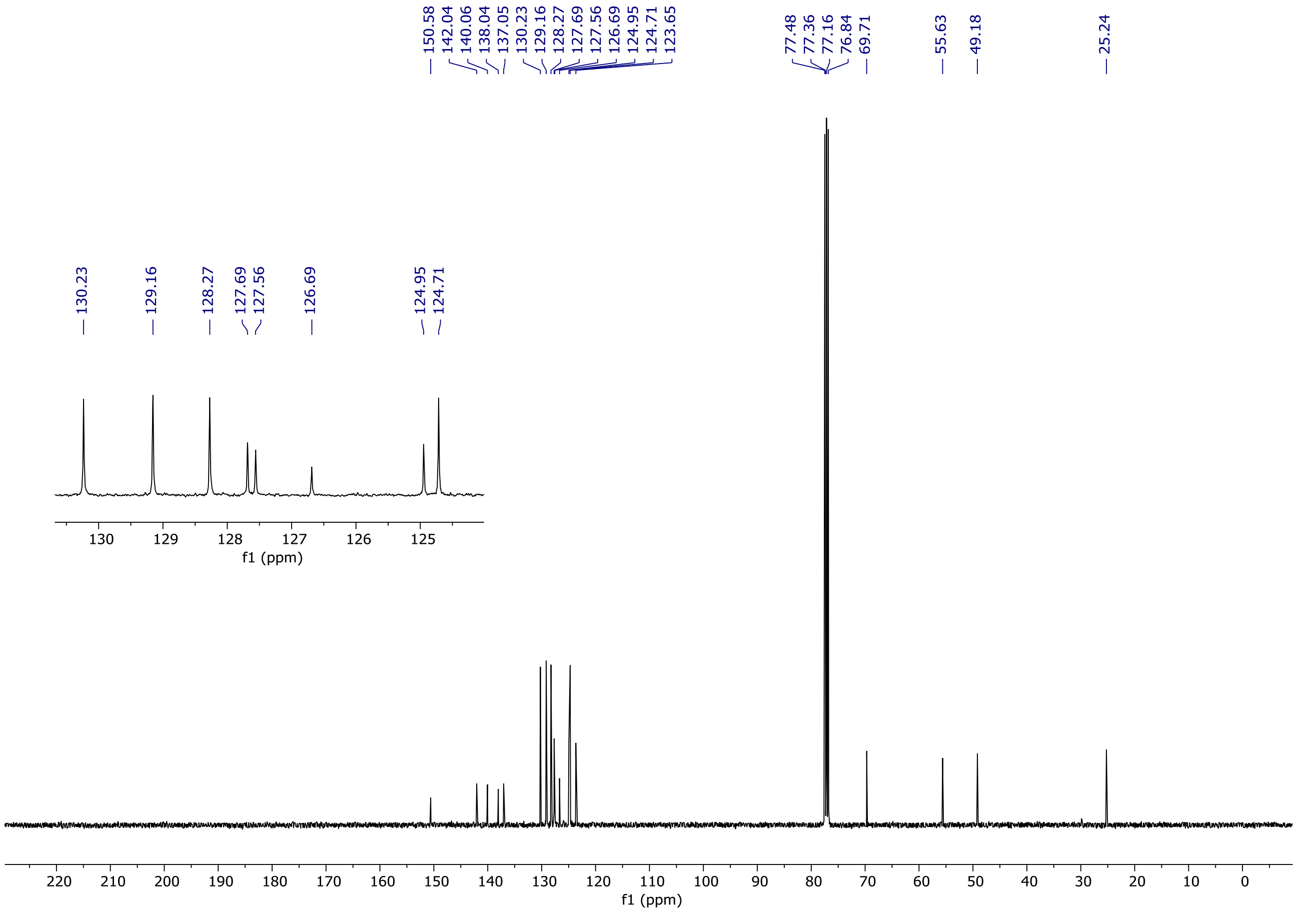
150.58
142.04
140.06
138.04
137.05
130.23
129.16
128.27
127.69
127.56
126.69
124.95
124.71
123.65
77.48
77.36
77.16
76.84
69.71
55.63
49.18
25.24
130.23
129.16
128.27
127.69
127.56
126.69
124.95
124.71
130 129 128 127 126 125
f1 (ppm)
220 210 200 190 180 170 160 150 140 130 120 110 100 90 80 70 60 50 40 30 20 10 0
f1 (ppm)

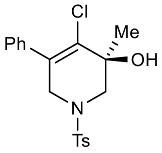
Cl
Me
Ph
OH
N
Ts

2q

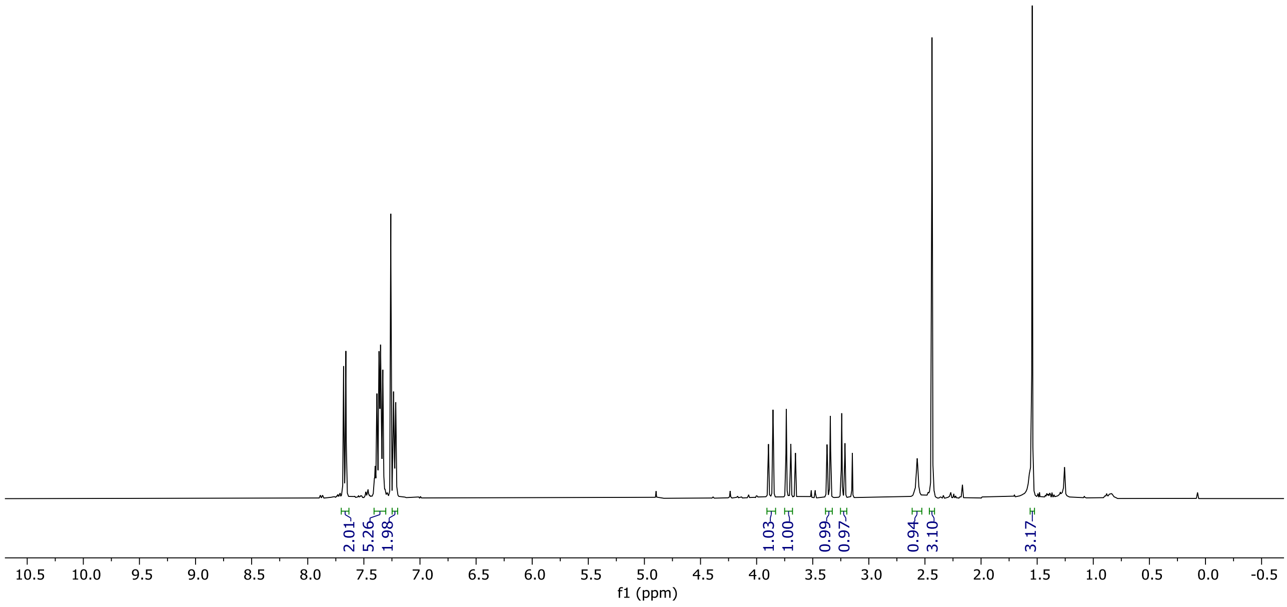
2.01
5.26
1.98
1.03
1.00
0.99
0.97
0.94
3.10
3.17
f1 (ppm)

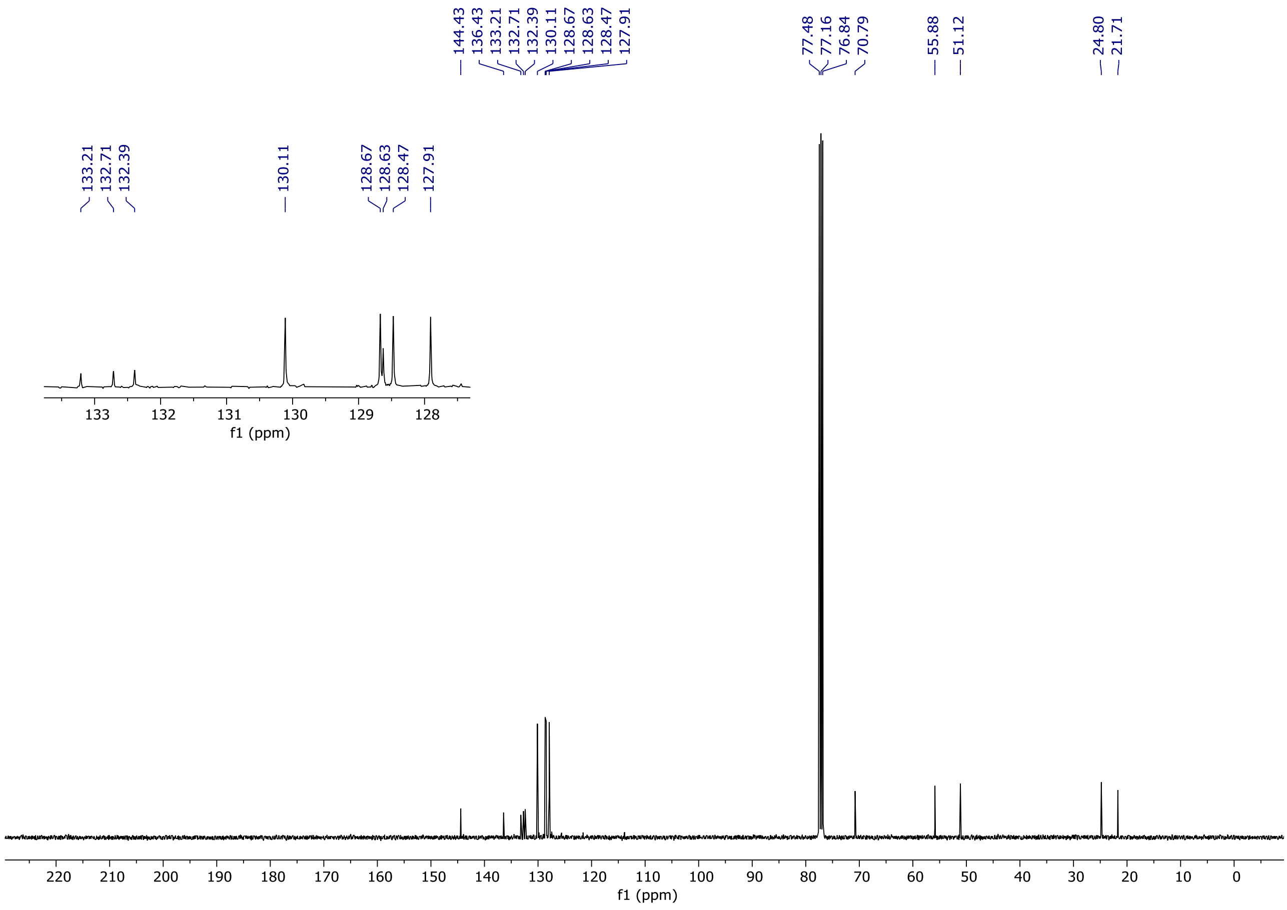
144.43
136.43
133.21
132.71
132.39
130.11
128.67
128.63
128.47
127.91
77.48
77.16
76.84
70.79
55.88
51.12
24.80
21.71
f1 (ppm)

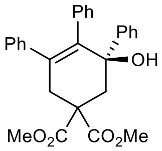
Ph
Ph
Ph
OH
MeO2C
CO2Me

5

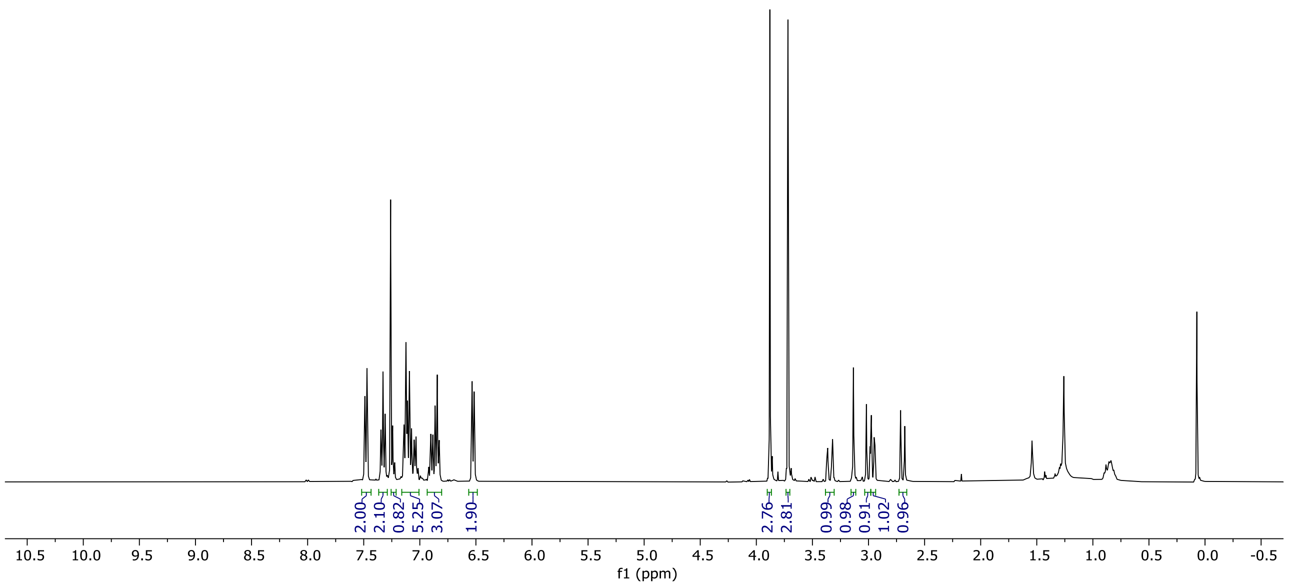
2.00
2.10
0.82
5.25
3.07
1.90
2.76
2.81
0.99
0.98
0.91
1.02
0.96
10.5 10.0 9.5 9.0 8.5 8.0 7.5 7.0 6.5 6.0 5.5 5.0 4.5 4.0 3.5 3.0 2.5 2.0 1.5 1.0 0.5 0.0 -0.5
f1 (ppm)

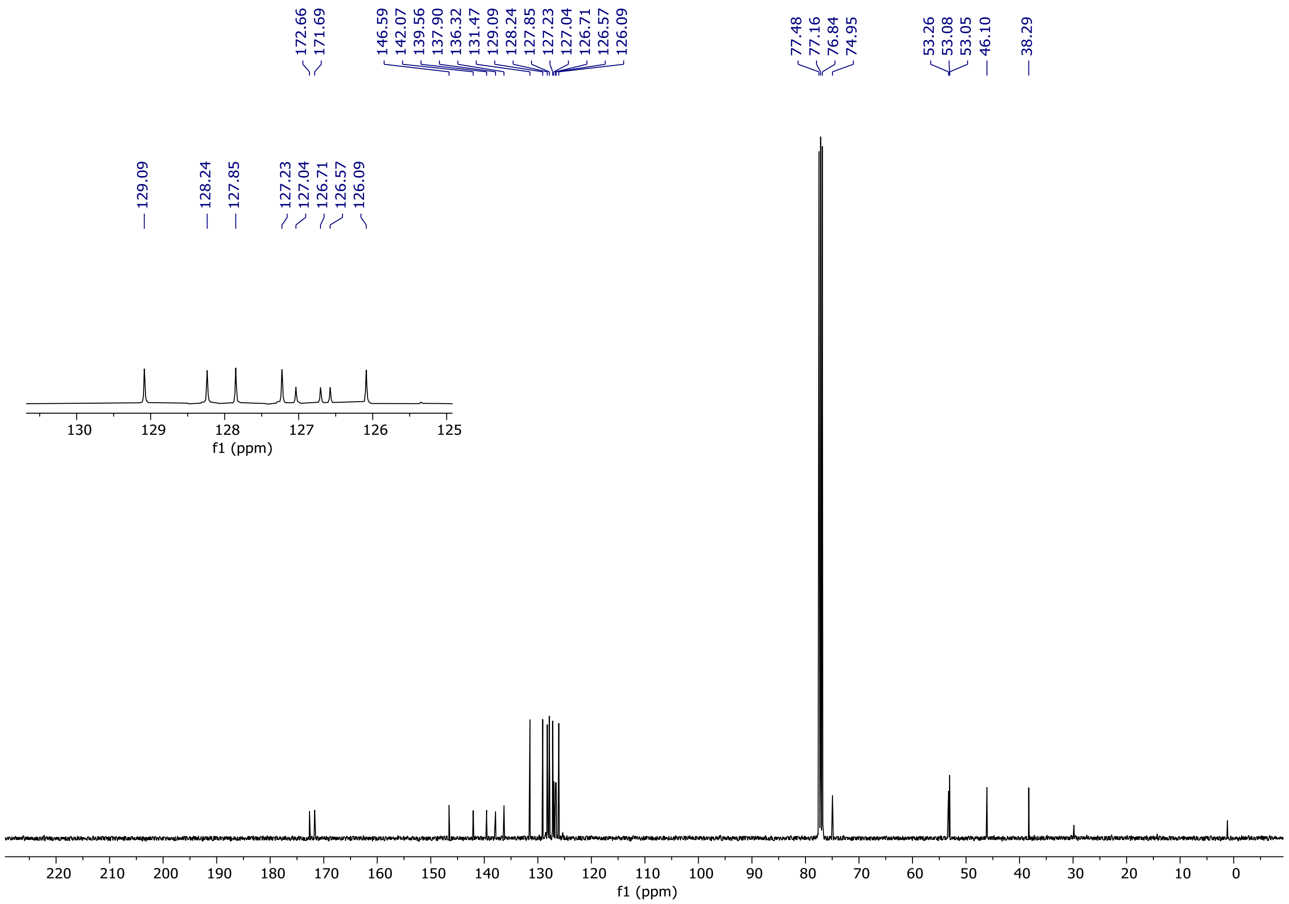
172.66
171.69
146.59
142.07
139.56
137.90
136.32
131.47
129.09
128.24
127.85
127.23
127.04
126.71
126.57
126.09
77.48
77.16
76.84
74.95
53.26
53.08
53.05
46.10
38.29
129.09
128.24
127.85
127.23
127.04
126.71
126.57
126.09
130 129 128 127 126 125
f1 (ppm)
220 210 200 190 180 170 160 150 140 130 120 110 100 90 80 70 60 50 40 30 20 10 0
f1 (ppm)

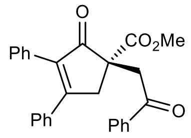

6a (*ca*. 65% purity)

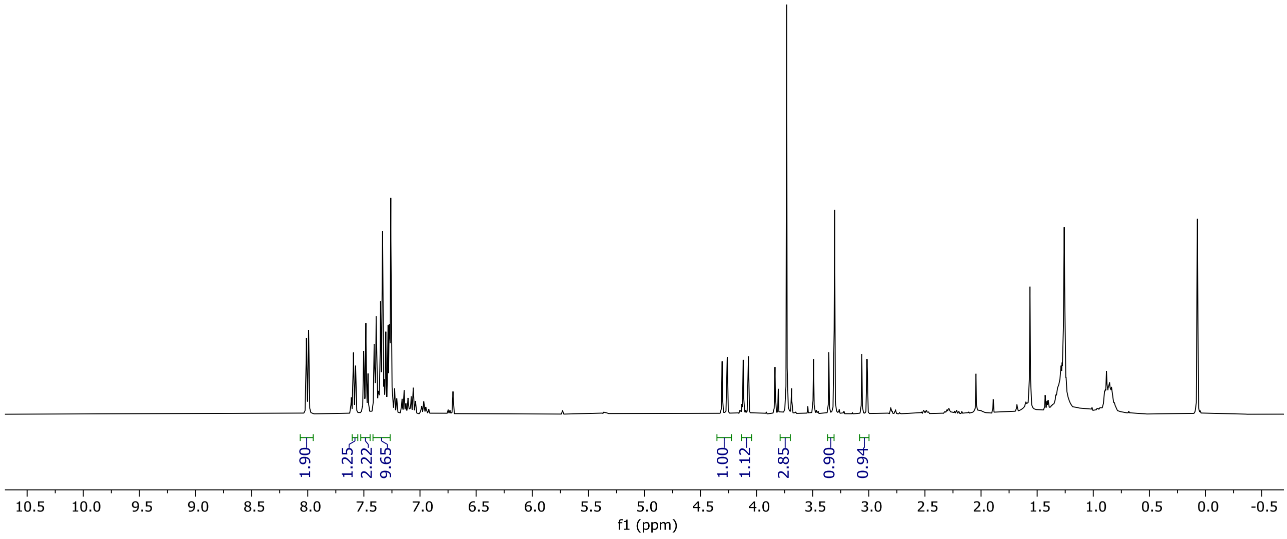

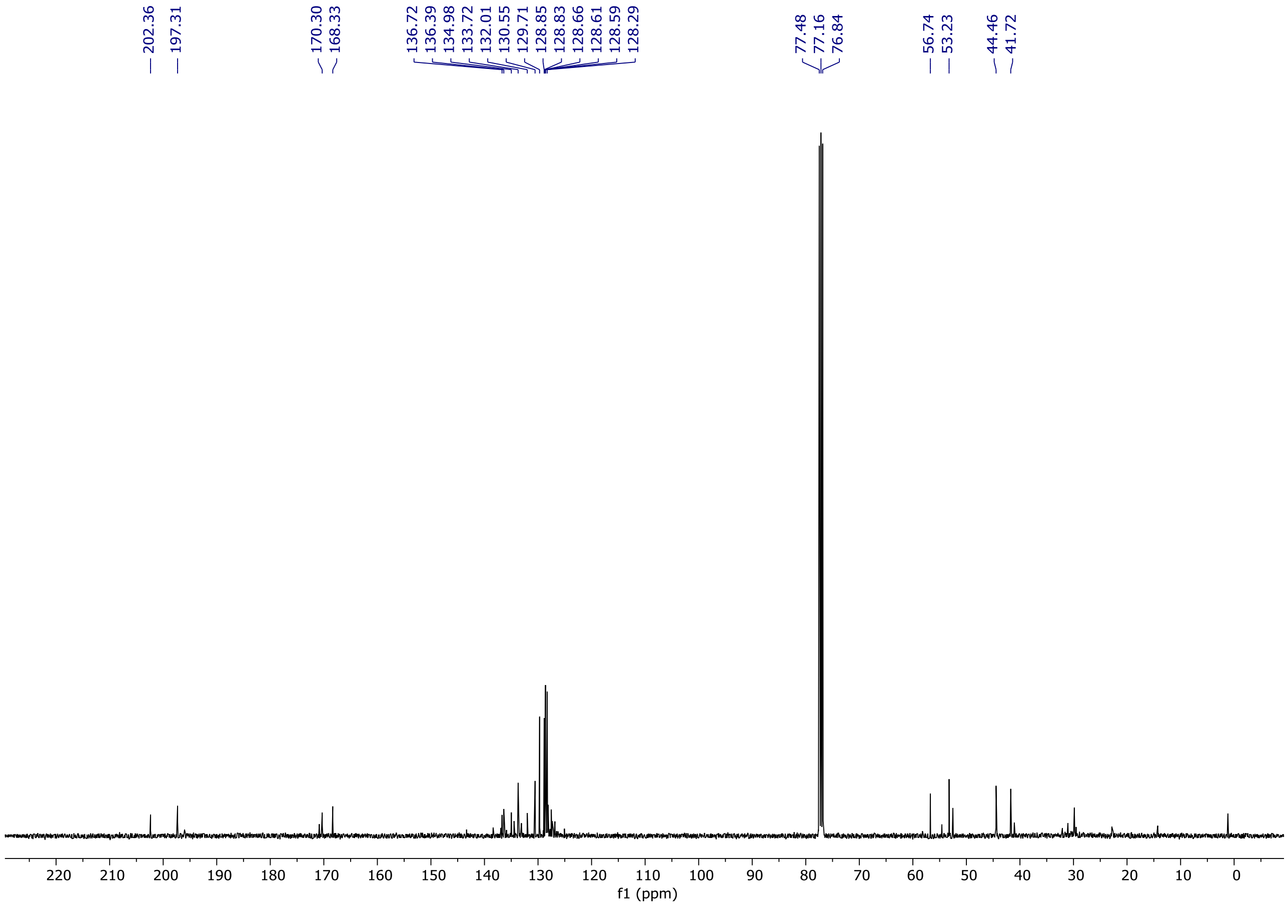

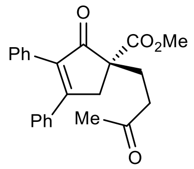

6b
(tentative assignment
of absolute configuration)

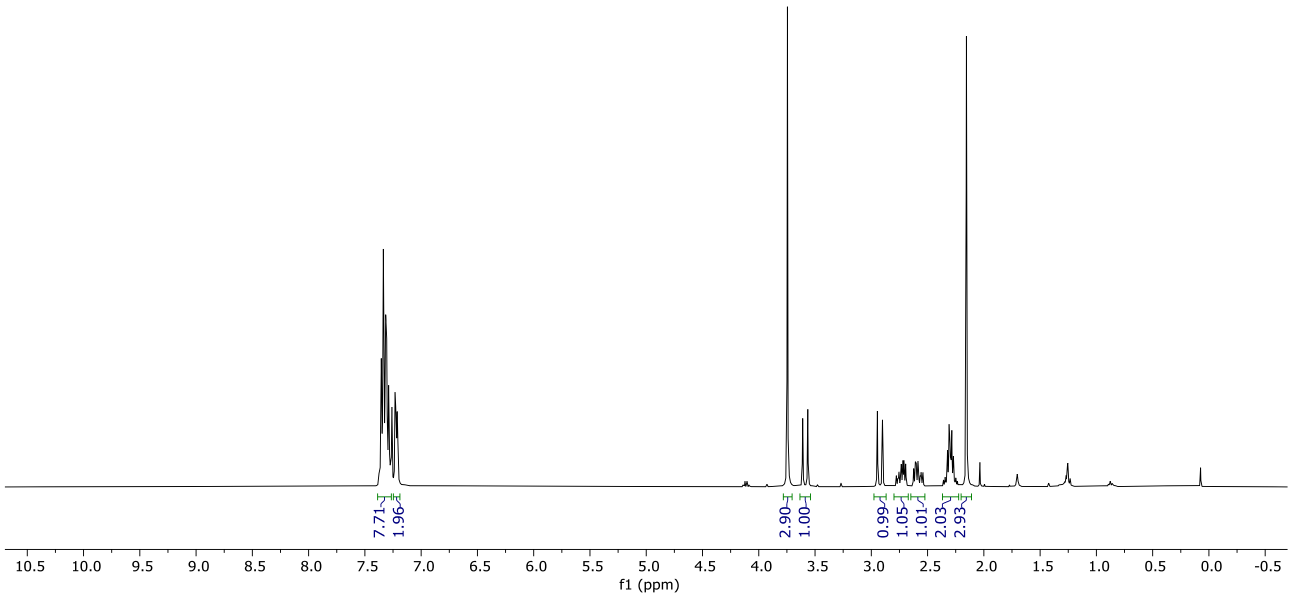

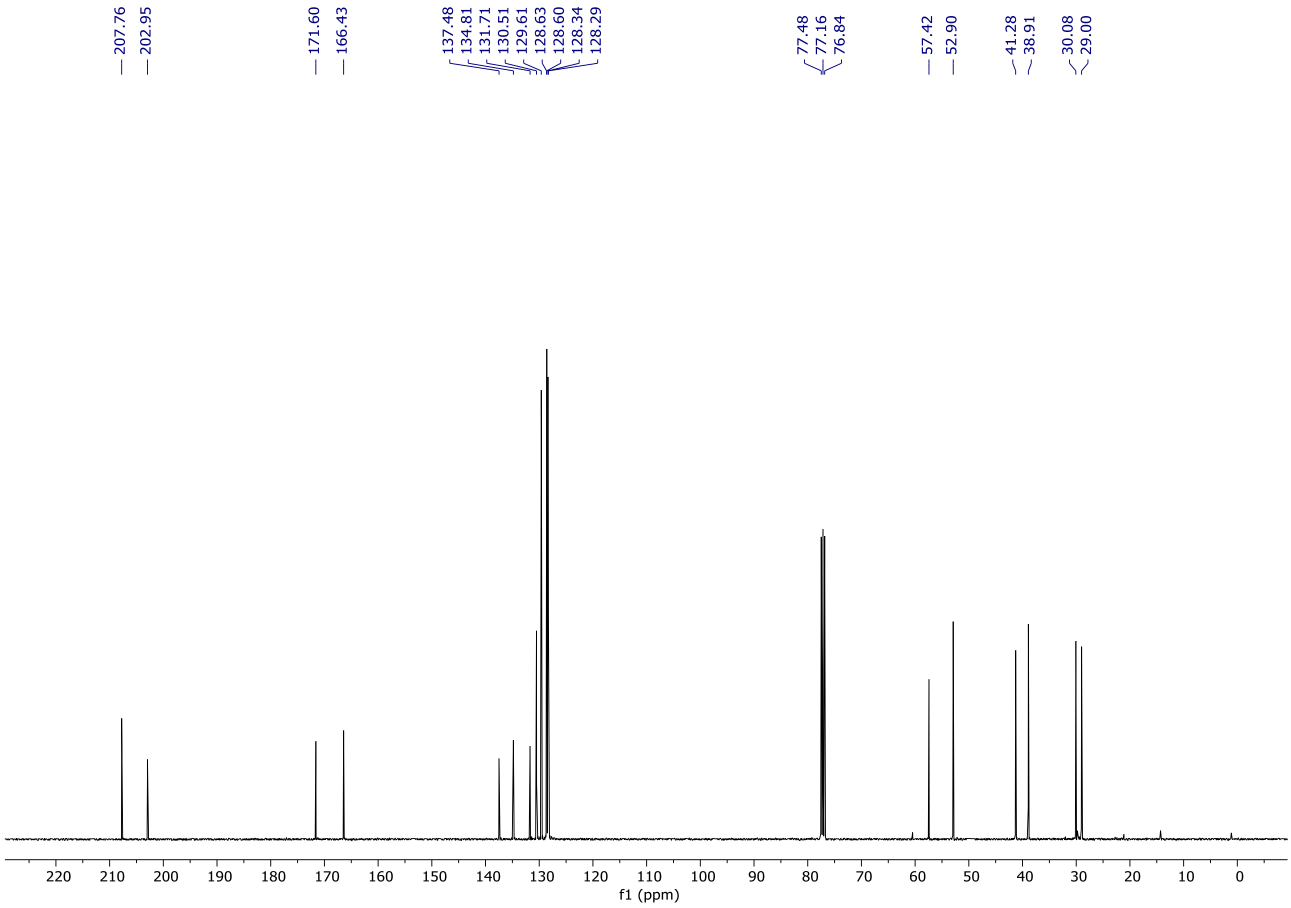

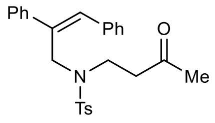
Ph
Ph
O
N
Me
Ts

(*Z*)-**10**

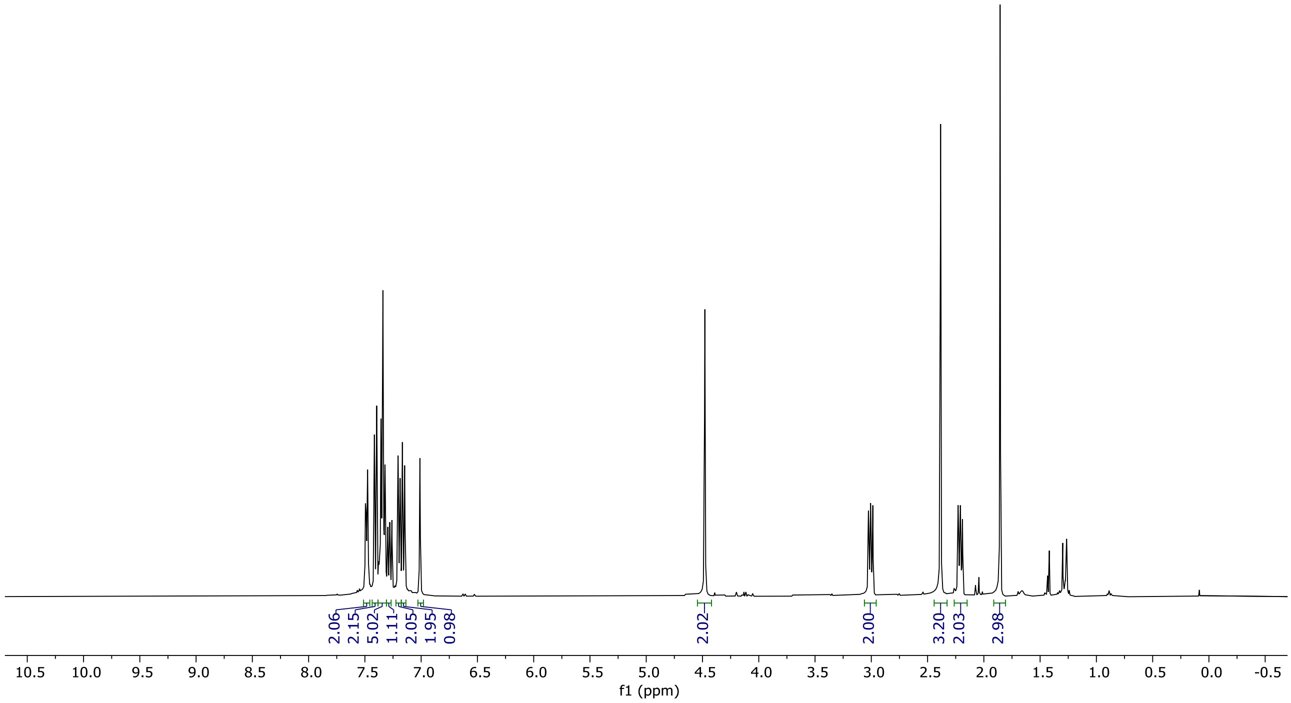
2.06
2.15
5.02
1.11
2.05
1.95
0.98
2.02
2.00
3.20
2.03
2.98
10.5 10.0 9.5 9.0 8.5 8.0 7.5 7.0 6.5 6.0 5.5 5.0 4.5 4.0 3.5 3.0 2.5 2.0 1.5 1.0 0.5 0.0 -0.5
f1 (ppm)

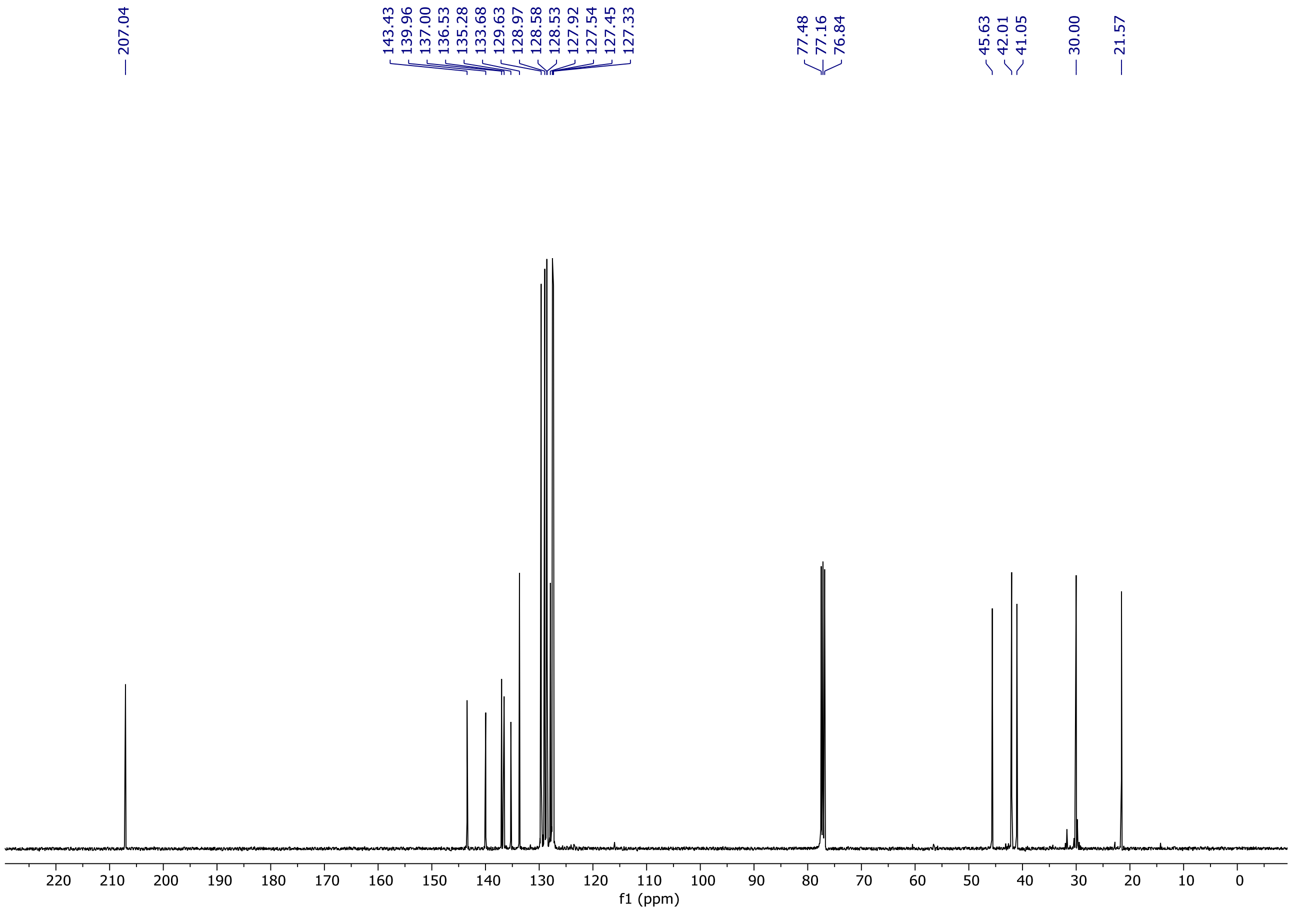
207.04
143.43
139.96
137.00
136.53
135.28
133.68
129.63
128.97
128.58
128.53
127.92
127.54
127.45
127.33
77.48
77.16
76.84
45.63
42.01
41.05
30.00
21.57
220 210 200 190 180 170 160 150 140 130 120 110 100 90 80 70 60 50 40 30 20 10 0
f1 (ppm)

5. HPLC Traces

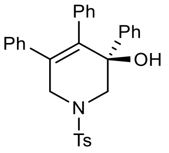

2a

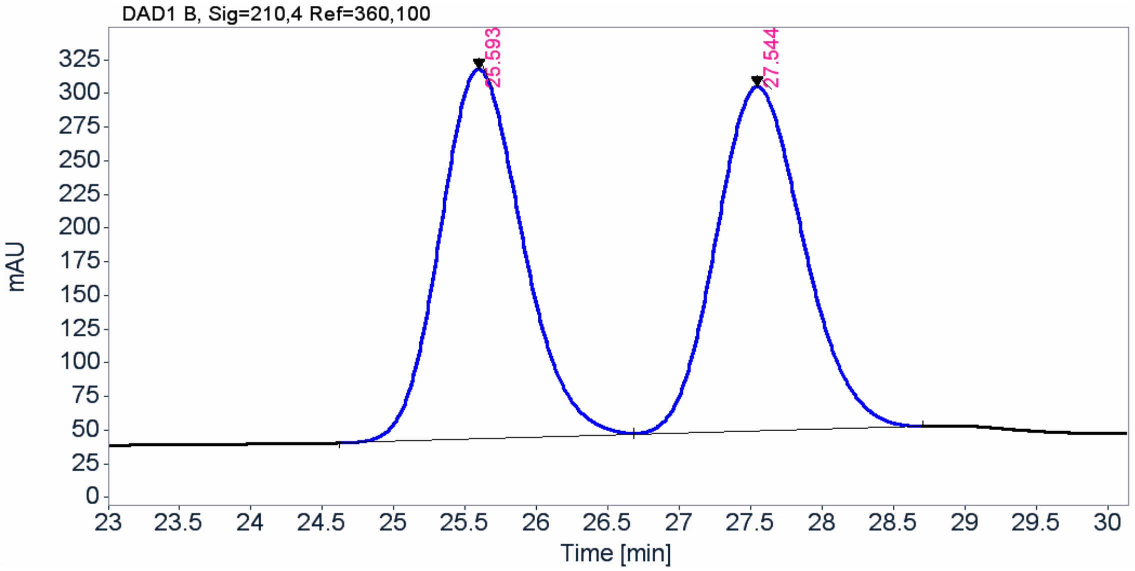

Signal:	DAD1 B, Sig=210,4 Ref=360,100				
RT [min]	Type	Width [min]	Area	Height	Area%
25.593	BV	0.6204	11020.091	274.4660	50.24
27.544	VB	0.6581	10915.397	255.6806	49.76

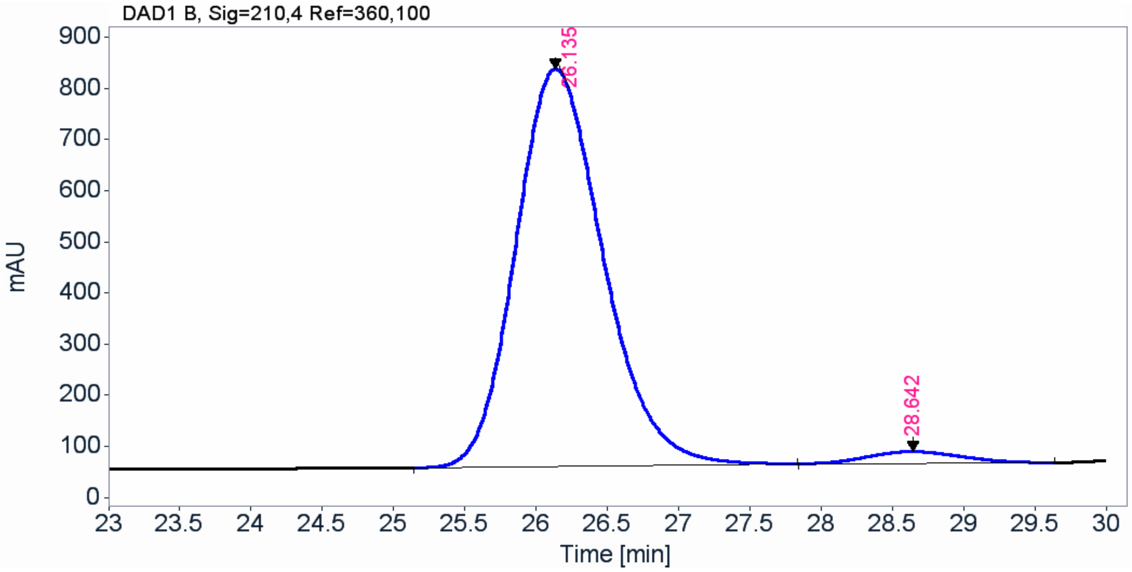

Signal:	DAD1 B, Sig=210,4 Ref=360,100				
RT [min]	Type	Width [min]	Area	Height	Area%
26.135	BB	0.6536	33005.941	776.9605	96.98
28.642	BB	0.5643	1026.198	22.7501	3.02

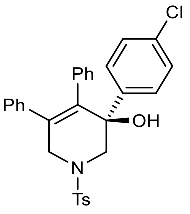

2b

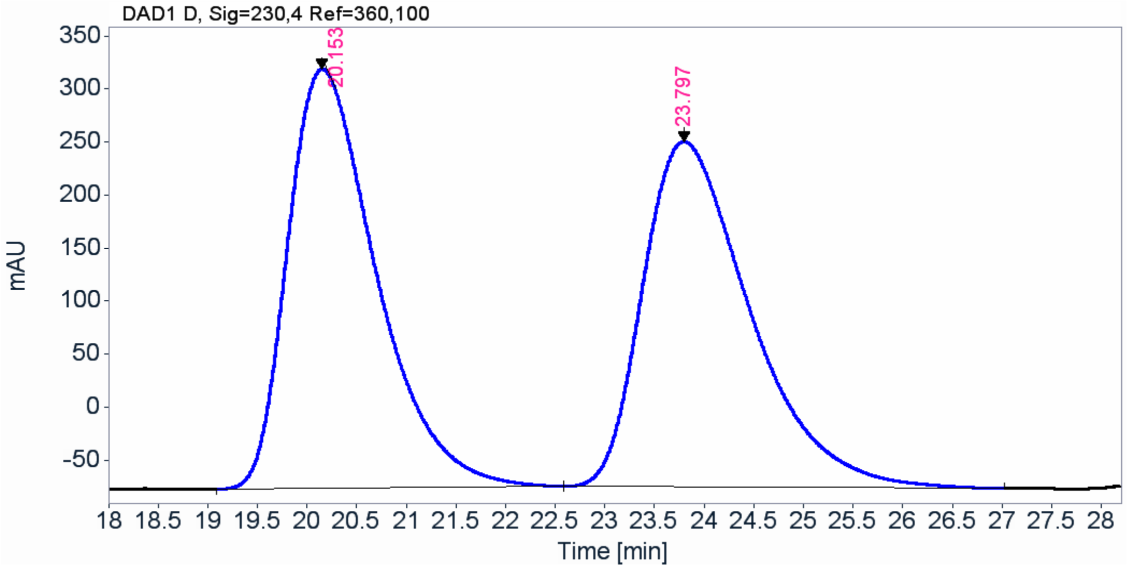

Signal: DAD1 D, Sig=230,4 Ref=360,100

RT [min]	Type	Width [min]	Area	Height	Area%
20.153	BB	0.9494	24513.537	395.3181	50.08
23.797	BB	1.1536	24432.350	325.5518	49.92

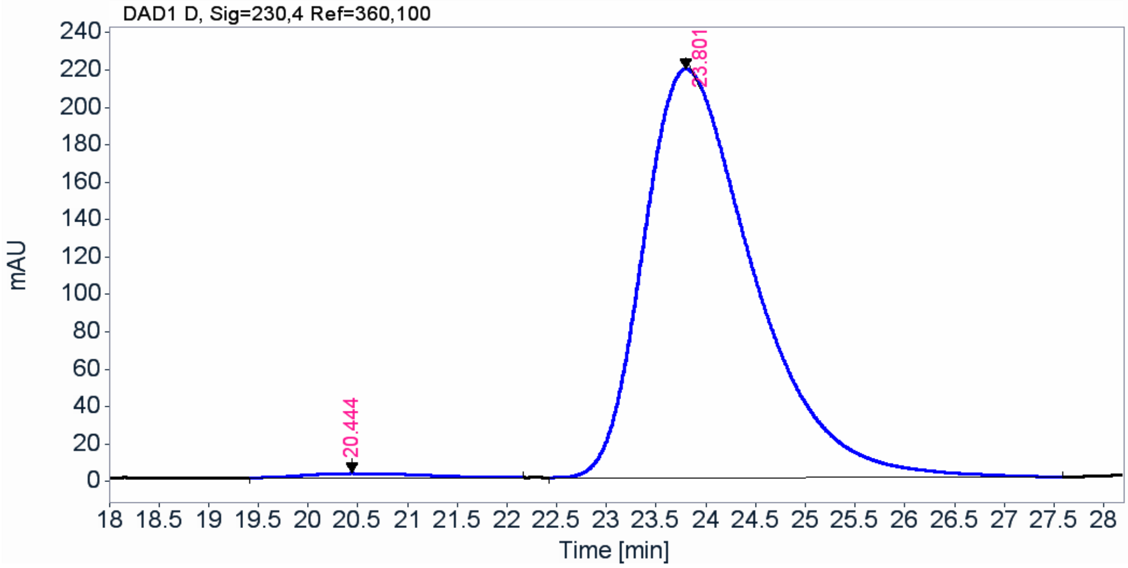

Signal: DAD1 D, Sig=230,4 Ref=360,100

RT [min]	Type	Width [min]	Area	Height	Area%
20.444	MM	1.3494	183.503	2.2664	1.09
23.801	BB	1.1602	16725.088	218.2215	98.91

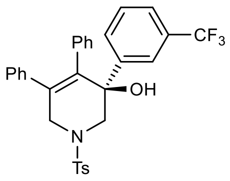

2c

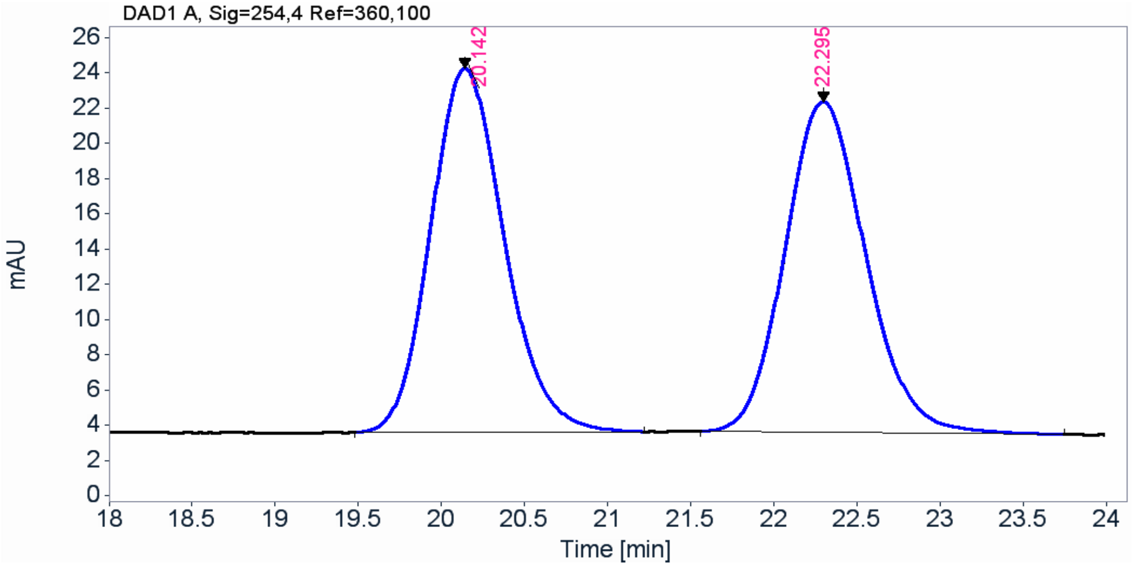

**Signal:**	DAD1 A, Sig=254,4 Ref=360,100				
**RT [min]**	**Type**	**Width [min]**	**Area**	**Height**	**Area%**
20.142	BB	0.4722	637.466	20.6496	50.11
22.295	BB	0.5128	634.616	18.7518	49.89

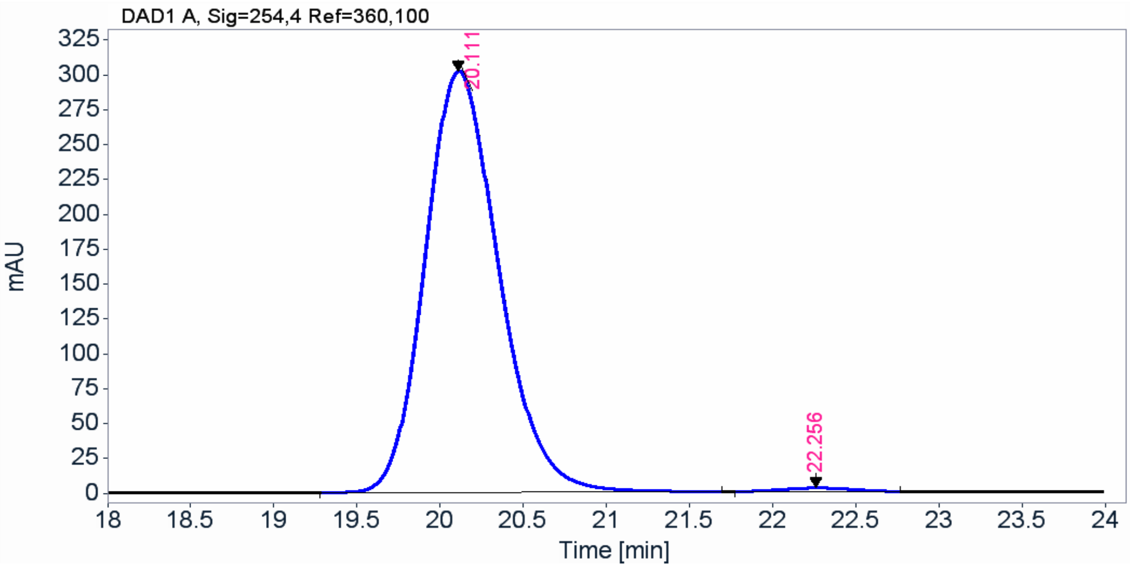

**Signal:**	DAD1 A, Sig=254,4 Ref=360,100				
**RT [min]**	**Type**	**Width [min]**	**Area**	**Height**	**Area%**
20.111	MM	0.5133	9298.565	301.9354	99.16
22.256	MM	0.5019	79.003	2.6235	0.84

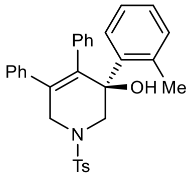

2d

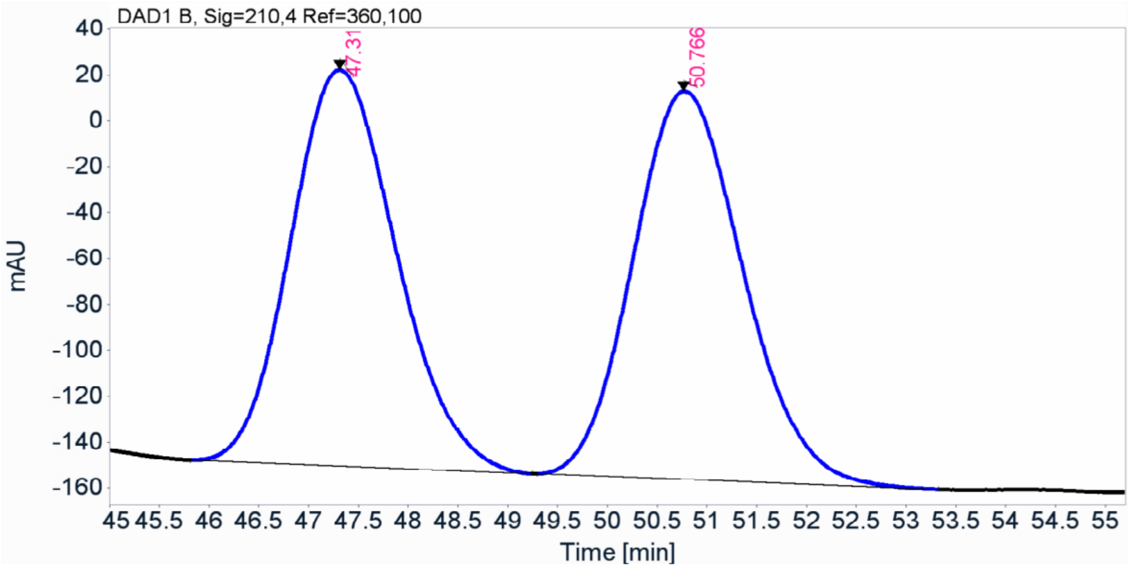

Signal: DAD1 B, Sig=210,4 Ref=360,100

RT [min]	Type	Width [min]	Area	Height	Area%
47.310	BB	1.0803	12749.140	172.4978	49.48
50.766	BB	1.1089	13018.757	168.9646	50.52

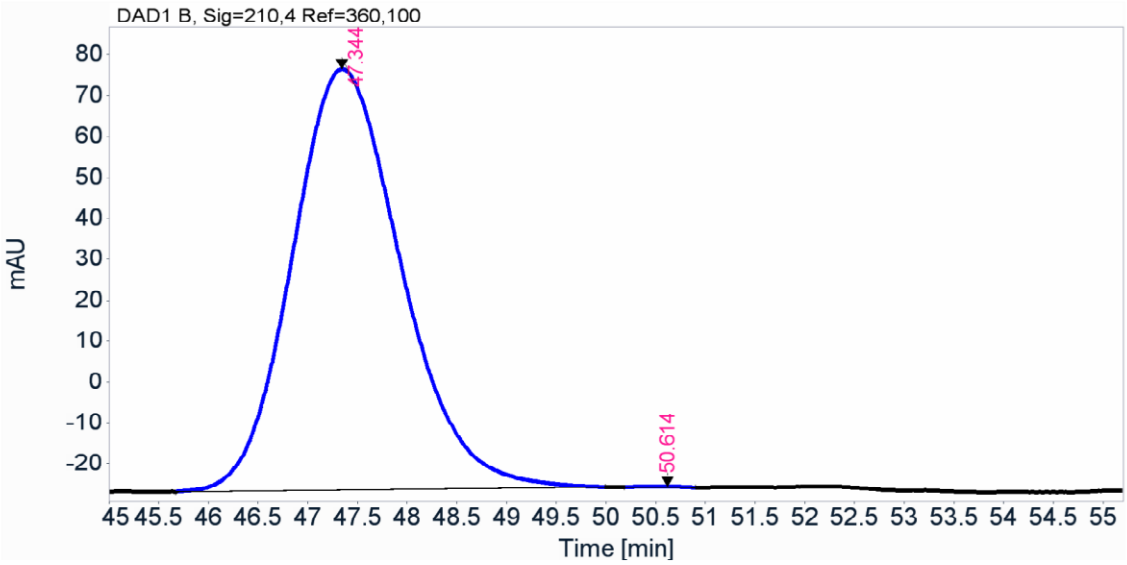

Signal: DAD1 B, Sig=210,4 Ref=360,100

RT [min]	Type	Width [min]	Area	Height	Area%
47.344	BB	1.0816	7832.324	102.7164	99.92
50.614	MM	0.3830	6.370	0.2772	0.08

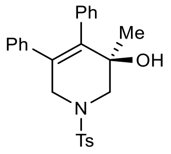

2e

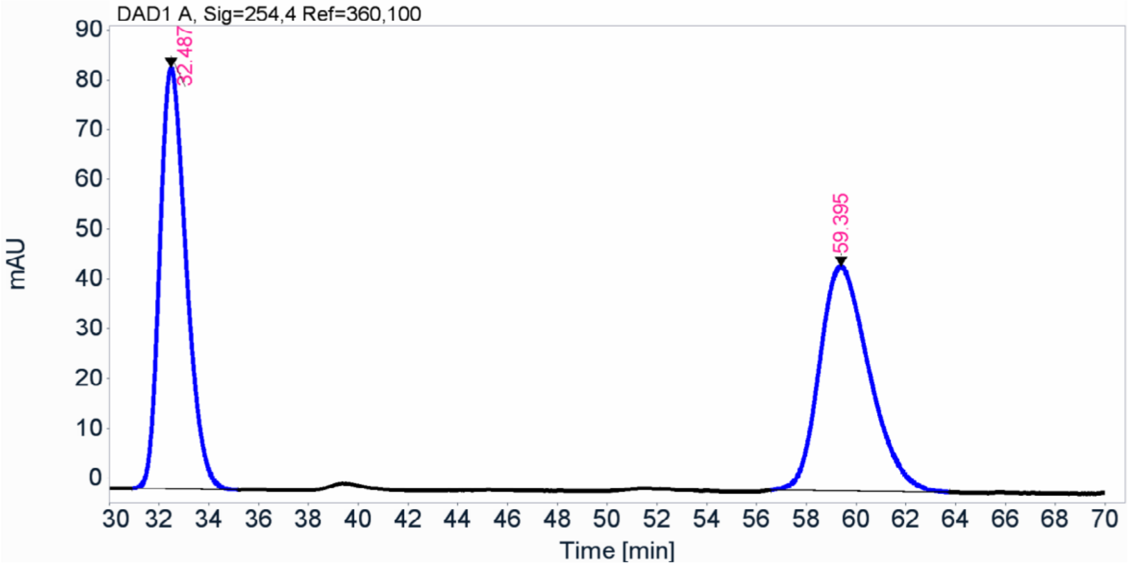

Signal:	DAD1 A, Sig=254,4 Ref=360,100				
RT [min]	Type	Width [min]	Area	Height	Area%
32.487	BB	1.1334	6240.076	84.5102	49.89
59.395	BB	1.7071	6268.154	44.9428	50.11

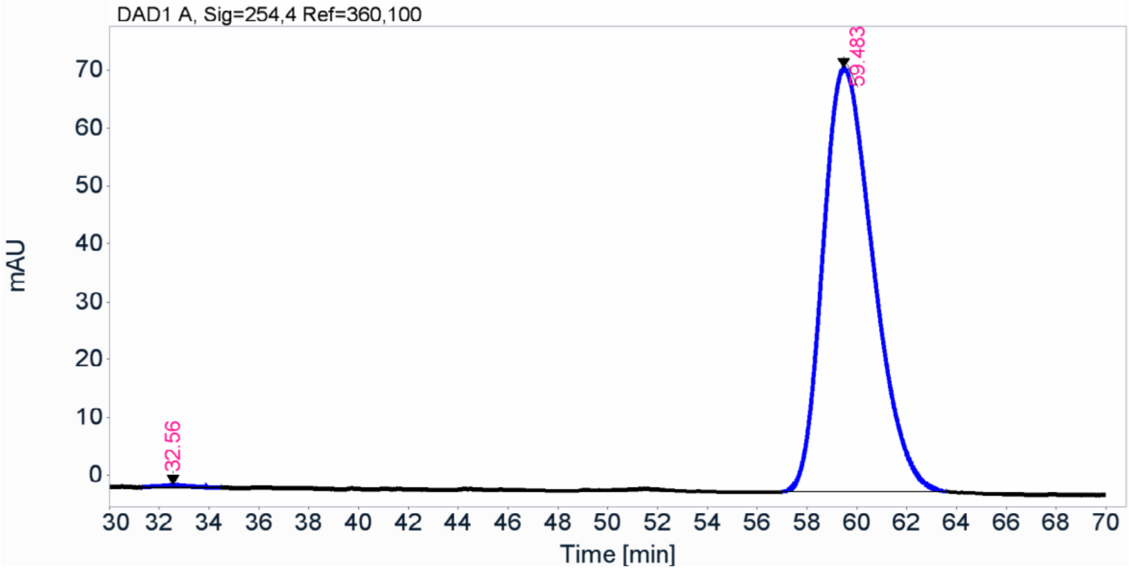

Signal:	DAD1 A, Sig=254,4 Ref=360,100				
RT [min]	Type	Width [min]	Area	Height	Area%
32.560	MM	1.4388	33.221	0.3848	0.32
59.483	BB	1.8837	10214.313	73.1202	99.68

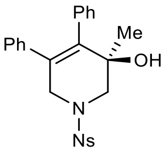

2f

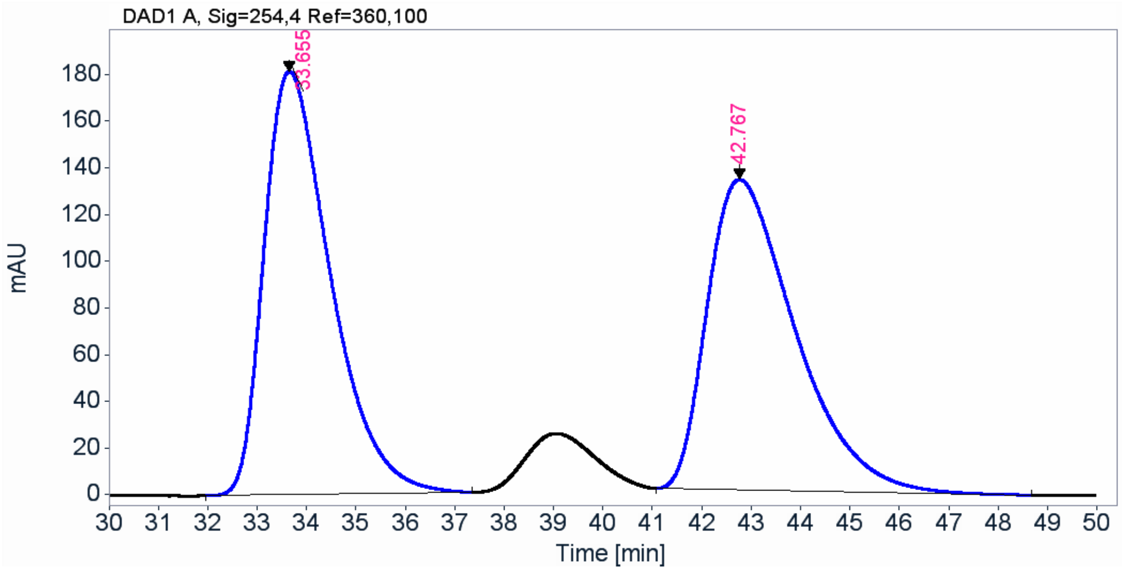

**Signal:**	DAD1 A, Sig=254,4 Ref=360,100				
**RT [min]**	**Type**	**Width [min]**	**Area**	**Height**	**Area%**
33.655	BB	1.4040	16964.090	181.2374	50.74
42.767	BB	1.7872	16471.771	132.9717	49.26

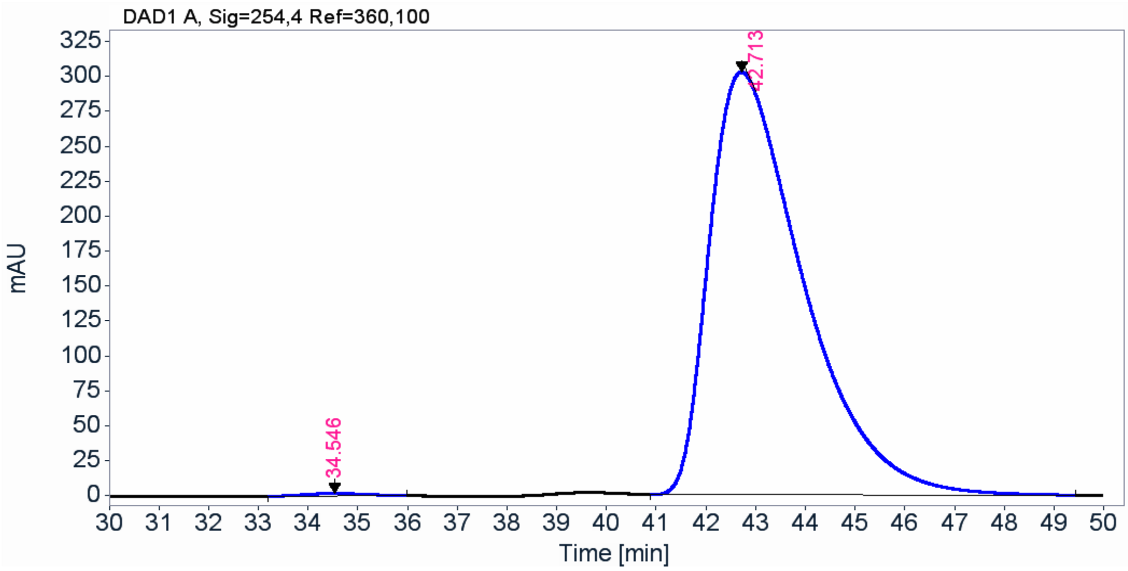

**Signal:**	DAD1 A, Sig=254,4 Ref=360,100				
**RT [min]**	**Type**	**Width [min]**	**Area**	**Height**	**Area%**
34.546	MM	1.4289	163.129	1.9027	0.41
42.713	BB	1.9386	39608.027	302.2963	99.59

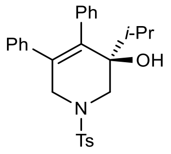

2g

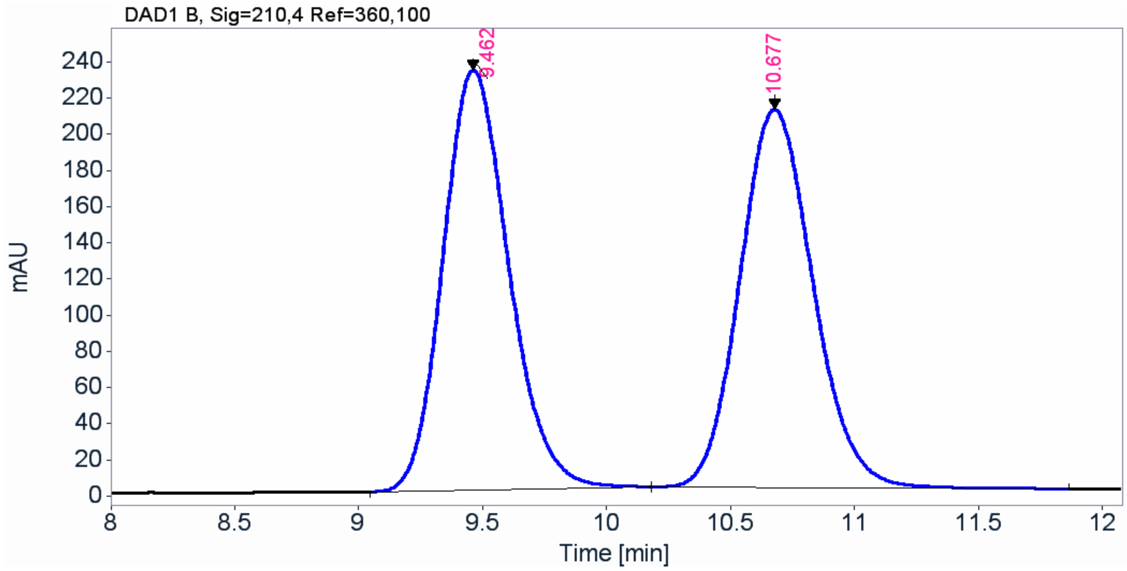

Signal: DAD1 B, Sig=210,4 Ref=360,100

RT [min]	Type	Width [min]	Area	Height	Area%
9.462	BB	0.2851	4279.364	232.0524	49.96
10.677	BB	0.3192	4286.675	209.1031	50.04

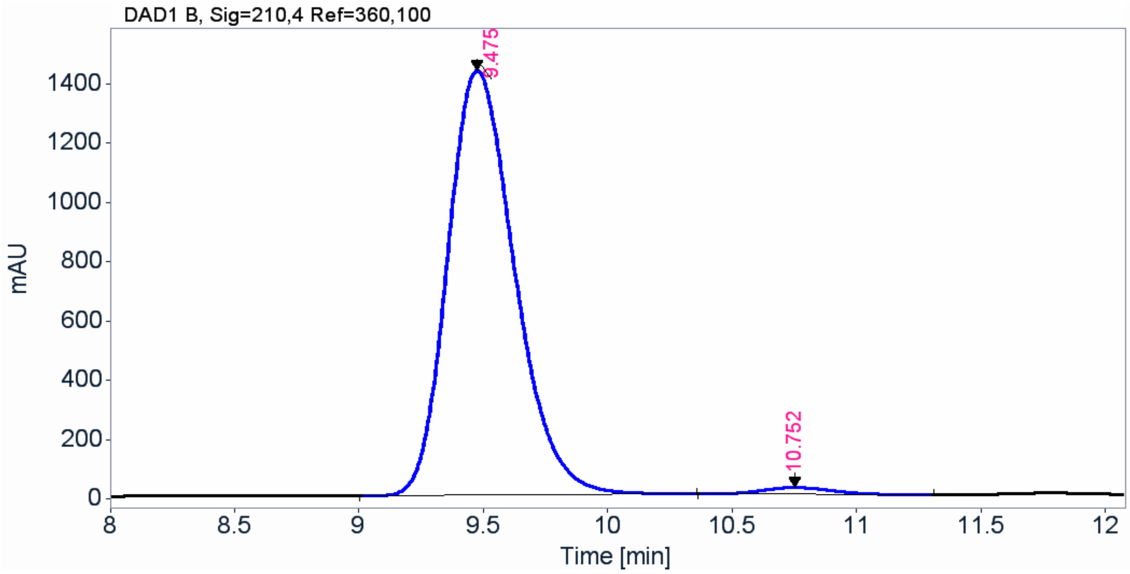

Signal: DAD1 B, Sig=210,4 Ref=360,100

RT [min]	Type	Width [min]	Area	Height	Area%
9.475	BB	0.2902	26776.230	1431.5212	98.33
10.752	BB	0.3040	454.136	22.8348	1.67

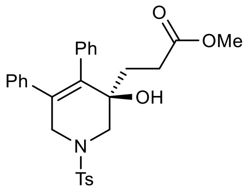

2h

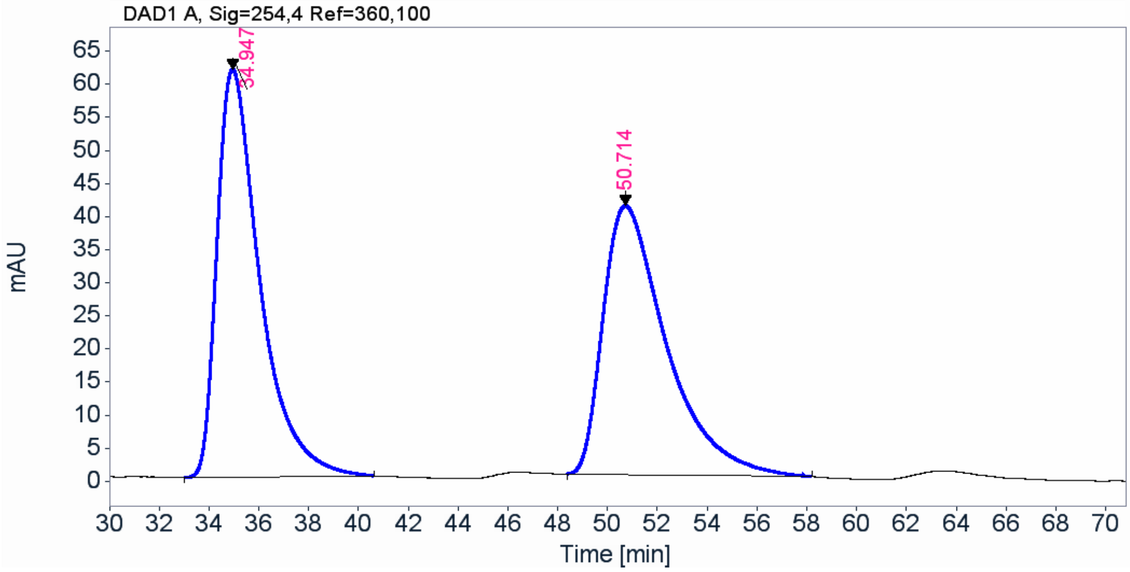

Signal:	DAD1 A, Sig=254,4 Ref=360,100				
RT [min]	Type	Width [min]	Area	Height	Area%
34.947	BB	1.6856	7344.606	61.5735	50.59
50.714	BB	2.0672	7174.683	40.6104	49.41

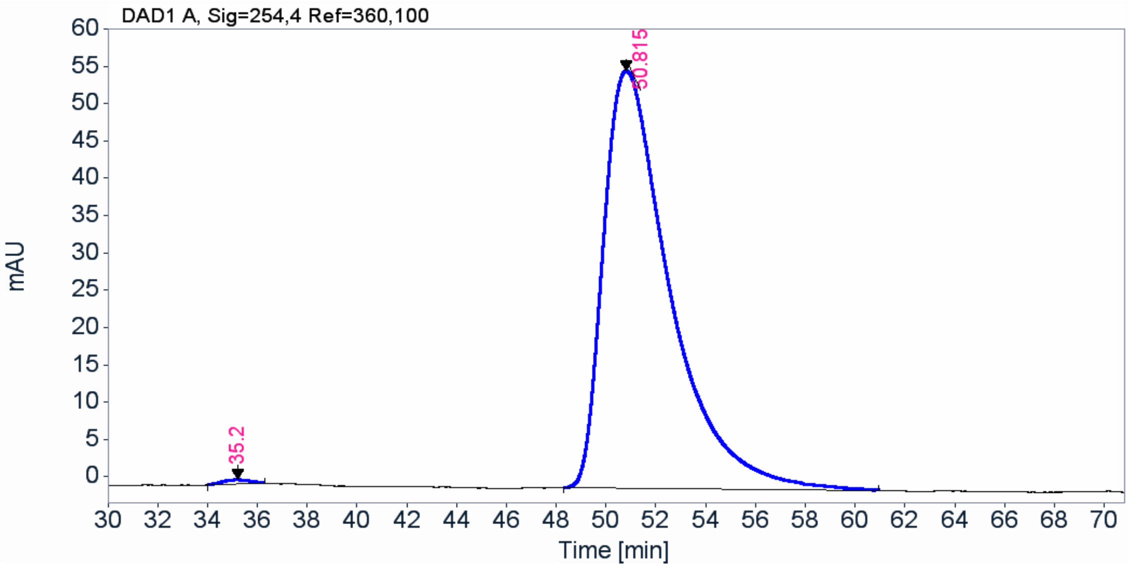

Signal:	DAD1 A, Sig=254,4 Ref=360,100				
RT [min]	Type	Width [min]	Area	Height	Area%
35.200	MM	1.2903	43.601	0.5632	0.42
50.815	MM	3.0890	10367.920	55.9404	99.58

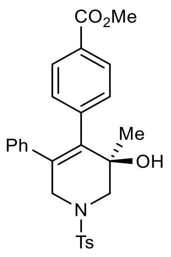

2i

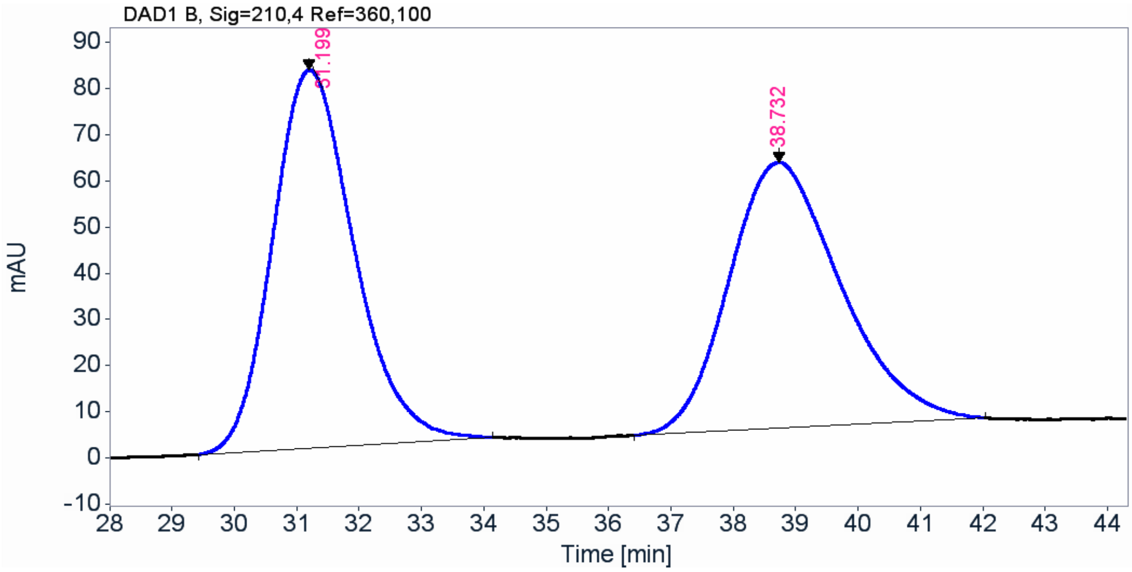

Signal:	DAD1 B, Sig=210,4 Ref=360,100				
RT [min]	Type	Width [min]	Area	Height	Area%
31.199	BB	1.1846	7416.474	81.7339	50.92
38.732	BB	1.4662	7149.200	57.5128	49.08

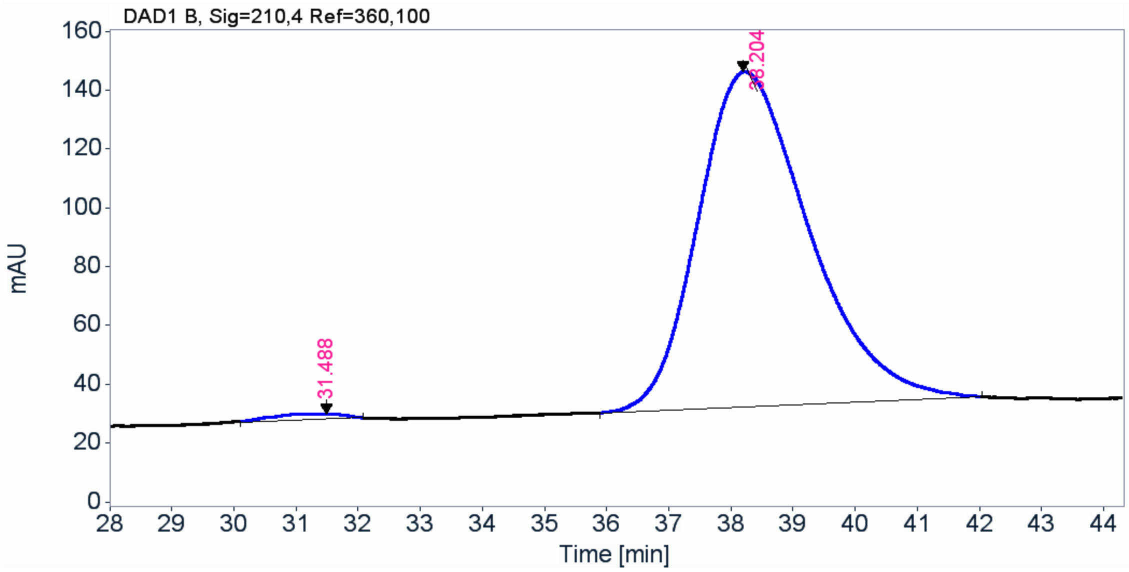

Signal:	DAD1 B, Sig=210,4 Ref=360,100				
RT [min]	Type	Width [min]	Area	Height	Area%
31.488	MM	1.3880	149.218	1.7918	1.05
38.204	BB	1.4630	14128.877	113.9146	98.95

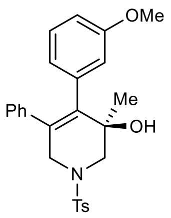

2j

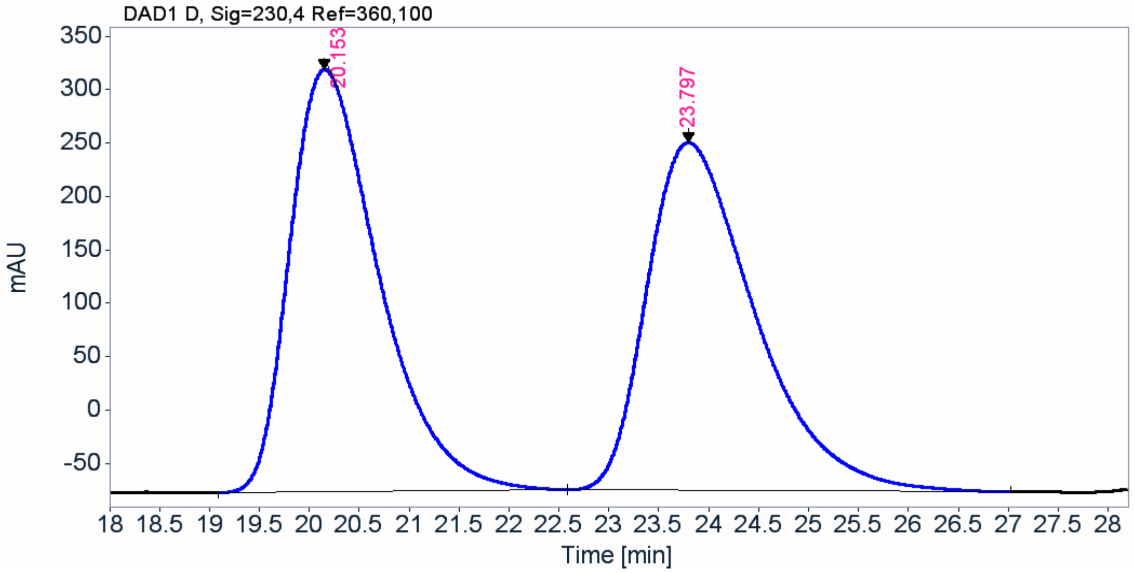

**Signal:**	DAD1 D, Sig=230,4 Ref=360,100				
**RT [min]**	**Type**	**Width [min]**	**Area**	**Height**	**Area%**
20.153	BB	0.9494	24513.537	395.3181	50.08
23.797	BB	1.1536	24432.350	325.5518	49.92

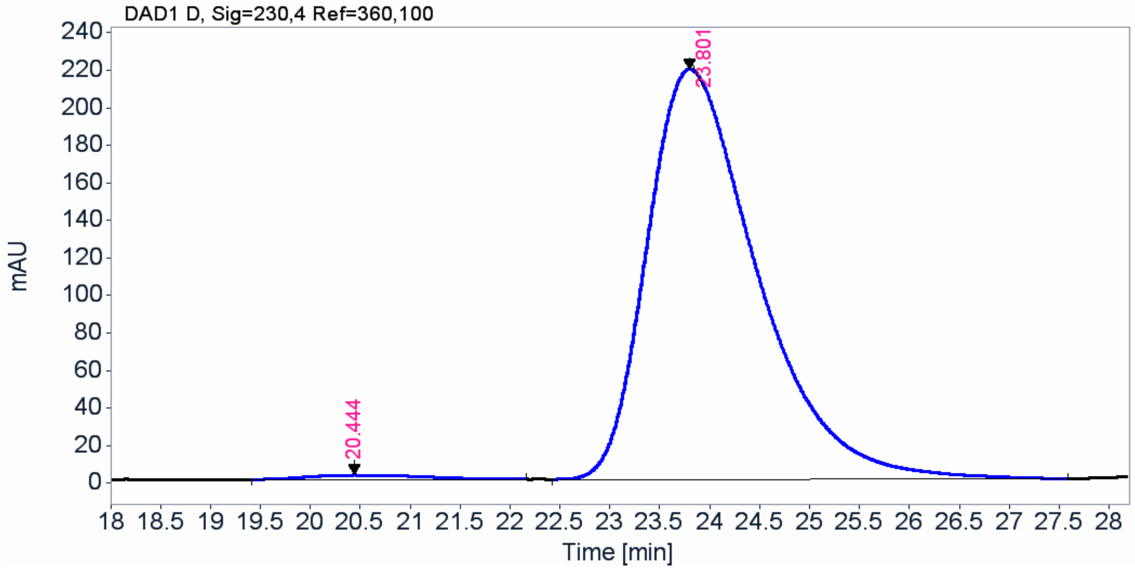

**Signal:**	DAD1 D, Sig=230,4 Ref=360,100				
**RT [min]**	**Type**	**Width [min]**	**Area**	**Height**	**Area%**
20.444	MM	1.3494	183.503	2.2664	1.09
23.801	BB	1.1602	16725.088	218.2215	98.91

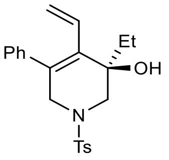

2k

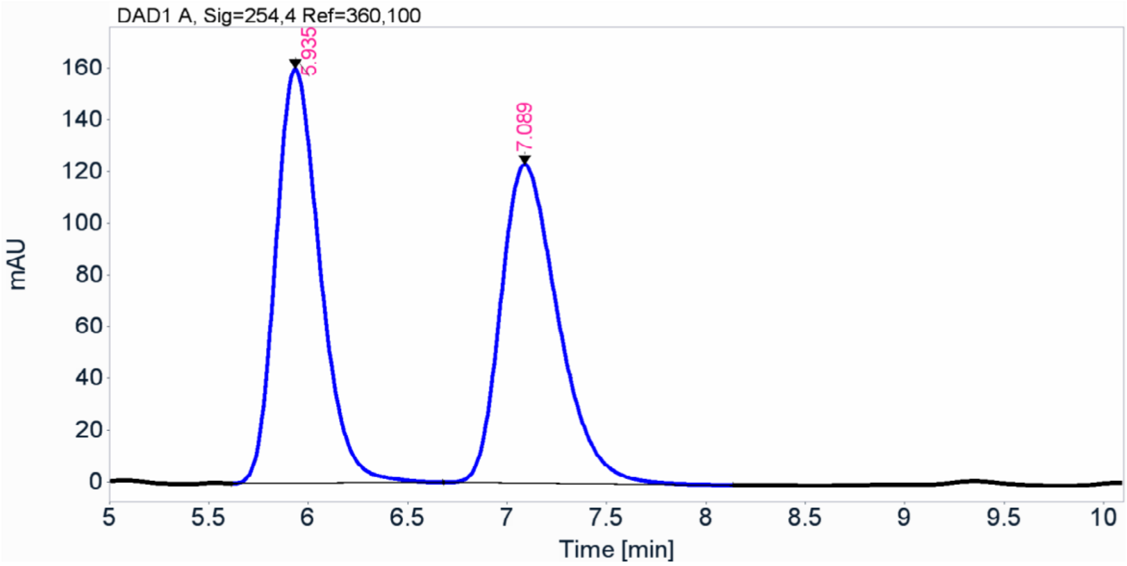

Signal: DAD1 A, Sig=254,4 Ref=360,100

RT [min]	Type	Width [min]	Area	Height	Area%
5.935	BB	0.2388	2479.966	160.0528	50.14
7.089	BB	0.3096	2466.294	123.1569	49.86

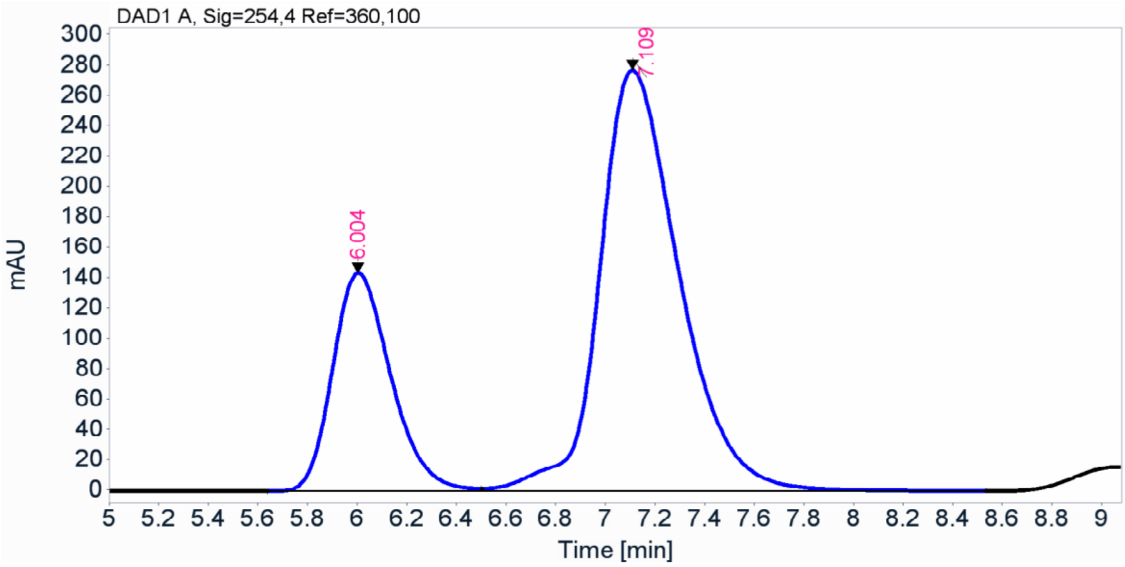

Signal: DAD1 A, Sig=254,4 Ref=360,100

RT [min]	Type	Width [min]	Area	Height	Area%
6.004	BV	0.2522	2345.651	143.8571	27.49
7.109	VB	0.3400	6188.533	277.6907	72.51

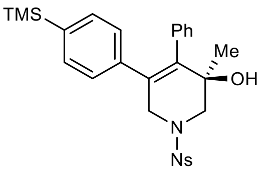

2l

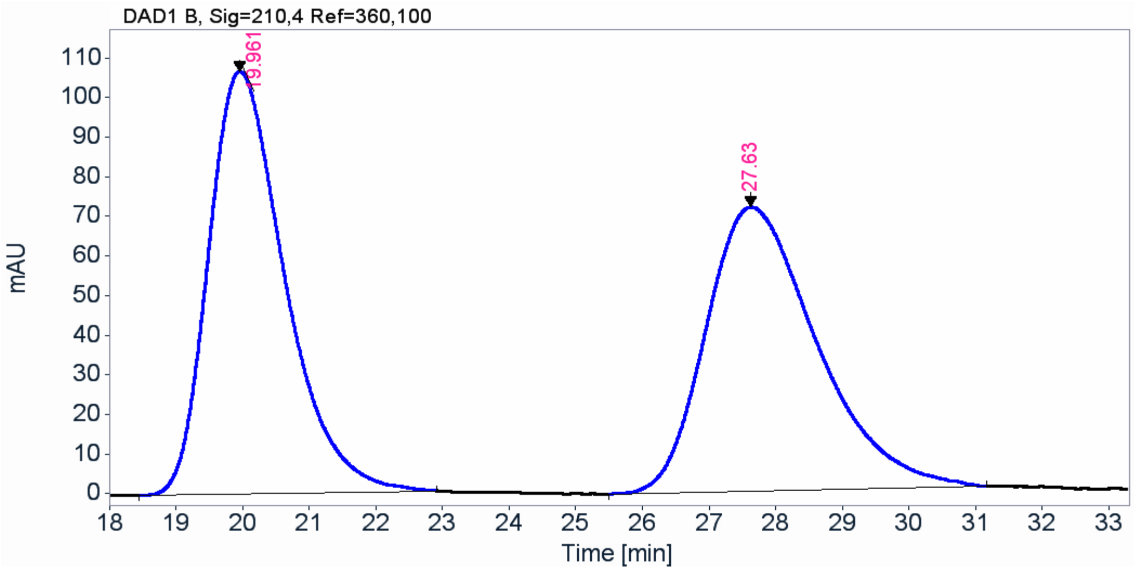

**Signal:**	DAD1 B, Sig=210,4 Ref=360,100				
**RT [min]**	**Type**	**Width [min]**	**Area**	**Height**	**Area%**
19.961	MM	1.3241	8472.582	106.6455	50.50
27.630	MM	1.9290	8305.185	71.7589	49.50

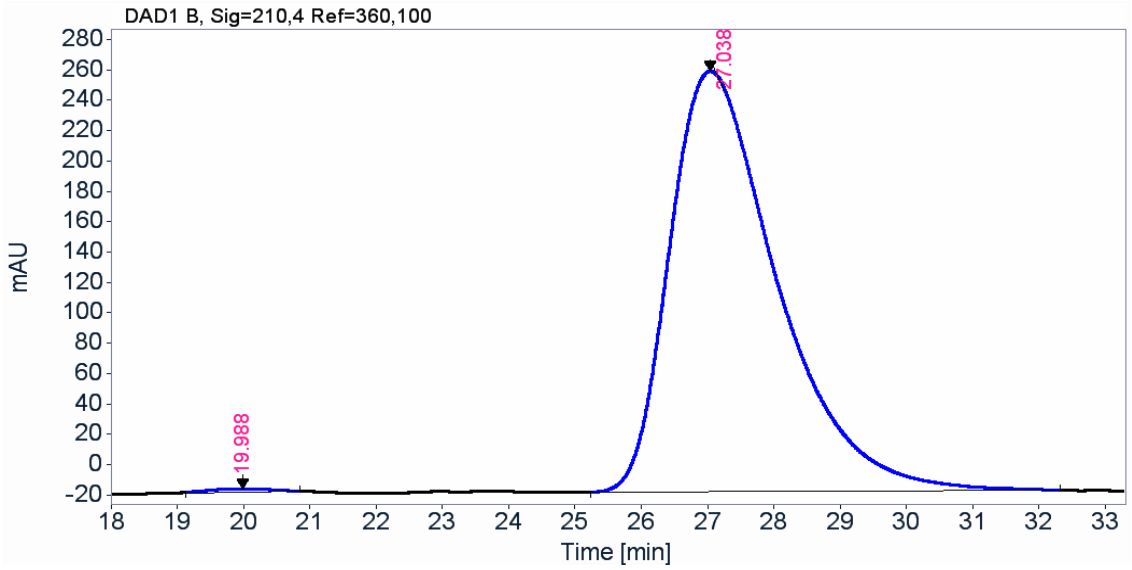

**Signal:**	DAD1 B, Sig=210,4 Ref=360,100				
**RT [min]**	**Type**	**Width [min]**	**Area**	**Height**	**Area%**
19.988	MM	1.0768	152.418	2.3591	0.49
27.038	BB	1.6171	31235.373	277.0887	99.51

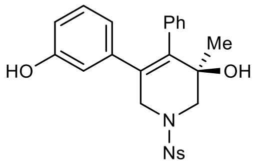

2m

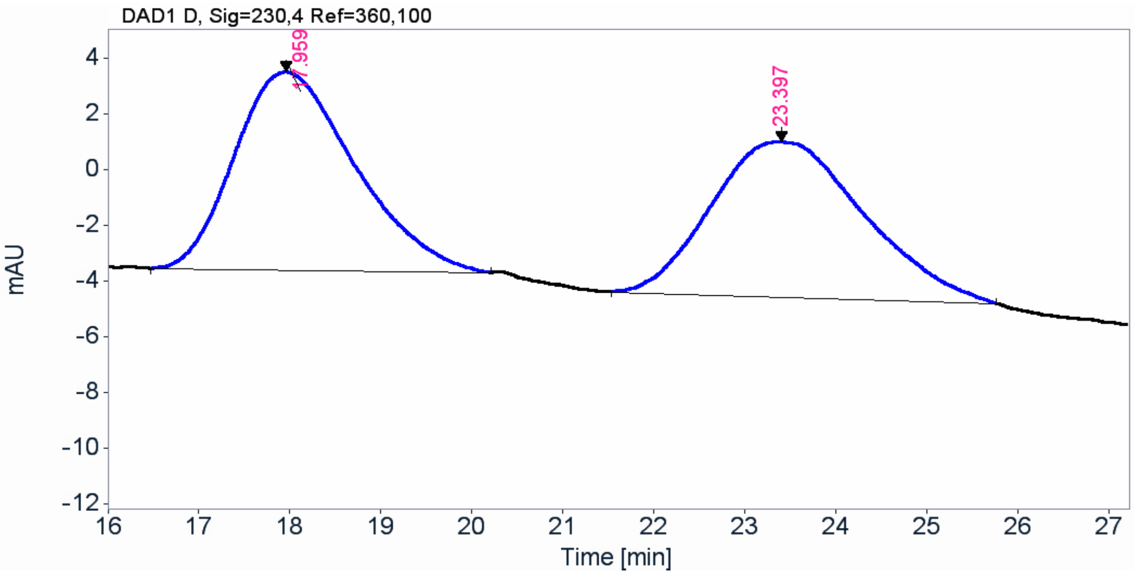

**Signal:**	DAD1 D, Sig=230,4 Ref=360,100				
**RT [min]**	**Type**	**Width [min]**	**Area**	**Height**	**Area%**
17.959	BB	1.0811	650.824	7.1288	49.98
23.397	MM	1.9390	651.388	5.5991	50.02

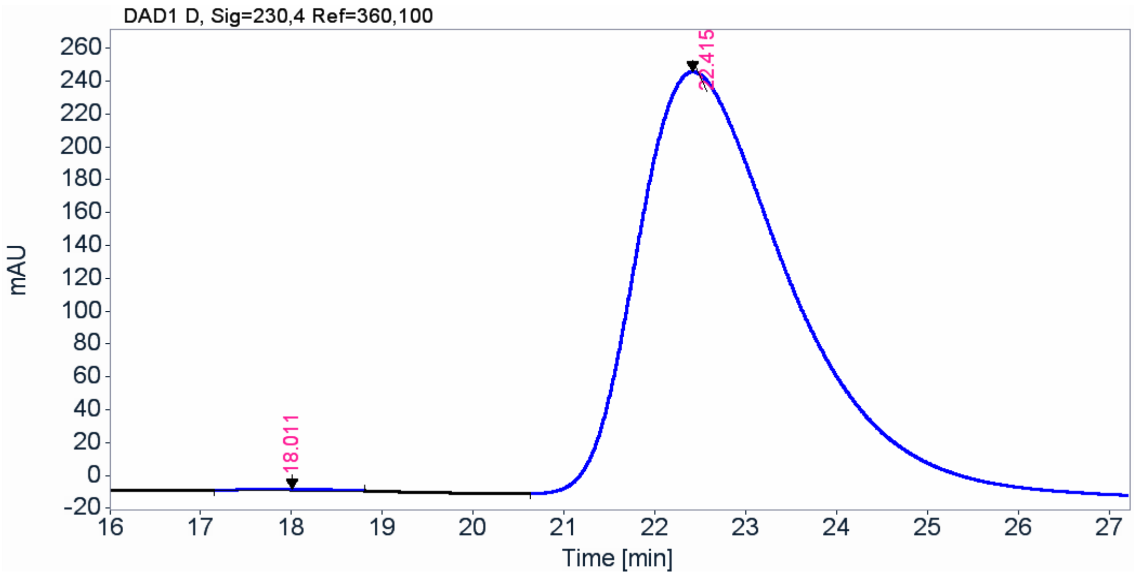

**Signal:**	DAD1 D, Sig=230,4 Ref=360,100				
**RT [min]**	**Type**	**Width [min]**	**Area**	**Height**	**Area%**
18.011	MM	1.0118	61.034	1.0053	0.20
22.415	BBA	1.6925	30490.006	257.6705	99.80

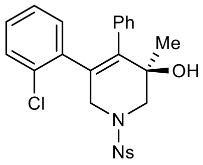

2n

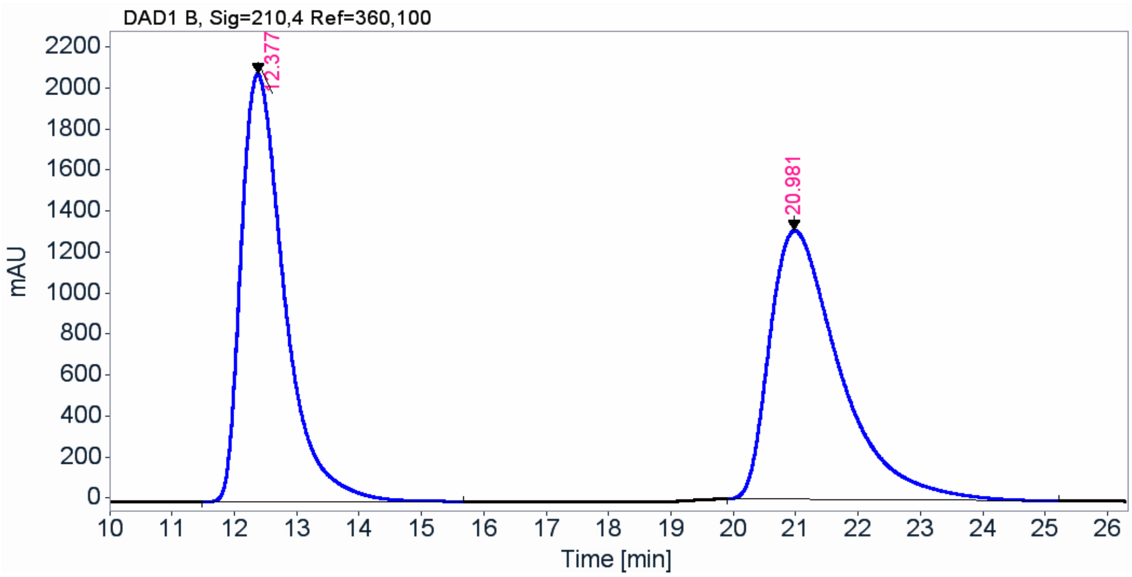

**Signal:**	DAD1 B, Sig=210,4 Ref=360,100				
**RT [min]**	**Type**	**Width [min]**	**Area**	**Height**	**Area%**
12.377	BB	0.5944	100258.352	2087.3564	49.41
20.981	MM	1.3045	102664.820	1311.7223	50.59

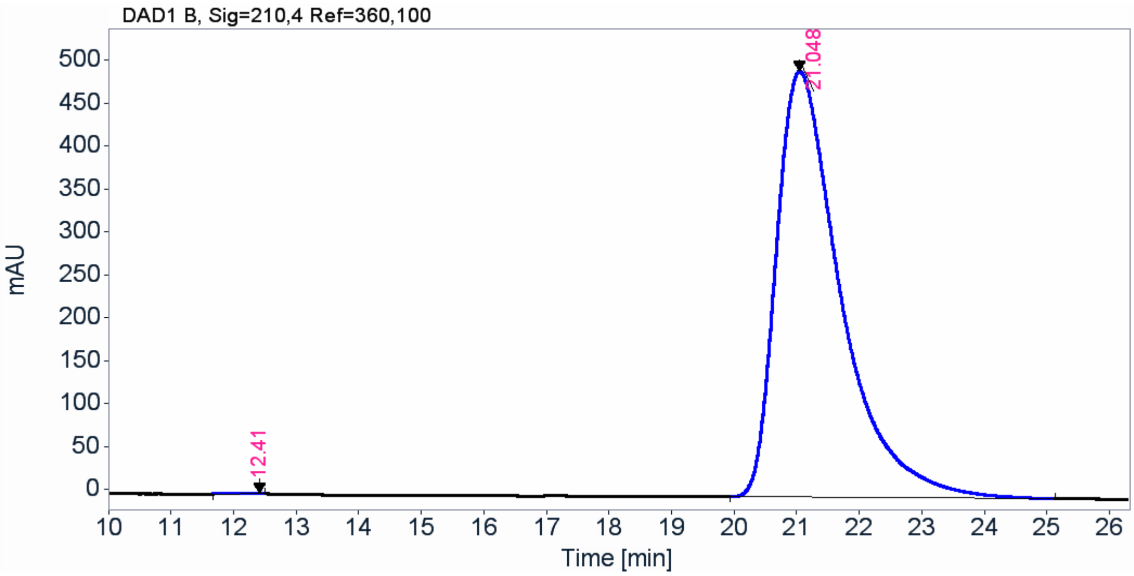

**Signal:**	DAD1 B, Sig=210,4 Ref=360,100				
**RT [min]**	**Type**	**Width [min]**	**Area**	**Height**	**Area%**
12.410	MM	0.5341	11.412	0.3561	0.03
21.048	BB	1.0415	35410.441	495.6667	99.97

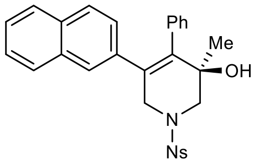

2o

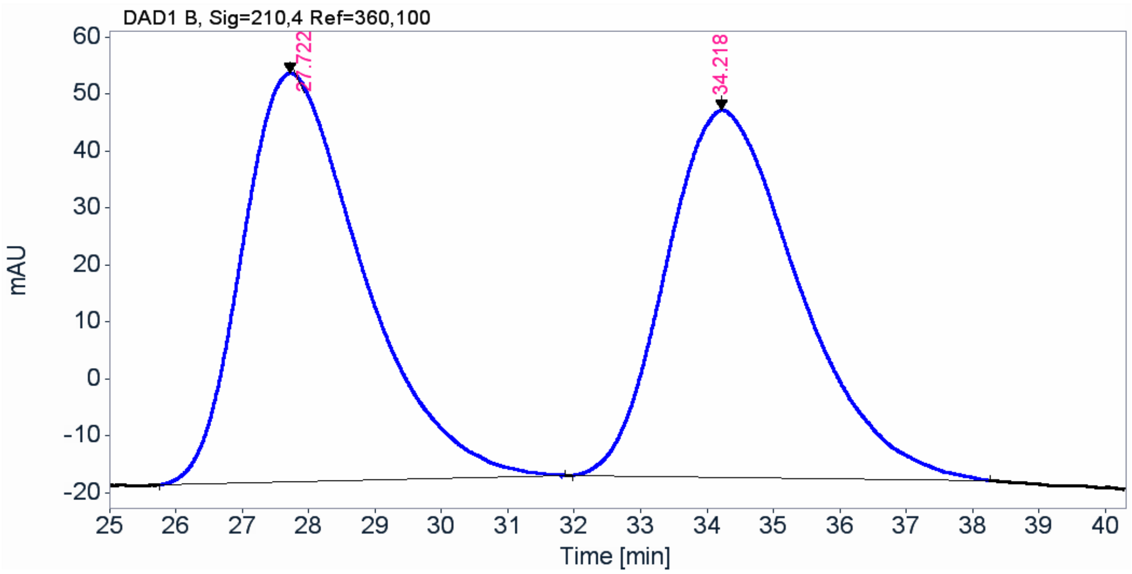

**Signal:**	DAD1 B, Sig=210,4 Ref=360,100				
**RT [min]**	**Type**	**Width [min]**	**Area**	**Height**	**Area%**
27.722	BB	1.5031	9001.102	71.6746	50.19
34.218	BB	1.6359	8932.927	64.4152	49.81

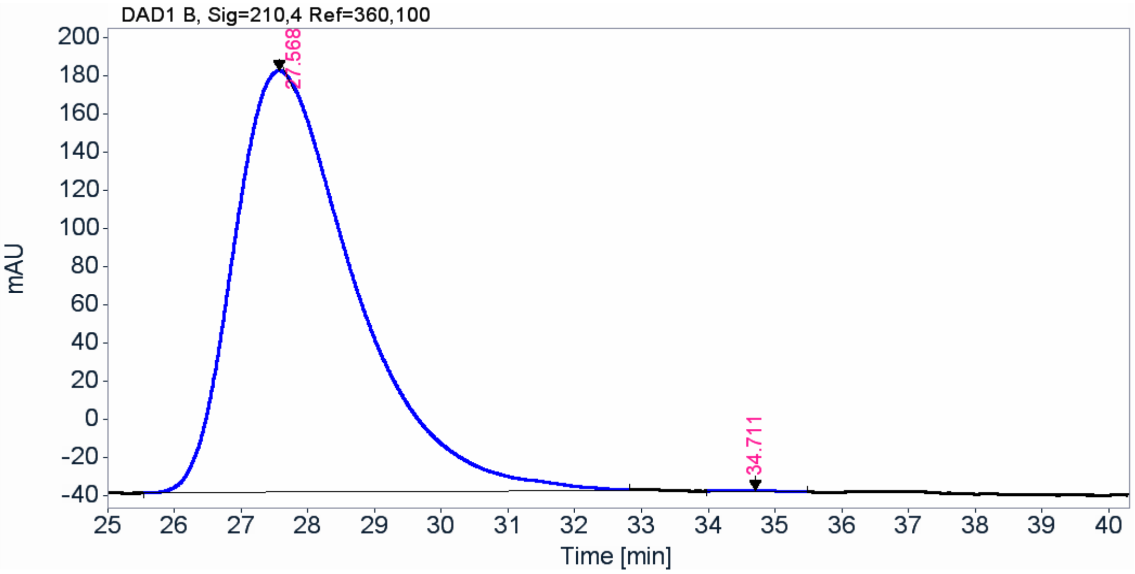

**Signal:**	DAD1 B, Sig=210,4 Ref=360,100				
**RT [min]**	**Type**	**Width [min]**	**Area**	**Height**	**Area%**
27.568	MM	2.0927	27740.098	220.9328	99.88
34.711	MM	0.9889	33.487	0.5644	0.12

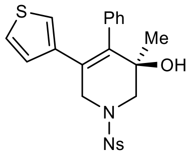

2p

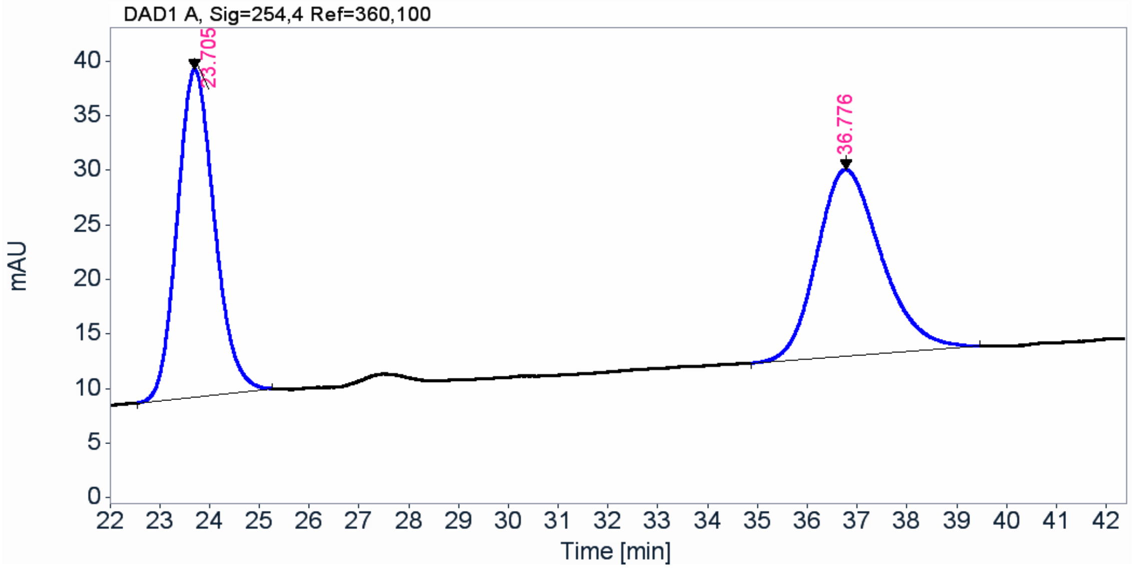

Signal: DAD1 A, Sig=254,4 Ref=360,100

RT [min]	Type	Width [min]	Area	Height	Area%
23.705	BB	0.7847	1555.172	30.0020	50.31
36.776	BB	1.0773	1535.826	17.1092	49.69

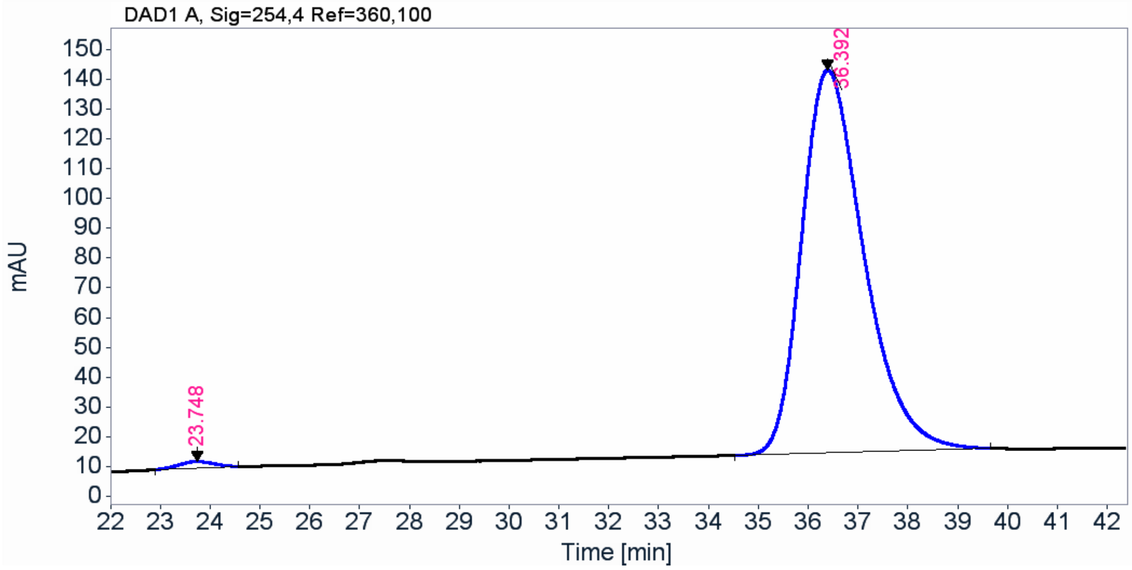

Signal: DAD1 A, Sig=254,4 Ref=360,100

RT [min]	Type	Width [min]	Area	Height	Area%
23.748	MM	0.5823	109.406	2.2200	0.97
36.392	BB	1.2919	11149.593	128.5547	99.03

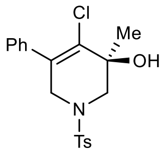

2q

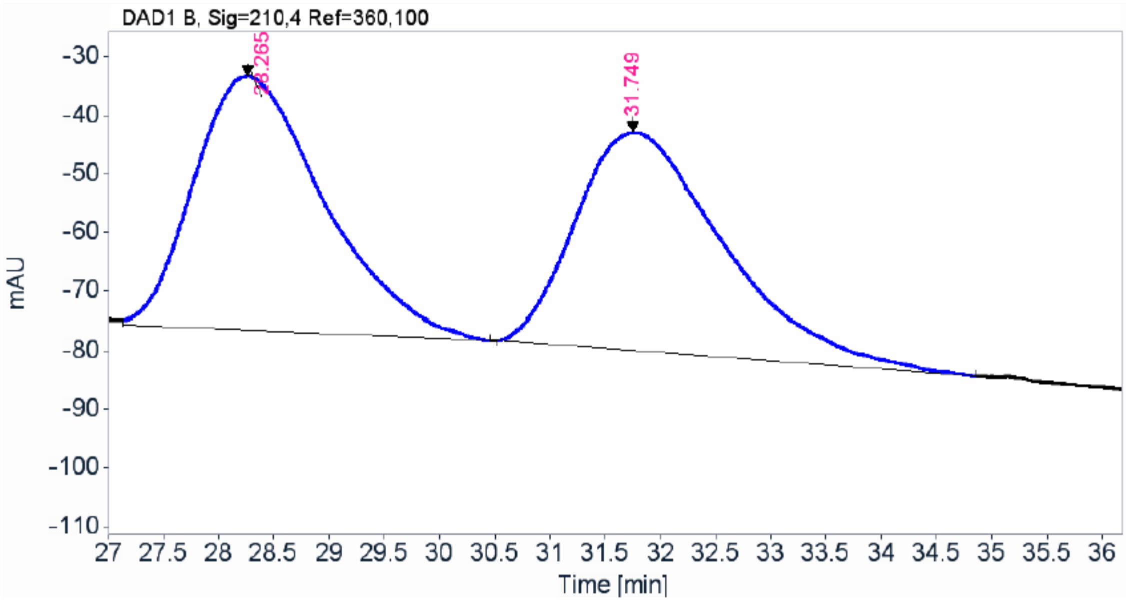

Signal: DAD1 B, Sig=210,4 Ref=360,100

RT [min]	Type	Width [min]	Area	Height	Area%
28.265	VB	0.9631	3511.032	43.2482	50.48
31.749	BB	1.0998	3443.906	37.0045	49.52

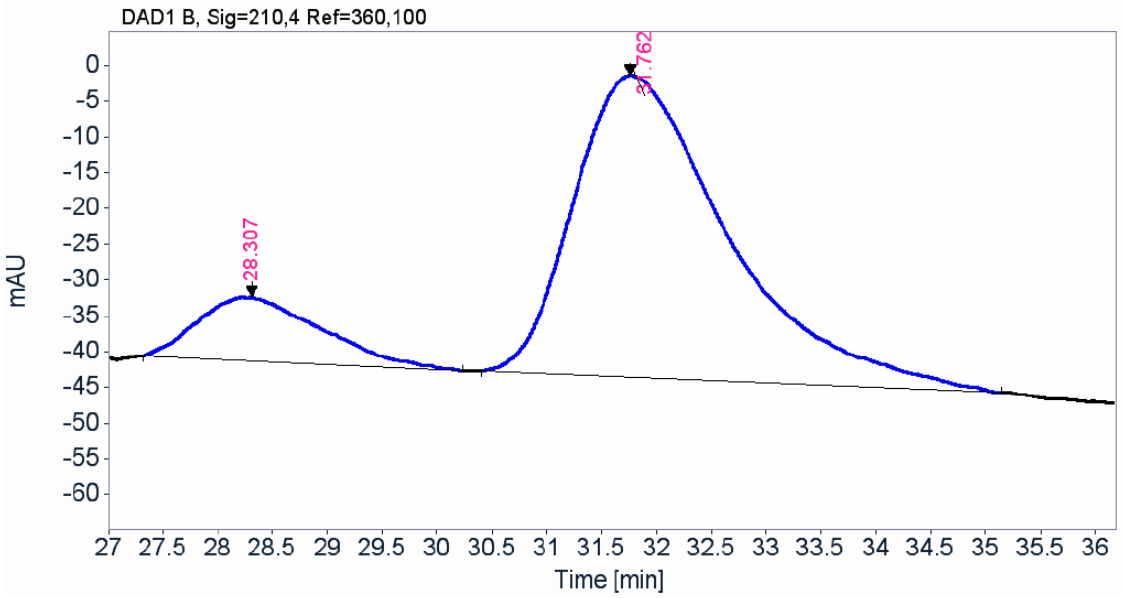

Signal: DAD1 B, Sig=210,4 Ref=360,100

RT [min]	Type	Width [min]	Area	Height	Area%
28.307	BB	0.9222	695.530	8.9155	14.46
31.762	BB	1.1745	4115.999	42.2530	85.54

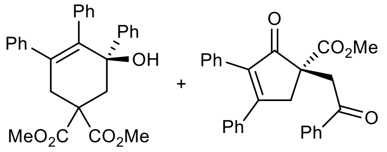

5 **6a**

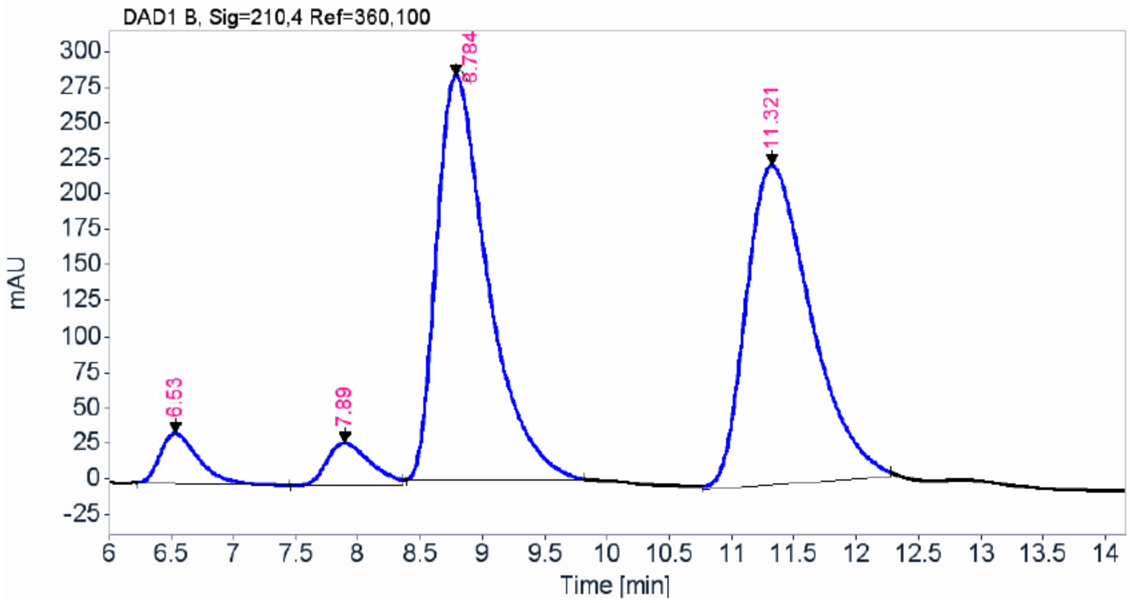

Signal:	DAD1 B, Sig=210,4 Ref=360,100				
RT [min]	Type	Width [min]	Area	Height	Area%
6.530	BB	0.3340	748.398	34.9250	4.22
7.890	BV	0.3673	732.597	29.9604	4.13
8.784	MM	0.4774	8135.735	284.0522	45.92
11.321	MM	0.6033	8101.574	223.8213	45.72

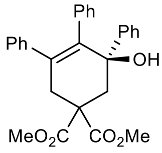

5

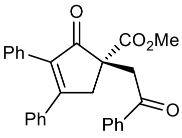

6a

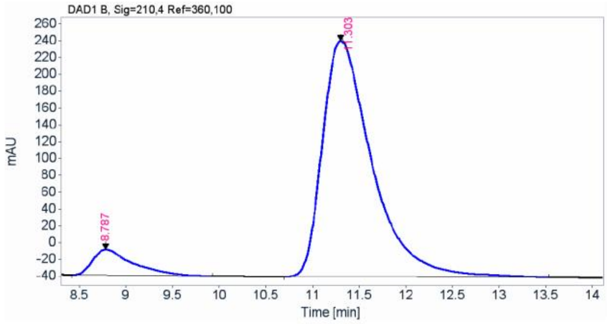

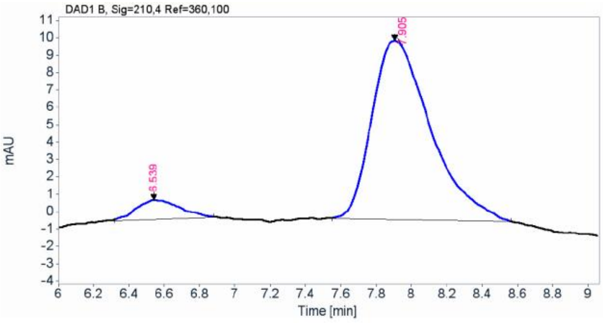

Signal:	DAD1 B, Sig=210,4 Ref=360,100				
RT [min]	Type	Width [min]	Area	Height	Area%
8.787	BB	0.4326	902.505	30.5548	8.03
11.303	BB	0.5583	10336.960	280.2965	91.97

Signal:	DAD1 B, Sig=210,4 Ref=360,100				
RT [min]	Type	Width [min]	Area	Height	Area%
6.539	MM	0.2890	19.297	1.1128	7.51
7.905	MM	0.3840	237.598	10.3115	92.49

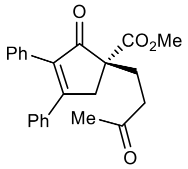

6b
(tentative assignment
of absolute configuration)

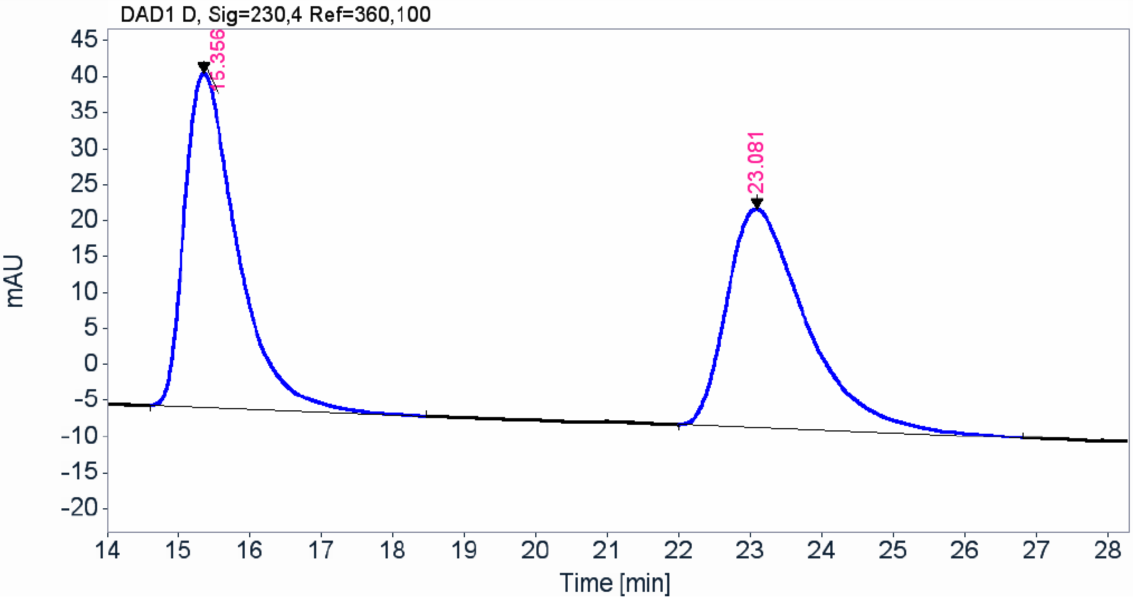

Signal: DAD1 D, Sig=230,4 Ref=360,100

RT [min]	Type	Width [min]	Area	Height	Area%
15.356	BB	0.7578	2373.339	46.4757	50.23
23.081	BB	0.9218	2351.302	30.3523	49.77

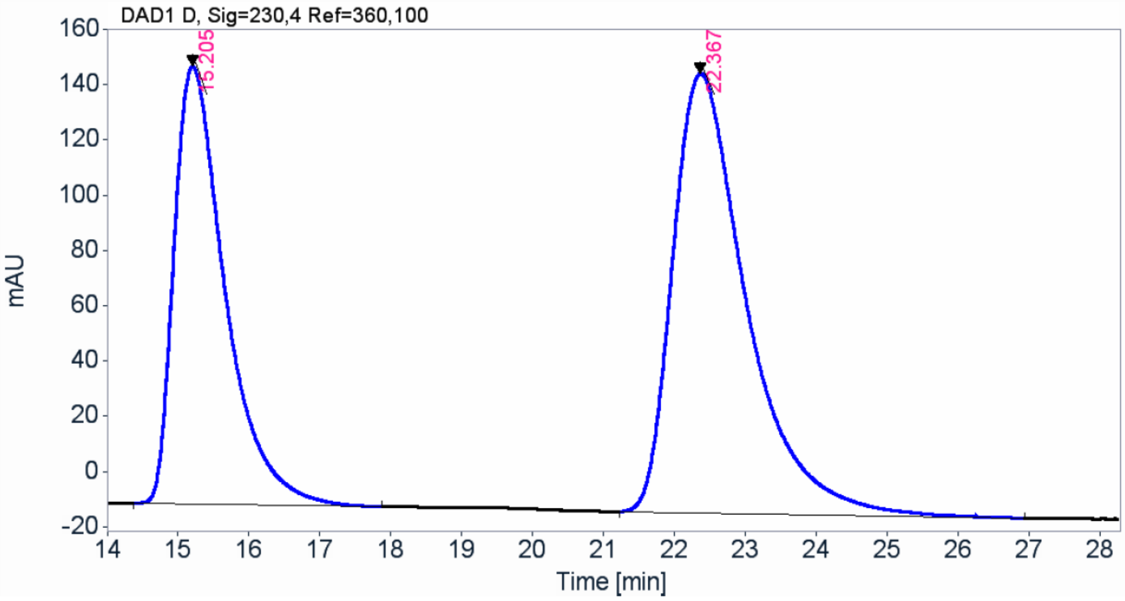

Signal: DAD1 D, Sig=230,4 Ref=360,100

RT [min]	Type	Width [min]	Area	Height	Area%
15.205	BB	0.7568	7905.807	158.2562	40.74
22.367	BB	1.0885	11500.991	158.8771	59.26

6. References

1. M. G. Schrems, A. Pfaltz, *Chem. Commun.* **2009**, 6210-6212.
2. S. M. Gillbard, C.-H. Chung, S. N. Karad, H. Panchal, W. Lewis, H. W. Lam, *Chem. Commun.* **2018**, *54*, 11769-11772.
3. K. R. Strom, A. C. Impastato, K. J. Moy, A. J. Landreth, J. K. Snyder, *Org. Lett.* **2015**, *17*, 2126-2129.
4. T. Nishimura, Y. Maeda, T. Hayashi, *Org. Lett.* **2011**, *13*, 3674-3677.
5. B. DeBoef, W. R. Counts, S. R. Gilbertson, *J. Org. Chem.* **2007**, *72*, 799-804.
6. K. Shen, X. Han, X. Lu, *Org. Lett.* **2013**, *15*, 1732-1735.
7. N. M. Groome, E. E. Elboray, M. W. Inman, H. A. Dondas, R. M. Phillips, C. Kilner, R. Grigg, *Chem. Eur. J.* **2013**, *19*, 2180-2184.
8. N. A. Setterholm, F. E. McDonald, *J. Org. Chem.* **2018**, *83*, 6259-6274.
9. L. M. Geary, J. C. Leung, M. J. Krische, *Chem. Eur. J.* **2012**, *18*, 16823-16827.
10. F. Cambeiro, S. López, J. A. Varela, C. Saá, *Angew. Chem., Int. Ed.* **2014**, *53*, 5959-5963.
11. S. Bräse, H. Wertal , D. Frank, D. Vidović, A. de Meijere, *Eur. J. Org. Chem.* **2005**, 4167-4178.
12. I. Matsuda, K.-i. Komori, K. Itoh, *J. Am. Chem. Soc.* **2002**, *124*, 9072-9073.
13. A. G. Campaña, N. Fuentes, E. Gómez-Bengoa, C. Mateo, J. E. Oltra, A. M. Echavarren, J. M. Cuerva, *J. Org. Chem.* **2007**, *72*, 8127-8130.
